# 4-Arylthieno[2,3-*b*]pyridine-2-carboxamides Are a New Class of Antiplasmodial Agents

**DOI:** 10.3390/molecules25143187

**Published:** 2020-07-13

**Authors:** Sandra I. Schweda, Arne Alder, Tim Gilberger, Conrad Kunick

**Affiliations:** 1 Institut für Medizinische und Pharmazeutische Chemie, Technische Universität Braunschweig, 38106 Braunschweig, Germany; sandra.schweda@tu-braunschweig.de; 2 Zentrum für Pharmaverfahrenstechnik (PVZ), Technische Universität Braunschweig, Franz-Liszt-Straße 35A, 38106 Braunschweig, Germany; 3 Centre for Structural Systems Biology, 22607 Hamburg, Germany; alder@bnitm.de (A.A.); gilberger@bnitm.de (T.G.); 4 Bernhard Nocht Institute for Tropical Medicine, 20359 Hamburg, Germany; 5 Department of Biology, University of Hamburg, 20146 Hamburg, Germany

**Keywords:** anti-malarial drugs, antiplasmodial, malaria, *Pf*GSK-3, *Plasmodium falciparum*, thieno[2,3-*b*]pyridine, Thorpe-Ziegler reaction

## Abstract

Malaria causes hundreds of thousands of deaths every year, making it one of the most dangerous infectious diseases worldwide. Because the pathogens have developed resistance against most of the established anti-malarial drugs, new antiplasmodial agents are urgently needed. In analogy to similar antiplasmodial ketones, 4-arylthieno[2,3-*b*]pyridine-2-carboxamides were synthesized by Thorpe-Ziegler reactions. In contrast to the related ketones, these carboxamides are only weak inhibitors of the plasmodial enzyme *Pf*GSK-3 but the compounds nevertheless show strong antiparasitic activity. The most potent representatives inhibit the pathogens with IC_50_ values in the two-digit nanomolar range and exhibit high selectivity indices (>100).

## 1. Introduction

Malaria is a tropical infectious disease caused by unicellular parasites of the genus *Plasmodium* which is transmitted by the bite of the Anopheles mosquito. The majority of malaria cases occur in African regions where *Plasmodium falciparum* is the most prevalent [1]. This pathogen causes *Malaria tropica*, the most dangerous form of the infection [2]. In 2018, there were 228 million cases of malaria worldwide, 405,000 of which were fatal. While the number of malaria infections decreased significantly in the years 2000–2015, this development unfortunately came to a standstill in recent years [2,3,4].

Because resistance to all older drugs against malaria occurs worldwide [5], the World Health Organisation (WHO) in 2001 recommended artemisinin combination therapy as the first treatment option for *Malaria tropica* [6]. As early as 2006, individual reports of therapy failure under artemisinins in Thailand and West Cambodia were published [7,8,9]. Since then there have been more reports of artemisinin resistance [10,11,12,13].

Alternatives to artemisinins must therefore be developed as quickly as possible and with high priority. These new drugs should have a novel structural design and address previously unexploited biological targets in order to be suitable as combination partners for established antimalarial drugs as well as to avoid cross-resistance.

**Figure 1:**
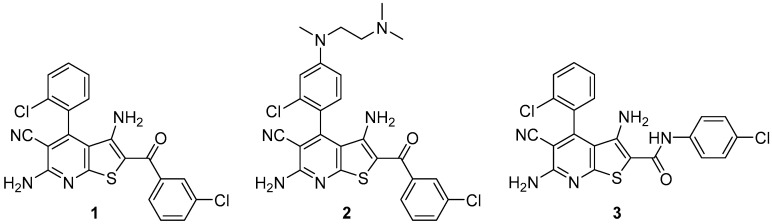
Thieno[2,3-*b*]pyridines with antiplasmodial activity.

In previous studies the antiplasmodial thieno[2,3-*b*]pyridine **1** was identified as a selective inhibitor of the plasmodial glycogen synthase kinase-3 (*Pf*GSK-3) (IC_50_ *Pf*GSK-3 = 0.48 µM) [14]. Since *Pf*GSK-3 is considered an essential enzyme for the asexual proliferation of the parasite, it was also postulated as a relevant target of antiplasmodial activity of **1** [15,16,17]. In subsequent studies it could be shown that the potency against the enzyme as well as against the pathogen can be increased by attachment of a basic structural element such as in compound **2** [18]. In the framework of the structural modifications, the thieno[2,3-*b*]pyridine-2-carboxamide **3** (Figure 1) was also synthesized, in which the keto group is replaced by a carboxylic acid amide structure [14].

In this study, we show that structure **3** is neither an inhibitor of plasmodial nor human GSK-3 but inhibits the proliferation of erythrocytic parasite forms much more strongly (IC_50_ = 199 nM) than the *Pf*GSK-3 inhibitor **1**. On the one hand, this observation raises doubts as to whether *Pf*GSK-3 inhibition is also responsible for the antiplasmodial activity of **1** and **2** and, on the other hand, justifies structural modifications of carboxamides such as **3** with the aim of optimizing antiplasmodial activity.

Therefore, the thieno[2,3-*b*]pyridine-2-carboxamides were modified to allow studies of structure-activity relationships in this class of compounds. On the one hand, the substitution pattern at the two phenyl rings of the parent structure **3** was varied. In addition, it was investigated whether the 5-cyano and the 6-amino group on the heterocyclic parent ring system are necessary for antiplasmodial activity or may be replaced by less polar elements. Furthermore, compounds were synthesized in which the phenyl ring of the amide component was replaced by aliphatic structural elements. Within the scope of these structural variations, compounds were identified which clearly exceeded the original structures **1** and **2** in antiplasmodial activity but no longer caused inhibition of the plasmodial enzyme *Pf*GSK-3.

## 2. Results and Discussion

### 2.1. Syntheses

**Scheme 1:**
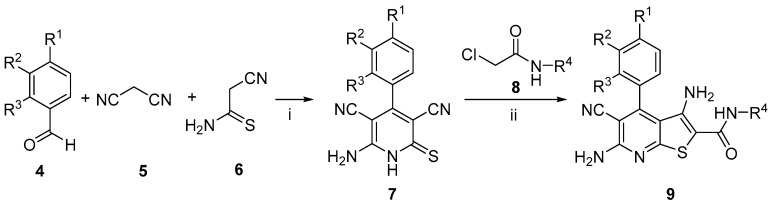
Syntheses procedures for the preparation of **9**. Reagents and conditions: (i) ethanol, piperidine, reflux, 3–6 h; (ii) 1. *N*,*N*-dimethylformamide, KOH (10%, 1 eq.), rt, 10–30 min; 2. KOH (10%, 1 eq.), 100 °C, 15 min–5 h.

According to established methods, the thioxo-1,2-dihydropyridines **7** were synthesized in a one-pot synthesis from an aromatic aldehyde (**4**), malononitrile (**5**) and 2-cyanothioacetamide (**6**) in the presence of catalytic amounts of piperidine [19,20]. **7** tend to decompose by oxidative dimerization and cannot be purified economically neither by chromatography nor crystallization [21,22]. The intermediates **7** were therefore alkylated directly with 2-chloroacetamide derivatives **8** in the presence of potassium hydroxide. By sequential addition of further potassium hydroxide, the desired thieno[2,3-*b*]pyridine-2-carboxamides **9** were formed from the intermediate thioethers by Thorpe-Ziegler cyclization (Scheme 1, Table 1) [23,24].

**Scheme 2:**
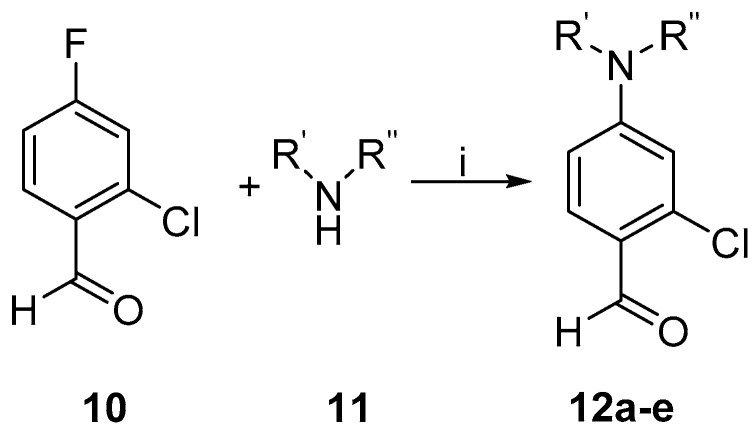
Syntheses procedure for the preparation of **12a**–**e**. Reagents and conditions: *N*,*N*-dimethylformamide, 2-chloro-4-fluorobenzaldehyde (**10**, 1 eq.), amine (**11**, 1.5 eq.), K_2_CO_3_, 100 °C, 5 h–14.5 h.

The aldehydes **4** needed as starting compounds were either commercially available or were synthesized from 2-chloro-4-fluorobenzaldehyde (**10**). For this purpose, **10** was converted with secondary amines **11** to 4-(*N*,*N*-dialkylamino)-2-chlorobenzaldehydes **12a**–**e** according to a method developed by Yongpruska et al. [25] (Scheme 2).

**Scheme 3:**
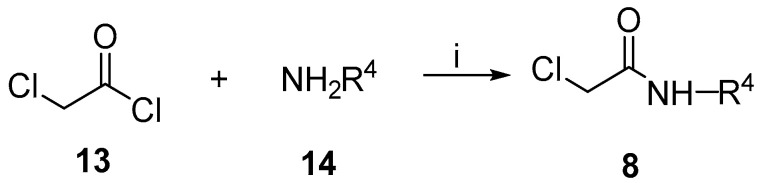
Syntheses of **8**. Reagents and conditions: dry dichloromethane, argon, amine (**14**, 1 eq.), K_2_CO_3_ (1.8 eq.), 2-choroacetyl chloride (**13**, 1.5 eq.), rt, 1 h, reflux, 1 h 10 min.

Aliphatic 2-chloro-*N*-alkylacetamide derivatives **8** were produced from 2-chloroacetyl chloride (**13**) and the aliphatic amines **14** in nucleophilic substitution reactions according to a modified procedure developed by Cho et al. (Scheme 3) [26].

**Scheme 4:**
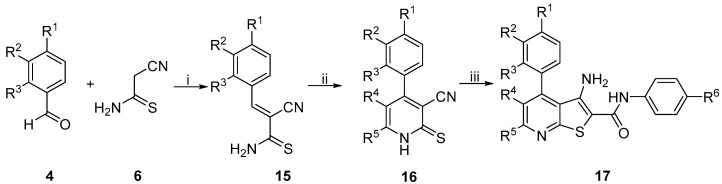
Syntheses of thieno[2,3-*b*]pyridine-2-carboxamides **17** with aliphatic or carbonyl substituents R^4^/R^5^. Reagents and conditions: (i) ethanol, triethylamine, 55 °C, 10 min–3 h; (ii) carbonyl component (R^4^-CH_2_-C(O)-R^5^), 1,4-dioxane, piperidine, 80 °C, 1.75 h–4 h; (iii) 1. *N*,*N*-dimethylform amide, KOH (10%, 1 eq.), rt, 10–30 min; 2. KOH (10%, 1 eq.), 100 °C, 15 min–2 h.

In cases where the 5-cyano-6-amino partial structure was replaced by aliphatic substituents or by methyl or ethyl ester structures, a two-step synthesis was used to produce 2-thioxopyridine **16**. First, **4** and **6** were reacted in a Knoevenagel reaction to form a 3-aryl-2-cyanoprop-2-enethioamide **15** [27]. Subsequently, piperidine-initiated Michael addition of a carbonyl component followed by intramolecular cyclization and oxidation yielded **16** [28]. These derivatives were converted afterwards to **17** (Scheme 4, Table 2). Boc protective groups and *tert*-butyl groups were cleaved off under argon atmosphere in dried dichloromethane in the presence of trifluoroacetic acid.

### 2.2. Biological Activity

All synthesized thieno[2,3-*b*]pyridine-2-carboxamides (Table 1 and Table 2) were initially tested for their effects on the viability of erythrocytic forms of the pathogen *P. falciparum* (strain 3D7, at 3 and 0.3 µM) and the initially assumed biological target structure *Pf*GSK-3 (at 10 µM). Additionally, the cytotoxicity of all compounds was determined on HEK293T cells at 30 µM (Table 3). For selected compounds the inhibition of the human *Hs*GSK-3 orthologue product was also investigated (Table 4) and the IC_50_ values for the viability of the parasites were determined (Table 5).

In the series of 3,6-diamino-5-cyanothieno[2,3-*b*]pyridine-2-carboxamides, consistent relationships between molecular structure and antiplasmodial activity were found. Nearly all compounds in a concentration of 3 µM reduced the viability of the pathogens to below 10% of the controls. Exceptions were the compounds in which the *N*-phenyl substituent was chloro-substituted in the ortho position (**9b**, **9c**, **9f**, **9t**, **9u**, **9ad**) or in which the *N*-phenyl substituent was replaced by an aliphatic radical (**9j**, **9k**, **9l**, **9m**, **9n**, **9o**).

The prerequisite for good antiplasmodial activity is obviously an aromatic substituent on the nitrogen atom of the carboxylic acid amide structure, the spatial orientation of which is not disturbed by ortho-substitution. The substitution pattern at the 4-aryl substituent of the heterocyclic basic structure, however, had little influence on the antiplasmodial activity. Both compounds with either a meta-substituted 4-phenyl ring (**9a**–**9i**) as well as derivatives with para-amino/ortho-chloro substitution (**9p**–**9af**) showed significant inhibition of pathogen viability at 3 µM concentration (<10% of the controls).

Also compounds that were linked to alkyl or carbonyl substituents in the 5- and 6-position of the thieno[2,3-*b*]pyridine scaffold (**17a**–**17l**) showed more than 90% inhibition of the plasmodial pathogens at a concentration of 3 µM. Exceptions were the carboxylic acids **17i** and **17j**, which in contrast to the analogous *tert*-butyl esters **17g** and **17h** showed no (**17j**) or only low (**17i)** antiplasmodial activity at the concentration investigated.

The antiplasmodial ketones **1** and **2**, which are related in structure to the carboxylic acid amides presented here and which served as the starting point for our investigations, had been shown to be potent inhibitors of the plasmodial enzyme *Pf*GSK-3. Therefore, the inhibition of this enzyme was supposed to be an antiplasmodial mechanism of action of this substance class [14,18]. However, the testing of carboxylic acid amides on *Pf*GSK-3 showed that at a concentration of 10 µM most compounds showed no or only low inhibitory activity. Exceptions were three substances with aliphatic substituents on the nitrogen atom of the carboxylic acid amide structure **9j**, **9m**, **9n**, which reduced the *Pf*GSK-3 activity to below 40% of the controls. Inhibition by these compounds was selective for the plasmodial enzyme, as shown by comparative experiments with the orthologue human *Hs*GSK-3 enzyme (Table 5). In contrast to most of the other compounds in this study, which showed strong antiplasmodial activity in the absence of *Pf*GSK-3 activity, the opposite was true for the three aliphatic carboxamides **9j**, **9m**, **9n**. This finding makes it very doubtful that *Pf*GSK-3 is the biological target structure responsible for antiplasmodial activity in this class of drugs.

For particularly potent compounds, IC_50_ values for the antiplasmodial activity were determined (Table 5). The results for four representatives were in the two-digit nanomolar range (**17c**, **17f**, **17g**, **17l**). These carboxylic acid amides, in which the 5-cyano-6-amino substitution pattern is replaced by other elements, exceed the antiplasmodial activity of the analogous ketones such as **1** [14] and **2 [18]** by 1–2 orders of magnitude.

It was shown that with few exceptions, the compounds in this study had only a low toxicity for the human HEK cells investigated for comparison. At a concentration of 30 µM the viability of these cells was reduced by only four compounds to less than 50% of the controls (**9i**, **9o**, **17d**, **17h**). In contrast, the selectivity indices for the particularly strongly antiplasmodic representatives of this study were well over 100 (Table 5).

The following structure-activity relationships can be derived from these results: The keto function of compounds like **1** and **2** can be replaced by a carboxamide function without loss of antiplasmodial activity. However, the inhibitory activity for *Pf*GSK-3 largely disappears through this modification, which is why the enzyme is unlikely to represent the relevant biological target for this class of compounds. Furthermore, in the group of these carboxamides there is no need for the presence of an ortho-halogen substituent at the 4-phenyl residue of the heterocyclic parent scaffold, as had been postulated for the analogous ketone derivatives (e.g., **1** and **2**) [14,18]. Eventually, the 5-cyano-6-amino substitution pattern on the heterocyclic parent scaffold is not necessary for antiplasmodial activity. Instead, substances such as **17c**, **17f**, **17g** and **17l**, in which the 5-cyano-6-amino substituents have been replaced by other elements, are the most potent representatives in this group. The aromatic substituent on the nitrogen atom of the carboxamide structure cannot be replaced by aliphatic elements without a drastic loss of the antiplasmodial activity, although these compounds still cause significant *Pf*GSK-3 inhibition. This is a further indication that the *Pf*GSK-3 inhibition does not contribute to the antiplasmodial effect in this substance class.

## 3. Materials and Methods

### 3.1. General Information

Structures of all test compounds are depicted in Figure S1. The starting materials and reagents were purchased from Acros Organics (Geel, Belgium), Alfa Aesar (Karlsruhe, Germany), Sigma-Aldrich (Steinheim, Germany), Fluorochem (Derbyshire, UK) and Enamine (Riga, Latvia). All reagents and solvents were used without further purification unless otherwise stated. Anhydrous dichloromethane was used if indicated and was dried according to published methods.[29] Silica gel (40–63 µm) was used for purification by column chromatography. Reaction monitoring was performed using thin layer chromatography (TLC): Polygram SIL G/UV_254_, 0.2 mm silica gel 60, 40 × 80 mm (Macherey-Nagel, Düren, Germany), visualization by UV light (254 nm, 366 nm). The melting points (m.p.) were detected in open-glass capillaries on an electric variable heater (Electrothermal IA 9200, Bibby Scientific, Stone, UK). The infrared spectra were recorded on a Thermo Nicolet FT-IR 200 spectrometer (Thermo Nicolet, Madison, WI, USA) using KBr pellets or NaCl windows. ^1^H-NMR spectra and ^13^C-NMR spectra were recorded on Bruker Avance III 400, Bruker Avance II 600 or Bruker Avance III HD 500 spectrometers (Bruker Biospin, Rheinstetten, Germany) (at the NMR laboratories of the Chemical Institutes of the Technische Universität Braunschweig, Germany) in DMSO-*d*_6_. Chemical shifts are reported as parts per million (ppm) relative to tetramethylsilane as internal standard (δ = 0 ppm). Signals in ^13^C-NMR spectra were assigned based on results of ^13^C-DEPT135 experiments. Electron ionization (EI) mass spectra were recorded on a Finnigan-MAT 95 (Thermo Finnigan, Bremen, Germany), (EI) MS: ionization energy 70 eV. Accurate measurements were performed according to the peakmatch method using perfluorokerosene (PFK) as an internal mass reference. Electron spray ionization (ESI) mass spectra were recorded on LTQ-Orbitrap Velos ThermoFisher Scientific (Bremen, Deutschland). Tetradecyltrimethylammonium bromide was used as internal standard. Compounds were dissolved in methanol; concentrations were about 50 μg/mL. The flow rate was 1 μg/min, spray voltage: pos. mode 2.3–2.8 kV, neg. mode: 1.7–2.5 kV (experiments were conducted at the department of mass spectrometry of the Chemical Institutes, TU Braunschweig, Germany). The calculated and found mass data are reported. Atmospheric pressure chemical ionization (APCI) and electrospray ionization (ESI) spectra were determined with an expression^L^ CMS spectrometer (Advion, Ltd., Harlow, UK). The ESI and APCI measurements were performed by dissolution of the compound in methanol and via direct injection. The elemental analyses were performed on a CE Instruments Flash EA^®^ 1112 Elemental Analyzer (Thermo Quest, San Jose, CA, USA). If the elemental analysis was inconclusive, a HRMS (described above) study was performed. Purity was determined using high performance liquid chromatography (HPLC) methods with isocratic or gradient elution. All compounds tested in biological systems had purity ≥ 95%. The following HPLC devices and settings were used: System 1: Merck Hitachi Elite LaChrom system (Hitachi High Technologies Corporation, Tokyo, Japan); the threshold of the integration method was set to 1000; (diode array detector (DAD): L-2450; pump: L-2130; autosampler: L-2200; organizer box: L-2000); System 3: Merck Hitachi Elite LaChrom system (Hitachi High Technologies Corporation, Tokyo, Japan); the threshold of the integration method was set to 50; detector: L-2400; pump: L-2130; autosampler: L-2200; organizer box: L-2000); flow rate: 1.000 mL/min; detection wavelength: 254 nm and 280 nm (isocratic), 254 nm (gradient); overall run time: 20 min (gradient), 15 min (isocratic) or 25 min (isocratic for **9ae** and **9af**); AUC, % method; t_ms_ = retention time, t_m_ = dead time related to DMSO. An acetonitrile/water mixture was used for gradient elution (0–2 min: 10% ACN; 2–12 min: 10%–90% ACN (linear) 12–20 min: 90% ACN). For isocratic elution, various acetonitrile/water or acetonitrile/buffer mixtures were used. Absorption maxima (λ_m__ax_) were extracted from the UV spectra recorded by the DAD detector in the peak maxima during HPLC runs. For the measurements of the purity of **9n**, **9r**, **9ac**, **9af** and **9ag**, a triethylamine/triethylammonium sulfate buffer (pH 2.7) was used. For its preparation, triethylamine (20 mL) was dissolved in water (980 mL) and sodium hydroxide (242 mg) was added. The pH was adjusted to 2.7 by adding concentrated H_2_SO_4_ dropwise. The column was equilibrated with ACN/buffer (10/90) for 40 min. Subsequently, the desired ACN/buffer ratio (range 10/90–60/40) was adjusted. Preparative high-performance liquid chromatography (HPLC) was performed on LaPrep (Merck, Darmstadt): LaPrep P110 preparative HPLC pump; sample loop (Knauer, Berlin); LaPrep P216 fraction collector; LaPrep P311 spectral photometer. Column tube: Length 125 mm, inner diameter 25 mm, column packed with self-fill level NW25, column material: LiChrospher^®^ 100 RP-18, 12 μm (Merck, Darmstadt). Sample preparation: approx. 100 mg substance is dissolved in 5 mL DMSO and injected into the sample loop. Eluent: ACN/H_2_O 70:30, flow rate: 40 mL/min, detection at 254 nm. Because phase transmission was observed during m.p. determination, melting points of **9k** and **9m** were measured by differential scanning calorimetry. The DSC spectra were recorded on DSC1 STAR^e^ System, Mettler Toledo (Columbus, OH, USA). The onset temperatures are given.

### 3.2. Syntheses and Characterization of ***7***, ***8***, ***9***, ***12***, ***15***, ***16*** and ***17***

#### 3.2.1. General Procedure for the Syntheses of 4-(*N*,*N*-Dialkylamino)-2-chlorobenzaldehydes **12** (Procedure A)

2-Chloro-4-fluorobenzaldehyde (**10**, 1 equivalent) and potassium carbonate (1.6 equivalents) are dissolved in DMF and the corresponding amine (**11**, 1.5 equivalents) is added. The mixture is heated at 100 °C for 3 h 30 min–8 h. Finally, ice water (20 mL) is added, forming a yellow-brownish precipitate. The precipitate is filtered off and the product is purified by column chromatography or crystallization.

#### 3.2.2. General Procedure for the Syntheses of 6-Amino-4-aryl-2-thioxo-1,2-dihydropyridine-3,5-dicarbonitriles **7** (Procedure B)

A mixture of malononitrile (**5**, 1 equivalent), 2-cyanothioacetamide (**6**, 1 equivalent) and the corresponding aromatic aldehyde (**4** or **12**, 1 equivalent) is dissolved in ethanol. After addition of catalytic amounts of piperidine, the mixture is refluxed for 3–6 h. Afterwards, the solvent is evaporated under reduced pressure. The brown oil obtained is mixed successively with water (10 mL), acetic acid (15 drops) and dichloromethane (2 mL) and stored at 4 °C for 15–30 min. The precipitate is filtered off and the product is used directly without further purification for the synthesis of the corresponding thieno[2,3-*b*]pyridine. If no precipitate is formed, the further work up is performed as indicated in the specific synthesis procedure.

#### 3.2.3. General Procedure for the Syntheses of 3-Aryl-2-cyanoprop-2-enthioamides **15** (Procedure C)

The aromatic aldehyde (**4** or **12**, 1 equivalent) is dissolved in ethanol. Then 2-cyanothioacetamide (**6**, 1 equivalent) and catalytic amounts of triethylamine (50 µL) are added. The reaction mixture is stirred at 55 °C for 10 min–2 h 30 min. The solvent is evaporated under reduced pressure and the residue is purified by column chromatography if necessary.

#### 3.2.4. General Procedure for the Syntheses of Dihydropyridines **16** with Alkyl Substituents (Procedure D)

The corresponding carbonyl compound (1 equivalent) is dissolved in 1,4-dioxane (1 mL) and the mixture is heated to 80 °C. Then catalytic amounts of piperidine (50 µL) and the respective 3-aryl-2-cyanoprop-2-enthioamide (**15**, 1 equivalent) are added in portions. The reaction is monitored by TLC. Afterwards, the solvent is evaporated under reduced pressure and the residue is purified by crystallization or column chromatography.

#### 3.2.5. General Procedure for the Syntheses of Aliphatic Amides **8** (Procedure E)

The corresponding amine (**14**, 1 equivalent) is dissolved in dried dichloromethane (8 mL) and mixed with K_2_CO_3_ (1.8 equivalents). The reaction vessel is charged with argon. 2-Chloroacetyl chloride (**13**, 1.5 equivalents) is added in 100 µL steps every 15 min and the mixture is stirred for 1 h at room temperature. Subsequently, the mixture is refluxed for 1 h 10 min. Then water (15 mL) is added and the mixture is stirred for another 15 min at room temperature. The mixture is extracted with dichloromethane (3 × 50 mL). Subsequently, the combined organic layers are dried over sodium sulfate and evaporated under reduced pressure. A clear liquid is obtained, which becomes solid upon scratching with a glass rod.

#### 3.2.6. General Procedure for the Syntheses of 4-Arylthieno[2,3-*b*]pyridine-2-carboxamides **9** and **17** (Procedure F)

The corresponding thioxo-1,2-dihydropyridine (**7** or **16**, 1 equivalent) is dissolved in DMF and aqueous potassium hydroxide solution (10%, 1 equivalent) is added. The 2-chloroacetamide derivative (**8**, 1 equivalent) is added and the mixture is stirred for 10–30 min at room temperature. Finally, further aqueous potassium hydroxide solution (10%, 1 equivalent) is added and the mixture is stirred at 100 °C until the reaction is finished. Upon addition of ice water (20 mL) a yellow-brownish precipitate is formed which is filtered off and washed successively with water, ethanol and petroleum ether. If no precipitate is formed, further work up is performed as indicated in the specific synthesis procedure. Column chromatography and/or crystallization are used for purification.

#### 3.2.7. General Method for Cleavage of Boc-protecting Groups (Procedure G)

The Boc-protected thieno[2,3-*b*]pyridine (**9q** or **9ae**, 0.1 equivalent) is dissolved in dried dichloromethane (8 mL) in an argon atmosphere. Trifluoroacetic acid (3 mL) is added and the mixture is stirred for 30 min–1 h at room temperature. The solvent is evaporated under reduced pressure. The oily brown residue is dissolved in propan-2-ol (3 mL). After addition of a 5–6 M hydrochloric acid solution (0.1 equivalent) in propan-2-ol a precipitate is formed which is filtered off. In the case of **9ae**, the mixture is stored at 4 °C for three days until a precipitate is formed.

#### 3.2.8. General Method for the Cleavage of the *tert*-Butyl esters **17i** and **17j** (Procedure H)

The corresponding *tert*-butyl ester **17i** or **17j** (1 equivalent) is stirred in dried dichloromethane (2 mL) and trifluoroacetic acid (2 mL) in an argon atmosphere at room temperature for 20–24 h. Afterwards, the solvent is evaporated under reduced pressure. The product is extracted with ethyl acetate (50 mL) and washed with water (3 × 50 mL). The combined organic layers are washed with saturated sodium chloride solution (30 mL), dried over sodium sulfate and the solvent is evaporated under reduced pressure. Purification is performed by crystallization from ethanol (70% *v*/*v*).

#### 3.2.9. Synthesis Procedures for Individual Compounds

*2-Chloro-4-(pyrrolidin-1-yl)benzaldehyde***12a***(KuSaSch045):* According to Procedure A from 2-chloro-4-fluorobenzaldehyde (**10**, 635 mg, 4.00 mmol), pyrrolidine (493 µL, 6.00 mmol) and K_2_CO_3_ (898 mg, 6.50 mmol) for 5 h 15 min at 100 °C. After purification by column chromatography (petroleum ether/ethyl acetate 9:1) a yellow-orange solid (719 mg, 86%) was obtained.

M.p.: 86–91 °C (Lit.: 90–92 °C [21]; 78–80 °C [25]); IR (KBr): ῦ_max_ 2978 cm^−1^, 2958 cm^−1^, 2916 cm^−1^, 2861 cm^−1^ (CH aliphatic), 1657 cm^−1^ (C = O); ^1^H-NMR (500 MHz, DMSO-*d*_6_) δ (ppm) = 1.92–2.02 (m, 4H, 2 CH_2_), 3.33–3.39 (m, 4H, 2 CH_2_), 6.57–6.64 (m, 2H, ArH), 7.68 (d, *J* = 8.8 Hz, 1H, ArH), 10.02 (d, *J* = 0.8 Hz, 1H, CHO); ^13^C-NMR (126 MHz, DMSO) δ (ppm) = 24.8 (2C), 47.5 (2 C) (CH_2_), 110.8, 111.2, 130.8, 186.6 (CH), 119.4, 138.6, 151.8 (C); C_11_H_12_ClNO (209.67); calcd. C 63.01, H 5.77, N 6.68, found C 63.36, H 5.82, N 6.39; MS (APCI pos.): *m*/*z* (%) = 210.0 (100) [M + H]^+^; HPLC (grad.): 97.8% at 254 nm, t_m_ = 1.1 min, t_ms_ = 11.7 min (system 3); λ_max_: 206 nm, 254 nm, 341 nm, 358 nm.

*2-Chloro-4-{[2-(dimethylamino)ethyl](methyl)amino}benzaldehyde***12b***(KuSaSch046):* According to Procedure A from 2-chloro-4-fluorobenzaldehyde (**10**, 686 mg, 4.33 mmol), *N*′,*N*′,*N*′′-trimethylethane-1,2-diamine (840 µL, 6.52 mmol) and K_2_CO_3_ (956 mg, 6.93 mmol) for 5 h 30 min at 100 °C. After purification by column chromatography (ethanol/triethylamine 1:0.05) a brown oil (1.04 g, 100%) was obtained.

IR (NaCl): ῦ_max_ 2971 cm^−1^, 2943 cm^−1^, 2911 cm^−1^ (CH aliphatic), 1668 cm^−1^ (C=O); ^1^H-NMR (600 MHz, DMSO-*d*_6_) δ (ppm) = 2.20 (s, 6H, 2 CH_3_), 2.40 (t, *J* = 6.9 Hz, 2H, CH_2_), 3.06 (s, 3H, CH_3_), 3.56 (t, *J* = 6.9 Hz, 2H, CH_2_), 6.71–6.75 (d, *J* = 2.5 Hz, 1H, ArH), 6.76–6.81 (m, 1H, ArH), 7.69 (d, *J* = 8.9 Hz, 1H, ArH), 10.04 (d, *J* = 0.8 Hz, 1H, CHO); ^13^C NMR (151 MHz, DMSO-*d_6_*) δ (ppm) = 38.4, 45.4 (2C) (CH_3_); 49.5, 55.7 (CH_2_); 110.3, 111.0, 130.7, 186.6 (CH); 119.7, 138.8, 153.6 (CH); C_12_H_17_ClN_2_O (240.73); calcd. C 59.87, H 7.12, N 11.64, found C 60.07, H 7.34, N 11.38; MS (APCI pos.): *m*/*z* (%) = 254.1 (100) [M + 13]^+^, 241.1 (162) [M + H]^+^, 196.0 (12) [M − 44.7]^+^; HPLC (isocr.): 99.8% at 254 nm und 99.4% at 280 nm, t_m_ = 1.2 min, t_ms_ = 8.7 min (ACN/buffer 10:90) (system 1); λ_max_: 255 nm, 326 nm, 352 nm.

*2-Chloro-4-morpholinobenzaldehyde***12c***(KuSaSch047):* According to Procedure A from 2-chloro-4-fluorobenzaldehyde (**10**, 794 mg, 5.01 mmol), morpholine (650 µL, 7.54 mmol) and K_2_CO_3_ (1.11 g, 8.01 mmol) for 6 h 30 min at 100 °C. A yellow solid (923 mg, 82%) was obtained.

M.p.: 93–94 °C (Lit.: 85–89 °C [21], 87 °C [25]); IR (KBr): ῦ_max_ 2962 cm^−1^, 2873 cm^−1^, 2831 cm^−1^ (CH aliphatic), 1657 cm^−1^ (C=O); ^1^H-NMR (500 MHz, DMSO-*d*_6_) δ (ppm) = 3.36–3.42 (m, 4H, 2 CH_2_), 3.68–3.74 (m, 4H, 2 CH_2_), 6.98–7.05 (m, 2H, ArH), 7.68–7.73 (m, 1H, ArH), 10.08 (s, 1H, CHO); ^13^C-NMR (126 MHz, DMSO-*d_6_*) δ (ppm) = 46.2 (2C), 65.6 (2C) (CH_2_), 112.0, 113.2, 130.7, 187.2 (CH), 121.6, 138.6, 155.0 (C); C_11_H_12_ClNO_2_ (225.67); calcd. C 58.55, H 5.36, N 6.21, found C 58.34, H 5.01, N 6.18; MS (APCI pos.): *m*/*z* (%) = 226.0 (100) [M + H]^+^, 184.0 (2) [M − 42]^+^; HPLC (grad.): 99.5% at 254 nm, t_m_ = 1.1 min, t_ms_ = 9.9 min (system 3); λ_max_: 204 nm, 252 nm, 326 nm.

*tert-Butyl {2-[(3-chloro-4-formylphenyl)(methyl)amino]ethyl}carbamate***12d***(KuSaSch052):* According to Procedure A from 2-chloro-4-fluorobenzaldehyde (**10**, 396 mg, 2.50 mmol), *tert*-butyl [2-(methyl-amino)ethyl]carbamate (797 mg, 3.75 mmol) and K_2_CO_3_ (552 mg, 4 mmol) for 5 h at 100 °C. After purification by column chromatography (toluene/ethyl acetate 2:1) a yellow solid (750 mg, 95%) was obtained.

M.p.: 90–92 °C (Lit.: 25–27 °C [21]); IR (KBr): ῦ_max_ 3368 cm^−1^ (NH), 3014 cm^−1^ (CH aromatic), 2989 cm^−1^, 2967 cm^−1^, 2903 cm^−1^, 2856 cm^−1^ (CH aliphatic), 1674 cm^−1^ (C=O); ^1^H-NMR (500 MHz, DMSO-*d*_6_) δ (ppm) = 1.32 (s, 9H, 3 CH_3_), 3.01 (s, 3H, CH_3_), 3.12 (q, *J* = 6.2 Hz, 2H, CH_2_), 3.49 (t, *J* = 6.3 Hz, 2H, CH_2_), 6.73–6.81 (m, 2H, ArH and NH), 6.93 (t, *J* = 6.0 Hz, 1H, ArH), 7.65 (d, *J* = 8.9 Hz, 1H, ArH), 10.02 (d, *J* = 0.6 Hz, 1H, CHO); ^13^C-NMR (126 MHz, DMSO-*d_6_*) δ (ppm) = 28.0 (3C), 38.0 (CH_3_), 37.2, 50.6 (CH_2_), 110.3, 111.0, 119.7, 130.6, 186.6 (CH), 77.6, 138.7, 154.0, 155.6 (C); C_15_H_21_ClN_2_O_3_ (312.79); calcd. C 57.60, H 6.77, N 8.96, found C 57.48, H 6.88, N 8.85; MS (APCI pos.): *m*/*z* (%) = 257.1 (100) [M − 56]^+^, 170.0 (32) [M − 143]^+^, 88.1 (28) [M − 225]^+^; HPLC (grad.): 99.7% at 254 nm, t_m_ = 1.1 min, t_ms_ = 11.0 min (system 3); λ_max_: 215 nm, 255 nm, 327 nm, 358 nm.

*tert-Butyl 4-(3-chloro-4-formylphenyl)piperazine-1-carboxylate***12e***(KuSaSch039):* According to Procedure A from 2-chloro-4-fluorobenzaldehyde (**10**, 674 mg, 4.25 mmol), *tert*-butyl piperazine-1-carboxylate (1.19 g, 6.38 mmol) and K_2_CO_3_ (938 mg, 6.80 mmol) for 6 h 30 min at 100 °C. Then ice water (50 mL) was added and stirring was continued for 30 min at room temperature. The suspension was stored at 4 °C for 12 h and the product was filtered off. The product was dried under reduced pressure at 40 °C for 8 h. A yellow solid (1.38 g, 100%) was obtained.

M.p.: 94–104 °C (Lit.: 95–102 °C [21]); IR (KBr): ῦ_max_ 1689 cm^−1^ (C=O); ^1^H-NMR (500 MHz, DMSO-d_6_) δ (ppm) = 1.40–1.45 (s, 9H, 3 CH_3_), 3.42–3.48 (m, 8H, CH_2_), 6.99–7.00 (m, 2H, ArH), 7.68–7.71 (m, 1H, ArH), 10.04–10.07 (s, 1H, aldehyde); ^13^C-NMR (126 MHz, DMSO-*d_6_*) δ (ppm) = 27.9 (3C), 45.7 (2C) (CH_2_) (two further secondary carbon signals were not detectable after 512 scans), 112.1, 113.2, 130.8, 187.1 (CH), 79.1, 121.3, 138.7, 153.7, 154.5 (C); C_16_H_21_ClN_2_O_3_ (324.12); calcd. C 59.17, H 6.52, N 8.62, found C 59.49, H 6.58, N 8.49; HPLC (grad.): 98.8% at 254 nm, t_m_ = 1.1 min, t_ms_ = 12.1 min (system 3), 205 nm, 253 nm, 340 nm.

*6-Amino-4-(3-fluorophenyl)-2-thioxo-1,2-dihydropyridine-3,5-dicarbonitrile***7a***(KuSaSch020):* According to Procedure B from 3-fluorobenzaldehyde (298 mg, 2.41 mmol), malononitrile (**5**, 136 mg, 2.05 mmol) and 2-cyanothioacetamide (**6**, 200 mg, 2.00 mmol) in ethanol (8 mL) and piperidine (100 μL, 1.01 mmol) for 3 h 30 min. A brown mass (463 mg, 86%) was filtered off. Due to the material′s sensitivity to oxidation, it was used without further purification for the subsequent synthesis of the corresponding thieno[2,3-*b*]pyridine.

*6-Amino-4-(3-chlorophenyl)-2-thioxo-1,2-dihydropyridine-3,5-dicarbonitrile***7b***(KuSaSch008):* According to Procedure B from 3-chlorobenzaldehyde (1.41 mg, 10.1 mmol), malononitrile (**5**, 663 mg, 10.0 mmol) and 2-cyanothioacetamide (**6**, 1.00 g, 9.98 mmol) in ethanol (30 mL) and piperidine (500 μL, 5.00 mmol) for 3 h. A brown mass (758 mg, 26%) was filtered off. Due to the material′s sensitivity to oxidation, it was used without further purification for the subsequent synthesis of the corresponding thieno[2,3-*b*]pyridine.

*6-Amino-2-thioxo-4-(3-methylphenyl)-1,2-dihydropyridine-3,5-dicarbonitrile***7c***(KuSaSch030):* According to Procedure B from 3-methylbenzaldehyde (627 mg, 5.21 mmol), malononitrile (**5**, 355 mg, 5.37 mmol) and 2-cyanothioacetamide (**6**, 502 mg, 5.01 mmol) in ethanol (20 mL) and piperidine (250 μL, 2.53 mmol) for 4 h. A brown mass (982 mg, 74%) was filtered off. Due to the material′s sensitivity to oxidation, it was used without further purification for the subsequent synthesis of the corresponding thieno[2,3-*b*]pyridine.

*tert-Butyl 4-[4-(6-amino-3,5-dicyano-2-thioxo-1,2-dihydropyridin-4-yl)-3-chlorophenyl]piperazine-1-carboxylate***7d***(KuSaSch040):* According to Procedure B, tert-butyl 4-(3-chloro-4-formylphenyl)piperazine-1-carboxylate (**12e**, 432 mg, 1.33 mmol), malononitrile (**5**, 137 mg, 2.10 mmol) and 2-cyanothioacetamide (**6**, 210 mg, 2.10 mmol) were refluxed in ethanol (8 mL) and piperidine (100 µL, 1.01 mmol) for 4 h 30 min. After water (20 mL), acetic acid (15 drops) and dichloromethane (2 mL) were added, no precipitate was formed. The product was extracted from the mixture with dichloromethane (3 × 50 mL). The organic layer was washed with saturated sodium chloride solution (50 mL) and dried over sodium sulfate. The solvent was evaporated under reduced pressure. A brown, viscous oil (951 mg, 152%) was obtained. Due to the material′s sensitivity to oxidation, it was used without further purification for the subsequent synthesis of the corresponding thieno[2,3-b]pyridine.

*6-Amino-4-(2-chloro-4-morpholinophenyl)-2-thioxo-1,2-dihydropyridine-3,5-dicarbonitrile***7e***(KuSaSch048):* According to Procedure B from 2-chloro-4-morpholinobenzaldehyde (**12c**, 785 mg, 3.48 mmol), malononitrile (**5**, 242 mg, 3.67 mmol) and 2-cyanothioacetamide (**6**, 353 mg, 3.53 mmol) in ethanol (14 mL) and piperidine (175 µL, 1.77 mmol) for 5 h 30 min. A red-brown mass (1.18 g, 91%) was filtered off. Due to the material′s sensitivity to oxidation, it was used without further purification for the subsequent synthesis of the corresponding thieno[2,3-*b*]pyridine.

*6-Amino-4-[2-chloro-4-(pyrrolidin-1-yl)phenyl]-2-thioxo-1,2-dihydropyridine-3,5-dicarbonitrile***7f***(KuSaSch049):* According to Procedure B from 2-chloro-4-(pyrrolidin-1-yl)benzaldehyde (**12a**, 419 mg, 2.00 mmol), malononitrile (**5**, 137 mg, 2.08 mmol) and 2-cyanothioacetamide (**6**, 200 mg, 2.00 mmol) in ethanol (10 mL) and piperidine (100 µL, 1.01 mmol) for 5 h 15 min. After water (20 mL), acetic acid (15 drops) and dichloromethane (2 mL) were added, no precipitate was formed. The product was extracted with dichloromethane (3 × 50 mL). The combined organic layers were washed with saturated sodium chloride solution (50 mL) and dried over sodium sulfate. The solvent was evaporated under reduced pressure. A red-brown mass (829 mg, 116%) was obtained. Due to the material′s sensitivity to oxidation, it was used without further purification for the subsequent synthesis of the corresponding thieno[2,3-*b*]pyridine.

*tert-Butyl (2-{[4-(6-amino-3,5-dicyano-2-thioxo-1,2-dihydropyridin-4-yl)-3-chlorophenyl]-(methyl)-amino}ethyl)carbamate***7g***(KuSaSch053):* According to Procedure B from *tert*-butyl {2-[(3-chloro-4-formylphenyl)(methyl)amino]ethyl}carbamate (**12d**, 363 mg, 1.16 mmol), malononitrile (**5**, 77 mg, 1.2 mmol) and 2-cyanothioacetamide (**6**, 116 mg, 1.16 mmol) in ethanol (5 mL) and piperidine (100 µL, 1.01 mmol) for 6 h. An orange solid (490 mg, 92%) was filtered off. Due to the material′s sensitivity to oxidation, it was used without further purification for the subsequent synthesis of the corresponding thieno[2,3-*b*]pyridine.

*6-Amino-4-(2-chloro-4-{[2-(dimethylamino)ethyl](methyl)amino}phenyl)-2-thioxo-1,2-dihydropyridine-3,5-dicarbonitrile***7h***(KuSaSch080):* According to Procedure B from 2-chloro-4-{[2-(dimethylamino)-ethyl](methyl)amino}benzaldehyde (**12b**, 1.75 g, 7.29 mmol), malononitrile (**5**, 481 mg, 7.28 mmol) and 2-cyanothioacetamide (**6**, 730 mg, 7.29 mmol) in ethanol (5 mL) and piperidine (250 µL, 2.53 mmol) for 3 h. After water (20 mL), acetic acid (15 drops) and dichloromethane (2 mL) were added, the precipitate was filtered off. An orange-yellow solid (996 mg, 35%) was obtained by purification through column chromatography (dichloromethane/methanol/triethylamine 1:1:0.05). The product contained small amounts of the corresponding disulfide. Due to the material′s sensitivity to oxidation, it was used without further purification for the subsequent synthesis of the corresponding thieno[2,3-b]pyridine.

M.p.: Dec. starting at 231 °C; ^1^H-NMR (500 MHz, DMSO-*d*_6_) δ (ppm) = 2.67 (s, 6H, CH_3_), 2.98 (s, 3H, CH_3_), 3.01–3.04 (m, 2H, CH_2_), 3.65 (t, *J* = 7.4 Hz, 2H, CH_2_), 6.81 (dd, *J* = 8.8, 2.6 Hz, 1H, ArH), 6.86 (d, *J* = 2.5 Hz, 1H, ArH), 6.91 (br s, 2H, NH_2_), 7.15 (d, *J* = 8.7 Hz, 1H, ArH), 10.38 (br s, 1H, NH); ^13^C-NMR (126 MHz, DMSO-*d_6_*) δ (ppm) = 37.9 (2C), 43.3 (CH_3_), 47.3, 53.1 (CH_2_), 110.5, 111.6, 130.5 (CH), 80.7, 103.0, 116.9, 118.5, 121.9, 132.0, 149.9, 154.1, 156.82 (C) (further quaternary C were not detectable after 2048 scans); C_18_H_19_ClN_6_S (386.90); MS (APCI pos.): *m*/*z* (%) = 401.3 (100) [M + 14.4]^+^, 387.3 (44) [M + H]^+^.

*tert-Butyl 4-[4-(3-amino-2-cyano-3-thioxoprop-1-en-1-yl)-3-chlorophenyl)piperazine-1-carboxylate***15a***(KuSaSch092):* According to Procedure C from *tert*-butyl-4-(3-chloro-4-formylphenyl)piperazine-1-carboxylate (**12e**, 565 mg, 1.74 mmol), 2-cyanothioacetamide (**6**, 174 mg, 1.74 mmol) and triethyl-amine (50 µL) in ethanol (2 mL) for 10 min at 55 °C. After evaporation under reduced pressure a red-orange solid (700 mg, 99%) was obtained.

M.p.: Dec. starting at 168 °C; IR (KBr): ῦ_max_ 3390 cm^−1^, 3291 cm^−1^, (NH), 2978 cm^−1^ (CH aliphatic), 2196 cm^−1^ (C≡N), 1686 cm^−1^ (C=O); ^1^H-NMR (500 MHz, DMSO-*d*_6_) δ (ppm) = 1.42 (s, 9H, CH_3_), 3.42–3.49 (m, 8H, CH_2_), 7.03–7.09 (dd, *J* = 9.2, 2.6 Hz, 1H, ArH), 7.12–7.16 (d, *J* = 2.7 Hz, 1H, ArH), 8.13–8.18 (d, *J* = 9.1 Hz, 1H, ArH), 8.39–8.42 (s, 1H, CH), 9.38–9.42 (s, 1H, NH), 9.96–10.00 (s, 1H, NH); ^13^C-NMR (126 MHz, DMSO-*d_6_*) δ (ppm) = 27.9 (3C) (CH_3_); 45.8 (2C) (CH_2_) (two further secondary carbon signals were not detectable after 512 scans); 112.4, 113.7, 130.2, 144.7 (CH), 79.1, 107.9, 116.8, 117.3, 138.2, 153.2, 153.7, 192.2 (C); C_19_H_25_ClN_4_O_2_S (406.93); MS (APCI pos.): *m*/*z* (%) = 407 (100) [M + H]^+^, 371 (10) [M − 36]^+^, 351 (18) [M − 56]^+^, 335 (15) [M − 72]^+^, 317 (74) [M − 90]^+^, 273 (100) [M − 134]^+^.

*2-Cyano-3-phenylprop-2-enthioamide***15b***(KuSaSch097):* According to Procedure C from benzaldehyde (503 µL, 4.43 mmol), 2-cyanothioacetamide (**6**, 446 mg, 4.46 mmol) and triethylamine (50 µL) in ethanol (5 mL) for 2 h 30 min at 55 °C. After purification by column chromatography (petroleum ether/ethyl acetate 3:1) an orange solid (275 mg, 33%) was obtained. Due to the material′s sensitivity to oxidation, it was used without further purification for the subsequent synthesis of the corresponding thieno[2,3-*b*]pyridine.

M.p.: 130–133 °C (Lit.: 145 °C [30], 149–150 °C [31]); ^1^H-NMR (600 MHz, DMSO-*d*_6_) δ (ppm) = 7.50–7.65 (m, 3H, ArH), 7.90–7.97 (m, 2H, ArH), 8.08 (s, 1H, CH), 9.66 (br s, 1H, NH), 10.14 (br s, 1H, NH); ^13^C-NMR (151 MHz, DMSO-*d_6_*) δ (ppm) = 129.2 (2C), 130.0 (2C), 132.1, 146.6 (CH), 112.4, 116.2, 131.8, 192.2 (C); C_10_H_8_N_2_S (188.25); MS (APCI pos.): *m*/*z* (%) = 189 (100) [M + H]^+^, 172 (56) [M − 17]^+^, 155 (24) [M − 34]^+^.

*2-Cyano-3-(3-methylphenyl)prop-2-enthioamide***15c***(KuSaSch102):* According to Procedure C from 3-methylbenzaldehyde (249 µL, 2.11 mmol), 2-cyanothioacetamide (**6**, 212 mg, 2.11 mmol) and triethylamine (50 µL) in ethanol (2 mL) for 3 h. After 12 h storage at 4 °C, water (10 mL) was added and the mixture was extracted with ethyl acetate (3 × 50 mL). The combined organic layers were washed with saturated sodium chloride solution (30 mL), dried over sodium sulfate and evaporated under reduced pressure. A brown mass (346 mg, 81%) was obtained, which was used without further purification for subsequent syntheses.

*tert-Butyl 4-[3-chloro-4-(3-cyano-2-thioxo-2,5,6,7-tetrahydro-1H-cyclopenta[b]pyridin-4-yl)phenyl]-piperazine-1-carboxylate***16a***(KuSaSch093):* According to Procedure D from tert-butyl 4-[4-(3-amino-2-cyano-3-thioxoprop-1-en-1-yl)-3-chlorophenyl)piperazine-1-carboxylate (**15a**, 406 mg, 1.00 mmol), cyclopentanone (89 µL, 1.0 mmol) and piperidine (50 µL) in 1,4-dioxane (1 mL) for 1 h 45 min at 80 °C. After purification by column chromatography (petroleum ether/ethyl acetate 1:1→ethyl acetate 1) a yellow solid (259 mg, 55%) was obtained which still contained small amounts of the corresponding disulfide. Due to the material′s sensitivity to oxidation, it was used without further purification for the subsequent synthesis of the corresponding thieno[2,3-b]pyridine.

M.p.: 202–212 °C; IR (KBr): ῦ_max_ 3432 cm^−1^ (br, NH), 2974 cm^−1^ (CH aliphatic), 2223 cm^−1^ (C≡N), 1692 cm^−1^ (C=O); C_24_H_27_ClN_4_O_2_S (471.02); MS (APCI pos.): *m*/*z* (%) = 471 (5) [M + H]^+^, 447 (100) [M − 24]^+^, 427 (66) [M − 44]^+^, 415 (26) [M − 56]^+^, 371 (100) [M − 100]^+^, 339 (34) [M − 132]^+^.

*4-Phenyl-2-thioxo-2,5,6,7-tetrahydro-1H-cyclopenta[b]pyridine-3-carbonitrile***16b***(KuSaSch098):* According to Procedure D from 2-cyano-3-phenylprop-2-enthioamide (**15b**, 267 mg, 1.42 mmol), cyclopentanone (126 µL, 1.42 mmol) and piperidine (50 µL) in 1,4-dioxane (1 mL) at 80 °C for 3 h. After purification by column chromatography (petroleum ether/ethyl acetate 3:1→ethyl acetate 1→ethanol 1) a brown-orange solid (155 mg, 43%) was obtained which still contained small amounts of the corresponding disulfide. Due to the material′s sensitivity to oxidation, it was used without further purification for the subsequent synthesis of the corresponding thieno[2,3-*b*]pyridine.

M.p.: Dec. starting at 200 °C (Lit.: 240–242 [32], 239–240 °C [33], 239–240 °C [34], 239–240 °C [35]); IR (KBr): ῦ_max_ 3432 cm^−1^ (br NH), 2944 cm^−1^, 2880 cm^−1^, 2809 cm^−1^ (CH aliphatic), 2219 cm^−1^ (C≡N); C_15_H_12_N_2_S (252.34); MS (APCI pos.): *m*/*z* (%) = 253 (100) [M + H]^+^.

*4-Phenyl-2-thioxo-1,2,5,6,7,8-hexahydroquinoline-3-carbonitrile***16c***(KuSaSch103):* According to Procedure D from 2-cyano-3-phenylprop-2-enthioamide (**15b**, 125 mg, 0.67 mmol), cyclohexanone (69 µL, 0.67 mmol) and piperidine (50 µL) in 1,4-dioxane (1 mL) at 80 °C for 1 h 45 min. Afterwards, the mixture was evaporated under reduced pressure and the residue was crystallized from ethanol. An orange solid (38 mg, 21%) was obtained, which still contained small amounts of the corresponding disulfide. Due to the material′s sensitivity to oxidation, it was used without further purification for the subsequent synthesis of the corresponding thieno[2,3-*b*]pyridine.

M.p.: Dec. starting at 207 °C (Lit.: 240–242 °C [36], 267–268 °C [34], 238 °C [37]); ^1^H-NMR (500 MHz, DMSO-*d*_6_) δ (ppm) = 1.53–1.64 (m, 2H, CH_2_), 1.64–1.74 (m, 2H, CH_2_), 2.08 (t, *J* = 6.3 Hz, 2H, CH_2_), 2.78 (t, *J* = 6.4 Hz, 2H, CH_2_), 7.30–7.40 (m, 2H, ArH), 7.46–7.59 (m, 3H, ArH), 13.98 (br s, 1H, NH); ^13^C-NMR (126 MHz, DMSO-*d_6_*) δ (ppm) = 20.3, 21.2, 25.2, 27.1 (CH_2_), 127.3 (2C), 128.6 (2C), 129.1 (CH), 113.7, 116.2, 120.3, 135.0, 152.3, 157.7, 175.3 (C); C_16_H_14_N_2_S (266.36); MS (APCI pos.): *m*/*z* (%) = 267 (100) [M + H]^+^, 235 (25) [M − 31]^+^.

*2-Thioxo-4-(3-methylphenyl)-1,2,5,6,7,8-hexahydroquinoline-3-carbonitrile***16d***(KuSaSch104):* According to Procedure D from 2-cyano-3-(3-methylphenyl)prop-2-enthioamide (**15c**, 119 mg, 0.591 mmol), cyclohexanone (61 µL, 0.59 mmol) and piperidine (50 µL) in 1,4-dioxane (1 mL) at 80 °C for 1 h 45 min. The mixture was evaporated under reduced pressure and the residue was purified by column chromatography (petroleum ether/ethyl acetate 3:1). A yellow solid (50 mg, 30%) was obtained which still contained small amounts of the corresponding disulfide. Due to the material′s sensitivity to oxidation, it was used without further purification for the subsequent synthesis of the corresponding thieno[2,3-*b*]pyridine.

M.p.: Dec. starting at 172 °C; IR (KBr): ῦ_max_ 3434 cm^−1^ (br NH), 2946 cm^−1^, 2926 cm^−1^, 2857 cm^−1^ (CH aliphatic), 2220 cm^−1^ (C≡N); C_17_H_16_N_2_S (280.39); MS (APCI pos.): *m*/*z* (%) = 281 (100) [M + H]^+^, 249 (27) [M − 31]^+^.

*2-Thioxo-4-(3-methylphenyl)-2,5,6,7-tetrahydro-1H-cyclopenta[b]pyridine-3-carbonitrile***16e***(KuSaSch106):* According to Procedure D from 2-cyano-3-(3-methylphenyl)prop-2-enthioamide (**15c**, 213 mg, 1.05 mmol), cyclopentanone (94 µL, 1.1 mmol) and piperidine (50 µL) in 1,4-dioxane (1 mL) at 80 °C for 4 h. The mixture was evaporated under reduced pressure and the residue was purified by column chromatography (petroleum ether/ethyl acetate 3:1). A yellow solid (82 mg, 29%) was obtained which still contained small amounts of the corresponding disulfide. Due to the material′s sensitivity to oxidation, it was used without further purification for the subsequent synthesis of the corresponding thieno[2,3-*b*]pyridine.

M.p.: 206–211 °C; IR (KBr): ῦ_max_ 3431 cm^−1^ (NH), 2948 cm^−1^, 2891 cm^−1^, 2824 cm^−1^ (CH aliphatic), 2236 cm^−1^ (C≡N); ^1^H-NMR (500 MHz, DMSO-*d*_6_) δ (ppm) = 2.03 (p, *J* = 7.5 Hz, 2H, CH_2_), 2.39 (s, 3H, CH_3_), 2. 56 (t, *J* = 7.4 Hz, 2H, CH_2_), 3.00 (t, *J* = 7.7 Hz, 2H, CH_2_), 7.22–7.34 (m, 2H, ArH), 7.29–7.39 (m, 1H, ArH), 7.39–7.48 (m, 1H, ArH), 14.39 (br s, 1H, NH); ^13^C-NMR (126 MHz, DMSO-*d_6_*) δ (ppm) = 20.8 (CH_3_), 22.0, 29.1, 31.4 (CH_2_), 124.9, 128.2, 128.5, 130. (CH), 112.1, 116.9, 126.5, 134.6, 137.9, 154.2, 159.0, 176.8 (C); C_16_H_14_N_2_S (266.36); MS (APCI pos.): *m*/*z* (%) = 267 (100) [M + H]^+^, 235 (8) [M − 31]^+^.

*tert-Butyl 5-cyano-2-methyl-4-phenyl-6-thioxo-1,6-dihydropyridine-3-carboxylate***16f***(KuSaSch108):* According to Procedure D from 2-cyano-3-phenylprop-2-enthioamide (**15b**, 207 mg, 1.10 mmol), *tert*-butyl acetoacetate (181 µL, 1.10 mmol) and piperidine (50 µL) in 1,4-dioxane (1 mL) at 80 °C for 1 h 30 min. The mixture was evaporated under reduced pressure and purified by column chromatography (petroleum ether/ethyl acetate 3:1). A yellow solid (117 mg, 33%) was obtained which still contained small amounts of the corresponding disulfide. Due to the material′s sensitivity to oxidation, it was used without further purification for the subsequent synthesis of the corresponding thieno[2,3-*b*]pyridine.

M.p.: Dec. starting at 231 °C; IR (KBr): ῦ_max_ 2236 cm^−1^ (C≡N), 1720 cm^−1^ (C=O); ^1^H-NMR (500 MHz, DMSO-*d*_6_) δ (ppm) = 1.10 (s, 9H, CH_3_), 2.47 (s, 3H, CH_3_), 7.23–7.45 (m, 3H, ArH), 7.51–7.55 (m, 2H, ArH), 14.35 (s, 1H, NH); ^13^C-NMR (126 MHz, DMSO-*d_6_*) δ (ppm) = 17.7, 26.9 (3C) (CH_3_), 127.5 (2C), 128.4 (2C), 129.6 (CH), 83.2, 113.7, 116.1, 120.4, 135.1, 152.2, 154.6, 164.3, 177.7 (C); C_18_H_18_N_2_O_2_S (326.41); MS (APCI pos.): *m*/*z* (%) = 327 (100) [M + H]^+^, 271 (66) [M − 35]^+^.

*tert-Butyl 5-cyano-2-methyl-6-thioxo-4-(3-methylphenyl)-1,6-dihydropyridine-3-carboxylate***16g***(KuSaSch109):* According to Procedure D from 2-cyano-3-(3-methylphenyl)prop-2-enthioamide (**15c**, 289 mg, 1.43 mmol), *tert*-butyl acetoacetate (237 µL, 1.43 mmol) and piperidine (50 µL) in 1,4-dioxane (1 mL) at 80 °C for 1 h. The mixture was evaporated under reduced pressure and the residue was purified by column chromatography (petroleum ether/ethyl acetate 6:1→3:1). A yellow solid (150 mg, 31%) was obtained which still contained small amounts of the corresponding disulfide. Due to the material′s sensitivity to oxidation, it was used without further purification for the subsequent synthesis of the corresponding thieno[2,3-*b*]pyridine.

M.p.: Dec. starting at 213 °C; IR (KBr): ῦ_max_ 3431 cm^−1^ (br, NH), 2928 cm^−1^, 2857 cm^−1^ (CH aliphatic), 2236 cm^−1^ (C≡N), 1718 cm^−1^ (C=O); ^1^H-NMR (500 MHz, DMSO-*d_6_*) δ (ppm) = 1.11 (s, 9H, CH_3_), 2.35 (s, 3H, CH_3_), 2.46 (s, 3H, CH_3_), 7.10–7.18 (m, 2H, ArH), 7.32–7.38 (m, 1H, ArH), 7.38–7.45 (m, 1H, ArH), 14.33 (s, 1H, NH); ^13^C-NMR (126 MHz, DMSO-*d_6_*) δ (ppm) = 17.7, 20.7, 26.8 (3C) (CH_3_), 124.7, 127.8, 128.4, 130.1 (CH), 82.4, 113.9, 115.7, 119.8, 135.0, 137.7, 152.2, 154.7, 163.3, 178.1 (C); C_19_H_20_N_2_O_2_S (340.44); MS (APCI pos.): *m*/*z* (%) = 34 (100) [M + H]^+^; 285 (20) [M − 45]^+^, 177 (38) [M − 163]^+^, MS (APCI neg.): *m*/*z* (%) = 339 (100) [M − H]^−^, 211 (20) [M − 129]^−^.

*tert-Butyl 4-[3-chloro-4-(3-cyano-2-thioxo-1,2,5,6,7,8-hexahydroquinolin-4-yl)phenyl]piperazine-1-carboxylate***16h***(KuSaSch117):* According to Procedure D from *tert*-butyl 4-[4-(3-amino-2-cyano-3-thioxoprop-1-en-1-yl)-3-chloropheny])piperazine-1-carboxylate (**15a**, 546 mg, 1.24 mmol), cyclohexanone (130 µL, 1.24 mmol) and piperidine (50 µL) in 1,4 dioxane (1 mL) at 80 °C for 1 h. The mixture was evaporated under reduced pressure and the residue was purified by column chromatography (petroleum ether/ethyl acetate 2:1). An orange solid (134 mg, 22%) was obtained which still contained small amounts of the corresponding disulfide. Due to the material′s sensitivity to oxidation, it was used without further purification for the subsequent synthesis of the corresponding thieno[2,3-*b*]pyridine.

M.p.: Dec. starting at 199 °C; IR (KBr): ῦ_max_ 3428 cm^−1^ (br, NH), 2979 cm^−1^, 2926 cm^−1^ (CH aliphatic), 2225 cm^−1^ (C≡N), 1694 cm^−1^ (C=O); ^1^H-NMR (500 MHz, DMSO-*d*_6_) δ (ppm) = 1.43 (s, 9H, CH_3_), 1.53–1.63 (m, 2H, CH_2_), 1.63–1.74 (m, 2H, CH_2_), 1.97–2.12 (m, 2H, CH_2_), 2.71–2.86 (m, 2H, CH_2_), 3.24–3.29 (m, 4H, CH_2_), 3.43–3.49 (m, 4H, CH_2_), 7.01–7.08 (dd, *J* = 8.7, 2.5 Hz, 1H, ArH), 7.11–7.15 (d, *J* = 2.4 Hz, 1H, ArH), 7.17 (d, *J* = 8.6 Hz, 1H, ArH), 14.03 (br s, 1H, NH); ^13^C-NMR (126 MHz, DMSO-*d_6_*) δ (ppm) = 28.0 (3C) (CH_3_); 20.3, 21.0, 24.6, 27.0, 46.7 (2C) (CH_2_) (two further secondary carbon signals were not detectable after 480 scans); 113.8, 114.8, 129.6 (CH); 79.0, 115.9, 121.2, 122.8, 131.1, 151.9, 152.5, 153.8, 155.5, 175.2 (C); C_25_H_29_ClN_4_O_2_S (485.03); calcd. C 61.91, H 6.03, N 11.55, found C 62.13, H 5.94, N 11.25; MS (APCI pos.): *m*/*z* (%) = 485 (20) [M + H]^+^, 441 (51) [M − 44]^+^, 429 (84) [M − 56]^+^, 385 (58) [M − 100]^+^, 267 (100) [M − 218]^+^.

*tert-Butyl 4-[3-chloro-4-(3-cyano-6-methyl-2-thioxo-1,2-dihydropyridin-4-yl)phenyl]piperazine-1-carboxylate***16i***(KuSaSch121):* According to Procedure D from *tert*-butyl 4-[4-(3-amino-2-cyano-3-thioxoprop-1-en-1-yl)-3-chlorophenyl)piperazine-1-carboxylate (**15a**, 782 mg, 1.92 mmol), acetone (142 µL, 1.92 mmol) and piperidine (50 µL) in 1,4-dioxane (1 mL) at 50 °C for 30 min. The mixture was evaporated under reduced pressure and the residue was purified by column chromatography (petroleum ether/ethyl acetate 1:1→1:3). A light orange solid (292 mg, 33%) was obtained which still contained small amounts of the corresponding disulfide. Due to the material′s sensitivity to oxidation, it was used without further purification for the subsequent synthesis of the corresponding thieno[2,3-*b*]pyridine.

M.p.: 202–212 °C; IR (KBr): ῦ_max_ 3432 cm^−1^ (br, NH), 2968 cm^−1^, 2923 cm^−1^, 2855 cm^−1^ (CH aliphatic), 2226 cm^−1^ (C≡N), 1704 cm^−1^ (C=O); ^1^H-NMR (500 MHz, DMSO-*d*_6_) δ (ppm) = 1.43 (s, 9H, CH_3_), 2.41–2.43 (s, 3H, CH_3_), 3.26–3.31 (m, 4H, CH_2_), 3.46 (t, *J* = 5.4 Hz, 4H, CH_2_), 6.69–6.73 (m, 1H, ArH), 7.04 (dd, *J* = 8.8, 2.5 Hz, 1H, ArH), 7.12 (d, *J* = 2.4 Hz, 1H, ArH), 7.30 (d, *J* = 8.8 Hz, 1H, ArH), 14.12 (s, 1H, NH); ^13^C-NMR (126 MHz, DMSO-*d_6_*) δ (ppm) = 18.9, 28.0 (3C) (CH_3_); 46.7 (2C) (CH_2_) (two further secondary carbon signals were not detectable after 480 scans), 113.3, 114.9, 115.0, 130.6 (CH), 79.0, 113.6, 116.2, 123.8, 131.7, 152.2, 153.1, 153.8, 154.9, 177.9 (C); C_22_H_25_ClN_4_O_2_S (444.98); MS (APCI pos.): *m*/*z* (%) = 447 (100) [M + H]^+^, 307 (8) [M − 138]^+^, 181 (15) [M − 264]^+^.

*2-Chloro-N-heptylacetamide***8a***(KuSaSch128):* According to Procedure E from heptan-1-amine (595 µL, 4.00 mmol) in dried dichloromethane (8 mL) and K_2_CO_3_ (994 mg, 7.19 mmol). 2-Chloroacetyl chloride (**13**, 478 µL total, 6.00 mmol) was added in 100 µL steps every 15 min. The mixture was stirred for 1 h at room temperature and subsequently refluxed for 1 h 10 min. After addition of water (15 mL), the mixture was stirred for another 15 min at room temperature. Finally, extraction was carried out with dichloromethane (3 × 50 mL). The combined organic layers were dried over sodium sulfate. The solvent was evaporated under reduced pressure. A colorless solid (767 mg, 100%) was obtained.

M.p.: 33–34 °C; IR (KBr): ῦ_max_ 3311 cm^−1^ (NH), 2954 cm^−1^, 2925 cm^−1^, 2848 cm^−1^ (CH aliphatic); 1672 cm^−1^ (C=O), 1643 cm^−1^ (C=O); ^1^H-NMR (500 MHz, DMSO-*d*_6_) δ (ppm) = 0.82–0.89 (m, 3H, CH_3_), 1.16–1.34 (m, 8H, CH_2_), 1.41 (p, *J* = 7.3 Hz, 2H, CH_2_), 3.07 (m, 2H, CH_2_), 4.02 (s, 2H, CH_2_), 8.18 (m, 1H, amide); ^13^C-NMR (126 MHz, DMSO-*d_6_*) δ (ppm) = 13.9 (CH_3_), 24.4, 26.2, 28.3, 28.7, 31.1, 38.8, 42.6 (CH_2_); 165.6 (C); C_9_H_18_ClNO (191.70), calcd. C 56.39, H 9.46, N 7.31, found C 56.77, H 9.51, N 7.13.

*2-Chloro-N-isopropylacetamide***8b***(KuSaSch130):* According to Procedure E from propane-2-amine (328 µL, 4.00 mmol) in dried dichloromethane (8 mL) and K_2_CO_3_ (994 mg, 7.19 mmol). 2-Chloroacetyl chloride (**13**, 478 µL total, 6.00 mmol) was added and the mixture was stirred for 1 h at room temperature. Subsequently, the mixture was refluxed for 1 h 10 min. After the addition of water (15 mL), stirring was continued for another 15 min at room temperature. Finally, extraction was carried out with dichloromethane (3 × 50 mL) and the combined organic layers were dried over sodium sulfate. Evaporation under reduced pressure yielded a clear liquid which became solid when scratched with a glass rod. A colorless solid (297 mg, 85%) was obtained.

M.p.: 59–60 °C (Lit.: 60–62 °C [38], 60 °C [39]); IR (KBr): ῦ_max_ 3285 cm^−1^ (NH), 2976 cm^−1^ (CH aliphatic), 1652 cm^−1^ (C=O); ^1^H-NMR (500 MHz, DMSO-*d*_6_) δ (ppm) = 1.07 (d, *J* = 6.5 Hz, 6H, CH_3_), 3.83 (m, 1H, CH), 3.99 (s, 2H, CH_2_), 8.06 (d, *J* = 5.4 Hz, 1H, amide); ^13^C-NMR (126 MHz, DMSO-*d_6_*) δ (ppm) = 22.0 (2C) (CH_3_), 42.7 (CH_2_), 40.9 (CH), 164.7 (C); C_5_H_10_ClNO (135.59), calcd. C 44.29, H 7.43, N 10.33, found C 44.41, H 7.53, N 10.12.

*2-Chloro-N-cyclopropylacetamide***8c***(KuSaSch132):* According to Procedure E from cyclopropane-amine (278 µL, 4.00 mmol) in dried dichloromethane (8 mL) and K_2_CO_3_ (994 mg, 7.19 mmol). 2-Chloroacetyl chloride (**13**, 478 µL total, 6.00 mmol) was added and the mixture was stirred for 1 h at room temperature. Subsequently the mixture was refluxed for 1 h 10 min. After addition of water (15 mL), stirring was continued for another 15 min at room temperature. Extraction was carried out with dichloromethane (3 × 50 mL) and the combined organic layers were dried over sodium sulfate. Evaporation under reduced pressure yielded a clear liquid which became solid when scratched with a glass rod. A colorless solid (538 mg, 100%) was obtained.

M.p.: 86–87 °C (Lit.: 81–84 °C [40]); IR (KBr): ῦ_max_ 3271 cm^−1^ (NH), 1692 cm^−1^ (C=O), 1649 cm^−1^ (C=O);^1^H-NMR (400 MHz, DMSO-*d*_6_) δ (ppm) = 0.38–0.47 (m, 2H, CH_2_), 0.55–0.70 (m, 2H, CH_2_), 2.59–2.70 (m, 1H, CH), 3.97 (s, 2H, CH_2_), 8.28 (br s, 1H, amide); ^13^C-NMR (101 MHz, DMSO-*d_6_*) δ (ppm) = 5.5 (2C), 43.8 (CH_2_); 22.5 (CH), 166.9 (C); C_5_H_8_ClNO (133.58), calcd. C 44.96, H 6.04, N 10.49, found C 45.20, H 6.07, N 10.22.

*2-Chloro-N-(2-morpholinoethyl)acetamide***8d***(KuSaSch133):* According to Procedure E from 2-morpholinoethan-1-amine (522 µL, 4.00 mmol) in dried dichloromethane (8 mL) and K_2_CO_3_ (994 mg, 7.19 mmol). 2-Chloroacetyl chloride (**13**, 478 µL total, 6.00 mmol) was added and the mixture was stirred for 1 h at room temperature. Subsequently the mixture was refluxed for 1 h 10 min. After addition of water (15 mL), stirring was continued for another 15 min at room temperature. Finally, extraction was carried out with dichloromethane (3 × 50 mL). The combined organic layers were dried over sodium sulfate. Evaporation under reduced pressure yielded a clear liquid which became solid when scratched with a glass rod. A colorless solid (967 mg, 117%) was obtained. The raw product was used without purification. Both the NMR-spectrum and the yield of >100% indicated impurities.

*2-Chloro-N-(2-cyclopropylethyl)acetamide***8e***(KuSaSch136):* According to Procedure E from 2-cyclopropylethane-1-amine hydrochloride (243 mg, 2.00 mmol) in dried dichloromethane (8 mL) and K_2_CO_3_ (497 mg, 3.60 mmol). 2-Chloroacetyl chloride (**13**, 239 µL total, 3.00 mmol) was added and the mixture was stirred for 1 h at room temperature. Subsequently the mixture was refluxed for 1 h 10 min. After addition of water (15 mL), stirring was continued for another 15 min at room temperature. Finally, extraction was carried out with dichloromethane (3 × 50 mL) and the combined organic layers were dried over sodium sulfate. After evaporation under reduced pressure a colorless oil (323 mg, 100%) was obtained.

IR (KBr): ῦ_max_ 3422 cm^−1^, 3297 cm^−1^ (NH), 2932 cm^−1^ (CH aliphatic), 1660 cm^−1^ (C=O); ^1^H-NMR (500 MHz, DMSO-*d*_6_) δ (ppm) = 0.01–0.16 (m, 2H, CH_2_), 0.34–0.44 (m, 2H, CH_2_), 0.61–0.73 (m, 1H, CH), 1.32 (q, *J* = 7.1 Hz, 2H, CH_2_), 3.10–3.19 (m, 2H, CH_2_), 4.03 (s, 2H, CH_2_), 8.20 (s, 1H, amide); ^13^C-NMR (126 MHz, DMSO-*d_6_*) δ (ppm) = 4.0 (2C), 33.7, 38.9, 42.6 (CH_2_); 7.8 (CH), 165.5 (C).

*3,6-Diamino-4-(3-chlorophenyl)-N-(4-chlorophenyl)-5-cyanothieno[2,3-b]pyridine-2-carboxamide***9a***(KuSaSch018):* According to Procedure F from 6-amino-4-(3-chlorophenyl)-2-thioxo-1,2-dihydropyridine-3,5-dicarbonitrile (**7b**, 141 mg, 0.492 mmol), 2-chloro-*N*-(4-chlorophenyl)acetamide (103 mg, 0.505 mmol) and aqueous potassium hydroxide solution (10%, twice 258 μL, 0.500 mmol) for 4 h 30 min at 100 °C. After crystallization from ethanol a yellowish-ochre solid (105 mg, 47%) was obtained.

M.p.: 280–281 °C; IR (KBr): ῦ_max_ 3455 cm^−1^, 3402 cm^−1^, 3331 cm^−1^, 3288 cm^−1^ (NH), 2212 cm^−1^ (C≡N), 1630 cm^−1^ (C=O); ^1^H-NMR (DMSO-*d*_6_, 600 MHz): δ (ppm) = 5.80 (br s, 2H, NH_2_, signal deletion after D_2_O addition); 7.31–7.41 (m, 2H, ArH), 7.48–7.53 (m, 3H, ArH and NH_2_), 7.62–7.73 (m, 5H, ArH), 9.42–9.45 (br s, 1H, amide, signal deletion after D_2_O addition); ^13^C-NMR: (DMSO-*d*_6_, 151 MHz): δ = 122.3 (2C), 126.8, 128.0, 128.2 (2C), 130.0, 130.9 (CH); 90.3, 92.4, 113.5, 115.4, 126.8, 133.7, 135.4, 137.9, 147.6, 150.7, 158.5, 163.6, 163.7 (C); C_21_H_13_Cl_2_N_5_OS (454.33); HRMS (EI): *m*/*z* [M]^+•^; calcd. 453.02179, found 453.02118; MS (APCI pos.): *m*/*z* (%) = 454.1 (12) [M + H]^+^, 327.1 (100) [M − C_6_H_5_ClN]^+^, MS (APCI neg.): *m*/*z* (%) = 452.1 (100) [M − H]^−^, 299.0 (18) [M − C_7_H_5_ClNO]^−^; HPLC (isocr.): 97.8% at 254 nm and 98.6% at 280 nm, t_m_ = 1.0 min, t_ms_ = 5.8 min (ACN/H_2_O 60:40) (system 1); λ_max_: 283 nm, 319 nm; HPLC (grad.): 95.7% at 254 nm, t_m_ = 1.1 min, t_ms_ = 12.5 min (system 3).

*3,6-Diamino-N-(2-chlorophenyl)-5-cyano-4-(3-fluorophenyl)thieno[2,3-b]pyridine-2-carboxamide***9b***(KuSaSch022):* According to Procedure F from 6-amino-4-(3-fluorophenyl)-2-thioxo-1,2-dihydropyridine-3,5-dicarbonitrile (**7a**, 216 mg, 0.800 mmol), 2-chloro-*N*-(2-chlorophenyl)acetamide (164 mg, 0.804 mmol) and aqueous potassium hydroxide solution (10%, twice 413 μL, 0.800 mmol) for 2 h at 100 °C. After crystallization from ethanol an orange solid (135 mg, 39%) was obtained.

M.p.: 255–256 °C; IR (KBr): ῦ_max_ 3454 cm^−1^, 3385 cm^−1^, 3318 cm^−1^(NH), 2216 cm^−1^ (C≡N), 1631 cm^−1^ (C=O); ^1^H-NMR (600 MHz, DMSO-*d*_6_) δ (ppm) = 5.72 (br s, 2 H, NH_2_), 7.24 (td, *J* = 7.7, 1.7 Hz, 1H, ArH), 7.34 (td, *J* = 7.7, 1.5 Hz, 1H, ArH), 7.36–7.39 (m, 1H, ArH), 7.43–7.56 (m, 6H, ArH and NH_2_), 7.63–7.70 (m, 1H, ArH), 9.10–9.13 (br s, 1H, amide); ^13^C-NMR (151 MHz, DMSO-*d_6_*) δ (ppm)= 115.5 (d, *J* = 23.0 Hz, C-C-C-C-F), 116.9 (d, *J* = 20.9 Hz, C-C-C-C-F), 124.3 (d, *J* = 2.6 Hz, C-C-C-C-F), 131.3 (d, *J* = 8.5 Hz, C-C-C-C-F), 127.0, 127.3, 128.1, 129.3 (CH), 90.3, 92.7, 113.6, 115.4, 129.4, 135.1, 135.6 (d, *J* = 8.1 Hz, C-C-C-C-F), 147.2, 151.0, 158.5, 161.9 (d, *J* = 246.4 Hz, C-C-C-C-F), 163.5, 163.7 (C); ^19^F-NMR (337 MHz, DMSO-*d_6_*) δ (ppm) = −110.8 (s, 1F); C_21_H_13_ClFN_5_OS (437.88); calcd. C 57.60, H 2.99, N 15.99, found C 57.83, H 2.88, N 15.66; MS (APCI pos.): *m*/*z* (%) = 438.1 (100) [M + H]^+^, 311.1 (78) [M − C_6_H_5_ClN]^+^, MS (APCI neg.): *m*/*z* (%) = 436.1 (100) [M − H]^−^, 283.0 (15) [M − C_7_H_5_ClNO]^−^; HPLC (isocr.): 99.1% at 254 nm and 99.1% at 280 nm, t_m_ = 1.0 min, t_ms_ = 4.9 min (ACN/H_2_O 60:40) (system 1); λ_max_: 283 nm, 313 nm; HPLC (grad.): 95.4% at 254 nm, t_m_ = 1.1 min, t_ms_ = 12.2 min (system 3).

*3,6-Diamino-N-(2-chlorophenyl)-4-(3-chlorophenyl)-5-cyanothieno[2,3-b]pyridine-2-carboxamide***9c***(KuSaSch027):* According to Procedure F from 6-amino-4-(3-chlorophenyl)-2-thioxo-1,2-dihydropyridine-3,5-dicarbonitrile (**7b**, 226 mg, 0.788 mmol), 2-chloro-*N*-(2-chlorophenyl)acetamide (164 mg, 0.804 mmol) and aqueous potassium hydroxide solution (10%, twice 413 μL, 0.800 mmol) for 2 h at 100 °C. After crystallization from ethanol a yellow solid (129 mg, 35%) was obtained.

M.p.: 254–255 °C; IR (KBr): ῦ_max_ 3454 cm^−1^, 3386 cm^−1^, 3340 cm^−1^, 3218 cm^−1^ (NH), 2143 cm^−1^ (C≡N), 1631 cm^−1^ (C=O); ^1^H-NMR (600 MHz, DMSO-*d*_6_) δ (ppm) = 5.70 (br s, 2H, NH_2_), 7.24 (td, *J* = 7.7, 1.6 Hz, 1H, ArH), 7.34 (td, *J* = 7.8, 1.4 Hz, 1H, ArH), 7.48–7.56 (m, 5H, ArH and NH_2_), 7.64 (m, 1H, ArH), 7.66–7.73 (m, 2H, ArH), 9.12 (br s, 1H, amide); ^13^C-NMR (151 MHz, DMSO-*d*_6_) δ (ppm) = 126.8, 127.0, 127.3, 128.0, 128.1, 129.3, 130.0, 130.9 (CH), 90.3, 92.7, 113.6, 115.5, 129.4, 133.6, 135.0, 135.4, 147.2, 150.8, 158.4, 163.4, 163.7 (C); C_21_H_13_Cl_2_N_5_OS (454.33); calcd. C 55.52, H 2.88, N 15.42 found. C 55.51, H 2.85, N 15.07; MS (APCI pos.): *m*/*z* (%) = 454.1 (69) [M + H]^+^, 327.1 (100) [M − C_6_H_5_ClN]^+^, MS (APCI neg.): *m*/*z* (%) = 452.1 (100) [M − H]^−^, 299.0 (21) [M − C_7_H_5_ClNO]^−^; HPLC (isocr.): 96.6% at 254 nm and 99.6% at 280 nm, t_m_ = 1.0 min, t_ms_ = 6.2 min (ACN/H_2_O 60:40) (system 1); λ_max_: 283 nm, 316 nm; HPLC (grad.): 97.1% at 254 nm, t_m_ = 1.1 min, t_ms_ = 12.7 min (system 3).

*3,6-Diamino-4-(3-chlorophenyl)-5-cyano-N-(4-fluorophenyl)thieno[2,3-b]pyridine-2-carboxamide***9d***(KuSaSch028):* According to Procedure F from 6-amino-4-(3-chlorophenyl)-2-thioxo-1,2-dihydropyridine-3,5-dicarbonitrile (**7b**, 227 mg, 0.792 mmol), 2-chloro-*N*-(4-fluorophenyl)acetamide (172 mg, 0.917 mmol) and aqueous potassium hydroxide solution (10%, twice 448 μL, 0.800 mmol) for 2 h at 100 °C. After purification by column chromatography (petroleum ether/ethyl acetate 1.5:1) a yellow solid (75 mg, 22%) was obtained.

M.p.: 250–251 °C; IR (KBr): ῦ_max_ 3471 cm^−1^, 3415 cm^−1^, 3318 cm^−1^, 3204 cm^−1^ (NH), 2215 cm^−1^ (C≡N), 1638 cm^−1^ (C=O); ^1^H-NMR (600 MHz, DMSO-*d*_6_) δ (ppm) = 5.77 (br s, 2H, NH_2_), 7.09–7.16 (m, 2H, ArH), 7.49 (br s, 2H, NH_2_), 7.51 (m, 1H, ArH), 7.59–7.68 (m, 3H, ArH), 7.68–7.72 (m, 2H, ArH), 9.38 (br s, 1H, amide); ^13^C-NMR (151 MHz, DMSO-*d*_6_) δ (ppm) = 114.8 (d, *J* = 22.1 Hz, 2C, C-C-C-C-F), 122.9 (d, *J* = 7.8 Hz, 2C, C-C-C-C-F), 126.8, 128.0, 129.9, 130.9 (CH), 90.3, 92.6, 113.5, 115.5, 126.8, 128.0, 130.0, 130.9, 133.6, 135.1 (d, *J* = 2.6 Hz, C-C-C-C-F), 135.5, 147.3, 150.7, 158.4, 158.1 (d, *J* = 240.0 Hz, C-C-C-C-F), 163.5, 163.6 (C); ^19^F-NMR (377 MHz, DMSO-*d*_6_) δ (ppm) = −119.0 (s, 1F), C_21_H_13_ClFN_5_OS (437.88); calcd. C 57.60, H 2.99, N 15.99, found C 57.83, H 3.06, N 15.61; MS (APCI pos.): *m*/*z* (%) = 438.2 (68) [M + H]^+^, 327.1 (100) [M − C_6_H_5_FN]^+^, MS (APCI neg.): *m*/*z* (%) = 436.2 (100) [M − H]^−^, 299.0 (24) [M − C_7_H_5_FNO]^−^; HPLC (isocr.): 97.0% at 254 nm and 97.7% at 280 nm, t_m_ = 1.0 min, t_ms_ = 3.9 min (ACN/H_2_O 60:40) (system 1); λ_max_: 283 nm, 317 nm; HPLC (grad.): 98.7% at 254 nm, t_m_ = 1.1 min, t_ms_ = 11.6 min (system 3).

*3,6-Diamino-N-(4-chlorophenyl)-5-cyano-4-(3-methylphenyl)thieno[2,3-b]pyridine-2-carboxamide***9e***(KuSaSch031):* According to Procedure F from 6-amino-2-thioxo-4-(3-methylphenyl)-1,2-dihydropyridine-3,5-dicarbonitrile (**7c**, 218 mg, 0.819 mmol), 2-chloro-*N*-(4-chlorophenyl)acetamide (161 mg, 0.789 mmol) and aqueous potassium hydroxide solution (10%, twice 413 μL, 0.800 mmol) for 2 h at 100 °C. After crystallization from ethanol an orange solid (94 mg, 27%) was obtained.

M.p.: 255–256 °C; IR (KBr): ῦ_max_ 3473 cm^−1^, 3362 cm^−1^, 3307 cm^−1^(NH), 2216 cm^−1^ (C≡N), 1631 cm^−1^ (C=O); ^1^H-NMR (600 MHz, DMSO-*d*_6_) δ (ppm) = 2.41 (s, 3H, CH_3_), 5.79 (br s, 2H, NH_2_), 7.29–7.39 (m, 4H, ArH), 7.44–7.45 (m, 3H, ArH and NH_2_), 7.52 (m, 1H, ArH), 7.70–7.64 (m, 2H, ArH), 9.41 (br s, 1H, amide); ^13^C-NMR (151 MHz, DMSO-*d_6_*) δ (ppm) = 20.9 (CH_3_), 122.3 (2C), 124.9, 128.2 (2C), 128.2, 129.0, 130.6 (CH), 90.2, 92.1, 113.5, 115.6, 126.7, 133.4, 137.9, 138.5, 147.7, 152.5, 158.6, 163.6, 163.6 (C); C_22_H_16_ClN_5_OS (433.91); calcd. C 60.90, H 3.72, N 16.14, found C 60.94, H 3.64, N 15.98; MS (APCI pos.): *m*/*z* (%) = 434.2 (41) [M + H]^+^, 307.1 (100) [M − C_6_H_5_ClN]^+^, MS (APCI neg.): *m*/*z* (%) = 432.1 (100) [M − H]^−^, 279.0 (26) [M − C_7_H_5_ClNO]^−^; HPLC (isocr.): 97.4% at 254 nm and 98.5% at 280 nm, t_m_ = 1.0 min, t_ms_ = 6.0 min (ACN/H_2_O 50:50) (system 1); λ_max_: 263 nm, 313 nm; HPLC (grad.): 96.9% at 254 nm, t_m_ = 1.1 min, t_ms_ = 12.4 min (system 3).

*3,6-Diamino-N-(2-chlorophenyl)-5-cyano-4-(3-methylphenyl)thieno[2,3-b]pyridine-2-carboxamide***9f***(KuSaSch032):* According to Procedure F from 6-amino-2-thioxo-4-(3-methylphenyl)-1,2-dihydropyridine-3,5-dicarbonitrile (**7c**, 231 mg, 0.867 mmol), 2-chloro-*N*-(2-chlorophenyl)acetamide (163 mg, 0.799 mmol) and aqueous potassium hydroxide solution (10%, twice 413 μL, 0.800 mmol) for 1 h at 100 °C. After crystallization from ethanol an orange solid (147 mg, 42%) was obtained.

M.p.: 218–219 °C; IR (KBr): ῦ_max_ 3467 cm^−1^, 3386 cm^−1^, 3313 cm^−1^ (NH), 2214 cm^−1^ (C≡N), 1633 cm^−1^ (C=O); ^1^H-NMR (600 MHz, DMSO-*d*_6_) δ (ppm) = 2.41 (s, 3H, CH_3_), 5.69 (br s, 2H, NH_2_), 7.21–7.26 (m, 1H, ArH), 7.25–7.39 (m, 3H, ArH), 7.41–7.49 (m, 3H, ArH and NH_2_), 7.51 (m, 3H, ArH), 9.08 (br s, 1H, amide); ^13^C-NMR (151 MHz, DMSO-*d*_6_) δ (ppm) = 20.9 (CH_3_), 124.9, 126.9, 127.3, 127.9, 128.2, 129.0, 129.3, 130.6 (CH), 90.2, 92.4, 113.6, 115.6, 129.2, 133.4, 135.1, 138.5, 147.3, 152.6, 158.5, 163.4, 163.6 (C); C_22_H_16_ClN_5_OS (433.91); calcd. C 60.90, H 3.72, N 16.14, found C 60.64, H 3.66, N 15.88; MS (APCI pos.): *m*/*z* (%) = 434.2 (54) [M + H]^+^, 307.1 (92) [M − C_6_H_5_ClN]^+^, 280.1 (18) [M − C_7_H_5_ClNO + H]^+^, MS (APCI neg.): *m*/*z* (%) = 432.1 (100) [M − H]^−^, 279.0 (86) [M − C_7_H_5_ClNO]^−^; HPLC (isocr.): 98.6% at 254 nm and 99.5% at 280 nm, t_m_ = 1.0 min, t_ms_ = 6.7 min (ACN/H_2_O 50:50) (system 1); λ_max_: 283 nm, 315 nm; HPLC (grad.): 95.3% at 254 nm, t_m_ = 1.1 min, t_ms_ = 12.6 min (system 3).

*3,6-Diamino-5-cyano-N-(4-fluorophenyl)-4-(3-methylphenyl)thieno[2,3-b]pyridine-2-carboxamide***9g***(KuSaSch033):* According to Procedure F from 6-amino-2-thioxo-4-(3-methylphenyl)-1,2-dihydropyridine-3,5-dicarbonitrile (**7c**, 214 mg, 0.804 mmol), 2-chloro-*N*-(4-fluorophenyl)acetamide (153 mg, 0.816 mmol) and aqueous potassium hydroxide solution (10%, twice 413 μL, 0.800 mmol) for 1 h 30 min at 100 °C. After crystallization from ethanol an orange-yellow solid (45 mg, 13%) was obtained.

M.p.: 255–256 °C; IR (KBr): ῦ_max_ 3473 cm^−1^, 3366 cm^−1^, 3305 cm^−1^(NH), 2212 cm^−1^ (C≡N), 1631 cm^−1^ (C=O); ^1^H-NMR (400 MHz, DMSO-*d*_6_) δ (ppm) = 2.41 (s, 3H, CH_3_), 5.76 (br s, 2H, NH_2_), 7.07–7.18 (m, 2H, ArH), 7.28–7.36 (m, 2H, ArH), 7.41–7.48 (m, 3H, ArH and NH_2_), 7.52 (m, 1H, ArH), 7.57–7.67 (m, 2H, ArH), 9.34 (br s, 1H, amide); ^13^C-NMR (101 MHz, DMSO-*d_6_*) δ (ppm) = 20.9 (CH_3_), 114.8 (d, *J* = 22.1 Hz, 2C, C-C-C-C-F), 122.8 (d, *J* = 7.9 Hz, 2C, C-C-C-C-F), 124.9, 128.2, 129.0, 130.6 (CH), 90.2, 92.3, 113.6, 115.6, 124.9, 128.2, 129.0, 130.6, 133.5, 135.2 (d, *J* = 2.5 Hz, C-C-C-C-F), 138.5, 147.4, 152.5, 158.1 (d, *J* = 239.8 Hz, C-C-C-C-F), 158.5, 163.5, 163.6 (C); ^19^F-NMR (377 MHz, DMSO) δ (ppm) =−119.0 (s, 1F); C_22_H_16_FN_5_OS (417.46); calcd. C 63.30, H 3.86, N 16.78, found C 63.08, H 3.80, N 16.45; MS (APCI pos.): *m*/*z* (%) = 417.9 (100) [M + H]^+^, 306.9 (80) [M − C_6_H_5_FN]^+^, MS (APCI neg.): *m*/*z* (%) = 415.9 (100) [M − H]^−^, 278.8 (8) [M − C_7_H_5_FNO]^−^; HPLC (isocr.): 98.1% at 254 nm and 98.5% at 280 nm, t_m_ = 1.0 min, t_ms_ = 4.0 min (ACN/H_2_O 60:40) (system 1); λ_max_: 215 nm, 261 nm, 280 nm, 310 nm; HPLC (grad.): 96.0% at 254 nm, t_m_ = 1.1 min, t_ms_ = 11.6 min (system 3).

*3,6-Diamino-N-(4-chlorophenyl)-5-cyano-4-(3-fluorophenyl)thieno[2,3-b]pyridine-2-carboxamide***9h***(KuSaSch037):* According to Procedure F from 6-amino-4-(3-fluorophenyl)-2-thioxo-1,2-dihydropyridine-3,5-dicarbonitrile (**7a**, 211 mg, 0.781 mmol), 2-chloro-*N*-(4-chlorophenyl)acetamide (161 mg, 0.789 mmol) and aqueous potassium hydroxide solution (10%, twice 403 μL, 0.781 mmol) for 30 min at 100 °C. After purification by column chromatography (petroleum ether/ethyl acetate 1.5:1) an ochre-colored solid (118 mg, 35%) was obtained.

M.p.: 255–256 °C; IR (KBr): ῦ_max_ 3463 cm^−1^, 3414 cm^−1^, 3296 cm^−1^ (NH), 2215 cm^−1^ (C≡N), 1731 cm^−1^ (C=O); ^1^H-NMR (600 MHz, DMSO-*d*_6_) δ (ppm) = 5.82 (br s, 2H, NH_2_), 7.31–7.36 (m, 2H, ArH), 7.37–7.39 (m, 1H, ArH), 7.45–7.55 (m, 4H, ArH and NH_2_), 7.64–7.71 (m, 3 H, ArH), 9.44 (br s, 1H, amide); ^13^C-NMR (151 MHz, DMSO-*d*_6_) δ (ppm) = 115.4 (d, *J* = 22.9 Hz; C-C-C-C-F), 117.0 (d, *J* = 20.8 Hz, C-C-C-C-F), 122.3 (2C), 124.3 (d, *J* = 3.0 Hz, C-C-C-C-F), 128.2 (2C), 131.4 (d, *J* = 8.5 Hz, C-C-C-C-F) (CH), 90.3, 92.4, 113.4, 126.8, 135.6 (d, *J* = 8.2 Hz, C-C-C-C-F), 137.9, 147.6, 150.9, 158.5, 161.9 (d, *J* = 246.4 Hz, C-C-C-C-F), 163.6, 163.7 (C); ^19^F-NMR (471 MHz, DMSO-*d*_6_) δ (ppm) =−110.70–−110.91 (td, *J* = 9.2, 5.9 Hz); C_21_H_13_ClFN_5_SO (437.88); calcd. C 57.60, H 2.99, N 15.99, found C 57.89, H 3.11, N 16.03; MS (APCI pos.): *m*/*z* (%) = 437.9 (50) [M + H]^+^, 310.9 (100) [M − C_6_H_5_ClN]^+^, 285 (17) [M − 153]^+^, MS (APCI neg.): *m*/*z* (%) = 435.9 (100) [M − H]^−^, 282.8 (19) [M − C_7_H_5_ClNO]^−^; HPLC (grad.): 96.1% at 254 nm, t_m_ = 1.1 min, t_ms_ = 11.9 min (system 3); λ_max_: 225 nm, 283 nm, 319 nm.

*3,6-Diamino-5-cyano-4-(3-fluorophenyl)-N-(4-fluorophenyl)thieno[2,3-b]pyridine-2-carboxamide***9i***(KuSaSch038):* According to Procedure F from amino-4-(3-fluorophenyl)-2-thioxo-1,2-dihydropyridine-3,5-dicarbonitrile (**7a**, 252 mg, 0.932 mmol), 2-chloro-*N*-(4-fluorophenyl)acetamide (164 mg, 0.876 mmol) and aqueous potassium hydroxide solution (10%, twice 449 μL, 0.870 mmol) for 1 h 30 min at 100 °C. After purification by column chromatography (petroleum ether/ethyl acetate 1.5:1) an orange solid (137 mg, 37%) was obtained.

M.p.: 257–258 °C; IR (KBr): ῦ_max_ 3464 cm^−1^, 3414 cm^−1^, 3306 cm^−1^ (NH), 2216 cm^−1^ (C≡N), 1630 cm^−1^ (C=O); ^1^H-NMR (600 MHz, DMSO-*d*_6_) δ (ppm) = 5.78 (br s, 2H, NH_2_), 7.09–7.16 (m, 2H, ArH), 7.35–7.40 (m, 1H, ArH), 7.44–7.53 (m, 4H, ArH and NH_2_), 7.59–7.65 (m, 2H, ArH), 7.64–7.70 (m, 1H, ArH), 9.36–9.39 (s, 1H, amide); ^13^C-NMR (151 MHz, DMSO-*d_6_*) δ (ppm) = 114.8 (d, *J* = 22.1 Hz, 2C, C-C-C-C-F), 115.5 (d, *J* = 23.0 Hz, C-C-C-C-F), 117.0 (d, *J* = 21.0 Hz, C-C-C-C-F), 122.9 (d, *J* = 7.9 Hz, 2C, C-C-C-C-F), 124.4 (d, *J* = 2.7 Hz, C-C-C-C-F), 131.4 (d, *J* = 8.5 Hz, C-C-C-C-F) (CH), 90.2, 92.5, 113.5, 115.4, 135.1 (d, *J* = 2.6 Hz, C-C-C-C-F), 135.6 (d, *J* = 8.1 Hz, C-C-C-C-F), 147.3, 150.8, 158.1 (d, *J* = 240.0 Hz, C-C-C-C-F), 158.4, 161.9 (d, *J* = 246.2 Hz, C-C-C-C-F), 163.5, 163.6 (C); ^19^F-NMR (471 MHz, DMSO-*d*_6_) δ (ppm) = 118.95–119.03 (ddd, *J* = 13.8, 9.2, 5.1 Hz), 110.77–110.84 (td, *J* = 9.2, 6.5 Hz); C_21_H_13_F_2_N_5_OS (421.43); calcd. C 59.85, H 3.11, N 16.62, found. C 59.60, H 3.16, N 16.52; MS (APCI pos.): *m*/*z* (%) = 421.9 (76) [M + H]^+^, 310.9 (100) [M − C_6_H_5_FN]^+^, 327.0 (12) [M − C_6_H_4_F + H]^+^; MS (APCI neg.): *m*/*z* (%) = 419.9 (100) [M − H]^−^, 282.8 (25) [M − C_7_H_5_FNO]^−^; HPLC (grad.): 98.9% at 254 nm, t_m_ = 1.1 min, t_ms_ = 11.2 min (system 3); λ_max_: 224 nm, 259 nm, 282 nm, 316 nm.

*3,6-Diamino-5-cyano-N-methyl-4-(3-methylphenyl)thieno[2,3-b]pyridine-2-carboxamide***9j***(KuSaSch127):* According to Procedure F from 6-amino-2-thioxo-4-(3-methylphenyl)-1,2-dihydropyridine-3,5-dicarbonitrile (**7c**, 529 mg, 1.99 mmol), 2-chloro-*N*-methylacetamide (213 mg, 1.99 mmol) and potassium hydroxide solution (10%, twice 1.03 mL, 1.99 mmol) in DMF (1 mL) for 2 h at 100 °C. Subsequently, ice water (20 mL) was added to the mixture and the resulting precipitate was filtered off. After column chromatographic purification (toluene/ethyl acetate 4:1→3:1) and crystallization from ethanol a yellow solid (584 mg, 87%) was obtained.

M.p.: 272 °C; IR (KBr): ῦ_max_ 3480 cm^−1^, 3461 cm^−1^, 3432 cm^−1^(NH), 2209 cm^−1^ (C≡N), 1630 cm^−1^ (C=O); ^1^H-NMR (500 MHz, DMSO-*d*_6_) δ (ppm) = 2.40 (s, 3H, CH_3_), 2.66 (d, *J* = 4.5 Hz, 3H, CH_3_), 5.57 (br s, 2H, NH_2_, were deleted after D_2_O addition), 7.24–7.32 (m, 4H, ArH and NH_2_, signals of 2H were deleted after D_2_O addition), 7.39–7.45 (m, 1H, ArH), 7.49 (m, 2H, ArH and amide, signal of 1H was deleted after D_2_O addition); ^13^C-NMR (126 MHz, DMSO-*d_6_*) δ (ppm) = 20.9, 25.8 (CH_3_); 125.0, 128.2, 128.8, 130.4 (CH); 89.9, 93.3, 114.0, 115.7, 133.6, 138.3, 145.3, 152.1, 158.3, 163.0, 165.1 (C); C_17_H_15_N_5_OS (337.40), calcd. C 60.52, H 4.48, N 20.76, found. C 20.65, H 4.42, N 20.65; MS (APCI pos.): *m*/*z* (%) = 338.1 (100) [M + H]^+^, 392.1 (10) [M + 55]^+^, 307.1 (25) [M − 31]^+^; MS (APCI neg.): *m*/*z* (%) = 336.1 (100) [M − H]^−^; HPLC (grad.): 95.4% at 254 nm, t_m_ = 1.1 min, t_ms_ = 9.6 min (system 3); λ_max_: 280 nm, 310 nm.

*3,6-Diamino-5-cyano-N-heptyl-4-(3-methylphenyl)thieno[2,3-b]pyridine-2-carboxamide***9k***(KuSaSch129):* According to Procedure F from 6-amino-2-thioxo-4-(3-methylphenyl)-1,2-dihydropyridine-3,5-dicarbonitrile (**7c**, 415 mg, 3.46 mmol) and 2-chloro-*N*-heptylacetamide (**8a**, 664 mg, 3.46 mmol) in DMF (2 mL) and potassium hydroxide solution (10%, twice 1.79 mL, 3.46 mmol) for 5 h at 100 °C. Ice water (20 mL) was added and subsequently the mixture was extracted with ethyl acetate (5 × 75 mL). The combined organic layers were washed with saturated sodium chloride solution (50 mL), dried over sodium sulfate and evaporated under reduced pressure. The oily residue was mounted onto silica gel (3 g). Column chromatography (toluene/ethyl acetate 5:1) and crystallization from ethanol (70%) were used for purification. A yellow solid (408 mg, 28%) was obtained.

M.p.: (onset) 58 °C, 94 °C, 126 °C (determined by DSC, transformation between polymorphs was observed); IR (KBr): ῦ_max_ 3471 cm^−1^, 3399 cm^−1^, 3303 cm^−1^ (NH), 2925 cm^−1^, 2854 cm^−1^ (CH aliphatic), 2213 cm^−1^ (C≡N), 1625 cm^−1^ (C=O); ^1^H-NMR (500 MHz, DMSO-*d*_6_) δ (ppm) = 0.82–0.88 (m, 3H, CH_3_), 1.19–1.31 (m, 8H, CH_2_), 1.44 (m, 2H, CH_2_), 2.40 (s, 3H, CH_3_), 3.12 (q, *J* = 6.6 Hz, 2H, CH_2_), 5.57 (s, 2H, NH_2_, were deleted after D_2_O addition), 7.24–7.34 (m, 4H, ArH and NH_2_, signals of 2H were deleted after D_2_O addition), 7.39–7.45 (m, 1H, ArH), 7.46–7.55 (m, 2H, ArH and amide, signal of 1H was deleted after D_2_O addition); ^13^C-NMR (126 MHz, DMSO-*d_6_*) δ (ppm) = 13.9, 20.9 (CH_3_); 22.0, 26.3, 28.4, 29.3, 31.1, 38.9 (CH_2_); 125.0, 128.2, 128.8, 130.4 (CH); 89.9, 93.3, 114.0, 115.7, 133.6, 138.3, 145.4, 152.1, 158.2, 163.1, 164.5 (C); C_23_H_27_N_5_OS (421.56), calcd. C 65.53, H 6.46, N 16.61, found C 65.55, H 6.48, N 16.45; MS (APCI pos.): *m*/*z* (%) = 422.2 (100) [M + H]^+^; MS (APCI neg.): *m*/*z* (%) = 420.2 (100) [M − H]^−^; HPLC (isocr.): 95.6% at 254 nm and 97.8% at 280 nm, t_m_ = 1.0 min, t_ms_ = 5.8 min (ACN/H_2_O 65:35) (system 1); λ_max_: 252 nm, 292 nm, 343 nm; HPLC (grad.): 98.2% at 254 nm, t_m_ = 1.1 min, t_ms_ = 13.0 min (system 3).

*3,6-Diamino-5-cyano-N-isopropyl-4-(3-methylphenyl)thieno[2,3-b]pyridine-2-carboxamide***9l***(KuSaSch131):* According to Procedure F from 6-amino-2-thioxo-4-(3-methylphenyl)-1,2-dihydropyridine-3,5-dicarbonitrile (**7c**, 376 mg, 1.41 mmol) and 2-chloro-*N*-isopropylacetamide (**8b**, 192 mg, 1.41 mmol) in DMF (10 mL) and aqueous potassium hydroxide solution (10%, twice 728 µL, 1.41 mmol) for 6 h at 100 °C. Subsequently, ice water (20 mL) was added and the mixture was extracted with ethyl acetate (3 × 100 mL). The combined organic layers were washed with saturated sodium chloride solution (50 mL) and dried over sodium sulfate. The solvent was evaporated under reduced pressure and the residue was purified by column chromatography (toluene/propan-2-ol 10:1). After crystallization from toluene orange crystals (84 mg, 16%) were obtained.

M.p.: 255–257 °C; IR (KBr): ῦ_max_ 3470 cm^−1^, 3355 cm^−1^, 3306 cm^−1^ (NH), 2206 cm^−1^ (C≡N), 1620 cm^−1^ (C=O); ^1^H-NMR (500 MHz, DMSO-*d*_6_) δ (ppm) = 1.10 (d, *J* = 6.5 Hz, 6H, CH_3_), 2.40 (s, 3H, CH_3_), 4.02 (m, 1H, CH), 5.59 (br s, 2H, NH_2_, signals were deleted after D_2_O addition), 7.22–7.34 (m, 5H, ArH, NH_2_ and amide, signals of 3H were deleted after D_2_O addition), 7.39–7.45 (m, 1H, ArH), 7.45–7.53 (m, 1H, ArH); ^13^C-NMR (126 MHz, DMSO-*d_6_*) δ (ppm) = 20.9, 22.2, 22.2 (CH_3_); 40.4, 125.0, 128.2, 128.8, 130.4 (CH); 89.9, 93.4, 114.0, 115.7, 133.6, 138.3, 145.5, 152.1, 158.2, 163.1, 163.9; C_19_H_19_N_5_OS (365.46), calcd. C 62.45, H 5.24, N 19.16, found C 62.59, H 5.31, N 18.95; MS (APCI pos.): *m*/*z* (%) = 366.3 (38) [M + H]^+^, 307.2 (100) [M − 58]^+^, 281.2 (64) [M − 84]^+^; MS (APCI neg.): *m*/*z* (%) = 364.3 (100) [M − H]^−^, 279.1 (13) [M − 86]^−^; HPLC (isocr.): 95.1% at 254 nm and 98.5% at 280 nm, t_m_ = 1.1 min, t_ms_ = 4.5 min (ACN/H_2_O 50:50) (system 1); λ_max_: 251 nm, 292 nm, 343 nm; HPLC (grad.): 98.9% at 254 nm, t_m_ = 1.1 min, t_ms_ = 10.7 min (system 3).

*3,6-Diamino-5-cyano-N-cyclopropyl-4-(3-methylphenyl)thieno[2,3-b]pyridine-2-carboxamide***9m***(KuSaSch134):* According to Procedure F from 6-amino-2-thioxo-4-(3-methylphenyl)-1,2-dihydropyridine-3,5-dicarbonitrile (**7c**, 346 mg, 1.30 mmol) and 2-chloro-*N*-isopropylacetamide (**8c**, 265 mg, 1.30 mmol) in DMF (10 mL) and aqueous potassium hydroxide solution (10%, twice 671 µL, 1.30 mmol) for 3 h 30 min at 100 °C. Subsequently, ice water (20 mL) was added and the mixture was extracted with ethyl acetate (3 × 100 mL). The organic phases were combined, washed with saturated sodium chloride solution (50 mL) and dried over sodium sulfate. The solvent was evaporated and the residue was purified by column chromatography (dichloromethane/methanol 10:1). Subsequent crystallization from ethanol yielded a yellow solid (73 mg, 15%).

M.p.: (onset) 132 °C, 171 °C, 241 °C (determined by DSC, transformation between polymorphs was observed); IR (KBr): ῦ_max_ 3470 cm^−1^, 3309 cm^−1^, (NH), 2215 cm^−1^ (C≡N); ^1^H-NMR (500 MHz, DMSO-*d*_6_) δ (ppm) = 0.48–0.57 (m, 2H, CH_2_), 0.54–0.65 (m, 2H, CH_2_), 2.41 (s, 3H, CH_3_), 2.64–2.73 (m, 1H, CH), 5.62 (br s, 2H, NH_2_, signals was deleted after D_2_O addition), 7.25–7.31 (m, 2H, ArH), 7.33 (br s, 2H, NH_2_, signal was deleted after D_2_O addition), 7.39–7.45 (m, 1H, ArH), 7.46–7.53 (t, *J* = 7.6 Hz, 1H, ArH), 7.55–7.59 (d, *J* = 3.8 Hz, 1H, amide, signal was deleted after D_2_O addition); ^13^C-NMR (126 MHz, DMSO-*d_6_*) δ (ppm) = 20.9 (CH_3_, determined by HSQC), 5.7 (2C) (CH_2_); 22.7, 124.9, 128.2, 128.8, 130.4 (CH); 89.9, 93.0, 113.8, 115.7, 133.6, 138.6, 145.6, 152.2, 158.3, 163.2, 166.0 (C); C_19_H_17_N_5_OS (363.44), calcd. C 62.79, H 4.71, N 19.27, found C 62.60, H 4.63, N 19.00; MS (APCI pos.): *m*/*z* (%) = 364.2 (100) [M + H]^+^, 307.1 (58) [M − 56]^+^; MS (APCI neg.): *m*/*z* (%) = 362.2 (100) [M − H]^−^; HPLC (isocr.): 96.2% at 254 nm and 97.2% at 280 nm, t_m_ = 1.0 min, t_ms_ = 3.2 min (ACN/H_2_O 50:50) (system 1); λ_max_: 281 nm, 311 nm; HPLC (grad.): 97.9% at 254 nm, t_m_ = 1.1 min, t_ms_ = 10.0 min (system 3).

*3,6-Diamino-5-cyano-N-(2-morpholinoethyl)-4-(3-methylphenyl)thieno[2,3-b]pyridine-2-carboxamide***9n***(KuSaSch135):* According to Procedure A from 6-amino-2-thioxo-4-(3-methylphenyl)-1,2-dihydropyridine-3,5-dicarbonitrile (**7c**, 609 mg, 2.29 mmol) and 2-chloro-*N*-(2-morpholinoethyl)acetamide (**8d**, 473 mg, 2.29 mmol) in DMF (10 mL) and aqueous potassium hydroxide solution (10%, twice 1.18 mL, 2.29 mmol) for 2 h at 100 °C. Subsequently, ice water (20 mL) was added and the mixture was extracted with ethyl acetate (3 × 100 mL). The combined organic layers were washed with saturated sodium chloride solution (50 mL), dried over sodium sulfate and evaporated under reduced pressure. The residue was purified by column chromatography (ethyl acetate/ethanol 10:1→4:1). After crystallization from ethanol a yellow solid (379 mg, 38%) was obtained.

M.p.: Dec. starting at 246 °C; IR (KBr): ῦ_max_ 3464 cm^−1^, 3322 cm^−1^ (NH), 2206 cm^−1^ (C≡N), 1621 cm^−1^ (C=O); ^1^H-NMR (400 MHz, DMSO-*d*_6_) δ (ppm) = 2.34–2.42 (m, 9H, CH_2_), 3.26 (m, 2H, CH_2_), 3.55 (t, *J* = 4.7 Hz, 4H, CH_2_), 5.58 (br s, 2H, NH_2_, were deleted after D_2_O addition), 7.24–7.32 (m, 2H, ArH), 7.34 (br s, 2H, NH_2_, were deleted at D_2_O addition), 7.41–7.47 (m, 2H, ArH and amide, 1H was deleted after D_2_O addition), 7.47–7.53 (m, 1H, ArH); ^13^C-NMR (101 MHz, DMSO-*d_6_*) δ (ppm) = 20.9 (CH_3_); 36.0, 53.2 (2C), 57.4, 66.1 (2C) (CH_2_); 125.0, 128.2, 128.8, 130.5 (CH), 90.0, 93.2, 114.0, 115.7, 133.5, 138.3, 145.5, 152.2, 158.3, 163.1, 164.6 (C); C_22_H_24_N_6_O_2_S (436.53), calcd. C 60.53, H 5.54, N 19.25, found C 60.67, H 5.62, N 19.14; (APCI pos.): *m*/*z* (%) = 437.2 (100) [M + H]^+^; MS (APCI neg.): *m*/*z* (%) = 435.2 (100) [M − H]^−^; HPLC (isocr.; RP18 endcapped.): 98.6% at 254 nm and 99.2% at 280 nm, t_m_ = 1.8 min, t_ms_ = 5.1 min (ACN/buffer 30:70) (system 1); λ_max_: 282 nm, 308 nm.

*3,6-Diamino-5-cyano-N-(2-cyclopropylethyl)-4-(3-methylphenyl)thieno[2,3-b]pyridine-2-carboxamide***9o***(KuSaSch137):* According to Procedure F from 6-amino-2-thioxo-4-(3-methylphenyl)-1,2-dihydropyridine-3,5-dicarbonitrile (**7c**, 451 mg, 1.70 mmol) and 2-chloro-*N*-isopropylacetamide (**8e**, 274 mg, 1.70 mmol) in DMF (4 mL) and aqueous potassium hydroxide solution (10%, twice 877 µL, 1.70 mmol) for 2 h at 100 °C. Subsequently, ice water (20 mL) was added and the mixture was extracted with ethyl acetate (3 × 100 mL). The combined organic layers were washed with saturated sodium chloride solution (50 mL), dried over sodium sulfate and evaporated under reduced pressure. The residue was purified by column chromatography (toluene/ethyl acetate 9:1→4:1). A subsequent crystallization from ethanol yielded a yellow solid (215 mg, 32%).

M.p.: Dec. starting at 214–216 °C; IR (KBr): ῦ_max_ 3476 cm^−1^, 3417 cm^−1^, 3292 cm^−1^, 3198 cm^−1^ (NH), 2211 cm^−1^ (C≡N), 1624 cm^−1^ (C=O); ^1^H-NMR (500 MHz, DMSO-*d*_6_) δ (ppm) = 0.01–0.04 (m, 2H, CH_2_), 0.33–0.42 (m, 2H, CH_2_), 0.59–0.70 (m, 1H, CH), 1.35 (q, *J* = 7.1 Hz, 2H, CH_2_), 2.40 (s, 3H, CH_3_), 3.16–3.24 (m, 2H, CH_2_), 5.57 (br s, 2H, NH_2_, signal was deleted after D_2_O addition), 7.29 (m, 2H, ArH), 7.32 (br s, 2H, NH_2_, were deleted after D_2_O addition), 7.39–7.45 (m, 1H, ArH), 7.46–7.56 (m, 2H, ArH and amide, signal of 1H was deleted after D_2_O addition); ^13^C-NMR (126 MHz, DMSO-*d_6_*) δ (ppm) = 20.9 (CH_3_); 4.00 (2C), 34.2, 38.9 (CH_2_); 8.5 (CH, was determined by HSQC), 125.0, 128.2, 128.8, 130.4 (CH); 90.0, 93.4, 114.0, 115.7, 133.6, 138.3, 145.4, 152.1, 158.2, 163.1, 164.5, C_21_H_21_N_5_OS (391.49), calcd. C 64.43, H 5.41, N 17.89, found C 60.32, H 5.34, N 17.61; MS (APCI pos.): *m*/*z* (%) = 392.2 (100) [M + H]^+^, 307.1 (20) [M − 84]^+^; MS (APCI neg.): *m*/*z* (%) = 390.2 (100) [M − H]^−^; HPLC (isocr.): 95.7% at 254 nm and 95.9% at 280 nm, t_m_ = 1.0 min, t_ms_ = 6.6 min (ACN/H_2_O 50:50) (system 1); λ_max_: 280 nm, 310 nm; HPLC (grad.): 95.7% at 254 nm, t_m_ = 1.1 min, t_ms_ = 11.3 min (system 3).

*tert-Butyl 4-(3-chloro-4-{3,6-diamino-2-[(3-chlorophenyl)carbamoyl]-5-cyanothieno[2,3-b]pyridin-4-yl}phenyl)piperazine-1-carboxylate***9p***(KuSaSch041):* According to Procedure F from tert-Butyl 4-[4-(6-amino-3,5-dicyano-2-thioxo-1,2-dihydropyridin-4-yl)-3-chlorophenyl]piperazine-1-carboxylate (7d, 925 mg, 1.96 mmol) and 2-bromo-3′-chloroacetophenone (488 mg, 1.96 mmol) and aqueous potassium hydroxide solution (10%, twice 1.12 mL, 1.96 mmol) for 3 h 30 min at 100 °C. After purification by column chromatography (toluene/ethyl acetate 9:1) a yellow solid (120 mg, 10%) was obtained.

M.p.: Dec. starting at 163 °C; IR (KBr): ῦ_max_ 3480 cm^−1^, 3314 cm^−1^, 3163 cm^−1^ (NH), 2215 cm^−1^ (C≡N), 1673 cm^−1^ (C=O); ^1^H-NMR (500 MHz, DMSO-*d*_6_) δ (ppm) = 1.44 (s, 9H, 3 CH_3_), 3.29–3.35 (m, 4H, 2 CH_2_), 3.46–3.51 (m, 4H, 2 CH_2_), 5.88 (br s, 2H, NH_2_), 7.08 (ddd, *J* = 7.9, 2.1, 0.9 Hz, 1H, ArH), 7.13 (dd, *J* = 8.8, 2.4 Hz, 1H, ArH), 7.23 (d, *J* = 2.4 Hz, 1H, ArH), 7.32 (t, *J* = 8.1 Hz, 1H, ArH), 7.39 (d, *J* = 8.6 Hz, 1H, ArH), 7.49 (br s, 2H, NH_2_), 7.55–7.61 (m, 1H, ArH), 7.82 (t, *J* = 2.0 Hz, 1H; ArH), 9.44 (br s, 1H, amide); ^13^C-NMR (126 MHz, DMSO-*d_6_*) δ (ppm) = 46.5 (4 CH_2_), 28.0 (3 C), 113.7, 114.8, 119.0, 120.1, 122.7, 129.9, 130.3 (CH), 79.0, 91.1, 92.0, 114.2, 115.3, 120.4, 132.1, 132.6, 140.5, 147.8, 149.8, 152.3, 153.8, 158.7, 163.6, 163.7 (C); C_30_H_29_Cl_2_N_7_O_3_S (638.57); calcd. C 56.43, H 4.58, N 15.35, found C 56.44, H 4.58, N 15.21; MS (APCI pos.): *m*/*z* (%) = 638.0 (3) [M + H]^+^, 484.9 (12) [M − 153]^+^, 384.9 (100) [M − 253]; HPLC (grad.): 97.2% at 254 nm, t_m_ = 1.1 min, t_ms_ = 13.6 min (system 3); λ_max_: 217 nm, 267 nm, 316 nm.

*3,6-Diamino-4-[2-chloro-4-(piperazin-1-yl)phenyl]-N-(3-chlorophenyl)-5-cyanothieno[2,3-b]pyridine-2-carboxamide hydrochloride***9q***(KuSaSch043):* According to Procedure G from *tert*-butyl 4-(3-chloro-4-{[3,6-diamino-2-(3-chlorophenyl)carbamoyl]-5-cyanothieno[2,3-*b*]pyridin-4-yl}-phenyl)-piperazine-1-carboxylate (**9p**, 72 mg, 0.11 mmol) in dried dichloromethane (8 mL) and trifluoroacetic acid (3 mL) for 1 h. After subsequent precipitation, an ochre-colored solid (23 mg, 37%) was obtained.

M.p.: Dec. starting at 203 °C; IR (KBr): ῦ_max_ 3476 cm^−1^ (NH), 2362 cm^−1^ (NH_2_^+^), 2216 cm^−1^ (C≡N); ^1^H-NMR (500 MHz, DMSO-*d*_6_) δ (ppm) = 3.24 (s, 4H, CH_2_), 3.58 (t, *J* = 5.2 Hz, 4H, CH_2_), 5.87 (br s, 2H, NH_2_), 7.06–7.12 (m, 1H, ArH), 7.18 (dd, *J* = 8.8, 2.5 Hz, 1H, ArH), 7.28–7.35 (m, 2H, ArH), 7.41–7.47 (m, 1H, ArH), 7.49–7.52 (br s, 2H, NH_2_), 7.55–7.61 (m, 1H, ArH), 7.83 (t, *J* = 2.1 Hz, 1H, ArH), 9.09 (br s, 2H, NH_2_^+^), 9.475 (br s, 1H, amide); ^13^C-NMR (101 MHz, DMSO-*d*_6_): δ (ppm) = 42.4 (2C), 44.0 (2C), 114.1, 115.4, 119.0, 120.1, 122.7, 130.0, 130.4 (CH), 91.0, 92.2, 114.1, 115.3, 121.5, 132.2, 132.6, 140.5, 147.8, 149.7, 151.6, 158.7, 163.6, 163.7 (C); C_25_H_22_Cl_3_N_7_OS (573.07); HRMS (EI): *m*/*z* [M]^+•^ calcd. 537.09053, found 537.08999; MS (APCI pos.): *m*/*z* (%) = 537.7 (2) [M − HCl + H]^+^, 176.9 (100) [M − 396]^+^, MS (APCI neg.): *m*/*z* (%) = 535.7 (62) [M − HCl-H]^−^, 297.7 (100) [M − 275]^−^, 282.9 (27) [M − 290]^−^, 265.7 (37) [M − 307]^−^, 254.9 (40) [M − 318]^−^; HPLC (isocr.): 95.3% at 254 nm and 96.8% at 280 nm, t_m_ = 1.0 min, t_ms_ = 5.8 min (ACN/buffer pH 2.7, 35:65) (system 1); λ_max_: 260 nm, 320 nm.

*3,6-Diamino-4-(2-chloro-4-morpholinophenyl)-N-(3-chlorophenyl)-5-cyanothieno[2,3-b]pyridine-2carboxamide***9r***(KuSaSch050):* According to Procedure F from 6-amino-4-(2-chloro-4-morpholinophenyl)-2-thioxo-1,2-dihydropyridine-3,5-dicarbonitrile (**7e**, 156 mg, 0.420 mmol), 2-bromo-*N*-(3-chlorophenyl)acetamide (96 mg, 0.39 mmol) and aqueous potassium hydroxide solution (10%, twice 196 µL, 0.380 mmol) for 30 min at 100 °C. After purification by column chromatography (toluene/ethyl acetate 10:1→3.5:1) a yellow solid (73 mg, 36%) was obtained.

M.p.: Dec. starting at 253 °C; IR (KBr): ῦ_max_ 3480 cm^−1^, 3387 cm^−1^, 3313 cm^−1^ (NH), 2957 cm^−1^, 2923 cm^−1^, 2852 cm^−1^ (CH aliphatic), 2210 cm^−1^ (C≡N), 1629 cm^−1^ (C=O); ^1^H-NMR (600 MHz, DMSO-*d_6_*) δ (ppm) = 3.29–3.32 (m, 4H, CH_2_,), 3.74–3.78 (m, 4H, CH_2_), 5.88 (br s, 2H, NH_2_), 7.09 (ddd, *J* = 8.0, 2.1, 0.9 Hz, 1H, ArH), 7.13 (dd, *J* = 8.7, 2.5 Hz, 1H, ArH), 7.23 (d, *J* = 2.5 Hz, 1H, ArH), 7.30 (t, *J* = 8.1 Hz, 1H, ArH), 7.39 (d, *J* = 8.6 Hz, 1H, ArH), 7.50 (br s, 2H, NH_2_), 7.58 (ddd, *J* = 8.4, 2.0, 1.0 Hz, 1H, ArH), 7.82 (t, *J* = 2.1 Hz, 1H, ArH), 9.44 (br s, 1H, amide); ^13^C-NMR (151 MHz, DMSO-*d_6_*) δ (ppm) = 46.9 (2C), 65.8 (2C) (CH_2_), 113.3, 114.4, 119.0, 120.1, 122.7, 129.9, 130.2 (CH), 91.1, 92.0, 114.3, 115.3, 120.6, 132.1, 132.6, 140.5, 147.8, 149.8, 152.7, 158.7, 163.6, 163.7 (C); C_25_H_20_Cl_2_N_6_O_2_S (539.44); HRMS (EI): *m*/*z* [M]^+•^ calcd. 538.07455, found 538.07400; MS (APCI pos.): *m*/*z* (%) = 539.3 (4) [M + H]^+^, 412.3 (28) [M − 127]^+^, 386.3 (100) [M − 154]^+^; HPLC (isocr.): 96.2% at 254 nm and 98.0% at 280 nm, t_m_ = 1.1 min, t_ms_ = 5.0 min (ACN/H_2_O 60:40) (system 1); λ_max_: 211 nm, 265 nm, 319 nm; HPLC (grad.): 97.8% at 254 nm, t_m_ = 1.1 min, t_ms_ = 12.6 min (system 3).

*3,6-Diamino-4-[2-chloro-4-(pyrrolidin-1-yl)phenyl]-N-(3-chlorophenyl)-5-cyanothieno[2,3-b]pyridine-2-carboxamide***9s***(KuSaSch051):* According to Procedure F from 6-amino-4-[2-chloro-4-(pyrrolidin-1-yl)phenyl]-2-thioxo-1,2-dihydropyridine-3,5-dicarbonitrile (**7f**, 356 mg, 1.00 mmol), 2-bromo-*N*-(3-chlorophenyl)acetamide (242 mg, 0.974 mmol) and aqueous potassium hydroxide solution (10%, twice 501 µL, 0.970 mmol) for 30 min at 100 °C. After purification by column chromatography (toluene/ethyl acetate 10:1→3.5:1) an orange-yellow solid (186 mg, 37%) was obtained.

M.p.: Dec. starting at 254 °C; IR (KBr): ῦ_max_ 3480 cm^−1^, 3388 cm^−1^, 3312 cm^−1^ (NH), 2210 cm^−1^ (C≡N), 1704 cm^−1^ (C=O); ^1^H-NMR (500 MHz, DMSO-*d*_6_) δ (ppm) = 1.95–2.04 (m, 4H, CH_2_), 3.32–3.37 (m, 4H, CH_2_), 5.93 (br s, 2H, NH_2_), 6.70 (dd, *J* = 8.6, 2.4 Hz, 1H, ArH), 6.77 (d, *J* = 2.3 Hz, 1H, ArH), 7.10 (ddd, *J* = 8.0, 2.1, 1.1 Hz, 1H), 7.27–7.36 (m, 2H), 7.45 (br s, 2H, NH_2_), 7.55–7.61 (m, 1H, ArH), 7.83 (t, *J* = 2.1 Hz, 1H, ArH), 9.42 (br s, 1H, amide); ^13^C-NMR (126 MHz, DMSO-*d_6_*) δ (ppm) = 24.9 (2C), 47.2 (2C) (CH_2_), 110.4, 111.3, 117.7, 118.7, 123.2, 130.4, 130.7 (CH), 87.7, 94.6, 114.8, 114.9, 118.0, 131.7, 133.0, 140.1, 149.2, 159.3, 165.3, 166.0 (C); C_25_H_20_Cl_2_N_6_OS (523.44); HRMS (EI): *m*/*z* [M]^+•^ calcd. 522.07964, found 522.07909; MS (APCI pos.): *m*/*z* (%) = 523.3 (6) [M + H]^+^, 396.3 (26) [M − 127]^+^, 370.3 (100) [M − 153]^+^, 340.3 (45) [M − 183]^+^; HPLC (grad.): 95.2% at 254 nm, t_m_ = 1.1 min, t_ms_ = 12.4 min (system 3); λ_max_: 207 nm, 247 nm, 292 nm, 344 nm.

*3,6-Diamino-4-[2-chloro-4-(pyrrolidin-1-yl)phenyl]-N-(2-chlorophenyl)-5-cyanothieno[2,3-b]pyridine-2-carboxamide***9t***(KuSaSch055):* According to Procedure F from 6-amino-4-[2-chloro-4-(pyrrolidin-1-yl)phenyl]-2-thioxo-1,2-dihydropyridine-3,5-dicarbonitrile (**7f**, 363 mg, 1.02 mmol), 2-chloro-*N*-(2-chlorophenyl)acetamide (208 mg, 1.02 mmol) and aqueous potassium hydroxide solution (10%, twice 526 µL, 1.02 mmol) for 1 h at 100 °C. After purification by column chromatography (petroleum ether/ethyl acetate 5:1 3:1) a yellow solid (103 mg, 19%) was obtained.

M.p.: Dec. starting at 215 °C; IR (KBr): ῦ_max_ 3467 cm^−1^, 3448 cm^−1^, 3385 cm^−1^, 3313 cm^−1^ (NH), 3154 cm^−1^, 3128 cm^−1^, 3070 cm^−1^ (CH aromatic), 2968 cm^−1^, 2854 cm^−1^, 2840 cm^−1^ (CH aliphatic), 2218 cm^−1^ (C≡N), 1640 cm^−1^ (C=O); ^1^H-NMR (600 MHz, DMSO-*d*_6_) δ (ppm) = 1.95–2.02 (m, 4H, CH_2_), 3.30–3.34 (m, 4H, 2 CH_2_), 5.81 (br s, 2H, NH_2_), 6.69 (dd, *J* = 8.6, 2.3 Hz, 1H, ArH), 6.77 (d, *J* = 2.4 Hz, 1H, ArH), 7.24 (td, *J* = 7.7, 1.7 Hz, 1H, ArH), 7.29 (d, *J* = 8.5 Hz, 1H, ArH), 7.31–7.36 (td, *J* = 7.7, 1.5 Hz, 1H, ArH), 7.44 (br s, 2H, NH_2_), 7.48–7.55 (ddd, *J* = 15.0, 8.0, 1.6 Hz, 2H, ArH), 9.07 (br s, 1H, amide); ^13^C-NMR (151 MHz, DMSO-*d_6_*) δ (ppm) = 24.9 (2C), 47.2 (2C) (CH_2_), 110.8, 111.4, 126.9, 127.3, 128.0, 129.3, 130.2 (CH), 91.4, 92.2, 114.7, 115.4, 116.8, 129.2, 131.9, 135.0, 147.4, 149.1, 150.4, 158.7, 163.4, 163.5 (C); C_25_H_2_0Cl_2_N_6_OS (523.44); calcd. C 57.37, H 3.85, N 16.06, found C 57.50, H 3.82, N 16.42; MS (APCI pos.): *m*/*z* (%) = 523.4 (1) [M + H]^+^, 396.2 (16) [M − 127]^+^, 370.3 (100) [M − 153]^+^, 334.3 (20) [M − 189]^+^; HPLC (grad.): 98.0% at 254 nm, t_m_ = 1.1 min, t_ms_ = 13.7 min (system 3); λ_max_: 210 nm, 271 nm, 316 nm.

*3,6-Diamino-4-(2-chloro-4-morpholinophenyl)-N-(2-chlorophenyl)-5-cyanothieno[2,3-b]pyridine-2-carboxamide***9u***(KuSaSch056):* According to Procedure F from 6-amino-4-(2-chloro-4-morpholinophenyl)-2-thioxo-1,2-dihydropyridine-3,5-dicarbonitrile (**7e**, 374 mg, 1.01 mmol), 2-chloro-*N*-(2-chlorophenyl)acetamide (206 mg, 1.01 mmol) and aqueous potassium hydroxide solution (10%, twice 516 µL, 1.00 mmol) for 45 min at 100 °C. After purification by column chromatography (toluene/ethyl acetate 10:1) a yellow solid (174 mg, 32%) was obtained.

M.p.: Dec. starting at 197 °C; IR (KBr): ῦ_max_ 3460 cm^−1^, 3451 cm^−1^, 3387 cm^−1^, 3310 cm^−1^ (NH), 3073 cm^−1^ (CH aromatic), 2964 cm^−1^, 2919 cm^−1^, 2885 cm^−1^, 2849cm^−1^ (CH aliphatic), 2221 cm^−1^ (C≡N), 1641 cm^−1^ (C=O); ^1^H-NMR (600 MHz, DMSO-*d_6_*) δ (ppm) = 3.27–3.31 (m, 4H, CH_2_), 3.73–3.78 (m, 4H, CH_2_), 5.76 (br s, 2H, NH_2_), 7.12 (dd, *J* = 8.7, 2.5 Hz, 1H, ArH), 7.20–7.27 (m, 2H, ArH), 7.33 (td, *J* = 7.6, 1.5 Hz, ArH), 7.39 (d, *J* = 8.6 Hz, 1H, ArH), 7.47 (br s, 2H, NH_2_), 7.51 (ddd, *J* = 9.6, 8.0, 1.5 Hz, 2H, ArH), 9.09 (br s, 1H, amide); ^13^C-NMR (151 MHz, DMSO-d_6_) δ (ppm) = 46.9 (2C), 65.8 (2C) (CH_2_), 113.3, 114.4, 127.0, 127.3, 128.1, 229.3, 130.3 (CH), 91.0, 92.4, 114.4, 115.3, 120.6, 129.3, 132.1, 135.0, 147.2, 149.8, 152.7, 158.7, 163.4, 163.6 (C); C_25_H_20_Cl_2_N_6_O_2_S (539.44); HRMS (EI): *m*/*z* [M]^+•^ calcd. 538.07455, found 538.07400; MS (APCI pos.): *m*/*z* (%) = 539.4 (5) [M + H]^+^, 412.2 (16) [M − 127]^+^, 386.3 (100) [M − 153]^+^, 218.2 (28) [M − 321]^+^; HPLC (grad.): 97.8% at 254 nm, t_m_ = 1.1 min, t_ms_ = 12.2 min (system 3); λ_max_: 221 nm, 259 nm, 271 nm, 309 nm.

*3,6-Diamino-4-(2-chloro-4-morpholinophenyl)-N-(4-chlorophenyl)-5-cyanothieno[2,3-b]pyridine-2-carboxamide***9v***(KuSaSch057):* According to Procedure F from 6-amino-4-(2-chloro-4-morpholinophenyl)-2-thioxo-1,2-dihydropyridine-3,5-dicarbonitrile (**7e**, 282 mg, 0.758 mmol), 2-chloro-*N*-(4-chlorophenyl)acetamide (151 mg, 0.740 mmol) and aqueous potassium hydroxide solution (10%, twice 377 µL, 0.730 mmol) for 45 min at 100 °C. After column chromatographic purification (toluene/ethyl acetate 10:1) a yellow solid (77 mg, 20%) was obtained.

M.p.: Dec. starting at 277 °C; IR (KBr): ῦ_max_ 3482 cm^−1^, 3466 cm^−1^, 3405 cm^−1^, 3316 cm^−1^(NH), 2962 cm^−1^, 2923 cm^−1^, 2894 cm^−1^, 2853 cm^−1^ (CH aliphatic); 2216 cm^−1^ (C≡N), 1631 cm^−1^ (C=O); ^1^H-NMR (600 MHz, DMSO-*d*_6_) δ (ppm) = 3.28–3.33 (m, 4H, 2 CH_2_), 3.74–3.78 (m, 4H, 2 CH_2_), 5.85 (br s, 2H, NH_2_), 7.14 (dd, *J* = 8.7, 2.5 Hz, 1H, ArH), 7.23 (d, *J* = 2.5 Hz, 1H, ArH), 7.31–7.38 (m, 2H, ArH), 7.39 (d, *J* = 8.6 Hz, 1H, ArH), 7.48 (br s, 2H, NH_2_), 7.62–7.69 (m, 2H, ArH), 9.42 (br s, 1H, amide); ^13^C-NMR (151 MHz, DMSO-*d_6_*) δ (ppm) = 46.9 (2C), 65.8 (2C) (CH_2_), 113.3, 114.4, 122.4 (2C), 128.2 (2C), 130.2 (CH), 91.0, 92.2, 114.3, 115.3, 120.6, 126.7, 132.1, 137.9, 147.6, 149.8, 152.7, 158.7, 163.5, 163.6 (C); C_25_H_20_Cl_2_N_6_O_2_S (539.44); HRMS (EI): *m*/*z* [M]^+•^ calcd. 538.07455, found 538.07400; MS (APCI pos.): *m*/*z* (%) = 539.4 (22) [M + H]^+^, 412.2 (57) [M − 127]^+^, 386.3 (100) [M − 153]^+^, 362.4 (26) [M − 177]^+^, 325.4 (40) [M − 214]^+^; HPLC (grad.): 97.3% at 254 nm, t_m_ = 1.1 min, t_ms_ = 12.0 min (system 3); λ_max_: 213 nm, 265 nm, 319 nm.

*tert-Butyl {2-[(3-chloro-4-{3,6-diamino-2-[(3-chlorophenyl)carbamoyl]-5-cyanothieno[2,3-b]pyridin-4-yl}phenyl)(methyl)amino]ethyl}carbamate***9w***(KuSaSch058):* According to Procedure F from *tert*-butyl (2-{[4-(6-amino-3,5-dicyano-2-thioxo-1,2-dihydropyridin-4-yl)-3-chlorophenyl](methyl)-amino}ethyl)-carbamate (**7g**, 458 mg, 0.998 mmol), 2-chloro-*N*-(3-chlorophenyl)acetamide (204 mg, 1.00 mmol) and aqueous potassium hydroxide solution (10%, twice 516 µL, 1.00 mmol) for 30 min at 100 °C. After purification by column chromatography (toluene/ethyl acetate 5:1) and subsequent crystallization from ethanol (70%) a yellow solid (154 mg, 25%) was obtained.

M.p.: Dec. starting at 170 °C; IR (KBr): ῦ_max_ 3478 cm^−1^, 3400 cm^−1^, 3314 cm^−1^ (NH), 2926 cm^−1^, 2971 cm^−1^ (CH aliphatic), 2216 cm^−1^ (C≡N), 1695 cm^−1^ (C=O); ^1^H-NMR (500 MHz, DMSO-*d*_6_) δ (ppm) = 1.39 (s, 9H, 3 CH_3_), 3.01 (s, 3H, CH_3_), 3.15 (q, *J* = 6.7 Hz, 2H, CH_2_), 3.47 (t, *J* = 6.8 Hz, 2H, CH_2_), 5.93 (br s, 2H, NH_2_), 6.89 (dd, *J* = 8.7, 2.6 Hz, 1H, ArH), 6.94–7.01 (m, 2H, ArH), 7.07–7.13 (m, 1H, ArH), 7.28–7.37 (m, 2H, ArH), 7.47 (br s, 2H, NH_2_), 7.57–7.62 (m, 1H, ArH), 7.83 (t, *J* = 2.1 Hz, 1H, ArH), 9.44 (br s, 1H, amide); ^13^C-NMR (126 MHz, DMSO-*d_6_*) δ (ppm) = 28.2 (3C), 37.9 (CH_3_), 36.9, 50.6 (CH_2_), 110.6, 111.4, 119.0, 120.1, 122.7, 130.0, 130.2 (CH), 77.7, 91.3, 91.9, 114.5, 115.4, 117.3, 132.1, 132.6, 140.6, 148.0, 150.3, 151.0, 155.7, 158.8, 163.7, 163.7 (C); C_29_H_29_Cl_2_N_7_O_3_S (626.56); HRMS (EI): *m*/*z* [M]^+•^ calcd. 625.14296, found 625.14242; MS (APCI pos.): *m*/*z* (%) = 399.3 (20) [M − 227]^+^, 373.3 (100) [M − 253]^+^, 330.3 (20) [M − 269]; HPLC (grad.): 96.5% at 254 nm, t_m_ = 1.1 min, t_ms_ = 12.9 min (system 3); λ_max_: 207 nm, 269 nm, 318 nm.

*3,6-Diamino-4-(2-chloro-4-morpholinophenyl)-5-cyano-N-(4-fluorophenyl)thieno[2,3-b]pyridine-2-carboxamide***9x***(KuSaSch059):* According to Procedure F from 6-amino-4-(2-chloro-4-morpholinophenyl)-2-thioxo-1,2-dihydropyridine-3,5-dicarbonitrile (**7e**, 293 mg, 0.788 mmol), 2-chloro-*N*-(4-fluorophenyl)acetamide (148 mg, 0.789 mmol) and aqueous potassium hydroxide solution (10%, twice 408 µL, 0.790 mmol) for 1 h at 100 °C. After column chromatographic purification (toluene/ethyl acetate/TEA 5:1:0.05) a yellow solid (83 mg, 20%) was obtained.

M.p.: Dec. starting at 267 °C; IR (KBr): ῦ_max_ 3462 cm^−1^, 3408 cm^−1^, 3308 cm^−1^, 3169 cm^−1^ (NH), 2959 cm^−1^, 2923 cm^−1^, 2852 cm^−1^ (CH aliphatic), 2214 cm^−1^ (C≡N), 1634 cm^−1^ (C=O); ^1^H-NMR (500 MHz, DMSO-*d*_6_) δ (ppm) = 3.28–3.33 (m, 4H, 2 CH_2_), 3.73–3.79 (m, 4H, 2 CH_2_), 5.82 (br s, 2H, NH_2_), 7.08–7.20 (m, 3H, ArH), 7.22 (d, *J* = 2.4 Hz, 1H, ArH), 7.39 (d, *J* = 8.7 Hz, 1H, ArH), 7.46 (br s, 2H, NH_2_), 7.58–7.65 (m, 2H, ArH), 9.35 (br s, 1H, amide); ^13^C-NMR (126 MHz, DMSO-*d_6_*) δ (ppm) = 46.9 (2C), 65.8 (2C) (CH_2_); 113.2, 114.4, 114.8 (d, *J* = 22.1 Hz, 2C, C-C-C-C-F), 122.9 (d, *J* = 7.8 Hz, 2C, C-C-C-C-F), 130.2 (CH), 91.0, 92.3, 115.3, 120.7, 132.1, 135.1 (d, *J* = 2.2 Hz, C-C-C-C-F), 147.3, 149,7, 152.7, 158.1 (d, *J* = 240.1 Hz, C-C-C-C-F), 158.6, 160.9, 163.5, 163.6 (C); ^19^F-NMR (471 MHz, DMSO-*d*_6_) δ (ppm) = −119.04 (tt, ^3^*J* (H, F) = 8.7, ^4^*J* (H, F) 5.1 Hz); C_25_H_20_ClFN_6_O_2_S (522.98); HRMS (EI): *m*/*z* [M]^+•^ calcd. 522.10355, found 522.10408; MS (APCI pos.): *m*/*z* (%) = 522.4 (3) [M + H]^+^, 446.4 (28) [M − 76]^+^, 412.2 (34) [M − 111]^+^, 386.3 (100) [M − 137]^+^, 210.2 (67) [M − 312]^+^; HPLC (grad.): 96.6% at 254 nm, t_m_ = 1.1 min, t_ms_ = 11.3 min (system 3); λ_max_: 213 nm, 264 nm, 318 nm.

*tert-Butyl {2-[(3-chloro-4-{3,6-diamino-2-[(4-chlorophenyl)carbamoyl]-5-cyanothieno[2,3-b]pyridin-4-yl}phenyl)(methyl)amino]ethyl}carbamate***9y***(KuSaSch060):* According to Procedure F from tert-butyl (2-{[4-(6-amino-3,5-dicyano-2-thioxo-1,2-dihydropyridin-4-yl)-3-chlorophenyl](methyl)amino}ethyl)-carbamate (**7g**, 317 mg, 0.692 mmol), 2-chloro-N-(4-chlorophenyl)acetamide (141 mg, 0.691 mmol) and aqueous potassium hydroxide solution (10%, twice 356 µL, 0.690 mmol) for 45 min at 100 °C. After addition of water (20 mL), extraction was performed with dichloromethane (3 × 75 mL). The combined organic layers were washed with saturated sodium chloride solution (50 mL), dried with sodium sulfate and evaporated under reduced pressure. After purification by column chromatography (petroleum ether/propan-2-ol 5:1) and subsequent crystallization from ethanol a yellow solid (112 mg, 26%) was obtained.

M.p.: Dec. starting at 192 °C; IR (KBr): ῦ_max_ 3461 cm^−1^, 3437 cm^−1^, 3383 cm^−1^, 3314 cm^−1^ (NH), 2971 cm^−1^, 2927 cm^−1^ (CH aliphatic), 2213 cm^−1^ (C≡N), 1695 cm^−1^ (C=O); ^1^H-NMR (600 MHz, DMSO-*d*_6_) δ (ppm) = 1.38 (s, 9H, 3 CH_3_), 3.00 (s, 3H, CH_3_), 3.14 (q, *J* = 6.7 Hz, 2H, CH_2_), 3.46 (t, *J* = 6.7 Hz, 2H, CH_2_), 5.89 (br s, 2H, NH_2_), 6.87 (dd, *J* = 8.8, 2.6 Hz, 1H, ArH), 6.93 (d, *J* = 2.5 Hz, 1H, ArH), 6.98 (t, *J* = 5.9 Hz, 1H, ArH), 7.29 (t, *J* = 8.9 Hz, 1H, ArH), 7.31–7.36 (m, 2H, ArH), 7.45 (br s, 2H, NH_2_), 7.63–7.69 (m, 2H, ArH), 9.40 (br s, 1H, amide); ^13^C-NMR (151 MHz, DMSO-*d_6_*) δ (ppm) = 28.1 (3C), 37.9 (CH_3_), 36.9, 55.6 (CH_2_), 110.5, 111.3, 122.3 (2C), 128.2 (2C), 130.1 (CH), 77.7, 91.3, 92.0, 114.5, 115.4, 117.3, 126.7, 132.1, 137.9, 147.7, 150.2, 151.0, 155.7, 158.7, 163.6, 163.6 (C); C_29_H_29_Cl_2_N_7_O_3_S (626.56); calcd. C 55.59, H 4.67, N 15.65, found C 55.55, H 4.66, N 15.58; MS (APCI pos.): *m*/*z* (%) = 473.3 (10) [M − 153]^+^, 399.3 (69) [M − C_11_H_14_ClO_2_N]^+^, 373.3 (100) [M − 253]^+^, 356.4 (24) [M − 270]^+^, 337.3 (20) [M − 289]^+^, 330.3 (30) [M − 296]^+^; HPLC (isocr.): 95.9% at 254 nm and 97.5% at 280 nm, t_m_ = 1.2 min, t_ms_ = 3.7 min (ACN/H_2_O 70:30) (system 1); λ_max_: 219 nm, 270 nm, 312 nm; HPLC (grad.): 98.5% at 254 nm, t_m_ = 1.1 min, t_ms_ = 12.8 min (system 3).

*3,6-Diamino-4-[2-chloro-4-(pyrrolidin-1-yl)phenyl]-N-(4-chlorophenyl)-5-cyanothieno[2,3-b]pyridine-2-carboxamide***9z***(KuSaSch063):* According to Procedure F from 6-amino-4-[2-chloro-4-(pyrrolidin-1-yl)phenyl]-2-thioxo-1,2-dihydropyridine-3,5-dicarbonitrile (**7f**, 378 mg, 1.06 mmol), 2-chloro-N-(4-chlorophenyl)acetamide (217 mg, 1.06 mmol) and aqueous potassium hydroxide solution (10%, twice 547 µL, 1.06 mmol) for 2 h 15 min at 100 °C. After dual purification by column chromatography (toluene/ethyl acetate 2:1) a yellow solid (65 mg, 12%) was obtained.

M.p.: Dec. starting at 258 °C; IR (KBr): ῦ_max_ 3479 cm^−1^, 3466 cm^−1^, 3402 cm^−1^, 3321 cm^−1^ (NH), 2923 cm^−1^ (CH aliphatic), 2192 cm^−1^ (C≡N), 1630 cm^−1^ (C=O); ^1^H-NMR (500 MHz, DMSO-*d*_6_) δ (ppm) = 1.97–2.05 (m, 4H, CH_2_), another 2 CH_2_ were identified at 3.33 by HSQC under the HDO signal, 5.92 (br s, 2H, NH_2_), 6.71 (dd, *J* = 8.7, 2.4 Hz, 1H, ArH), 6.79 (d, *J* = 2.4 Hz, 1H, ArH), 7.31 (d, *J* = 8.5 Hz, 1H, ArH), 7.32–7.38 (m, 2H, ArH), 7.45 (br s, 2H, NH_2_), 7.65–7.71 (m, 2H, ArH), 9.41 (br s, 1H, amide); ^13^C-NMR (126 MHz, DMSO-*d_6_*) δ (ppm) = 24.7 (2C), 47.0 (2C) (CH_2_), 110.6, 111.1, 122.1 (2C), 127.9 (2C), 130.0 (CH), 91.4, 92.0, 114.6, 115.5, 116.8, 126.7, 132.0, 138.0, 147.8, 149.2, 150.4, 158.7, 163.6, 163.0 (C); C_25_H_20_Cl_2_N_6_OS (523.44); calcd. C 57.37, H 3.85, N 16.06, found C 57.68, H 4.01, N 16.03; MS (APCI pos.): *m*/*z* (%) = 523.4 (3) [M + H]^+^, 473.4 (30) [M − 50]^+^, 417.3 (11) [M − 106]^+^, 396.3 (25) [M − 127]^+^, 373.3 (100) [M − 150]^+^, 370.3 (60) [M − 153]^+^, 356.3 (17) [M − 167]^+^, 330.3 (17) [M − 193]^+^; HPLC (grad.): 96.6% at 254 nm, t_m_ = 1.1 min, t_ms_ = 13.4 min (system 3); λ_max_: 210 nm, 270 nm, 319 nm.

*3,6-Diamino-4-[2-chloro-4-(pyrrolidin-1-yl)phenyl]-5-cyano-N-(4-fluorophenyl)thieno[2,3-b]pyridine-2-carboxamide***9aa***(KuSaSch064):* According to Procedure F from 6-amino-4-[2-chloro-4-(pyrrolidin-1-yl)phenyl]-2-thioxo-1,2-dihydropyridine-3,5-dicarbonitrile (**7f**, 438 mg, 1.23 mmol), 2-chloro-*N*-(4-fluorophenyl)acetamide (231 mg, 1.23 mmol) and aqueous potassium hydroxide solution (10%, twice 635 µL, 1.23 mmol) for 2 h 15 min at 100 °C. After addition of water (20 mL), the product was extracted with dichloromethane (3 × 75 mL). The combined organic layers were washed with saturated sodium chloride solution (50 mL), dried with sodium sulfate and evaporated under reduced pressure. Purification of the residue by two successive independent column chromatographies (toluene/ethyl acetate 5:2 and petroleum ether/propan-2-ol 5:1) yielded a yellow solid (146 mg, 23%).

M.p.: Dec. starting at 249 °C; IR (KBr): ῦ_max_ 3478 cm^−1^, 3471 cm^−1^, 3406 cm^−1^, 3321 cm^−1^, 3303 cm^−1^ (NH), 2957 cm^−1^, 2922 cm^−1^ (CH aliphatic), 2213 cm^−1^ (C≡N),1633 cm^−1^ (C=O); ^1^H-NMR (500 MHz, DMSO-*d*_6_) δ (ppm) = 1.95–2.04 (m, 4H, 2 CH_2_), another 2 CH_2_ were identified by HSQC under the HDO signal, 5.87 (br s, 2H, NH_2_), 6.69 (dd, *J* = 8.6, 2.4 Hz, 1H, ArH), 6.77 (d, *J* = 2.4 Hz, 1H, ArH), 7.08–7.17 (m, 2H, ArH), 7.30 (d, *J* = 8.6 Hz, 1H, ArH), 7.42 (br s, 2H, NH_2_), 7.58–7.66 (m, 2H, ArH), 9.34 (s, 1H, amide); ^13^C-NMR (126 MHz, DMSO-*d_6_*) δ (ppm) = 24.9 (2C), 47.2 (2C) (CH_2_), 110.8, 111.4, 114.8 (d, *J* = 22.2 Hz, 2C, C-C-C-C-F), 122.9 (d, *J* = 7.9 Hz, 2C, C-C-C-C-F), 130.2 (CH), 91.4, 92.1, 114.6, 115.4, 116.8, 132.0, 135.2 (d, *J* = 2.6 Hz, C-C-C-C-F), 147.5, 149.1, 150.3, 158.0 (d, *J* = 239.9 Hz, C-C-C-C-F), 158.7, 163.5, 163.5 (C); ^19^F-NMR (471 MHz, DMSO-*d*_6_) δ (ppm) = −118.05 (tt, ^3^*J* (H, F) = 8.7, ^4^*J* (H, F) = 5.1 Hz).; C_25_H_20_ClFN_6_OS (506.98); HRMS (EI): *m*/*z* [M]^+•^ calcd. 506.10919, found 506.10864; MS (APCI pos.): *m*/*z* (%) = 507.4 (3) [M + H]^+^, 396.3 (13) [M − 111]^+^, 370.3 (100) [M − 136]^+^, 334.3 (11) [M − 173]^+^; MS (APCI neg.): *m*/*z* (%) = 505.4 (100) [M − H]^−^, 469.5 (42) [M − 38]^−^, 368.3 (25) [M − 139]^−^; HPLC (grad.): 97.5% at 254 nm, t_m_ = 1.1 min, t_ms_ = 12.6 min (system 3); λ_max_: 213 nm, 271 nm, 316 nm.

*3,6-Diamino-4-(2-chloro-4-{[2-(dimethylamino)ethyl](methyl)amino}phenyl)-5-cyano-N-(4-fluorophenyl)thieno[2,3-b]pyridine-2-carboxamide***9ab***(KuSaSch067):* According to Procedure F from 6-amino-4-(2-chloro-4-{[2-(dimethylamino)ethyl](methyl)amino}phenyl)-2-thioxo-1,2-dihydropyridine-3,5-dicarbonitrile (**7h**, 485 mg, 1.25 mmol), 2-chloro-N-(4-fluorophenyl)acetamide (251 mg, 1.34 mmol) and aqueous potassium hydroxide solution (10%, twice 645 µL, 1.25 mmol) for 30 min at 100 °C. After addition of ice water (25 mL), the mixture was extracted with dichloromethane (3 × 100 mL). The combined organic layers were washed with saturated sodium chloride solution (50 mL), dried over sodium sulfate and evaporated under reduced pressure. After purification by two successive independent column chromatographies (methanol/petroleum ether/TEA 10:3:0.05 and ethyl acetate/TEA 1:0.05) a yellow solid (16 mg, 2%) was obtained.

M.p.: Dec. starting at 190 °C; IR (KBr): ῦ_max_ 3471 cm^−1^, 3460 cm^−1^, 3411 cm^−1^, 3314 cm^−1^ (NH), 2211 cm^−1^ (C≡N), 1634 cm^−1^ (C=O); ^1^H-NMR (500 MHz, DMSO-*d*_6_) δ (ppm) = 2.22 (s, 6H, 2 CH_3_), 2.45 (t, *J* = 7.0 Hz, 2H, CH_2_), 3.01 (s, 3H, CH_3_), 3.48–3.54 (m, 2H, CH_2_), 5.85 (br s, 2H, NH_2_), 6.84 (dd, *J* = 8.7, 2.6 Hz, 1H, ArH), 6.89 (d, *J* = 2.5 Hz, 1H, ArH), 7.08–7.17 (m, 2H, ArH), 7.27–7.33 (d, *J* = 8.7 Hz, 1H, ArH), 7.42 (br s, 2H, NH_2_), 7.58–7.66 (m, 2H, ArH), 9.32–9.36 (s, 1H, amide); ^13^C-NMR (126 MHz, DMSO-*d_6_*) δ (ppm) = 38.1, 45.5 (2C) (CH_3_), 49.5, 55.6 (CH_2_), 110.6, 111.4, 114.8 (d, *J* = 22.2 Hz, 2C, C-C-C-C-F), 122.9 (d, *J* = 7.8 Hz, 2C, C-C-C-C-F), 130.2 (CH), 91.2, 92.2, 114.6, 115.4, 117.3, 132.1, 135.2 (d, *J* = 2.7 Hz, C-C-C-C-F), 147.4, 150.1, 150.8, 158.1 (d, *J* = 240.1 Hz, C-C-C-C-F), 158.7, 163.5 (C) (another quaternary carbon could not be detected even after 2048 scans); ^19^F-NMR (471 MHz, DMSO-*d*_6_) δ (ppm) = −119.07 (tt, ^3^*J* (H, F) = 8.7, ^4^*J* (H, F) 5.1 Hz); C_26_H_25_ClFN_7_OS (538.04); HRMS (EI): *m*/*z* [M]^+•^ calcd. 537.15139, found 537.15084; MS (APCI pos.): *m*/*z* (%) = 538.5 (8) [M + H]^+^, 377.4 (20) [M − 160]^+^, 338.6 (38) [M − 200]^+^, 282.4 (63) [M − 256]; HPLC (isocr.): 99.5% at 254 nm and 99.6% at 280 nm, t_m_ = 0.9 min, t_ms_ = 3.7 min (ACN/buffer 35:65) (system 1); λ_max_: 263 nm, 316 nm.

*tert-Butyl {2-[(3-chloro-4-{3,6-diamino-5-cyano-2-[(4-fluorophenyl)carbamoyl]thieno[2,3-b]pyridin-4-yl}phenyl)(methyl)amino]ethyl}carbamate***9ac***(KuSaSch073):* According to Procedure F from tert-butyl (2-{[4-(6-amino-3,5-dicyano-2-thioxo-1,2-dihydropyridin-4-yl)-3-chlorophenyl]-(methyl)-amino}ethyl)carbamate (**7g**, 517 mg, 1.13 mmol), 2-chloro-N-(4-fluorophenyl)acetamide (213 mg, 1.14 mmol) and aqueous potassium hydroxide solution (10%, twice 583 µL, 1.13 mmol) for 2 h 30 min at 100 °C. After purification by two independent column chromatographies (petroleum ether/propan-2-ol 5:1 and toluene/ethyl acetate 5:1) a yellow solid (279 mg, 40%) was obtained.

M.p.: Dec. starting at 181 °C; IR (KBr): ῦ_max_ 3480 cm^−1^, 3460 cm^−1^, 3414 cm^−1^, 3368 cm^−1^, 3313 cm^−1^ (NH), 2974 cm^−1^, 2932 cm^−1^ (CH aliphatic), 2215 cm^−1^ (C≡N), 1717 cm^−1^ (C=O); ^1^H-NMR (500 MHz, DMSO-*d*_6_) δ (ppm) = 1.39 (s, 9H, 3 CH_3_), 3.01 (s, 3H, CH_3_), 3.15 (q, *J* = 6.7 Hz, 2H, CH_2_), 3.47 (t, *J* = 6.7 Hz, 2H, CH_2_), 5.87 (br s, 2H, NH_2_), 6.88 (dd, *J* = 8.8, 2.6 Hz, 1H, ArH), 6.93–7.01 (m, 2H, ArH), 7.10–7.18 (m, 2H, ArH), 7.31 (d, *J* = 8.6 Hz, 1H, ArH), 7.44 (br s, 2H, NH_2_), 7.59–7.67 (m, 2H, ArH), 9.35 (br s, 1H, amide); ^13^C-NMR (126 MHz, DMSO-*d_6_*) δ (ppm) = 28.2 (3C), 37.9 (CH_3_), 36.9, 50.6 (CH_2_), 110.5, 111.4, 114.9 (d, *J* = 22.3 Hz, 2C, C-C-C-C-F), 122.9 (d, *J* = 7.7 Hz, 2C, C-C-C-C-F), 130.2 (CH), 77.7, 91.3, 92.2, 114.6, 115.4, 117.3, 132.1, 135.18 (d, *J* = 2.6 Hz, C-C-C-C-F), 147.5, 150.2, 151.0, 155.7, 158.1 (d, *J* = 240.0 Hz, C-C-C-C-F), 158.7, 163.5, 163.6 (C); ^19^F-NMR (471 MHz, DMSO-*d*_6_) δ (ppm) = −119.06 (tt, ^3^*J* (H, F) = 8.7, ^4^*J* (H, F) = 5.1 Hz); C_29_H_29_ClFN_7_O_3_S (610.11); calcd. C 57.09, H 4.79, N 16.07, found C 57.25, H 4.77, N 15.71; MS (APCI pos.): *m*/*z* (%) = 510.5 (3) [M − 100]^+^, 473.4 (12) [M − 137]^+^, 417.3 (16) [M − 193]^+^, 399.3 (27) [M − 211]^+^, 373.3 (100), [M − 237]^+^, 356.3 (22) [M − 254]^+^, 330.3 (22) [M − 280]^+^; HPLC (isocr.): 96.4% at 254 nm and 97.7% at 280 nm, t_m_ = 1.1 min, t_ms_ = 5.5 min (ACN/H_2_O 60:40) (system 1); λ_max_: 212 nm, 269 nm, 317 nm; HPLC (grad.): 97.5% at 254 nm, t_m_ = 1.1 min, t_ms_ = 12.2 min (system 3).

*tert-Butyl {2-[(3-chloro-4-{3,6-diamino-2-[(2-chlorophenyl)carbamoyl]-5-cyanothieno[2,3-b]pyridin-4-yl}phenyl)(methyl)amino]ethyl}carbamate***9ad***(KuSaSch074):* According to Procedure F from *tert*-butyl (2-{[4-(6-amino-3,5-dicyano-2-thioxo-1,2-dihydropyridin-4-yl)-3-chlorophenyl]-(methyl)-amino}ethyl)carbamate (**7g**, 465 mg, 1.01 mmol), 2-chloro-*N*-(2-chlorophenyl)acetamide (211 mg, 1.03 mmol) and aqueous potassium hydroxide solution (10%, twice 521 µL, 1.01 mmol) for 2 h at 100 °C. After column chromatographic purification (toluene/ethyl acetate 10:1) a yellow solid (188 mg, 30%) was obtained.

M.p.: Dec. starting at 203 °C; IR (KBr): ῦ_max_ 3474 cm^−1^, 3449 cm^−1^, 3389 cm^−1^, 3312 cm^−1^ (NH), 2974 cm^−1^, 2930 cm^−1^ (CH aliphatic), 2216 cm^−1^ (C≡N), 1700 cm^−1^ (C=O); ^1^H-NMR (500 MHz, DMSO-*d*_6_) δ (ppm) = 1.37 (s, 9H, 3 CH_3_), 2.99 (s, 3H, CH_3_), 3.13 (q, *J* = 7.3, 6.8 Hz, 2H, CH_2_), 3.45 (t, *J* = 6.8 Hz, 2H, CH_2_), 5.79 (br s, 2H, NH_2_), 6.86 (dd, *J* = 8.8, 2.5 Hz, 1H, ArH), 6.91–6.99 (m, 2H, ArH), 7.20–7.28 (m, 1H, ArH), 7.29 (d, *J* = 8.6 Hz, 1H, ArH), 7.34 (td, *J* = 7.7, 1.5 Hz, 1H, ArH), 7.44 (br s, 2H, NH_2_), 7.52 (ddd, *J* = 11.2, 8.0, 1.5 Hz, 2H, ArH), 9.06 (br s, 1H, amide); ^13^C-NMR (126 MHz, DMSO-*d_6_*) δ (ppm) = 28.2 (3C), 37.9 (CH_3_), 26.9, 50.6 (CH_2_), 110.5, 111.3, 126.9, 127.3, 128.0, 129.3, 130.1 (CH), 77.7, 91.3, 92.2, 114.6, 115.4, 117.3, 129.3, 132.1, 135.1, 147.4, 150.3, 151.0, 155.7, 158.7, 163.5, 163.5 (C); C_29_H_29_Cl_2_N_7_O_3_S (626.56); calcd. C 55.59, H 4.67, N 15.65, found C 55.64, H 4.87, N 15.28; MS (APCI pos.): *m*/*z* (%) = 526.4 (20) [M − 100]^+^, 473.5 (100) [M − 153]^+^, 443.3 (27) [M − 183]^+^, 399.3 (72) [M − 227]^+^, 373.3 (58) [M − 253]^+^, 335.2 (100) [M − 291]^+^, 255.2 (38) [M − 371]^+^; HPLC (grad.): 98.6% at 254 nm, t_m_ = 1.0 min, t_ms_ = 12.2 min (system 3); λ_max_: 211 nm, 269 nm, 316 nm.

*3,6-Diamino-4-{4-[(2-aminoethyl)(methyl)amino]-2-chlorophenyl}-N-(3-chlorophenyl)-5-cyanothieno-[2,3-b]pyridine-2-carboxamide hydrochloride***9ae***(KuSaSch075):* According to Procedure G from *tert*-butyl {2-[(3-chloro-4-{3,6-diamino-2-[(3-chlorophenyl)carbamoyl]-5-cyanothieno[2,3-*b*]pyridin-4-yl}phenyl)(methyl)amino]ethyl}carbamate (**9w**, 83 mg, 0.13 mmol) in dried dichloromethane (3 mL) and trifluoroacetic acid (3 mL) for 30 min. The mixture was evaporated under reduced pressure and the residue was dissolved in propan-2-ol (4 mL). Hydrogen chloride (5–6 M) in isopropan-2-ol (26 µL) was added to the mixture. After 3 days at room temperature a precipitate was formed which was filtered off. A yellow solid (22 mg, 30%) was obtained.

M.p.: Dec. starting at 209 °C; IR (KBr): ῦ_max_ 3429 cm^−1^, 3394 cm^−1^, 3378 cm^−1^ (NH), 2217 cm^−1^ (C≡N), 1618 cm^−1^ (C=O); ^1^H-NMR (600 MHz, DMSO-*d*_6_) δ (ppm) = 2.99–3.07 (m, 5H, CH_3_ und CH_2_), 3.66 (t, *J* = 7.0 Hz, 2H, CH_2_), 5.91 (br s, 2H, NH_2_), 6.96 (dd, *J* = 8.8, 2.6 Hz, 1H, ArH), 7.05 (d, *J* = 2.5 Hz, 1H, ArH), 7.07–7.12 (m, 1H, ArH), 7.31 (t, *J* = 8.1 Hz, 1H, ArH), 7.38 (d, *J* = 8.7 Hz, 1H, ArH), 7.49 (br s, 2H, NH_2_), 7.55–7.60 (m, 1H, ArH), 7.83 (t, *J* = 2.1 Hz, 1H, ArH), 7.96 (br s, 3H, NH_3_^+^), 9.46 (s, 1H, amide); ^13^C-NMR (151 MHz, DMSO-*d_6_*) δ (ppm) = 38.0 (CH_3_), 35.9, 48.9 (CH_2_), 111.1, 112.0, 119.0, 120.1, 122.7, 130.0, 130.3 (CH), 91.2, 92.0, 114.4, 115.4, 118.3, 129.9, 132.6, 140.5, 147.9, 150.0, 150.7, 158.8, 163.7, 163.7 (C); C_24_H_22_Cl_3_N_7_OS (562.90); MS (APCI pos.): *m*/*z* (%) = 526.3 (3) [M − 37]^+^, 397.2 (24) [M − 166]^+^, 373.3 (100) [M − 190]^+^, 356.2 (30) [M − 207]^+^, 330.2 (49) [M − 233]^+^, 320.2 (15) [M − 243]^+^, 128.1 (30) [M − 436]^+^; HPLC (isocr.): 97.5% at 254 nm and 98.1% at 280 nm, t_m_ = 1.0 min, t_ms_ = 15.6 min (ACN/buffer 70:30) (system 1); λ_max_: 265 nm, 319 nm.

*3,6-Diamino-4-(2-chloro-4-{[2-(dimethylamino)ethyl](methyl)amino}phenyl)-N-(3-chlorophenyl)-5-cyanothieno[2,3-b]pyridine-2-carboxamide***9af***(KuSaSch090):* According to Procedure F from 6-amino-4-(2-chloro-4-{[2-(dimethylamino)ethyl](methyl)amino}phenyl)-2-thioxo-1,2-dihydropyridine-3,5-dicarbonitrile (**7h**, 289 mg, 0.749 mmol), 2-chloro-N-(3-chlorophenyl)acetamide (161 mg, 0.789 mmol) and aqueous potassium hydroxide solution (10%, twice 387 µL, 0.750 mmol) for 30 min at 100 °C. After addition of ice water (25 mL), the mixture was extracted with dichloromethane (3 × 75 mL). The combined organic layers were washed with saturated sodium chloride solution (50 mL), dried over sodium sulfate and evaporated under reduced pressure. The residue was purified by column chromatography (dichloromethane/methanol/TEA 10:0.5:0.05) and the solvent of the corresponding fractions was evaporated under reduced pressure. The obtained solid was extracted with ethyl acetate (2 × 50 mL). The combined organic layers were washed with saturated sodium chloride solution (50 mL), dried over sodium sulfate and evaporated under reduced pressure. After crystallization from ethanol a yellow solid (25 mg, 6%) was obtained.

M.p.: Dec. starting at 156 °C; IR (KBr): ῦ_max_ 3435 cm^−1^ (NH), 2221 cm^−1^ (C≡N), 1635 cm^−1^ (C=O); ^1^H-NMR (500 MHz, DMSO-*d*_6_) δ (ppm) = 2.21 (s, 6H, 2 CH_3_), 2.44 (t, *J* = 7.0 Hz, 2H, CH_2_), 3.01 (s, 3H, CH_3_), 3.51 (t, *J* = 7.0 Hz, 2H, CH_2_), 5.91 (br s, 2H, NH_2_), 6.85 (dd, *J* = 8.8, 2.5 Hz, 1H, ArH), 6.90 (d, *J* = 2.5 Hz, 1H, ArH), 7.06–7.12 (ddd, *J* = 8.0, 2.2, 0.9 Hz, 1H, ArH), 7.31 (dd, *J* = 8.5, 7.2 Hz, 2H, ArH), 7.46 (br s, 2H, NH_2_), 7.55–7.61 (m, 1H, ArH), 7.83 (t, *J* = 2.0 Hz, 1H, ArH), 9.43 (s, 1H, amide); ^13^C-NMR (126 MHz, DMSO-*d_6_*) δ (ppm) = 38.1, 45.5 (2C) (CH_3_), 49.5, 55.6 (CH_2_), 110.6, 111.4, 119.0, 120.1, 122.7, 129.9, 130.2 (CH), 91.3, 91.1, 114.4, 115.4, 117.2, 132.1, 132.6, 140.5, 14.9, 150.2, 150.8, 158.8, 162.6, 162.6 (C); C_26_H_25_Cl_2_N_7_OS (554.49); HRMS (EI): *m*/*z* [M]^+•^ calcd. 553.12183, found 553.12129; MS (APCI pos.): *m*/*z* (%) = 554.4 (44) [M + H]^+^, 401.3 (100) [M − 153]^+^, 257.2 (15) [M − 297]^+^; HPLC (isocr.): 98.7% at 254 nm and 99.8% at 280 nm, t_m_ = 1.0 min, t_ms_ = 20.8 min (ACN/buffer 30:70) (system 1); λ_max_: 263 nm, 319 nm.

*tert-Butyl 4-(4-{3-amino-2-[(4-chlorophenyl)carbamoyl]-6,7-dihydro-5H-cyclopenta[b]thieno[3,2-e]-pyridin-4-yl}-3-chlorophenyl)piperazine-1-carboxylate***17a***(KuSaSch095):* According to Procedure F from *tert*-butyl 4-[3-chloro-4-(3-cyano-2-thioxo-2,5,6,7-tetrahydro-1*H*-cyclopenta[*b*]pyridin-4-yl)phenyl]-piperazine-1-carboxylate (**16a**, 462 mg, 0.980 mmol) and 2-chloro-*N*-(4-chlorophenyl)acetamide (200 mg, 0.980 mmol) in DMF (10 mL) and aqueous potassium hydroxide solution (10%, twice 506 µL, 0.980 mmol) for 1 h at 100 °C. Ice water (20 mL) was added and the mixture was extracted with ethyl acetate (3 × 50 mL). The combined organic layers were washed with saturated sodium chloride solution (30 mL), dried over sodium sulfate and evaporated under reduced pressure. The residue was purified by column chromatography (toluene/ethyl acetate 10:1) to yield a yellow solid (58 mg, 9%).

M.p.: 198–200 °C; IR (KBr): ῦ_max_ 3481 cm^−1^, 3330 cm^−1^ (NH); 2969 cm^−1^ (CH aliphatic), 1701 cm^−1^ (C=O urethane), 1632 cm^−1^(C=O amide); ^1^H-NMR (600 MHz, DMSO-*d*_6_) δ (ppm) = 1.44 (s, 9H, CH_3_), 2.09 (p, *J* = 7.6 Hz, 2H, CH_2_), 2.54–2.75 (m, 2H, CH_2_), 2.97–3.17 (m, 2H, CH_2_), 3.28–3.32 (m, 4H, CH_2_), 3.48 (t, *J* = 5.1 Hz, 4H, CH_2_), 5.90 (br s, 2H, NH_2_), 7.09 (dd, *J* = 8.7, 2.5 Hz, 1H, ArH), 7.21 (d, *J* = 2.5 Hz, 1H, ArH), 7.29 (d, *J* = 8.5 Hz, 1H, ArH), 7.36–7.39 (m, 2H, ArH), 7.67–7.73 (m, 2H, ArH), 9.56 (br s, 1H, amide); ^13^C-NMR (151 MHz, DMSO-*d_6_*) δ (ppm) = 28.0 (CH_3_), 22.4, 28.5, 33.8, 46.8 (2C) (CH_2_) (two further secondary carbon signals were not detectable after 1024 scans), 114.0, 115.0, 122.5 (2C), 128.2 (2C), 130.1 (CH), 79.0, 96.2, 120.7, 122.2, 126.9, 132.2, 133.7, 137.8, 139.2, 147.1, 151.8, 153.8, 158.1, 163.8, 167.2 (C); C_32_H_33_Cl_2_N_5_O_3_S (638.61); calcd. C 60.19, H 5.21, N 10.97, found C 60.22, H 5.01, N 10.81; MS (APCI pos.): *m*/*z* (%) = 638 (100) [M + H]^+^, 538.2 (37) [M − 99]^+^, 399.1 (12) [M − 238]^+^, 385.2 (30) [M − 252]^+^, 239.1 (31) [M − 398]^+^, MS (APCI neg.): *m*/*z* (%) = 636.2 (100) [M − H]^−^, 536.2 (42) [M − 101]^−^, 237.0 (13) [M − 400]^−^, HPLC (isocr.): 97.9% at 254 nm and 97.4% at 280 nm, t_m_ = 1.0 min, t_ms_ = 5.6 min (ACN/H_2_O 80:20) (system 1); λ_max_: 221 nm, 256 nm, 308 nm, 370 nm; HPLC (grad.): 98.7% at 254 nm, t_m_ = 1.0 min, t_ms_ = 15.7 min (system 3).

*3-Amino-N-(4-chlorophenyl)-4-phenyl-6,7-dihydro-5H-cyclopenta[b]thieno[3,2-e]pyridine-2-carboxamide***17b***(KuSaSch100):* According to Procedure F from 4-phenyl-2-thioxo-2,5,6,7-tetrahydro-1*H*-cyclopenta[*b*]pyridine-3-carbonitrile (**16b**, 76 mg, 0.30 mmol), 2-chloro-*N*-(4-chlorophenyl)acetamide (62 mg, 0.30 mmol) and aqueous potassium hydroxide solution (10%, twice 155 µL, 0.300 mmol) in DMF (1 mL) for 1 h. After addition of ice water (20 mL), the mixture was extracted with ethyl acetate (3 × 50 mL). The combined organic layers were washed with saturated sodium chloride solution (30 mL), dried over sodium sulfate and evaporated under reduced pressure. The residue was purified by column chromatography (petroleum ether/ethyl acetate 3:1). A yellow solid (59 mg, 47%) was obtained.

M.p.: Dec. starting at 229 °C (Lit.: 260 °C [41]); IR (KBr): ῦ_max_ 3477 cm^−1^, 3327 cm^−1^ (NH), 2954 cm^−1^ (CH aliphatic), 1637 cm^−1^ (C=O); ^1^H-NMR (600 MHz, DMSO-*d*_6_) δ (ppm) = 2.04–2.12 (m, 2H, CH_2_), 2.63–2.69 (m, 2H, CH_2_), 3.06–3.12 (m, 2H, CH_2_), 5.84–5.87 (br s, 2H, NH_2_), 7.29–7.38 (m, 2H, ArH), 7.42–7.51 (m, 2H, ArH), 7.53–7.62 (m, 3H, ArH), 7.67–7.73 (m, 2H, ArH), 9.55–9.58 (br s, 1H, amide); ^13^C-NMR (151 MHz, DMSO-*d_6_*) δ (ppm) = 22.6, 28.7, 33.9 (CH_2_), 122.4 (2C), 128.0 (2C), 128.2 (2C), 128.9 (3 C) confirmed with HSQC; 96.2, 119.8, 126.9, 132.9, 134.9, 137.8, 141.9, 147.1, 158.2, 163.8, 167.2; C_23_H_18_ClN_3_OS (419.93); calcd. C 65.79, H 4.32, N 10.01, found C 65.49, H 4.18, N 9.65; MS (APCI pos.): *m*/*z* (%) = 420.1 (100) [M + H]^+^, MS (APCI neg.): *m*/*z* (%): 418.1 (100) [M − H]^−^; HPLC (isocr.): 99.4% at 254 nm and 99.6% at 280 nm, t_m_ = 1.0 min, t_ms_ = 3.7 min (ACN/H_2_O 80:20) (system 1); λ_max_: 304 nm, 370 nm; HPLC (grad.): 95.8% at 254 nm, t_m_ = 1.0 min, t_ms_ = 14.1 min (system 3).

*3-Amino-N-(4-fluorophenyl)-4-phenyl-6,7-dihydro-5H-cyclopenta[b]thieno[3,2-e]pyridine-2-carboxamide***17c***(KuSaSch101):* According to Procedure F from 4-phenyl-2-thioxo-2,5,6,7-tetrahydro-1*H*-cyclopenta[*b*]pyridine-3-carbonitrile (**16b**, 94 mg, 0.38 mmol), 2-chloro-*N*-(4-fluorophenyl)acetamide (71 mg, 0.38 mmol) and aqueous potassium hydroxide solution (10%, twice 196 µL, 0.380 mmol) in DMF (1 mL) for 15 min. After addition of ice water (20 mL) the mixture was extracted with ethyl acetate (3 × 50 mL). The combined organic layers were washed with saturated sodium chloride solution (30 mL), dried over sodium sulfate and evaporated under reduced pressure. After crystallization of the residue from ethanol a yellow solid (56 mg, 36%) was obtained.

M.p.: Dec. starting at 252 °C; IR (KBr): ῦ_max_ 3505 cm^−1^, 3423 cm^−1^ (NH), 1671 cm^−1^ (C=O); ^1^H-NMR (600 MHz, DMSO-*d*_6_) δ (ppm) = 2.08 (p, *J* = 7.7 Hz, 2H, CH_2_), 2.64–2.69 (m, 2H, CH_2_), 3.09 (t, *J* = 7.7 Hz, 2H, CH_2_), 5.83 (br s, 2H, NH_2_), 7.11–7.19 (m, 2H, ArH), 7.42–7.47 (m, 2H, ArH), 7.53–7.62 (m, 3H, ArH), 7.63–7.68 (m, 2H, ArH), 9.51 (br s, 1H, amide); ^13^C-NMR (151 MHz, DMSO-*d_6_*) δ (ppm) = 23.0, 28.7, 33.9 (CH_2_), 114.8 (d, *J* = 22.1 Hz, 2C), 123.0 (d, *J* = 7.8 Hz, 2C), 128.0 (2C), 128.9 (3C) (CH), 96.3, 119.9, 132.9, 134.9, 135.1 (d, *J* = 2.7 Hz, C-C-C-C-F); 141.9, 146.8, 158.1 (d, *J* = 240.0 Hz, C-C-C-C-F), 158.2, 163.8, 167.0 (C); C_23_H_18_FN_3_OS (403.48); calcd. C 68.47, H 4.50, N 10.41, found C 68.66, H 4.45, N 10.38; MS (APCI pos.): *m*/*z* (%) = 404.1 (100) [M + H]^+^, MS (APCI neg.): *m*/*z* (%) = 402.1 (100) [M − H]^−^; HPLC (isocr.): 95.8% at 254 nm and 97.3% at 280 nm, t_m_ = 1.1 min, t_ms_ = 4.5 min (ACN/H_2_O 70:30) (system 1); λ_max_: 299 nm, 369 nm; HPLC (grad.): 95.2% at 254 nm, t_m_ = 1.0 min, t_ms_ = 13.2 min (system 3).

*3-Amino-N-(4-chlorophenyl)-4-phenyl-5,6,7,8-tetrahydrothieno[2,3-b]quinoline-2-carboxamide***17d***(KuSaSch105):* According to Procedure F from 4-phenyl-2-thioxo-1,2,5,6,7,8-hexahydroquinoline-3-carbonitrile (**16c**, 246 mg, 0.924 mmol), 2-chloro-*N*-(4-chlorophenyl)acetamide (188 mg, 0.921 mmol) and aqueous potassium hydroxide solution (10%, twice 475 µL, 0.921 mmol) in DMF (2 mL) for 2 h 30 min. After addition of ice water (20 mL) the mixture was extracted with ethyl acetate (3 × 50 mL). The combined organic layers were washed with saturated sodium chloride solution (30 mL), dried over sodium sulfate and evaporated under reduced pressure. Purification of the residue by column chromatography (petroleum ether/ethyl acetate 4:1) and subsequent crystallization from ethanol yielded orange crystals (35 mg, 9%).

M.p.: 233–234 °C; IR (KBr): ῦ_max_ 3478 cm^−1^, 3353 cm^−1^ (NH), 2929 cm^−1^, 2855 cm^−1^ (CH aliphatic), 1627 cm^−1^ (C=O); ^1^H-NMR (600 MHz, DMSO-*d*_6_) δ (ppm) = 1.65–1.72 (m, 2H, CH_2_), 1.79–1.86 (m, 2H, CH_2_), 2.36 (t, *J* = 6.5 Hz, 2H, CH_2_), 3.02 (t, *J* = 6.5 Hz, 2H, CH_2_), 5.70 (br s, 2H, NH_2_), 7.32–7.40 (m, 4H, ArH), 7.54–7.64 (m, 3H, ArH), 7.65–7.72 (m, 2H, ArH), 9.53–9.56 (s, 1H, amide); ^13^C-NMR (151 MHz, DMSO-*d_6_*) δ (ppm) = 22.0, 22.1, 25.6, 33.0 (CH_2_), 122.5 (2C), 127.9 (2C), 128.2 (2C), 128.8, 129.1 (2C) (CH); 96.0, 120.5, 126.7, 126.9, 134.9, 137.8, 145.5, 147.0, 156.4, 158.9, 163.9 (C); C_24_H_20_ClN_3_OS (433.95); calcd. C 66.43, H 4.65, N 9.68, found C 66.34, H 4.53, N 9.28; MS (APCI pos.): *m*/*z* (%) = 434.1 (100) [M + H]^+^, MS (APCI neg.): *m*/*z* (%) = 432.2 (100) [M − H]^−^; HPLC (isocr.): 95.3% at 254 nm and 97.7% at 280 nm, t_m_ = 0.6 min, t_ms_ = 4.4 min (ACN/H_2_O 80:20) (system 1); λ_max_: 299 nm, 373 nm; HPLC (grad.): 96.3% at 254 nm, t_m_ = 1.1 min, t_ms_ = 14.7 min (system 3).

*3-Amino-N-(4-chlorophenyl)-4-(3-methylphenyl)-5,6,7,8-tetrahydrothieno[2,3-b]quinoline-2-carbox-amide***17e***(KuSaSch107):* According to Procedure F from 2-thioxo-4-(3-methylphenyl)-1,2,5,6,7,8-hexa-hydroquinoline-3-carbonitrile (**16d**, 134 mg, 0.478 mmol), 2-chloro-*N*-(4-chlorophenyl)acetamide (98 mg, 0.48 mmol) and aqueous potassium hydroxide solution (10%, twice 248 µL, 0.480 mmol) in DMF (1 mL) at 100 °C for 2 h 30 min. After addition of ice water (20 mL) the mixture was extracted with ethyl acetate (3 × 50 mL). The combined organic layers were washed with saturated sodium chloride solution (30 mL), dried over sodium sulfate and evaporated under reduced pressure. The residue was purified by preparative HPLC (acetonitrile/water 70:30). A yellow solid (18 mg, 9%) was obtained.

M.p.: Dec. starting at 241 °C; IR (KBr): ῦ_max_ 3518 cm^−1^, 3411 cm^−1^ (NH), 1664 cm^−1^ (C=O); ^1^H-NMR (500 MHz, DMSO-*d*_6_) δ (ppm) = 1.64–1.73 (m, 2H, CH_2_), 1.78–1.87 (m, 2H, CH_2_), 2.35 (t, *J* = 6.4, 2H, CH_2_), 2.41 (s, 3H, CH_3_), 3.01 (t, *J* = 6.5 Hz, 2H, CH_2_), 5.74 (br s, 2H, NH_2_), 7.13–7.20 (m, 2H, ArH), 7.30–7.38 (m, 2H, ArH), 7.35–7.41 (m, 1H, ArH), 7.45–7.52 (m, 1H, ArH), 7.64–7.72 (m, 2H, ArH), 9.53 (br s, 1H, amide); ^13^C-NMR (126 MHz, DMSO-*d_6_*) δ (ppm) = 20.9, 22.0, 26.1, 33.0 (CH_2_), 122.5 (2C), 124.9, 128.2 (2C), 128.3, 129.0, 129.4 (CH), 95.9, 120.5, 126.7, 126.9, 134.8, 137.8, 138.5, 145.6, 147.1, 156.4, 158.8, 163.9 (C); C_25_H_22_ClN_3_OS (447.98); calcd. C 67.03, H 4.95, N 9.38, found C 67.02, H 4.83, N 9.09; MS (APCI pos.): *m*/*z* (%) = 448.1 (100) [M + H]^+^, MS (APCI neg.): *m*/*z* (%) = 446.1 (100) [M − H]^−^; HPLC (isocr.): 97.8% at 254 nm and 98.4% at 280 nm, t_m_ = 1.0 min, t_ms_ = 5.5 min (ACN/H_2_O 80:20) (system 1); λ_max_: 222 nm, 236 nm, 310 nm, 368 nm; HPLC (grad.): 97.6% at 254 nm, t_m_ = 1.1 min, t_ms_ = 15.4 min (system 3).

*3-Amino-N-(4-chlorophenyl)-4-(3-methylphenyl)-6,7-dihydro-5H-cyclopenta[b]thieno[3,2-e]pyridine-2-carboxamide***17f***(KuSaSch110):* According to Procedure F from 2-thioxo-4-(3-methylphenyl)-2,5,6,7-tetrahydro-1*H*-cyclopenta[*b*]pyridine-3-carbonitrile (**16e**, 251 mg, 0.942 mmol), 2-chloro-*N*-(4-chlorophenyl)acetamide (199 mg, 0.975 mmol) and aqueous potassium hydroxide solution (10%, twice 485 µL, 0.940 mmol) in DMF (1 mL) at 100 °C for 1 h. Ice water (20 mL) was then added and the mixture was extracted with ethyl acetate (3 × 50 mL). The combined organic layers were washed with saturated sodium chloride solution (30 mL), dried over sodium sulfate and evaporated under reduced pressure. The residue was crystallized from ethanol to yield a yellow solid (88 mg, 22%).

M.p.: 240–242 °C; IR (KBr): ῦ_max_ 3473 cm^−1^, 3316 cm^−1^ (NH), 1628 cm^−1^ (C=O); ^1^H-NMR (600 MHz, DMSO-*d*_6_) δ (ppm) = 2.07 (p, *J* = 7.7 Hz, 2H, CH_2_), 2.40 (s, 3H, CH_3_), 2.67 (t, *J* = 7.3 Hz, 2H, CH_2_), 3.09 (t, *J* = 7.6 Hz, 2H, CH_2_), 5.89 (br s, 2H, NH_2_), 7.18–7.28 (m, 2H, ArH), 7.33–7.40 (m, 3H, ArH), 7.45–7.50 (t, *J* = 7.6 Hz, 1H, ArH), 7.67–7.73 (m, 2H, ArH), 9.56 (s, 1H, amide); ^13^C-NMR (151 MHz, DMSO-*d_6_*) δ (ppm) = 20.9 (CH_3_), 22.6, 28.7, 33.9 (CH_2_), 122.4 (2C), 125.0, 128.2 (2C), 128.4, 128.8, 129.5 (CH); 96.1, 119.8, 126.9, 132.8, 134.8, 137.8, 138.3, 142.0, 147.1, 158.2, 163.8, 167.1 (C); C_24_H_22_ClN_3_OS (435.95); HRMS (ESI) *m*/*z* (%) = [M + H]^+^ calcd. 434.10884, found 434.10905 (75), [M + Na]^+^ calcd. 456.09078, found 456.09101 (30), [M + K]^+^ calcd. 472.06472, found 472.06492 (5), [2M + Na]^+^ calcd. 889.19234, found 889.19277 (60); MS (APCI pos.): *m*/*z* (%) = 434.1 (100) [M + H]^+^, MS (APCI neg.): *m*/*z* (%) = 432.1 (100) [M − H]^−^; HPLC (isocr.): 96.1% at 254 nm and 98.4% at 280 nm, t_m_ = 1.0 min, t_ms_ = 4.5 min (ACN/H_2_O 80:20) (system 1); λ_max_: 302 nm, 370 nm; HPLC (grad.): 98.4% at 254 nm, t_m_ = 1.1 min, t_ms_ = 14.6 min (system 3).

*tert-Butyl 3-amino-2-[(4-chlorophenyl)carbamoyl]-6-methyl-4-phenylthieno[2,3-b]pyridine-5-carboxylate***17g***(KuSaSch111):* According to Procedure F from *tert*-butyl 5-cyano-2-methyl-4-phenyl-6-thioxo-1,6-dihydropyridine-3-carboxylate (**16f**, 145 mg, 0.444 mmol), 2-chloro-*N*-(4-chlorophenyl)acetamide (91 mg, 0.45 mmol) and aqueous potassium hydroxide solution (10%, twice 232 µL, 0.450 mmol) in DMF (1 mL) for 2 h at 100 °C. After addition of ice water (20 mL) the mixture was extracted with ethyl acetate (3 × 75 mL). The combined organic layers were washed with saturated sodium chloride solution (30 mL), dried over sodium sulfate and evaporated under reduced pressure. The residue was purified by column chromatography (toluene/ethyl acetate 10:1). A yellow solid (155 mg, 70%) was obtained.

M.p.: 180–181 °C; IR (KBr): ῦ_max_ 3470 cm^−1^, 3325 cm^−1^ (NH), 2976 cm^−1^, 2927 cm^−1^ (CH aliphatic), 1722 cm^−1^ (C=O urethane), 1633 cm^−1^ (C=O amide); ^1^H-NMR (500 MHz, DMSO-*d*_6_) δ (ppm) = 1.16 (s, 9H, CH_3_), 2.61 (s, 3H, CH_3_), 5.77 (br s, 2H, NH_2_), 7.28–7.45 (m, 4H, ArH), 7.54–7.63 (m, 3H, ArH), 7.66–7.72 (m, 2H, ArH), 9.67 (br s, 1H, amide); ^13^C-NMR (126 MHz, DMSO-*d_6_*) δ (ppm) = 22.4, 27.0 (3C) (CH_3_); 122.6 (2C), 128.2 (2C), 128.5 (2C), 128.6 (2C), 129.3 (CH); 82.1, 97.2, 119.6, 127.1, 127.4, 133.3, 137.6, 143.3, 146.9, 154.5, 158.8, 163.6, 165.9; C_26_H_24_ClN_3_O_3_S (494.00); calcd. C 63.22, H 4.90, N 8.51, found C 63.06, H 4.84, N 8.20; MS (APCI pos.): *m*/*z* (%) = 494.2 (100) [M + H]^+^, MS (APCI neg.): 492.2 (100) [M − H]^−^, 390.3 (20) [M − 103]^−^; HPLC (isocr.): 98.9% at 254 nm and 99.2% at 280 nm, t_m_ = 1.0 min, t_ms_ = 4.1 min (ACN/H_2_O 80:20) (system 1); λ_max_: 222 nm, 241 nm, 276 nm, 309 nm, 367 nm; HPLC (grad.): 96.2% at 254 nm, t_m_ = 1.1 min, t_ms_ = 14.3 min (system 3).

*tert-Butyl 3-amino-2-[(4-chlorophenyl)carbamoyl]-6-methyl-4-(3-methylphenyl)thieno[2,3-b]pyridine-5-carboxylate***17h***(KuSaSch112):* According to Procedure F from tert-butyl 5-cyano-2-methyl-6-thioxo-4-(3-methylphenyl)-1,6-dihydropyridine-3-carboxylate (**16g**, 134 mg, 0.394 mmol), 2-chloro-*N*-(4-chlorophenyl)acetamide (82 mg, 0.39 mmol) and aqueous potassium hydroxide solution (10%, twice 201 µL, 0.390 mmol) in DMF (1 mL) for 15 min at 100 °C. After addition of ice water (20 mL) the mixture was extracted with ethyl acetate (3 × 50 mL). The combined organic layers were washed with saturated sodium chloride solution (30 mL), dried over sodium sulfate and evaporated under reduced pressure. The residue was purified by column chromatography (toluene/ethyl acetate 10:1). A yellow solid (100 mg, 52%) was obtained.

M.p.: Dec. starting at 186 °C; IR (KBr): ῦ_max_ 3474 cm^−1^, 3327 cm^−1^ (NH), 2976 cm^−1^, 2927 cm^−1^ (CH aliphatic), 1725 cm^−1^ (C=O urethane), 1632 cm^−1^ (C=O amide); ^1^H-NMR (600 MHz, DMSO-*d*_6_) δ (ppm) = 1.17 (s, 9H, CH_3_), 2.39 (s, 3H, CH_3_), 2.61 (s, 3H, CH_3_), 5.83 (br s, 2H, NH_2_), 7.17–7.24 (m, 2H, ArH), 7.34–7.39 (m, 2H, ArH), 7.39–7.43 (m, 1H, ArH), 7.44–7.49 (m, 1H, ArH), 7.66–7.72 (m, 2H, ArH), 9.67 (br s, 1H, amide); ^13^C-NMR (151 MHz, DMSO-*d_6_*) δ (ppm) = 20.8, 22.4, 26.9 (3C) (CH_3_); 122.6 (2C); 125.6, 128.2 (2C); 128.4, 129.0, 129.9 (CH), 82.0, 97.0, 119.5, 127.1, 127.4, 133.3, 137.7, 137.9, 143.4, 146.9, 154.5, 158.8, 163.6, 165.9 (C); C_27_H_26_ClN_3_O_3_S (508.03); calcd. C 63.83, H 5.16, N 8.27, found C 63.81, H 5.09, N 8.17; MS (APCI pos.): *m*/*z* (%) = 508.2 (100) [M + H]^+^, 359.4 (39) [M − 148]^+^, 341.3 (60) [M − 166]^+^, MS (APCI neg.): *m*/*z* (%) = 506.2 (63) [M − H]^−^, 390.3 (100) [M − 117]^−^; HPLC (isocr.): 97.1% at 254 nm and 98.0% at 280 nm, t_m_ = 1.0 min, t_ms_ = 4.9 min (ACN/H_2_O 80:20) (system 1); λ_max_: 221 nm, 303 nm, 371 nm; HPLC (grad.): 97.5% at 254 nm, t_m_ = 1.0 min, t_ms_ = 14.9 min (system 3).

*3-Amino-2-[(4-chlorophenyl)carbamoyl]-6-methyl-4-(3-methylphenyl)thieno[2,3-b]pyridine-5-carboxylic acid***17i***(KuSaSch114):* According to Procedure H from *tert*-butyl 3-amino-2-[(4-chlorophenyl)-carbamoyl]-6-methyl-4-(3-methylphenyl)thieno[2,3-*b*]pyridine-5-carboxylate (**17h**, 40 mg, 0.078 mmol) in dried dichloromethane (2 mL) and trifluoroacetic acid (2 mL) under an argon atmosphere for 20 h at room temperature. Afterwards, the mixture was evaporated under reduced pressure. After addition of water (50 mL) the residue was extracted with ethyl acetate (3 × 50 mL). The combined organic layers were washed with saturated sodium chloride solution (30 mL), dried over sodium sulfate and evaporated under reduced pressure. The residue was purified by crystallization from ethanol (70%). A yellow solid (30 mg, 84%) was obtained.

M.p.: 237–240 °C; IR (KBr): ῦ_max_ 3482 cm^−1^, 3358 cm^−1^ (NH), 2925 cm^−1^ (CH aliphatic), 2500 cm^−1^ (br, OH), 1686 cm^−1^ (C=O), 1624 cm^−1^ (C=O); ^1^H-NMR (600 MHz, DMSO-*d*_6_) δ (ppm) = 2.38 (s, 3H, CH_3_), 2.63 (s, 3H, CH_3_), 5.80 (br s, 2H, NH_2_), 7.19–7.25 (m, 2H, ArH), 7.34–7.40 (m, 3H, ArH), 7.42–7.47 (m, 1H, ArH), 7.66–7.72 (m, 2H, ArH), 9.66 (br s, 1H, amide), 13.35 (br s, 1H, COOH); ^13^C-NMR (151 MHz, DMSO-*d_6_*) δ (ppm) = 20.9, 22.7 (CH_3_); 122.6 (2C), 125.6, 128.2 (2C), 128.4, 128.9, 129.9 (CH); 96.9, 119.5, 127.1, 128.0, 133.5, 137.7, 137.8, 143.0, 147.0, 154.3, 158.5, 163.6, 168.4 (C); C_23_H_18_ClN_3_O_3_S (451.93); HRMS (ESI) *m*/*z* (%) = [M + H]^+^ calcd. 452.08302, found 452.08333 (93), [M + Na]^+^ calcd. 474.06496, found 474.06536 (100), [2M + Na]^+^ calcd. 925.14070, found 925.14130 (58); MS (APCI pos.): *m*/*z* (%) = 452.2 (27) [M + H]^+^, MS (APCI neg.): *m*/*z* (%) = 450.2 (100) [M − H]^−^, 283.2 (82) [M − 168]^−^, 255.2 (70) [M − 196]^−^; HPLC (isocr.): 99.0% at 254 nm and 99.1% at 280 nm, t_m_ = 1.1 min, t_ms_ = 4.8 min (ACN/buffer 50:50) (system 1); λ_max_: 300 nm, 371 nm; HPLC (grad.): 99.3% at 254 nm, t_m_ = 1.1 min, t_ms_ = 7.1 min.

*3-Amino-2-[(4-chlorophenyl)carbamoyl]-6-methyl-4-phenylthieno[2,3-b]pyridine-5-carboxylic acid***17j***(KuSaSch115):* According to Procedure H from *tert*-butyl 3-amino-2-[(4-chlorophenyl)carbamoyl]-6-methyl-4-phenylthieno[2,3-*b*]pyridine-5-carboxylate (**17g**, 63 mg, 0.13 mmol) in dried dichloromethane (3 mL) and trifluoroacetic acid (3 mL) under an argon atmosphere at room temperature for 24 h. Afterwards, the mixture was evaporated under reduced pressure. After addition of water (50 mL) the residue was extracted with ethyl acetate (3 x 50 mL). The combined organic layers were washed with saturated sodium chloride solution (30 mL), dried over sodium sulfate and evaporated under reduced pressure. The residue was purified by crystallization from ethanol (70%). A yellow solid (39 mg, 68%) was obtained.

M.p.: Dec. starting at 260 °C; IR (KBr): ῦ_max_ 3487 cm^−1^, 3427 cm^−1^, 3357 cm^−1^ (NH), 2443 cm^−1^ (br, OH), 1681 cm^−1^, 1625 cm^−1^ (C=O); ^1^H-NMR (600 MHz, DMSO-*d*_6_) δ (ppm) = 2.64 (s, 3H, CH_3_), 5.76 (br s, 2H, NH_2_), 7.34–7.39 (m, 2H, ArH), 7.39–7.46 (m, 2H, ArH), 7.53–7.60 (m, 3H, ArH), 7.66–7.72 (m, 2H, ArH), 9.67 (br s, 1H, amide), 13.36 (br s, 1H, COOH); ^13^C-NMR (151 MHz, DMSO-*d_6_*) δ (ppm) = 22.7 (CH_3_); 122.6 (2C), 128.2 (2C), 128.5 (4C), 129.3 (CH); 97.1, 119.6, 127.1, 128.0, 133.5, 137.7, 142.9, 147.0, 154.3, 158.6, 163.6, 168.4 (C); C_22_H_16_ClN_3_O_3_S (437.90); HRMS (ESI) *m*/*z* (%) = [M + H]^+^ calcd. 438.06737, found 438.06772 (97), [M + Na]^+^ calcd. 460.04931, found 460.04967 (100), [2M + Na]^+^ calcd. 897.10940, found 897.10977 (49); MS (APCI pos.): *m*/*z* (%) = 438.1 (100) [M + H]^+^, MS (APCI neg.): *m*/*z* (%) = 436.1 (100) [M − H]^−^; HPLC (isocr.): 97.7% at 254 nm and 98.6% at 280 nm, t_m_ = 1.1 min, t_ms_ = 4.4 min (ACN/buffer 50:50) (system 1); λ_max_: 298 nm, 371 nm; HPLC (grad.): 98.6% at 254 nm, t_m_ = 1.1 min, t_ms_ = 7.8 min (system 3).

*tert-Butyl 4-(4-{3-amino-2-[(4-chlorophenyl)carbamoyl]-5,6,7,8-tetrahydrothieno[2,3-b]quinolin-4-yl}-3-chlorophenyl)piperazine-1-carboxylate***17k***(KuSaSch118):* According to Procedure F from tert-butyl 4-[3-chloro-4-(3-cyano-2-thioxo-1,2,5,6,7,8-hexahydroquinolin-4-yl)phenyl]piperazine-1-carboxylate (**16h**, 260 mg, 0.536 mmol), 2-chloro-N-(4-chlorophenyl)acetamide (110 mg, 0.539 mmol) and aqueous potassium hydroxide solution (10%, twice 281 µL, 0.544 mmol) in DMF (2 mL) for 30 min at 100 °C. After addition of ice water (20 mL) the mixture was extracted with ethyl acetate (3 × 50 mL). The combined organic layers were washed with saturated sodium chloride solution (30 mL) and dried over sodium sulfate. After evaporation under reduced pressure the residue was purified by column chromatography (petroleum ether/ethyl acetate 3:1). A yellow solid (127 mg, 36%) was obtained.

M.p.: 226–228 °C; IR (KBr): ῦ_max_ 3474 cm^−1^, 3324 cm^−1^ (NH), 2923 cm^−1^, 2859 cm^−1^ (CH aliphatic), 1660 cm^−1^, 1638 cm^−1^(C=O); ^1^H-NMR (500 MHz, DMSO-*d*_6_) δ (ppm) = 1.44 (s, 9H, CH_3_), 1.64–1.76 (m, 2H, CH_2_), 1.83 (p, *J* = 6.4 Hz, 2H, CH_2_), 2.24–2.42 (m, 2H, CH_2_), 2.94–3.08 (m, 2H, CH_2_), 3.27–3.32 (m, 4H, CH_2_), 3.45–3.52 (m, 4H, CH_2_), 5.82 (br s, 2H, NH_2_), 7.11 (dd, *J* = 8.7, 2.5 Hz, 1H, ArH), 7.21 (d, *J* = 2.5 Hz, 1H, ArH), 7.25 (d, *J* = 8.5 Hz, 1H, ArH), 7.32–7.39 (m, 2H, ArH), 7.66–7.72 (m, 2H, ArH), 9.54 (br s, 1H, amide); ^13^C-NMR (126 MHz, DMSO-*d_6_*) δ (ppm) = 28.0 (CH_3_); 22.0, 22.0, 25.7, 32.9, 46.8 (2C) (CH_2_) (two further secondary carbon signals were not detectable after 480 scans); 114.2, 115.1, 122.5 (2C), 128.2 (2C), 130.1 (CH); 79.0, 96.1, 121.2, 122.1, 126.9, 127.6, 132.3, 137.8, 142.8, 147.1, 151.8, 153.8, 156.4, 159.0, 163.9 (C); C_33_H_35_Cl_2_N_5_O_3_S (652.64); HRMS (ESI) *m*/*z* (%) = [M + H]^+^ calcd. 652.19104, found 652.19134 (47), [M + Na]^+^ calcd. 674.17299, found 674.17314 (100), [M + K]^+^ calcd. 690.14692, found 690.14672 (15); MS (APCI pos.): *m*/*z* (%) = 652.3 (78) [M + H]^+^, 552.2 (20) [M − 99]^+^, 447.4 (44) [M − 204]^+^, 339.2 (22) [M − 312]^+^, MS (APCI neg.): *m*/*z* (%) = 650.2 (100) [M − H]^−^, 550.1 (23) [M − 101]^−^; HPLC (isocr.): 95.8% at 254 nm and 97.0% at 280 nm, t_m_ = 1.0 min, t_ms_ = 6.8 min (ACN/H_2_O 80:20) (system 1); λ_max_: 262 nm, 299 nm, 374 nm; HPLC (grad.): 96.7% at 254 nm, t_m_ = 1.1 min, t_ms_ = 15.9 min (system 3).

*tert-Butyl 4-(4-{3-amino-2-[(4-chlorophenyl)carbamoyl]-6-methylthieno[2,3-b]pyridin-4-yl}-3-chloro-phenyl)piperazine-1-carboxylate***17l***(KuSaSch122):* According to Procedure F from tert-butyl 4-[3-chloro-4-(3-cyano-6-methyl-2-thioxo-1,2-dihydropyridin-4-yl)phenyl]-piperazine-1-carboxylate (**16i**, 439 mg, 0.987 mmol), 2-chloro-*N*-(4-chlorophenyl)acetamide (201 mg, 0.985 mmol) and aqueous potassium hydroxide solution (10%, twice 506 µL, 0.990 mmol) in DMF (15 mL) for 2 h at 100 °C. After addition of ice water (20 mL) and ethyl acetate (25 mL) a solid precipitated at the interface of the aqueous and the organic layers. It was filtered off and crystallized from toluene. The mixture was stored at 4 °C for three days. An impurity precipitated, which was filtered off and discarded. The solvent of the organic phase was evaporated under reduced pressure. Afterwards, the residue was purified by crystallization from ethyl acetate. A yellow solid (349 mg, 58%) was obtained.

M.p.: 238–240 °C; IR (KBr): ῦ_max_ 3478 cm^−1^, 3325 cm^−1^ (NH), 2968 cm^−1^, 2912 cm^−1^, 2862 cm^−1^(CH aliphatic), 1659 cm^−1^ (C=O), 1640 cm^−1^(C=O); ^1^H-NMR (500 MHz, DMSO-*d*_6_) δ (ppm) = 1.44 (s, 9H, CH_3_), 2.62 (s, 3H, CH_3_), 3.27–3.32 (m, 4H, CH_2_), 3.45–3.52 (m, 4H, CH_2_), 5.95 (br s, 2H, NH_2_), 7.05–7.11 (m, 2H, ArH), 7.19 (d, *J* = 2.4 Hz, 1H, ArH), 7.33 (d, *J* = 8.6 Hz, 1H, ArH), 7.33–7.40 (m, 2H, ArH), 7.68–7.73 (m, 2H, ArH), 9.60 (br s, 1H, amide); ^13^C-NMR (126 MHz, DMSO-*d_6_*) δ (ppm) = 23.9, 28.0 (3C) (CH_3_); 46.8 (2C) (CH_2_) (two further secondary carbon signals were not detectable after 480 scans), 113.8, 115.0, 121.9, 122.5 (2C), 128.2 (2C), 130.7 (CH); 79.0, 96.2, 120.7, 123.8, 127.0, 132.4, 137.8, 143.8, 147.1, 151.9, 153.8, 159.0, 159.0, 163.8 (C); C_30_H_31_Cl_2_N_5_O_3_S (612.57); HRMS (ESI) *m*/*z* (%) = [M + H]^+^ calcd. 612.15974, found 612.15998 (38), [M + Na]^+^ calcd. 634.14169, found 634.14195 (100), [M + K]^+^ calcd. 650.11562, found 650.11533 (10); MS (APCI pos.): *m*/*z* (%) = 612.2 (54) [M + H]^+^, 447.3 (86) [M − 164]^+^, MS (APCI neg.): *m*/*z* (%) = 610.2 (100) [M − H]^−^, 446.2 (10) [M − 165]^−^; HPLC (isocr.): 98.7% at 254 nm and 98.4% at 280 nm, t_m_ = 0.8 min, t_ms_ = 4.6 min (ACN/H_2_O 80:20) (system 1); λ_max_: 260 nm, 296 nm, 370 nm; HPLC (grad.): 96.9% at 254 nm, t_m_ = 1.1 min, t_ms_ = 14.7 min (system 3).

### 3.3. KinaseGlo Plus Assay for Inhibition of PfGSK-3 and HsGSK-3

A commercial luminescence-based kinase assay was used to investigate the enzymatic activity of kinases and to test potential kinase inhibitors. The KinaseGlo Plus assay (KinaseGlo Plus, Promega, Madison, WI, USA) provides a linear luminescence signal at ATP concentrations up to 100 μM and offers high signal stability. First, the kinase to be investigated and a corresponding substrate in the presence of ATP are used to perform the kinase reaction. During the substrate phosphorylation, ATP is consumed depending on the kinase activity. Subsequently, luminescence is generated by means of luciferase which is contained in the KinaseGlo Reagent. The intensity of the luminescence signal depends on the amount of ATP remaining in the reaction mixture. The luminescence signal is inversely proportional to the enzymatic activity of the kinase. The following approach was used for the kinase reaction: *rPf*GSK-3 (see 3.4) or *rHs*GSK-3 (Promega, Madison, WI, USA) (20 ng), GS-1 substrate (Promega, Madison, WI, USA) (12 µg) and ATP (6 µg) (Ultra Pure, Promega, Madison, WI, USA) in 1× kinase reaction buffer (40 mM Tris-HCl pH 7.5, 20 mM MgCl_2_, 0.1 mg/mL BSA; final reaction volume 5 µL). The kinase reaction was performed for 30 min at 30 °C. The reaction mixture was then mixed with KinaseGlo Reagent (5 µL) and incubated for 10 min at room temperature. The mixture was transferred to a white 384-well plate (NUNC, ThermoFisher, Waltham, MA, USA) and the luminescence signal was measured in an EnVision Multilable Plate Reader (PerkinElmer, Waltham, MA, USA; integration time 0.5 sec/well). For the investigation, potential kinase inhibitors could be added directly to the kinase reaction. All kinase inhibitors were dissolved in DMSO. By appropriate pre-dilution of the inhibitors in kinase reaction buffer the final DMSO concentration in the kinase reaction was at most 1%. As negative control 1% DMSO without substance was measured. Since the luminescence signal is inversely proportional to the ATP concentration, a control reaction (reaction without kinase) was measured simultaneously. The difference between kinase reaction and control reaction corresponds to the kinase activity. Using GraphPad Prism (version 6) the measured values were plotted and normalized to the DMSO control.

### 3.4. Production of Recombinant PfGSK-3

*Pf*GSK-3 was cloned into pOPIN J expression vector [42], using ligation independent cloning (InFusion, Takara Clontech). The expression vector was transformed into *E. coli* BL21 (DE3)-RIL and expression of recombinant *Pf*GSK-3-6xHis-GST was induced by incubation with 2 mM IPTG for 5 h at 37 °C. Recombinant protein was purified by affinity chromatography using glutathione resin (GenScript, Piscataway Township, NJ). Elution was achieved by digestion with human rhino virus 3C protease (provided by Dr. Sophia Reindl, BNITM).

### 3.5. HEK293T-Cell Based Cytotoxicity Assay

Cytotoxic effects of test compounds on eukaryotic cells were investigated using a HEK293T cell viability assay. First, the cell density of a HEK293T culture was determined. The cells were then seeded into a black 96-well plate (lumox^®^ multiwell 96 cell culture plate, Sarstedt, DE; 2.5 × 104 cells per well in a volume of 200 μL) and incubated for 24 h at 37 °C and 5% CO_2_. The supernatant was then aspirated and fresh culture medium was added to 200 μL into which the test compounds were previously added. All substances were previously dissolved in DMSO. The final DMSO concentration in the assay was 0.5% at maximum. In addition, 0.5% DMSO served as negative control and 10% DMSO as positive control. After addition of the test compounds, the HEK293T cell culture was incubated for another 48 h at 37 °C and 5% CO_2_. The viability of the cells was then examined by adding PrestoBlue^®^ Cell Viability Agent (Invitrogen, Carlsbad, CA, USA). The supernatant was removed, 10% PrestoBlue^®^ was added in preheated PBS and the culture was incubated for 30 min at 37 °C. In cells with an active metabolism, the PrestoBlue dye is reduced, causing it to fluorescence. The fluorescence (λ_ex_ = 560 nm, λ_em_ = 590 nm) was measured in an EnVision Multilable Plate Reader (Perkin Elmer, Waltham, MA, USA; integration time 0.1 sec/well).

### 3.6. Viability Screening of Antiplasmodial Activity

The proliferation of Plasmodium parasites was analyzed by quantifying the DNA content in infected erythrocytes [43]. Since red blood cells contain no DNA, the proliferation of the parasites in culture can be evaluated by staining the parasite DNA with SYBR-gold (Invitrogen, Carlsbad, CA, USA). To this purpose, the parasitemia of a parasite culture was first determined by flow cytometry (ACEA Novo Cyte 1000, ACEA Biosciences, San Diego, CA, USA) as previously described [44]. Subsequently, parasitemia and hematocrit were adjusted to 0.1% and 0.2%, respectively and the culture was distributed on opaque black 96-well plates (lumox^®^ multiwell 96 cell culture plate, Sarstedt, DE; 200 μL culture per well). To investigate potentially antiplasmodial substances, these were dissolved in DMSO and then added to the culture. The final DMSO concentration was 0.5% at maximum. DMSO without substance was used as negative control. The culture was then run under standard conditions [45] (Parasites in RPMI complete medium (1.587% (*m*/*v*) RPMI 1640, 12 mM NaHCO_3_, 6 mM d-glucose, 0.2 mM hypoxanthine, 0.4 mM gentamicin, 0.5% (*w*/*v*) Albumax II, sterile-filtered in H_2_O and adjusted to pH 7.2 with NaOH) with 5% human erythrocytes of blood group 0 Rh+ in the presence of CO_2_ (5%), O_2_ (1%) and N_2_ (94%) at 37 °C) for 96 h and gassed daily. The proliferation of the parasites after 96 h was quantified on the basis of the DNA content by a SYBR-gold staining. For this purpose, 100 μL of the culture supernatant were first removed from each well, then 100 μL lysis buffer (20 mM Tris; 5 mM EDTA; 0.008% saponin; 0.08 triton-X-100; 1x SYBR Gold) were added and re-suspended. Staining was performed by incubation for one hour in the dark at room temperature. The DNA content was then measured in an EnVision Multilable Plate Reader (Perki-nElmer, Waltham, MA, USA). To calculate IC_50_ values, the measured values were normalized to uninfected erythrocytes and plotted in GraphPad Prism (version 6) (GraphPad Software, San Diego, CA, USA) as % DMSO control. Dose-response curves were generated using nonlinear regression (curve fit > dose-response inhibition > (log) inhibitor vs. normalized response—variable slope.

## 4. Conclusions

The 4-arylthieno[2,3-*b*]pyridine-2-carboxamides derived from analogous ketones are a class of substances with potent antiparasitic activity against erythrocytic forms of the malaria pathogen *Plasmodium falciparum*. In contrast to the corresponding ketones, these carboxamides are only weak inhibitors of the plasmodial enzyme *Pf*GSK-3, which probably does not represent the biological target structure of the new compound class. Molecular structure modifications revealed that the substituents in the 5-and 6-positions of the heterocyclic parent scaffold can be exchanged for aliphatic residues, whereas an aromatic substituent on the nitrogen atom at the carboxamide function is essential. The most potent antiplasmodial representatives of the substance class such as **17f** inhibit the pathogens with IC_50_ values in the two-digit nanomolar range and exhibit very high selectivity indices (>100). These structures should therefore be excellent starting points for the development of new antimalarial agents. Certain substituents on the parent scaffold may be swapped during this development process. It must be kept in mind that such structural modifications can have a major impact on the metabolic and chemical stability of the congeners. The chemical stability aspects in particular are currently the subject of ongoing investigations.

## Figures and Tables

**Table 1:** 3,6-Diamino-5-cyanothieno[2,3-*b*]pyridine-2-carboxamides **9** ^a^.

Compound	R^1^	R^2^	R^3^	R^4^
**9a**	H	Cl	H	3-Cl-Ph
**9b**	H	F	H	2-Cl-Ph
**9c**	H	Cl	H	2-Cl-Ph
**9d**	H	Cl	H	4-F-Ph
**9e**	H	Me	H	4-Cl-Ph
**9f**	H	Me	H	2-Cl-Ph
**9g**	H	Me	H	4-F-Ph
**9h**	H	F	H	4-Cl-Ph
**9i**	H	F	H	4-F-Ph
**9j**	H	Me	H	Methyl
**9k**	H	Me	H	Heptyl
**9l**	H	Me	H	tert-Propyl
**9m**	H	Me	H	Cyclopropyl
**9n**	H	Me	H	*N*-(2-morpholinoethyl)
**9o**	H	Me	H	*N*-(2-cyclopropylethyl)
**9p**	*N*-BocPiperazino	H	Cl	3-Cl-Ph
**9q**	Piperazino (as hydrochloride)	H	Cl	3-Cl-Ph
**9r**	Morpholino	H	Cl	3-Cl-Ph
**9s**	Pyrrolidino	H	Cl	3-Cl-Ph
**9t**	Pyrrolidino	H	Cl	2-Cl-Ph
**9u**	Morpholino	H	Cl	2-Cl-Ph
**9v**	Morpholino	H	Cl	4-Cl-Ph
**9w**	*N*-[2-(*N*-Boc-amino)ethyl)]-*N*-(methyl)amino	H	Cl	3-Cl-Ph
**9x**	Morpholino	H	Cl	4-F-Ph
**9y**	*N*-[2-(*N*-Boc-amino)ethyl)]-*N*-(methyl)amino	H	Cl	4-Cl-Ph
**9z**	Pyrrolidino	H	Cl	4-Cl-Ph
**9aa**	Pyrrolidino	H	Cl	4-F-Ph
**9ab**	*N*-(2-dimethylaminoethyl)-*N*-(methyl)amino	H	Cl	4-F-Ph
**9ac**	*N*-[2-(*N*-Boc-amino)ethyl)]-*N*-(methyl)amino	H	Cl	4-F-Ph
**9ad**	*N*-[2-(*N*-Boc-amino)ethyl)]-*N*-(methyl)amino	H	Cl	2-Cl-Ph
**9ae**	*N*-(2-aminoethyl)-*N*-(methyl)amino (as hydrochloride)	H	Cl	3-Cl-Ph
**9af**	*N*-(2-dimethylaminoethyl)-*N*-(methyl)amino	H	Cl	3-Cl-Ph

^a^ For position of residues R^1^–R^4^ refer to Scheme 1.

**Table 2:** 6-Alkyl substituted thieno[2,3-*b*]pyridine-2-carboxamides **17** ^a^.

Compound	R^1^	R^2^	R^3^	R^4^	R^5^	R^6^
**17a**	*N*-BocPiperazino	H	Cl	-(CH_2_)_3_-		Cl
**17b**	H	H	H	-(CH_2_)_3_-		Cl
**17c**	H	H	H	-(CH_2_)_3_-		F
**17d**	H	H	H	-(CH_2_)_4_-		Cl
**17e**	H	Methyl	H	-(CH_2_)_4_-		Cl
**17f**	H	Methyl	H	-(CH_2_)_3_-		Cl
**17g**	H	H	H	(H_3_C)_3_COOC-	Methyl	Cl
**17h**	H	Methyl	H	(H_3_C)_3_COOC-	Methyl	Cl
**17i**	H	Methyl	H	-COOH	Methyl	Cl
**17j**	H	H	H	-COOH	Methyl	Cl
**17k**	*N*-BocPiperazino	H	Cl	-(CH_2_)_4_-		Cl
**17l**	*N*-BocPiperazino	H	Cl	H	Methyl	Cl

^a^ For position of residues R^1^–R^6^ refer to Scheme 4.

**Table 3:** Biological activity of thieno[2,3-*b*]pyridine-2-carboxamides.

Compound	Parasitemia (3D7) at 3 µM [%] ^a^	Parasitemia (3D7) at 0.3 µM [%] ^a^	HEK293T Viability at 30 µM [%] ^a^	r*Pf*GSK-3 Activity at 10 µM [%] ^a^
**9a**	3.7 ± 3.6	23.1 ± 10.8	123.0 ± 8.4	80.3 ± 18.2
**9b**	46.0 ± 19.7	96.0 ± 1.9	101.1 ± 19.6	101.9 ± 16.5
**9c**	40.0 ± 15.9	69.2 ± 26.0	99.3 ± 5.3	99.0 ± 14.1
**9d**	3.5 ± 3.5	88.5 ± 17.6	83.0 ± 21.6	69.7 ± 16.6
**9e**	5.6 ± 5.5	9.7 ± 6.8	83.3 ± 20.6	105.6 ± 6.5
**9f**	51.6 ± 15.7	80.2 ± 20.3	93.6 ± 29.9	90.9 ± 14.2
**9g**	3.1 ± 2.7	63.8 ± 16.5	67.8 ± 8.1	74.8 ± 21.7
**9h**	3.9 ± 2.9	63.2 ± 19.5	90.7 ± 13.2	103.5 ± 13.3
**9i**	6.1 ± 4.9	65.8 ± 24.6	39.9 ± 14.6	70.9 ± 19.8
**9j**	107.8 ± 24.2	74.6 ± 28.6	67.2 ± 21.5	15.4 ± 10.7
**9k**	84.0 ± 27.0	72.7 ± 28.2	54.3 ± 19.1	84.7 ± 10.6
**9l**	79.8 ± 20.3	80.6 ± 22.0	70.6 ± 23.0	54.1 ± 15.5
**9m**	88.0 ± 18.2	74.9 ± 15.7	74.3 ± 31.1	32.7 ± 7.3
**9n**	91.6 ± 19.2	58.7 ± 25.9	70.9 ± 23.6	32.9 ± 12.9
**9o**	40.8 ± 14.3	62.5 ± 23.5	25.2 ± 18.7	60.0 ± 11.2
**9p**	5.4 ± 3.2	48.3 ± 18.0	103.9 ± 14.1	111.0 ± 12.1
**9q**	4.9 ± 3.0	6.5 ± 3.1	−13.1 ± 5.4	72.6 ± 12.8
**9r**	2.8 ± 3.6	45.7 ± 17.2	104.7 ± 10.0	108.4 ± 16.5
**9s**	4.8 ± 5.0	78.3 ± 15.2	114.0 ± 40.3	112.9 ± 15.9
**9t**	60.6 ± 23.7	68.9 ± 27.3	99.0 ± 22.6	105.1 ± 6.6
**9u**	53.1 ± 29.6	74.5 ± 21.2	87.0 ± 26.7	109.9 ± 6.2
**9v**	6.2 ± 6.1	51.8 ± 38.6	92.3 ± 18.8	91.8 ± 18.7
**9w**	3.6 ± 2.4	50.1 ± 37.9	76.1 ± 17.3	95.3 ± 8.6
**9x**	8.7 ± 6.6	59.0 ± 43.6	86.1 ± 16.0	90.2 ± 8.9
**9y**	5.5 ± 4.6	36.6 ± 27.4	81.7 ± 19.6	110.0 ± 7.7
**9z**	6.4 ± 5.8	73.1 ± 22.1	91.9 ± 13.6	108.6 ± 4.9
**9aa**	5.3 ± 2.6	61.8 ± 27.7	74.0 ± 8.6	101.1 ± 3.7
**9ab**	1.6 ± 11.0	16.7 ± 19.5	−1.4 ± 13.1	112.2 ± 13.9
**9ac**	7.5 ± 5.9	30.1 ± 14.2	55.8 ± 25.1	85.7 ± 8.2
**9ad**	13.3 ± 9.9	57.7 ± 24.1	76.3 ± 24.1	95.0 ± 10.7
**9ae**	2.7 ± 7.8	1.7 ± 4.2	−8.5 ± 7.4	100.9 ± 20.2
**9af**	4.5 ± 7.4	5.8 ± 4.7	−7.4 ± 6.4	107.1 ± 11.2
**17a**	8.0 ± 10.2	50.5 ± 26.3	93.9 ± 6.5	101.8 ± 4.0
**17b**	7.7 ± 14.7	7.2 ± 11.3	92.7 ± 11.7	104.0 ± 15.5
**17c**	9.9 ± 17.1	23.6 ± 16.5	72.4 ± 23.4	102.8 ± 6.2
**17d**	8.4 ± 17.5	6.2 ± 5.7	24.4 ± 14.9	106.5 ± 10.6
**17e**	4.7 ± 13.5	5.0 ± 11.6	57.9 ± 20.6	103.1 ± 10.7
**17f**	1.0 ± 14.9	9.1 ± 16.1	72.4 ± 23.4	102.8 ± 6.2
**17g**	9.8 ± 15.1	11.7 ± 10.1	56.9 ± 31.3	100.8 ± 5.5
**17h**	2.3 ± 14.5	11.8 ± 12.3	44.1 ± 22.5	105.9 ± 5.3
**17i**	62.4 ± 21.5	99.8 ± 18.8	87.6 ± 19.6	106.2 ± 8.0
**17j**	97.1 ± 19.9	76.1 ± 23.1	95.8 ± 9.8	108.1 ± 10.4
**17k**	2.6 ± 9.0	30.0 ± 24.9	84.3 ± 14.3	105.8 ± 18.7
**17l**	1.0 ± 6.9	25.5 ± 14.4	68.4 ± 24.3	103.0 ± 12.9

^a^ calculated on DMSO controls.

**Table 4:** Biological activity of thieno[2,3-*b*]pyridine-2-carboxamides which lack antiplasmodial activity but are selective *Pf*GSK-3 inhibitors.

Compound	r*Pf*GSK-3 Activity at 10 µM [%] ^a^	r*Hs*GSK-3 Activity at 10 µM [%] ^a^
**9j**	15.4 ± 10.7	87.6 ± 7.4
**9m**	32.7 ± 7.3	93.8 ± 2.1
**9n**	32.9 ± 12.9	93.9 ± 3.2

^a^ calculated on DMSO controls.

**Table 5:** Biological activity of the most potent antiplasmodial thieno[2,3-*b*]pyridine-2-carboxamides.

Compound	IC_50_ (3D7) [nM]	HEK293T Viability at 30 µM [%] ^a^	SI ^b^
**3**	199.3	n.d.^c^	n.d.^c^
**9a**	410	123.0 ± 8.4	>70
**9e**	273	83.3 ± 20.6	>100
**9v**	1471	92.3 ± 18.8	>20
**9w**	860	76.1 ± 17.3	>30
**9y**	699	81.7 ± 19.6	>40
**17b**	192.5	89.0 ± 15.8	>100
**17c**	71.6	92.7 ± 11.7	>100
**17f**	19.2	72.4 ± 23.4	>100
**17g**	25.3	56.9 ± 31.3	>100
**17l**	55.5	68.4 ± 24.3	>100

^a^ calculated on DMSO controls. ^b^ SI = Selectivity index for 3D7 erythrocytic forms versus HEK293 cells; ^c^ n.d. = not determined.

## Supplementary Materials

The following are available online. Figure S1: Chemical structures of Test Compounds; Figures S2–S61: IR, ^1^H-NMR, ^13^C-NMR and APCI-MS data spectra of compounds **9a**, **9e**, **9j**, **9m**, **9n**, **9y**, **9z**, **9ac**, **17a**, **17b**, **17e**, **17f**, **17g** and **17h**; Figures S62–S79: HPLC chromatograms of **9a**, **9e**, **9m**, **9y**, **9ac**, **17a**, **17b**, **17f**, **17g**.

## References

[1] Snow R.W., Sartorius B., Kyalo D., Maina J., Amratia P., Mundia C.W., Bejon P., Noor A.M. (2017). The prevalence of Plasmodium falciparum in sub-saharan Africa since 1900. Nature.

[2] (2020). World Malaria Report 2019.

[3] Cibulskis R.E., Alonso P., Aponte J., Aregawi M., Barrette A., Bergeron L., Fergus C.A., Knox T., Lynch M., Patouillard E. (2016). Malaria: Global progress 2000–2015 and future challenges. Infect. Dis. Poverty.

[4] Bhatt S., Weiss D.J., Cameron E., Bisanzio D., Mappin B., Dalrymple U., Battle K., Moyes C.L., Henry A., Eckhoff P.A. (2015). The effect of malaria control on Plasmodium falciparum in Africa between 2000 and 2015. Nature.

[5] Dondorp A.M., Smithuis F.M., Woodrow C., Seidlein L.V. (2017). How to contain artemisinin and multidrug-resistant falciparum malaria. Trends Parasitol..

[6] (2006). WHO Briefing on Malaria Treatment Guidelines and Artemisinin Monotherapies.

[7] Vijaykadga S., Rojanawatsirivej C., Cholpol S., Phoungmanee D., Nakavej A., Wongsrichanalai C. (2006). In vivo sensitivity monitoring of mefloquine monotherapy and artesunate-mefloquine combinations for the treatment of uncomplicated falciparum malaria in Thailand in 2003. Trop. Med. Int. Health.

[8] Noedl H., Se Y., Schaecher K., Smith B.L., Socheat D., Fukuda M.M. (2008). Evidence of artemisinin-resistant malaria in western Cambodia. N. Engl. J. Med..

[9] Denis M.B., Tsuyuoka R., Lim P., Lindegardh N., Yi P., Top S.N., Socheat D., Fandeur T., Annerberg A., Christophel E.M. (2006). Efficacy of artemether-lumefantrine for the treatment of uncomplicated falciparum malaria in northwest Cambodia. Trop. Med. Int. Health.

[10] Haldar K., Bhattacharjee S., Safeukui I. (2018). Drug resistance in Plasmodium. Nat. Rev. Microbiol..

[11] Nsanzabana C. (2019). Resistance to artemisinin combination therapies (ACTs): Do not forget the partner drug!. Trop. Med. Infect. Dis..

[12] Pasupureddy R., Seshadri S., Pande V., Dixit R., Pandey K.C. (2019). Current scenario and future strategies to fight artemisinin resistance. Parasitol. Res..

[13] Daily J.P. (2017). Malaria 2017: Update on the clinical literature and management. Curr. Infect. Dis. Rep..

[14] Fugel W., Oberholzer A.E., Gschloessl B., Dzikowski R., Pressburger N., Preu L., Pearl L.H., Baratte B., Ratin M., Okun I. (2013). 3,6-Diamino-4-(2-halophenyl)-2-benzoylthieno[2,3-b]pyridine-5-carbonitriles are selective inhibitors of Plasmodium falciparum glycogen synthase kinase-3. J. Med. Chem..

[15] Solyakov L., Halbert J., Alam M.M., Semblat J.-P., Dorin-Semblat D., Reininger L., Bottrill A.R., Mistry S., Abdi A., Fennell C. (2011). Global kinomic and phospho-proteomic analyses of the human malaria parasite Plasmodium falciparum. Nat. Commun..

[16] Droucheau E., Primot A., Thomas V., Mattei D., Knockaert M., Richardson C., Sallicandro P., Alano P., Jafarshad A., Baratte B. (2004). Plasmodium falciparum glycogen synthase kinase-3: Molecular model, expression, intracellular localisation and selective inhibitors. BBA-Proteins Proteom..

[17] Zhang M., Wang C., Otto T.D., Oberstaller J., Liao X., Adapa S.R., Udenze K., Bronner I.F., Casandra D., Mayho M. (2018). Uncovering the essential genes of the human malaria parasite Plasmodium falciparum by saturation mutagenesis. Science.

[18] Masch A., Nasereddin A., Alder A., Bird M.J., Schweda S.I., Preu L., Doerig C., Dzikowski R., Gilberger T.W., Kunick C. (2019). Structure–activity relationships in a series of antiplasmodial thieno[2,3-b]pyridines. Malar. J..

[19] Sharanin Y.A., Shestopalov A.M., Litvinov V.P., Klokel G.V., Mortikov V.Y., Demerkov A.S. (1988). Cyclization of nitriles. XXV. Synthesis and reactions of chalkogen-containing 6-amino-3,5-dicyanopyridines. Russ. J. Org. Chem..

[20] Brandt W. (2009). Neue Inhibitoren der Proteinkinasen PfGSK-3 und RET. Dissertation Thesis.

[21] Masch A. (2017). Neue Thieno[2,3-b]pyridine als Hemmstoffe des Malaria-Erregers Plasmodium falciparum. Dissertation Thesis.

[22] Dyachenko I.V., Dyachenko V.D., Dorovatovskii P.V., Khrustalev V.N., Nenaydenko V.G. (2018). Synthesis of 2-alkylsulfanyl-6-amino-4-aryl-5-cyanonicotinonitriles by recyclization of 2,6-diamino-4-aryl-3,5-dicyano-4H-thiopyrans with alkyl halides. Russ. J. Org. Chem..

[23] Litvinov V.P., Dotsenko V.V., Krivokolysko S.G. (2005). Thienopyridines: Synthesis, properties and biological activity. Russ. Chem. Bull..

[24] Litvinov V.P., Dotsenko V.V., Krivokolysko S.G. (2007). The chemistry of thienopyridines. Adv. Heterocycl. Chem..

[25] Yongpruksa N., Pandey S., Baker G.A., Harmata M. (2011). Benzothiazines in organic synthesis. Synthesis of fluorescent 7-amino-2,1-benzothiazines. Org. Biomol. Chem..

[26] Cho S.D., Park Y.D., Kim J.J., Lee S.G., Ma C., Song S.Y., Joo W.H., Falck J.R., Shiro M., Shin D.S. (2003). A one-pot synthesis of pyrido[2,3-b][1,4]oxazin-2-ones. J. Org. Chem..

[27] Brunskill J.S.A., De A., Ewing D.F. (1978). Dimerisation of 3-aryl-2-cyanothioacrylamides. A [2s + 4s] cyclo-addition to give substituted 3,4-dihydro-2H-thiopyrans. J. Chem. Soc. Perk. Trans. 1.

[28] Elnagdi M.H., Abdelrazek F.M., Ibrahim N.S., Erian A.W. (1989). Studies on alkylheteroaromatic compounds. The reactivity of alkyl polyfunctionally substituted azines towards electrophilic reagents. Tetrahedron.

[29] Armarego W.L.F., Chai C.L.L. (2009). Purification of Laboratory Chemicals.

[30] Khidre M.D., Yakout E.S.M.A., Mahran M.R.H. (1998). Organophosphorus chemistry, 29. The action of 2,4-bis-(4-methoxy-phenyl)-1,3,2,4-dithiaphosphetane-2,4-disulfide (Lawesson′s reagent) on α, β-unsaturated nitriles. Phosphorus Sulfur Silicon Relat. Elem..

[31] McCall M.A. (1962). Reactions of substituted methylenemalononitriles and their derivatives 1. J. Org. Chem..

[32] Dyachenko V.D., Dyachenko A.D. (2007). Cross-recyclization of 4-aryl-2,6-diamino-4H-thiopyran-3,5-dicarbonitriles with 1-morpholino-1-cyclopentene: New route to 4-aryl-2-thioxo-2,5,6,7-tetrahydro-1H-[1]pyrindine-3-carbonitriles and their derivatives. Russ. J. Org. Chem..

[33] Sharanin Y.A., Promonenkov V.K., Shestopalov A.M. (1982). Recycyclization of 4-amino-6-aryl-5-cyano-1,3-dithia-4-cyclohexene-2-spirocycloalkanes to 4-aryl-3-cyano-5,6-polymethylenepyridine-2(1H)-thiones. Russ. J. Org. Chem..

[34] Sharanin Y.A., Shestopalov A.M., Promonenkov V.K., Rodinovskaya L.A. (1984). Cyclization of nitriles. X. Enaminonitriles of the 1,3-dithia-4-cyclohexene series and their recyclization into pyridine and thiazole derivatives. Russ. J. Org. Chem..

[35] Sharanin Y.A., Shestopalov A.M. (1989). Cyclization of nitriles. XXXIV. Transformation of 4-aryl-2,6-diamino-3,5-dicyano-4H-thiopyrans into substituted 4-aryl-3-cyano-2(1H)-pyridinethiones and 2-amino-4-aryl-7,7-dimethyl-5-oxo-3-cyano-5,6,7,8-tetrahydro-4H-benzo[b]pyrans. Russ. J. Org. Chem..

[36] Dyachenko V.D., Dyachenko A.D. (2008). Synthesis of 4-alkyl(aryl, hetaryl)-2-thioxo-5,6,7,8-tetrahydroquinoline-3-carbonitriles and their derivatives by cross-recyclization of 4-alkyl(aryl, hetaryl)-2,6-diamino-4H-thiopyran-3,5-dicarbonitriles with 4-(cyclohex-1-en-1-yl)-morpholine, alkyl halides and cyclohexanone. Russ. J. Org. Chem..

[37] Vieweg H., Leistner S., Wagner G. (1988). Synthese von 4-Aryl-3-cyan-5,6,7,8-tetrahydro-chinolin-2(1H)-thionen sowie von 3-Cyan-4-phenyl-6,7,8,9-tetrahydro-5H-cyclohepta-pyrid-2(1H)-thion und davon abgeleitete Thieno-Derivate. Pharmazie.

[38] Hauser M. (1962). The reaction of vinyl chloroacetate with some nucleophilic reagents. J. Org. Chem..

[39] Gaillot J.M., Gelas-Mialhe Y., Vessiere R. (1979). Synthesis and reactivity of 2-sulfonyl-2-haloaziridines. Can. J. Chem..

[40] Biel J.H., Warawa E.J. (1968). N-Aryl-N′-Cyclopropyl-Ethylene Diamine Derivatives. U.S. Patent.

[41] Abdel-rahman A.E., Bakhite E.A., Mohamed O.S., Thabet E.A. (2000). Synthesis of some new thieno[2,3-b]pyridines, pyrido[3′,2′:4,5]-thieno[3,2-d]pyrimidines and pyrido[3′,2′:4,5]thieno[3,2-d][1,2,3]-triazines. Phosphorus Sulfur Silicon Relat. Elem..

[42] Berrow N.S., Alderton D., Sainsbury S., Nettleship J., Assenberg R., Rahman N., Stuart D.I., Owens R.J. (2007). A versatile ligation-independent cloning method suitable for high-throughput expression screening applications. Nucleic Acids Res..

[43] Smilkstein M., Sriwilaijaroen N., Kelly J.X., Wilairat P., Riscoe M. (2004). Simple and inexpensive fluorescence-based technique for high-throughput antimalarial drug screening. Antimicrob. Agents Chemother..

[44] Malleret B., Claser C., Ong A.S.M., Suwanarusk R., Sriprawat K., Howland S.W., Russell B., Nosten F., Rénia L. (2011). A rapid and robust tri-color flow cytometry assay for monitoring malaria parasite development. Sci. Rep..

[45] Trager W., Jensen J.B. (1976). Human malaria parasites in continuous culture. Science.

