# Supplementary material for: 4-Arylthieno[2,3-b]pyridine-2-carboxamides Are a New Class of Antiplasmodial Agents
Source: Molecules. 2020 Jul 13;25(14):3187. doi: 10.3390/molecules25143187 (PMC7397174; doi:10.3390/molecules25143187)
Supplement: Supplementary file 1 [file molecules-25-03187-s001.pdf]

Supporting Information

4-Arylthieno[2,3-*b*]pyridine-2-carboxamides are a new class of antiplasmodial agents

Sandra I. Schweda [1,2], Arne Alder [3,4,5], Tim Gilberger [3,4,5] and Conrad Kunick [1, 2,*]

1 Institut für Medizinische und Pharmazeutische Chemie, Technische Universität Braunschweig, Beethovenstraße 55, 38106 Braunschweig, Germany
2 Zentrum für Pharmaverfahrenstechnik (PVZ), Technische Universität Braunschweig, Franz-Liszt-Straße 35A, 38106 Braunschweig, Germany
3 Centre for Structural Systems Biology, 22607 Hamburg, Germany
4 Bernhard Nocht Institute for Tropical Medicine, 20359 Hamburg, Germany
5 University of Hamburg, 20146 Hamburg, Germany
* Correspondence: c.kunick@tu-braunschweig.de

Table of contents

Figure S1: Structures and names of compounds **9a-9af** and **17a-17l** 1
Figure S2-S5: Spectral data of **9a** 9
Figure S6-S9: Spectral data of **9e** 13
Figure S10-S13: Spectral data of **9j** 17
Figure S14-S18: Spectral data of **9m** 21
Figure S19-S22: Spectral data of **9n** 26
Figure S23-S26: Spectral data of **9y** 30
Figure S27-S31: Spectral data of **9z** 34
Figure S32-S35: Spectral data of **9ac** 39
Figure S36-S40: Spectral data of **17a** 43
Figure S41-S45: Spectral data of **17b** 48
Figure S46-S49: Spectral data of **17e** 53
Figure S50-S53: Spectral data of **17f** 57
Figure S54-S57: Spectral data of **17g** 61
Figure S58-S61: Spectral data of **17h** 65
Figure S62-S79: HPLC chromatograms of **9a**, **9e**, **9m**, **9y**, **9ac**, **17a**, **17b**, **17f** and **17g** 69

Figure S1:

3,6-Diamino-4-(3-chlorophenyl)-*N*-(4-chlorophenyl)-5-cyanothieno[2,3-*b*]pyridine-2-carboxamide **9a** (KuSaSch018)

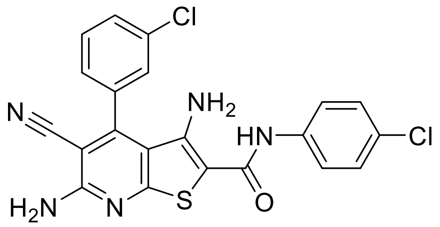

3,6-Diamino-*N*-(2-chlorophenyl)-5-cyano-4-(3-fluorophenyl)thieno[2,3-*b*]pyridine-2-carboxamide **9b** (KuSaSch022)

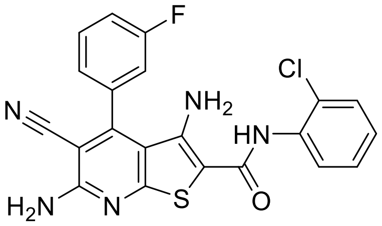

3,6-Diamino-*N*-(2-chlorophenyl)-4-(3-chlorophenyl)-5-cyanothieno[2,3-*b*]pyridine-2-carboxamide **9c** (KuSaSch027)

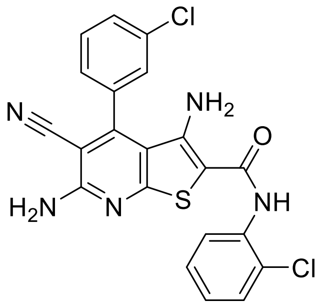

3,6-Diamino-4-(3-chlorophenyl)-5-cyano-*N*-(4-fluorophenyl)thieno[2,3-*b*]pyridine-2-carboxamide **9d** (KuSaSch028)

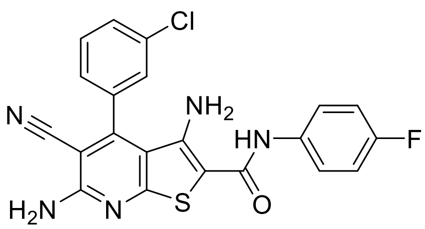

3,6-Diamino-*N*-(4-chlorophenyl)-5-cyano-4-(3-methylphenyl)thieno[2,3-*b*]pyridine-2-carboxamide **9e** (KuSaSch031)

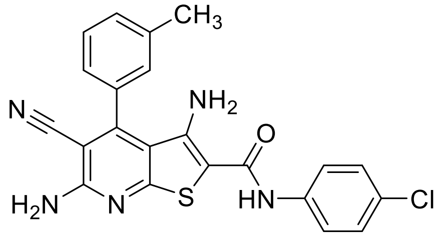

3,6-Diamino-*N*-(2-chlorophenyl)-5-cyano-4-(3-methylphenyl)thieno[2,3-*b*]pyridine-2-carboxamide **9f** (KuSaSch032)

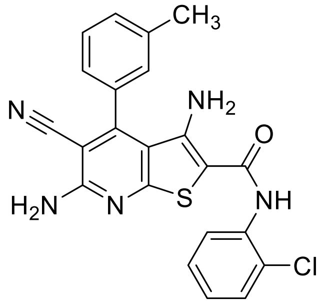

3,6-Diamino-5-cyano-*N*-(4-fluorophenyl)-4-(3-methylphenyl)thieno[2,3-*b*]pyridine-2-carboxamide **9g** (KuSaSch033)

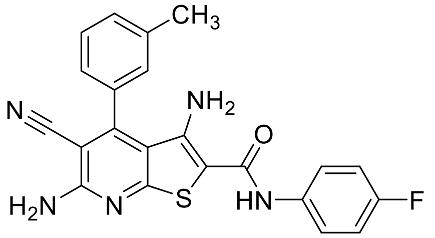

3,6-Diamino-*N*-(4-chlorophenyl)-5-cyano-4-(3-fluorophenyl)thieno[2,3-*b*]pyridine-2-carboxamide **9h** (KuSaSch037)

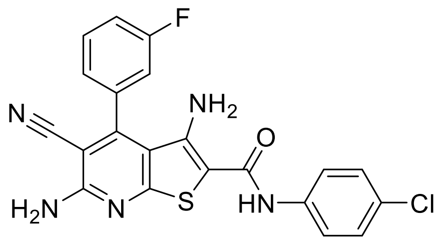

3,6-Diamino-5-cyano-4-(3-fluorophenyl)-*N*-(4-fluorophenyl)thieno[2,3-*b*]pyridine-2-carboxamide **9i** (KuSaSch038)

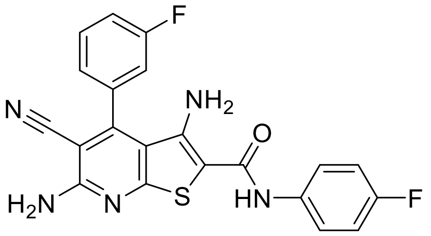

3,6-Diamino-5-cyano-*N*-methyl-4-(3-methylphenyl)thieno[2,3-*b*]pyridine-2-carboxamide **9j** (KuSaSch127)

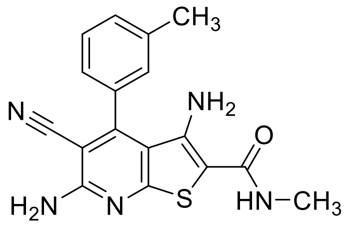

3,6-Diamino-5-cyano-*N*-heptyl-4-(3-methylphenyl)thieno[2,3-*b*]pyridine-2-carboxamide **9k** (KuSaSch129)

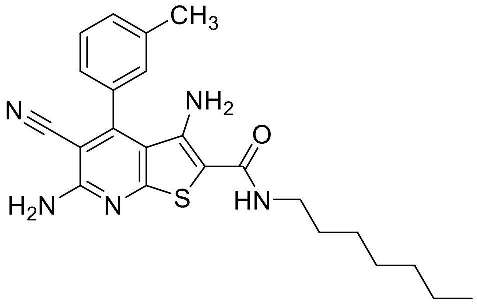

3,6-Diamino-5-cyano-*N*-isopropyl-4-(3-methylphenyl)thieno[2,3-*b*]pyridine-2-carboxamide **9l** (KuSaSch131)

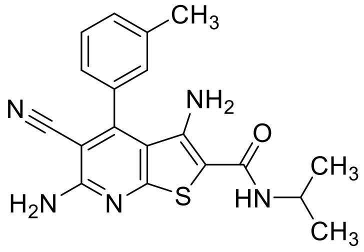

3,6-Diamino-5-cyano-*N*-cyclopropyl-4-(3-methylphenyl)thieno[2,3-*b*]pyridine-2-carboxamide **9m** (KuSaSch134)

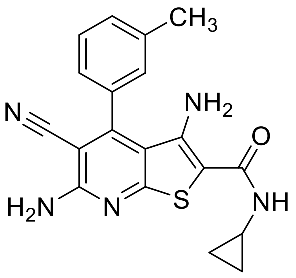

3,6-Diamino-5-cyano-*N*-(2-morpholinoethyl)-4-(3-methylphenyl)thieno[2,3-*b*]pyridine-2-carboxamide **9n** (KuSaSch135)

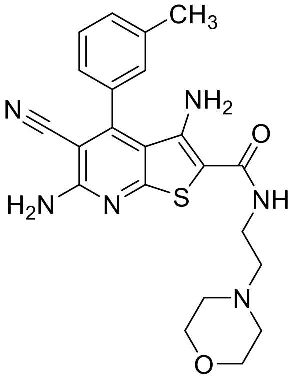

3,6-Diamino-5-cyano-*N*-(2-cyclopropylethyl)-4-(3-methylphenyl)thieno[2,3-*b*]pyridine-2-carboxamide **9o** (KuSaSch137)

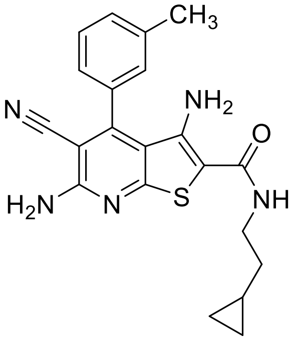

tert-Butyl 4-(3-chloro-4-{3,6-diamino-2-[(3-chlorophenyl)carbamoyl]-5-cyanothieno[2,3-*b*]pyridin-4-yl}phenyl)piperazine-1-carboxylate **9p** (KuSaSch041)

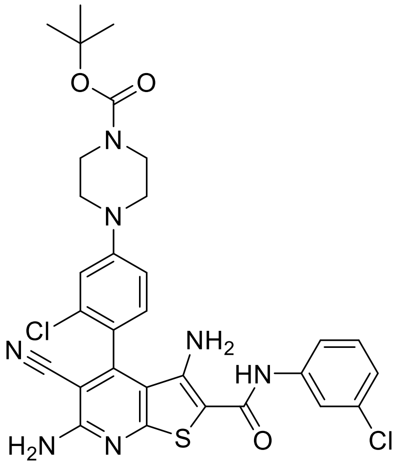

3,6-Diamino-4-[2-chloro-4-(piperazin-1-yl)phenyl]-*N*-(3-chlorophenyl)-5-cyanothieno[2,3-*b*]-pyridine-2-carboxamide hydrochloride **9q** (KuSaSch043)

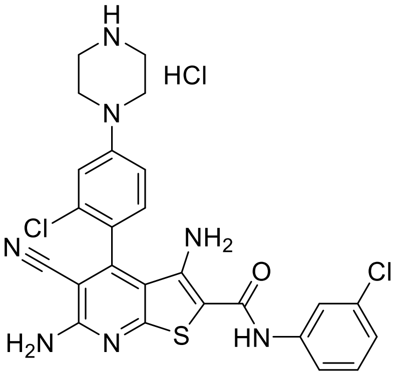

3,6-Diamino-4-(2-chloro-4-morpholinophenyl)-*N*-(3-chlorophenyl)-5-cyanothieno[2,3-*b*]pyridine-2-carboxamide **9r** (KuSaSch050)

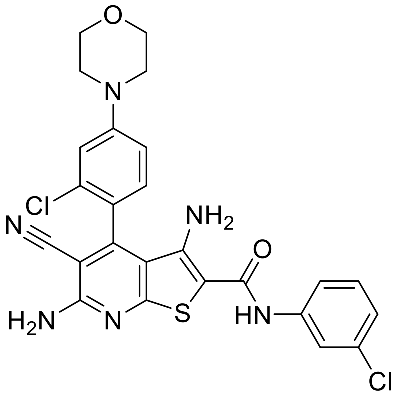

3,6-Diamino-4-[2-chloro-4-(pyrrolidin-1-yl)phenyl]-*N*-(3-chlorophenyl)-5-cyanothieno[2,3-*b*]-pyridine-2-carboxamide **9s** (KuSaSch051**)**

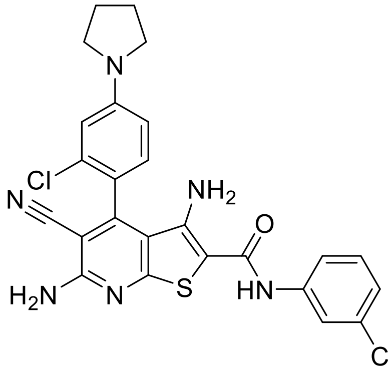

3,6-Diamino-4-[2-chloro-4-(pyrrolidin-1-yl)phenyl]-*N*-(2-chlorophenyl)-5-cyanothieno[2,3-*b*]-pyridine-2-carboxamid **9t** (KuSaSch055)

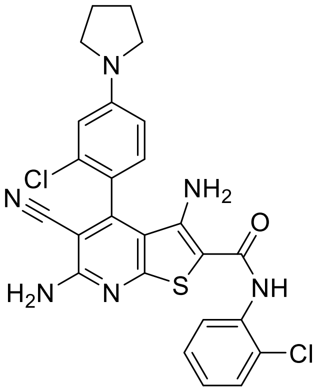

3,6-Diamino-4-(2-chloro-4-morpholinophenyl)-*N*-(2-chlorophenyl)-5-cyanothieno[2,3-*b*]pyridine-2-carboxamide **9u** (KuSaSch056)

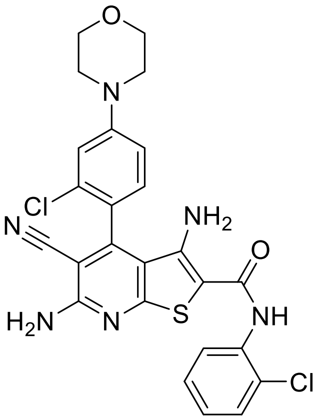

3,6-Diamino-4-(2-chloro-4-morpholinophenyl)-*N*-(4-chlorophenyl)-5-cyanothieno[2,3-*b*]pyridine-2-carboxamide **9v** (KuSaSch057)

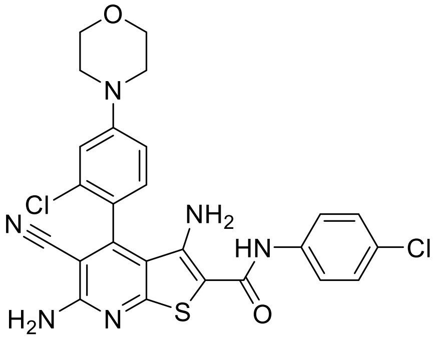

tert-Butyl {2-[(3-chloro-4-{3,6-diamino-2-[(3-chlorophenyl)carbamoyl]-5-cyanothieno[2,3-*b*]pyridin-4-yl}phenyl)(methyl)amino]ethyl}carbamate **9w** (KuSaSch058)

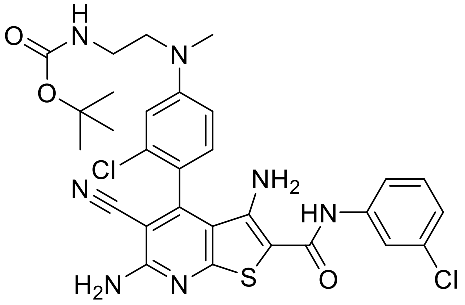

3,6-Diamino-4-(2-chloro-4-morpholinophenyl)-5-cyano-*N*-(4-fluorophenyl)thieno[2,3-*b*]pyridine-2-carboxamide **9x** (KuSaSch059)

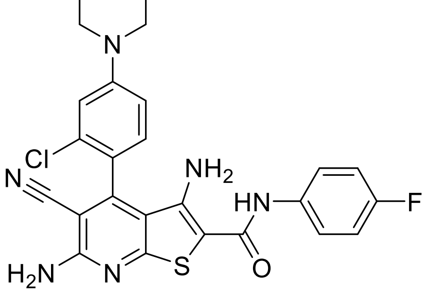

tert-Butyl {2-[(3-chloro-4-{3,6-diamino-2-[(4-chlorophenyl)carbamoyl]-5-cyanothieno[2,3-*b*]pyridin-4-yl}phenyl)(methyl)amino]ethyl}carbamate **9y** (KuSaSch060)

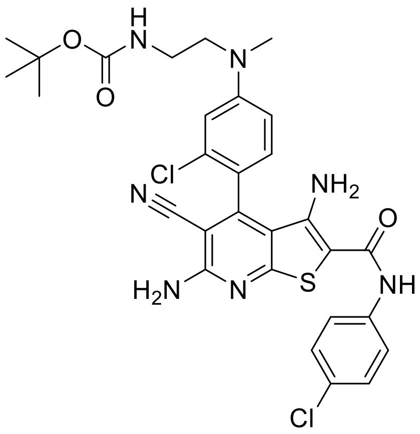

3,6-Diamino-4-[2-chloro-4-(pyrrolidin-1-yl)phenyl]-*N*-(4-chlorophenyl)-5-cyanothieno[2,3-*b*]-pyridine-2-carboxamide **9z** (KuSaSch063)

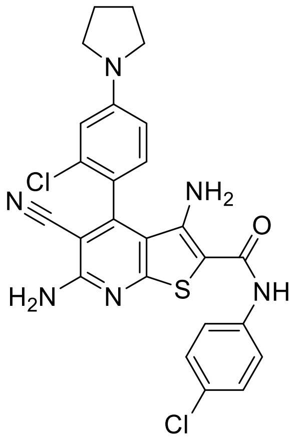

3,6-Diamino-4-[2-chloro-4-(pyrrolidin-1-yl)phenyl]-5-cyano-*N*-(4-fluorophenyl)thieno[2,3-*b*]-pyridine-2-carboxamide **9aa** (KuSaSch064)

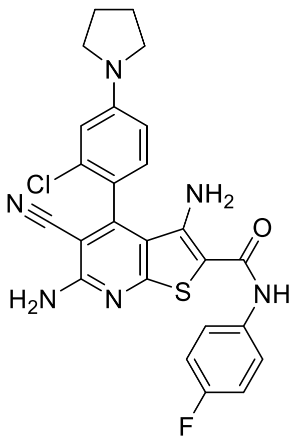

3,6-Diamino-4-(2-chloro-4-{[2-(dimethylamino)ethyl](methyl)amino}phenyl)-5-cyano-*N*-(4-fluoro-phenyl)thieno[2,3-*b*]pyridine-2-carboxamide **9ab** (KuSaSch067)

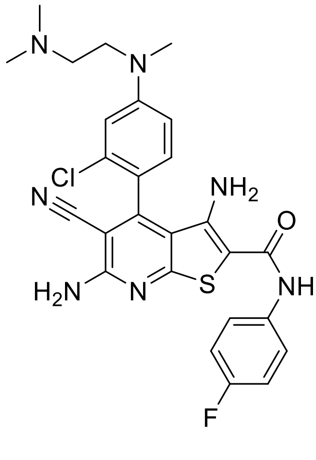

tert-Butyl {2-[(3-chloro-4-{3,6-diamino-5-cyano-2-[(4-fluorophenyl)carbamoyl]thieno[2,3-*b*]pyridin-4-yl}phenyl)(methyl)amino]ethyl}carbamate **9ac** (KuSaSch073)

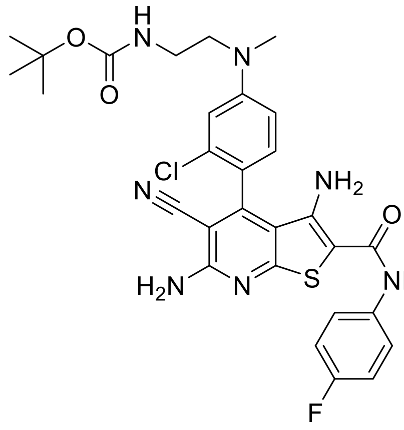

tert-Butyl {2-[(3-chloro-4-{3,6-diamino-2-[(2-chlorophenyl)carbamoyl]-5-cyanothieno[2,3-*b*]pyridin-4-yl}phenyl)(methyl)amino]ethyl}carbamate **9ad** (KuSaSch074)

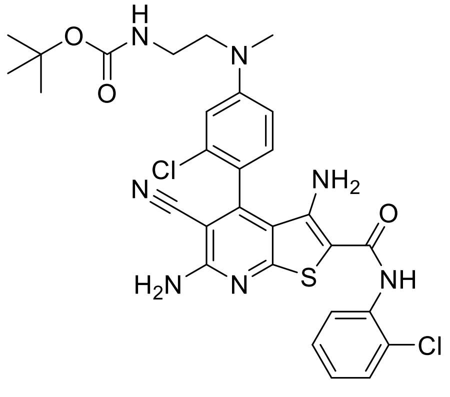

3,6-Diamino-4-{4-[(2-aminoethyl)(methyl)amino]-2-chlorophenyl}-*N*-(3-chlorophenyl)-5-cyano-thieno-[2,3-*b*]pyridine-2-carboxamide hydrochloride **9ae** (KuSaSch075)

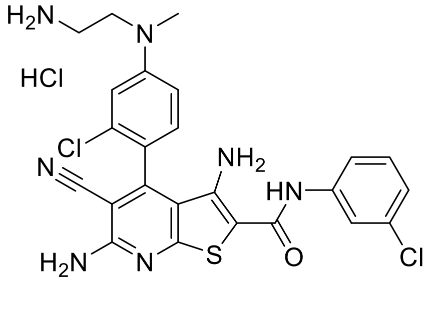

3,6-Diamino-4-(2-chloro-4-{[2-(dimethylamino)ethyl](methyl)amino}phenyl)-*N*-(3-chlorophenyl)-5-cyanothieno[2,3-*b*]pyridine-2-carboxamide **9af** (KuSaSch090)

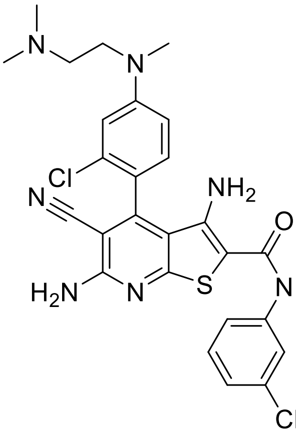

tert-Butyl 4-(4-{3-amino-2-[(4-chlorophenyl)carbamoyl]-6,7-dihydro-5*H*-cyclopenta[*b*]thieno[3,2-*e*]pyridin-4-yl}-3-chlorophenyl)piperazine-1-carboxylate **17a** (KuSaSch095)

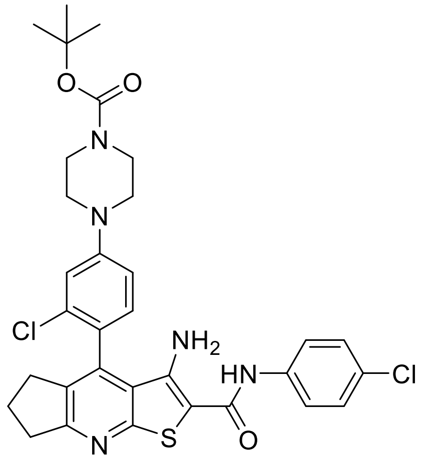

3-Amino-*N*-(4-chlorophenyl)-4-phenyl-6,7-dihydro-5*H*-cyclopenta[*b*]thieno[3,2-*e*]pyridine-2-carboxamide **17b** (KuSaSch100)

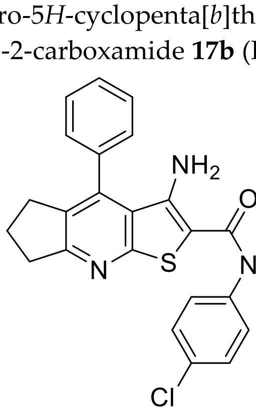

3-Amino-*N*-(4-fluorophenyl)-4-phenyl-6,7-dihydro-5*H*-cyclopenta[*b*]thieno[3,2-*e*]pyridine-2-carboxamide **17c** (KuSaSch101)

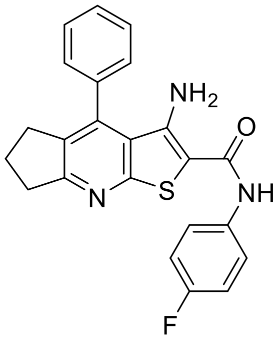

3-Amino-*N*-(4-chlorophenyl)-4-phenyl-5,6,7,8-tetrahydrothieno[2,3-*b*]quinoline-2-carboxamide **17d** (KuSaSch105)

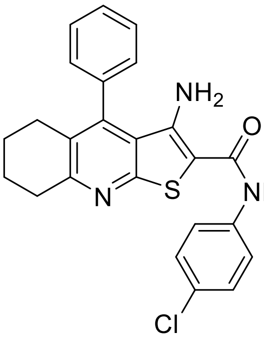

3-Amino-*N*-(4-chlorophenyl)-4-(3-methylphenyl)-5,6,7,8-tetrahydrothieno[2,3-*b*]quinoline-2-carboxamide **17e** (KuSaSch107)

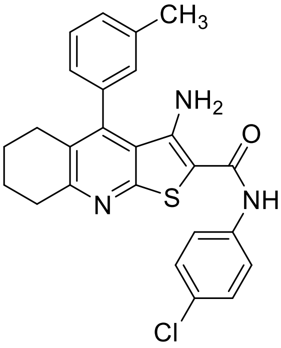

3-Amino-*N*-(4-chlorophenyl)-4-(3-methylphenyl)-6,7-dihydro-5*H*-cyclopenta[*b*]thieno[3,2-*e*]-pyridine-2-carboxamide **17f** (KuSaSch110)

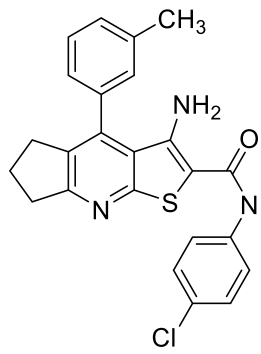

tert-Butyl 3-amino-2-[(4-chlorophenyl)carbamoyl]-6-methyl-4-phenylthieno[2,3-*b*]pyridine-5-carboxylate **17g** (KuSaSch111)

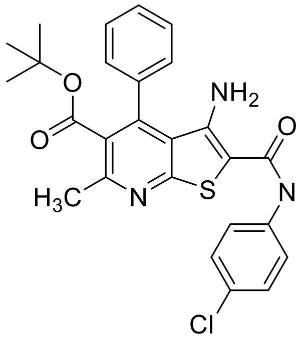

tert-Butyl 3-amino-2-[(4-chlorophenyl)carbamoyl]-6-methyl-4-(3-methylphenyl)thieno[2,3-*b*]-pyridine-5-carboxylate **17h** (KuSaSch112)

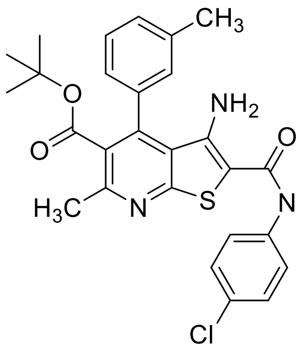

3-Amino-2-[(4-chlorophenyl)carbamoyl]-6-methyl-4-(3-methylphenyl)thieno[2,3-*b*]pyridine-5-carboxylic acid **17i** (KuSaSch114)

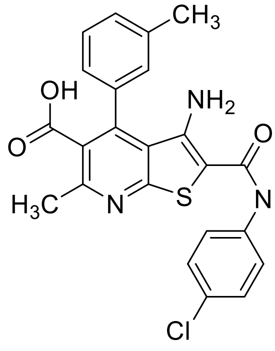

3-Amino-2-[(4-chlorophenyl)carbamoyl]-6-methyl-4-phenylthieno[2,3-*b*]pyridine-5-carboxylic acid **17j** (KuSaSch115)

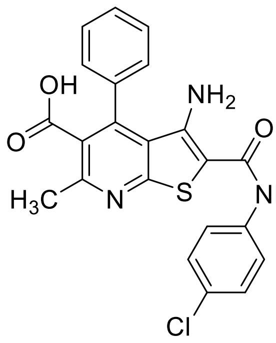

tert-Butyl 4-(4-{3-amino-2-[(4-chlorophenyl)carbamoyl]-5,6,7,8-tetrahydrothieno[2,3-*b*]quinolin-4-yl}-3-chlorophenyl)piperazine-1-carboxylate **17k** (KuSaSch118)

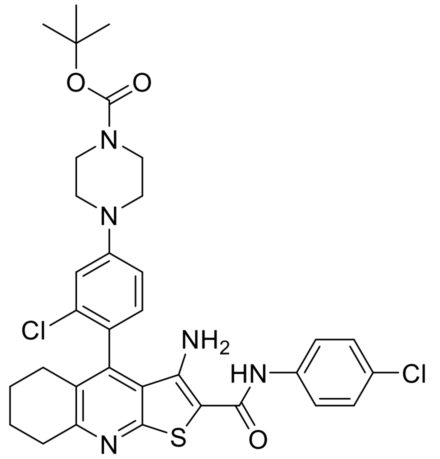

tert-Butyl 4-(4-{3-amino-2-[(4-chlorophenyl)carbamoyl]-6-methylthieno[2,3-*b*]pyridin-4-yl}-3-chlorophenyl)piperazine-1-carboxylate **17l** (KuSaSch122)

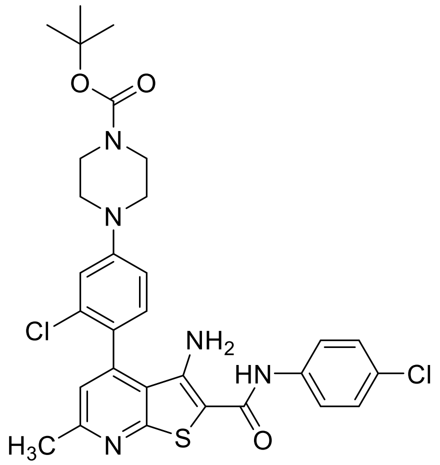

Figure S2: ^{1}H-NMR spectrum of **9a** (KuSaSch018).

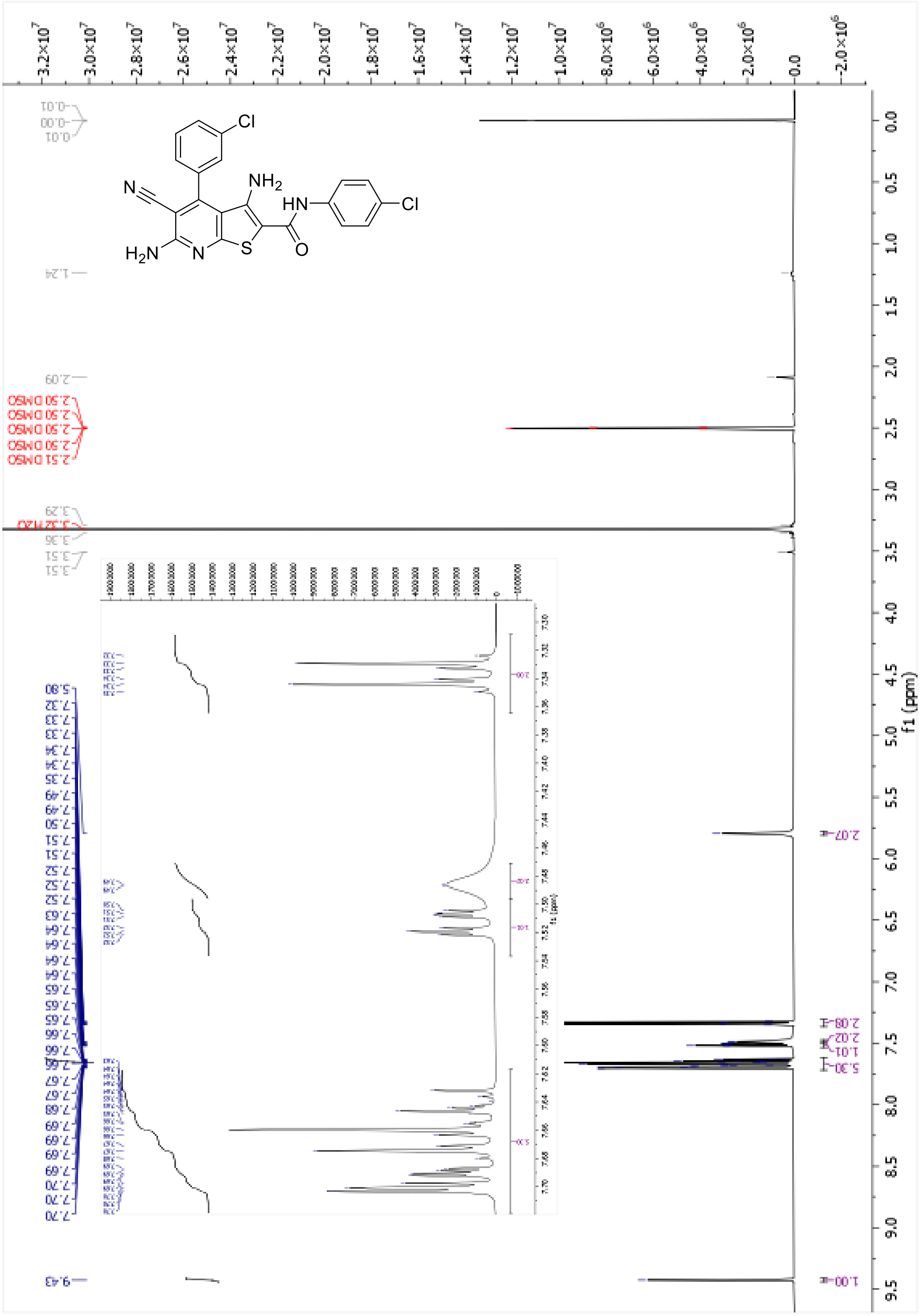

Figure S3: ^{13}C-NMR spectrum of **9a** (KuSaSch018).

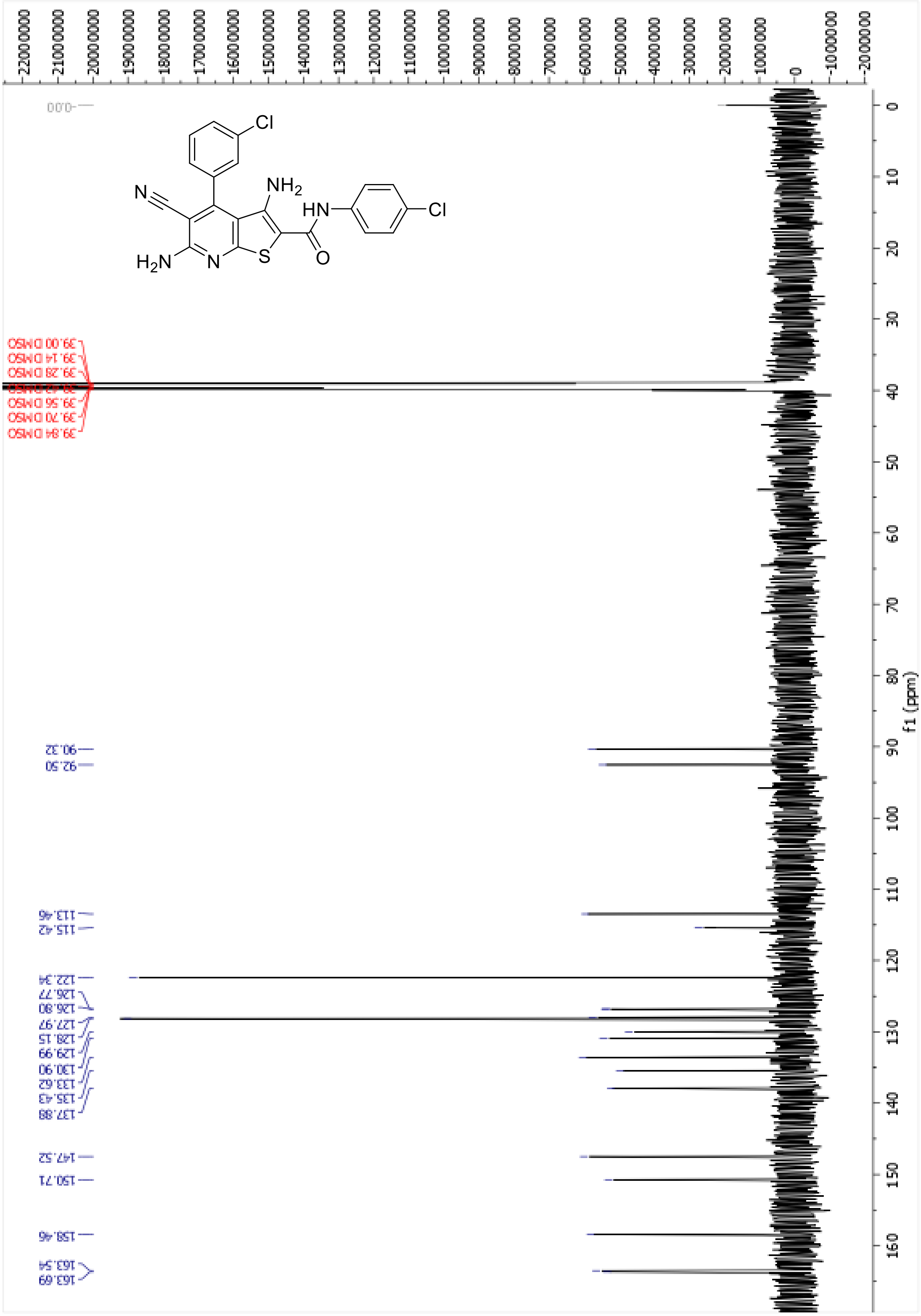

Figure S4: IR spectrum of **9a** (KuSaSch018).

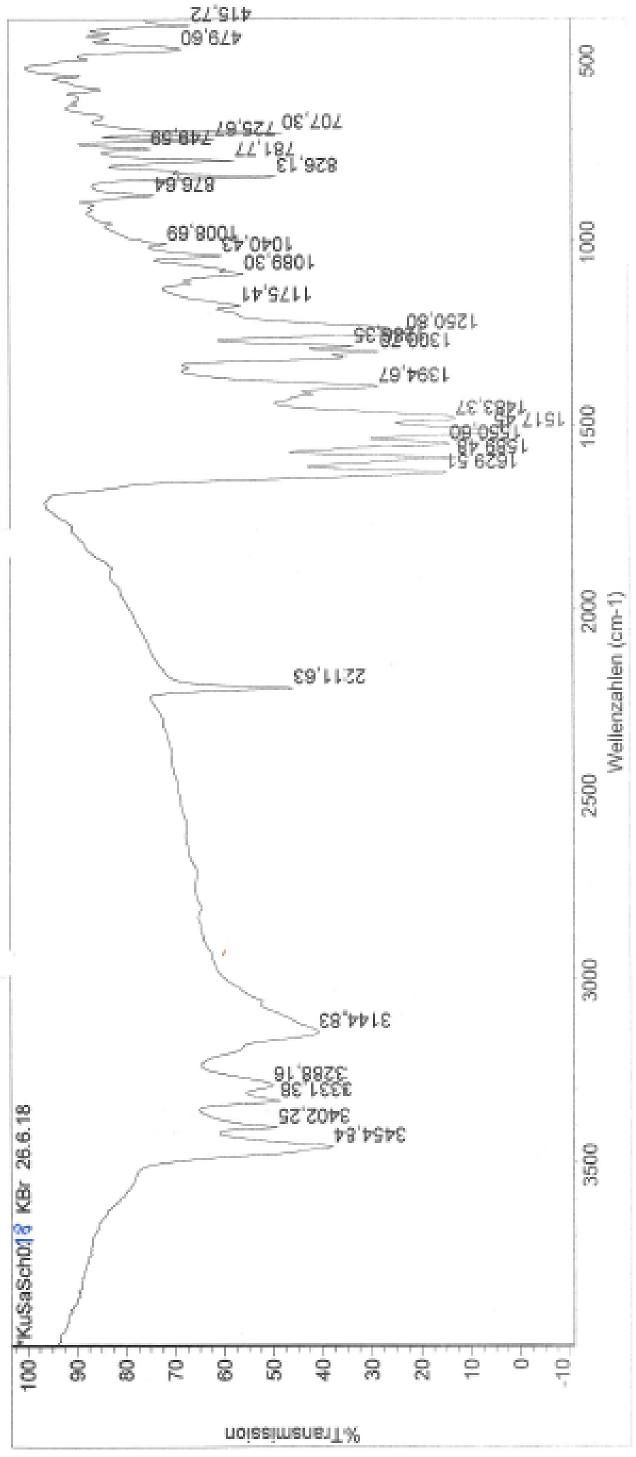

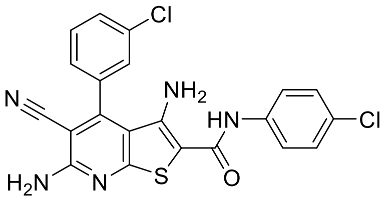

Tue Jun 26 08:29:56 2018 (GMT+02:00)
SUCHE BANDEN:
Spektrum: *KuSaSch025 KBr 26.6.18
Bereich: 3999,64 400,00
Absoluter Schwellwert: 75,549
Sensitivität: 50
Bandentabelle:

Position:	1517,45	Intensität:	4,961
Position:	1589,48	Intensität:	12,464
Position:	1483,37	Intensität:	12,752
Position:	1550,60	Intensität:	13,885
Position:	1629,51	Intensität:	14,589
Position:	1250,80	Intensität:	22,557
Position:	1394,67	Intensität:	28,263
Position:	1300,70	Intensität:	28,018
Position:	1286,35	Intensität:	33,230
Position:	3454,84	Intensität:	37,771

Figure S5: APCI-MS spectrum of **9a** (KuSaSch018).

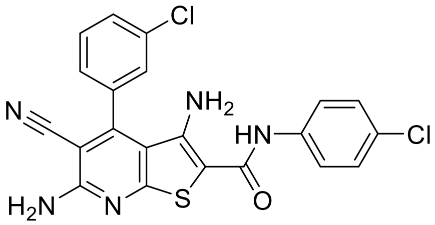

Exact Mass: 453,02179

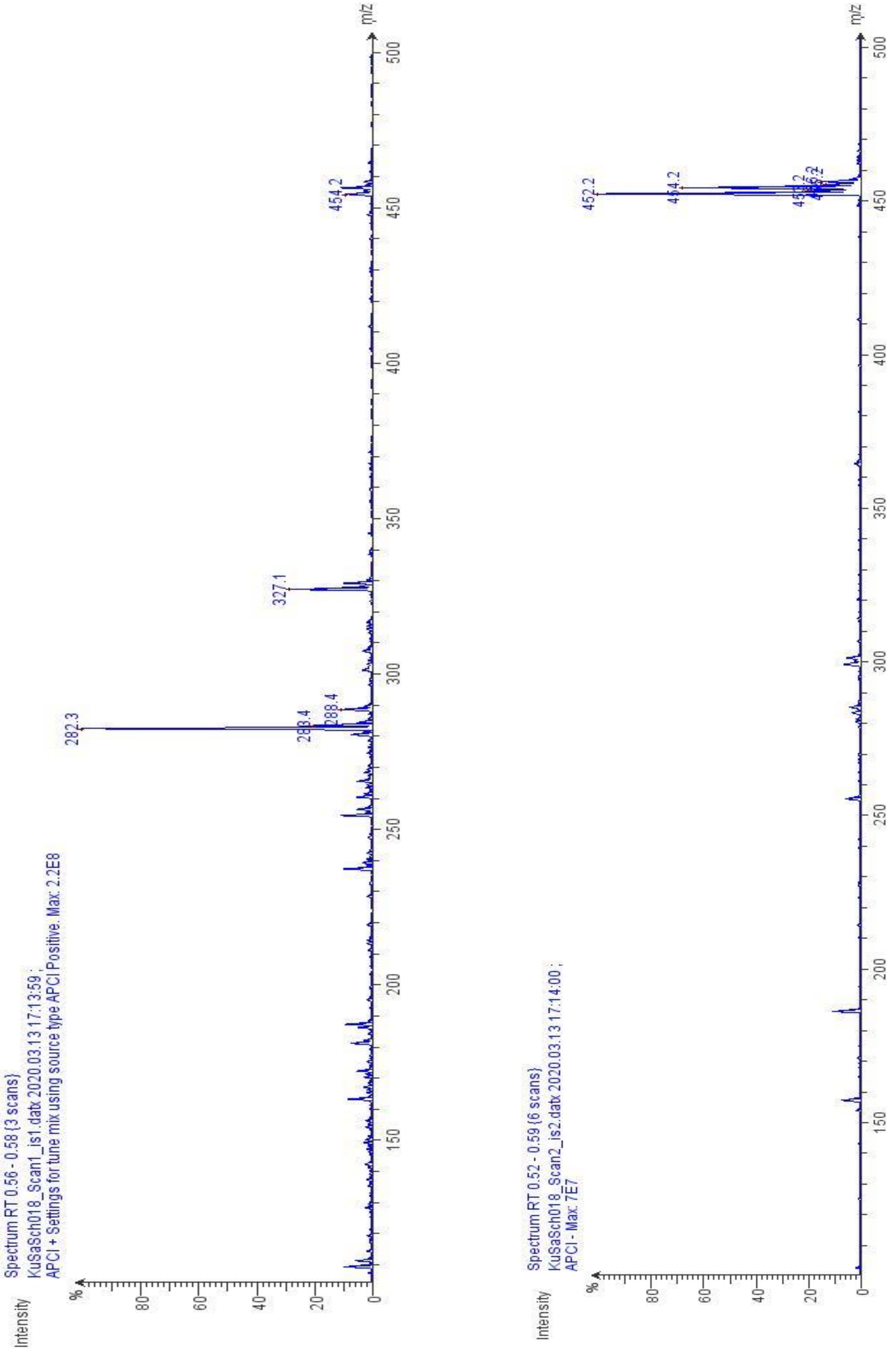

Figure S6: ^{1}H-NMR spectrum of **9e** (KuSaSch031).

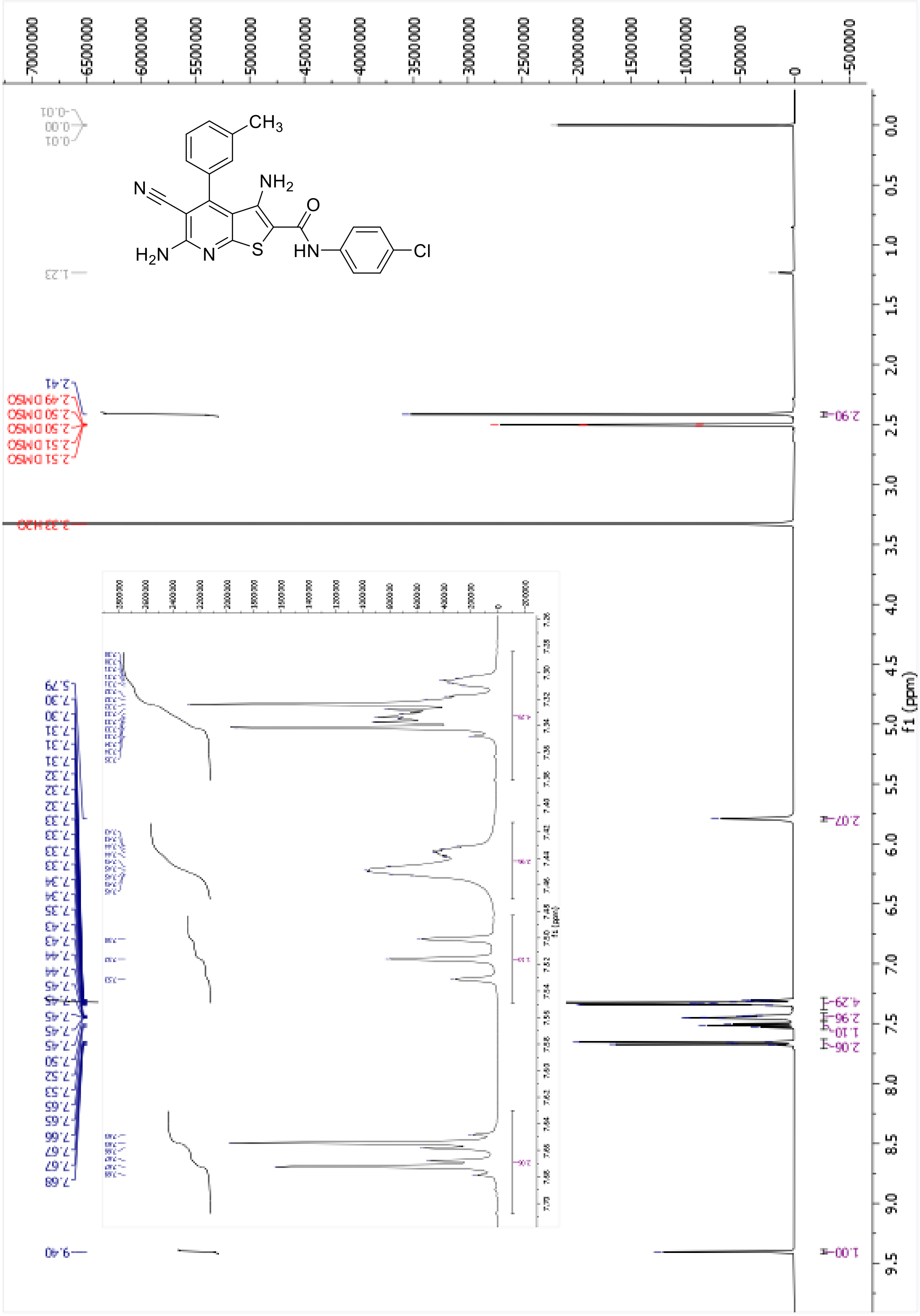

Figure S7: ^{13}C-NMR spectrum of **9e** (KuSaSch031).

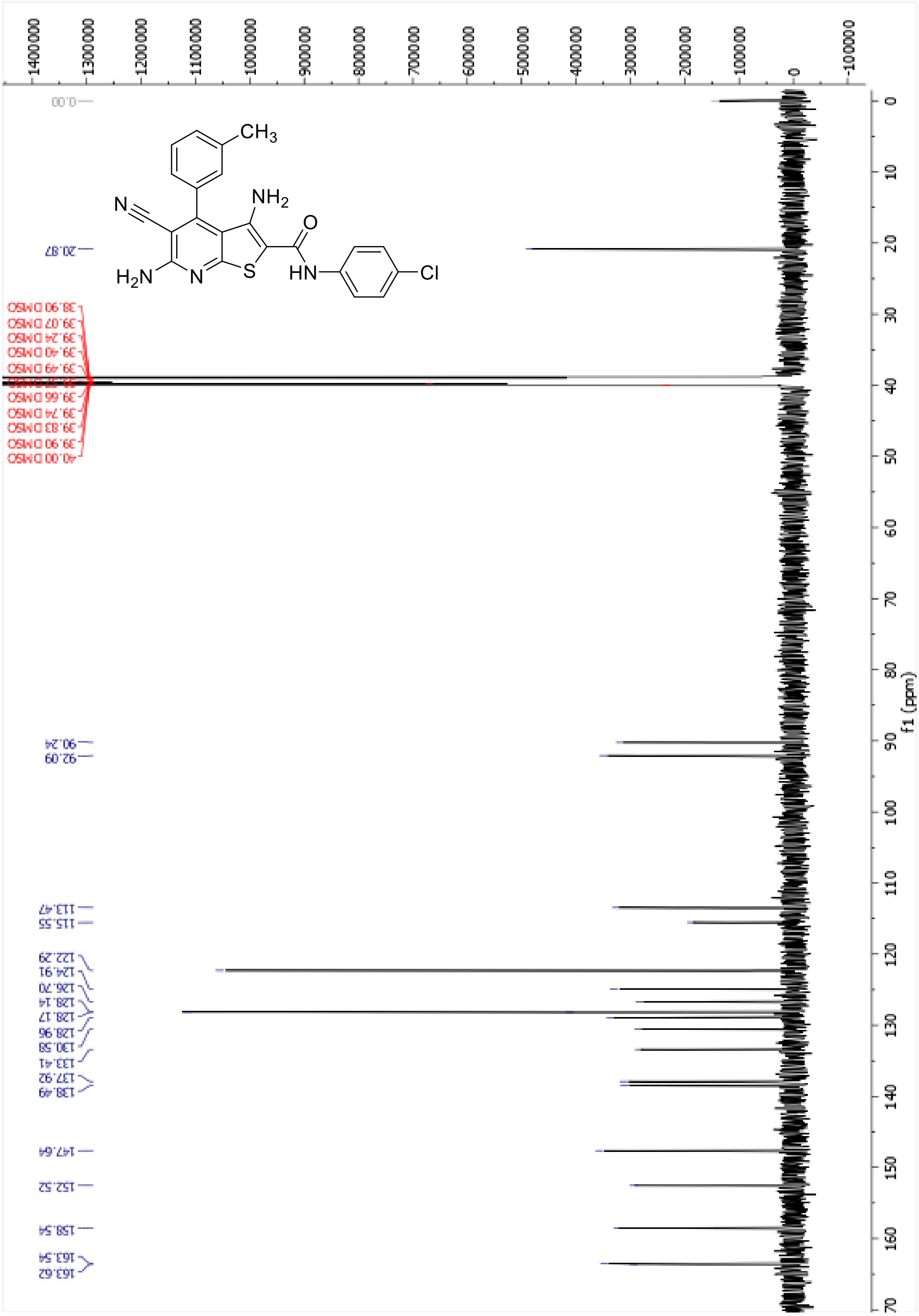

Figure S8: IR spectrum of **9e** (KuSaSch031).

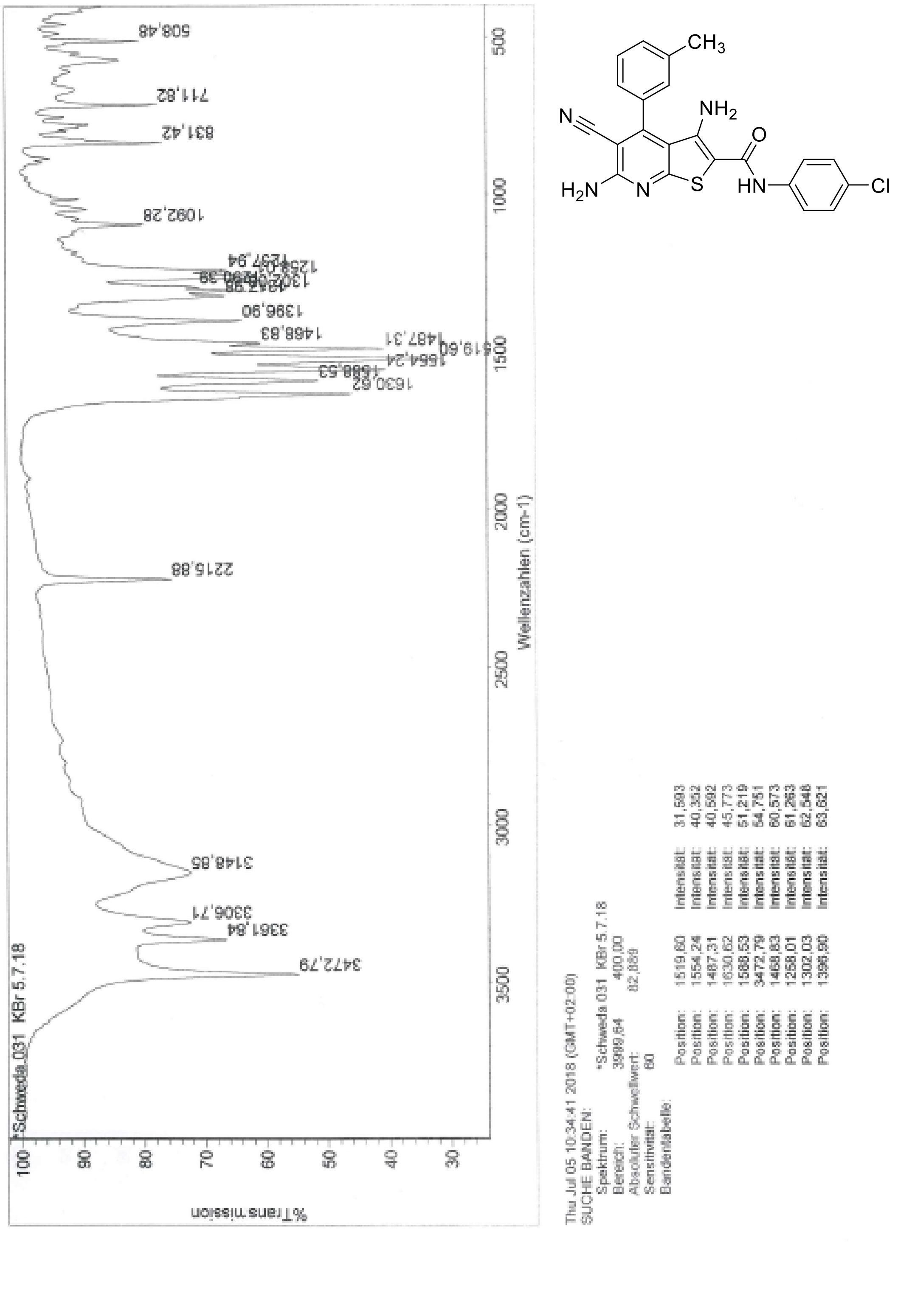

Figure S9: APCI-MS spectrum of **9e** (KuSaSch031).

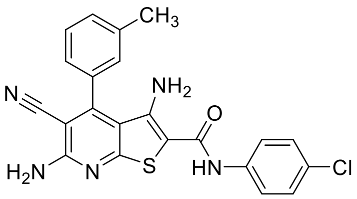

Exact Mass: 433,07641

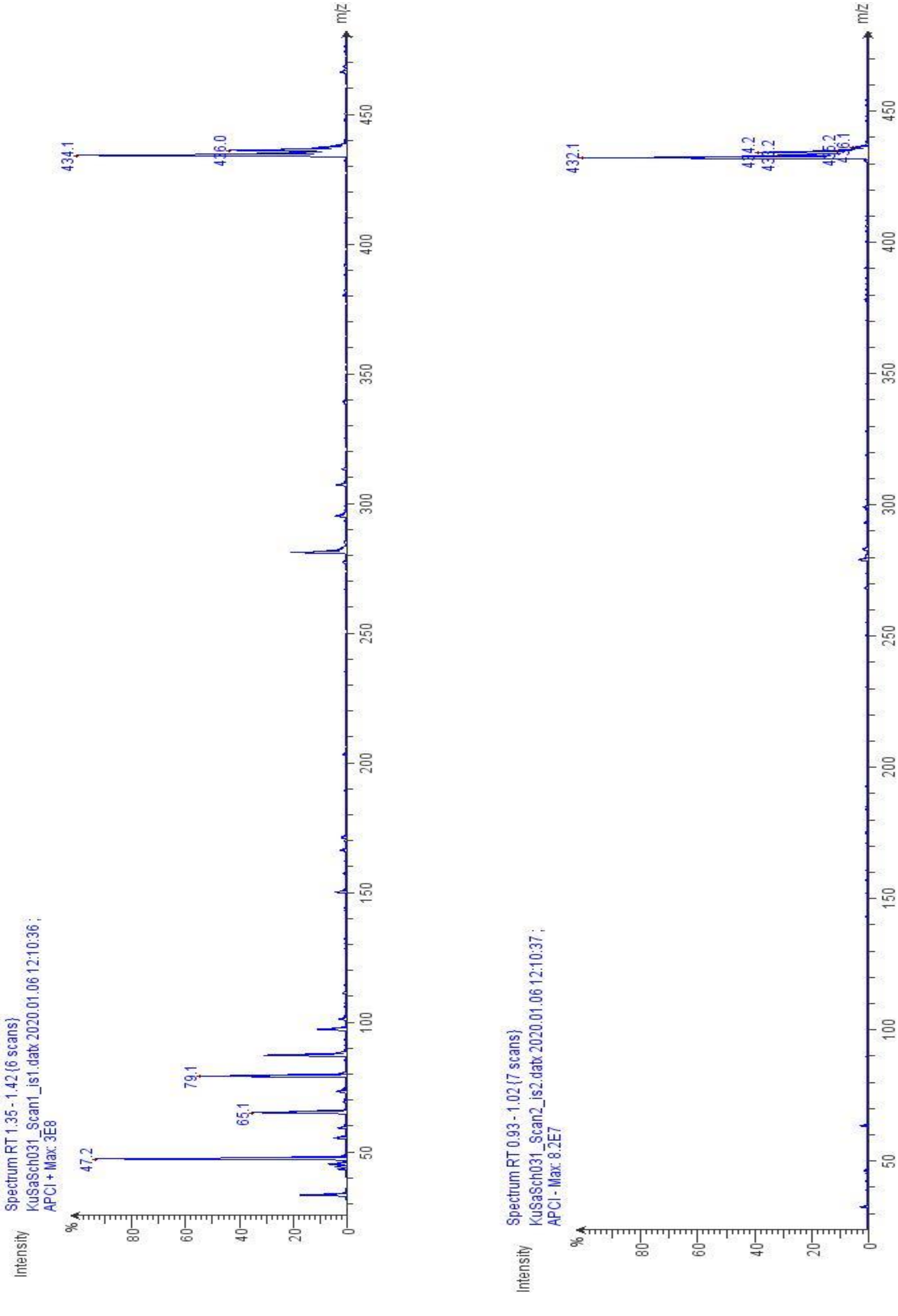

Figure S10: ^{1}H-NMR spectrum of **9j** (KuSaSch127).

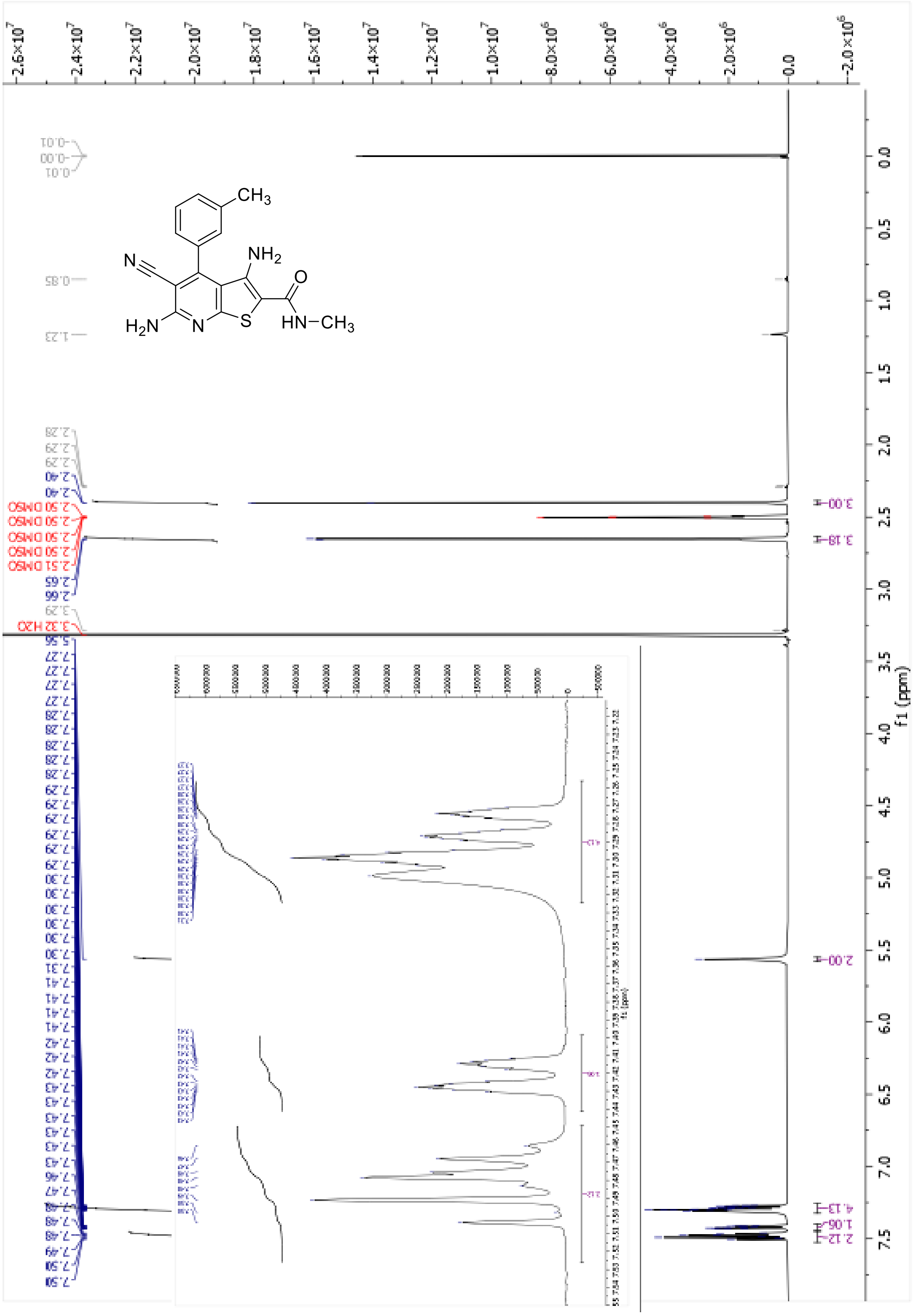

Figure S11: ^{13}C-NMR spectrum of **9j** (KuSaSch127).

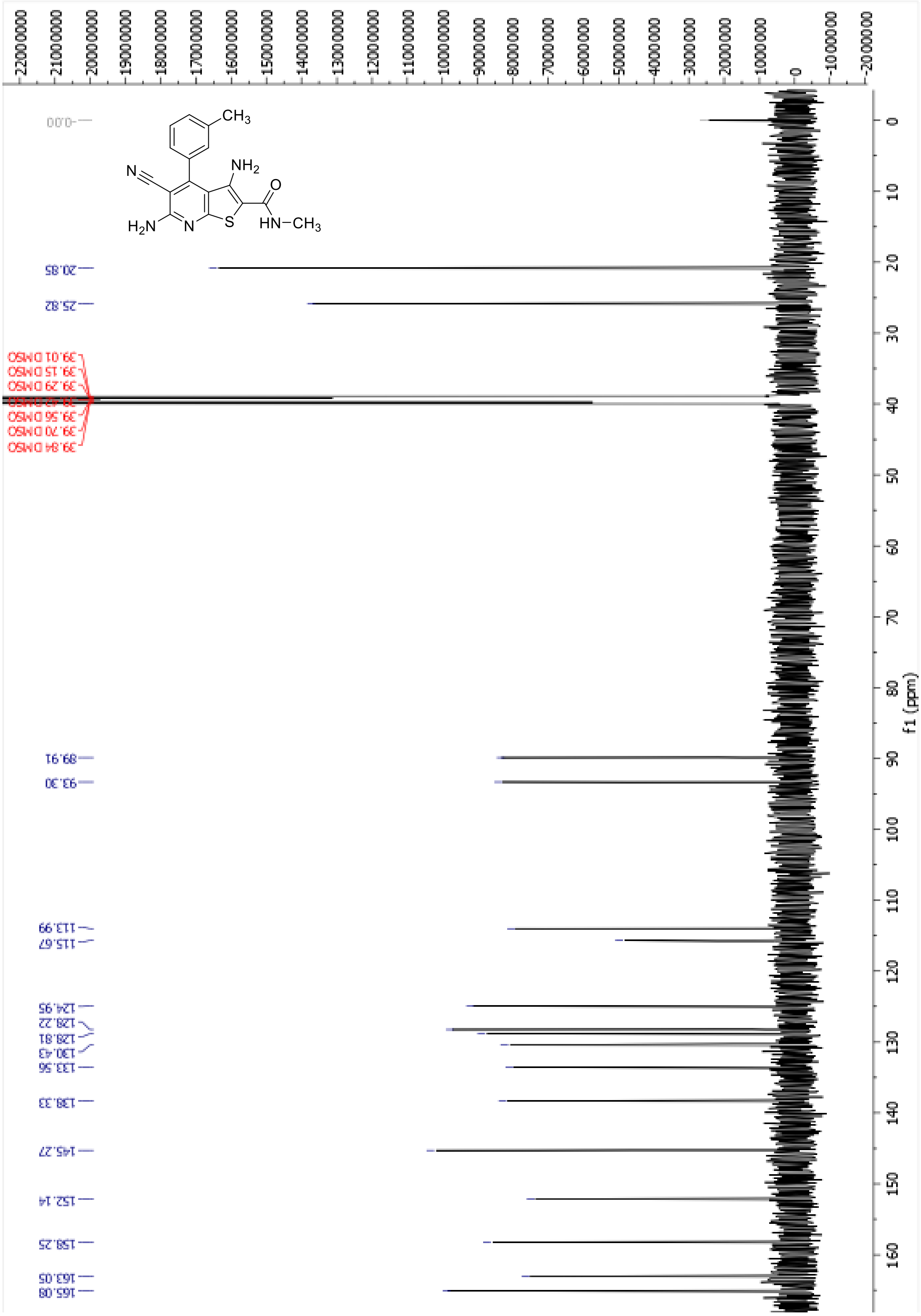

Figure S12: IR spectrum of **9j** (KuSaSch127).

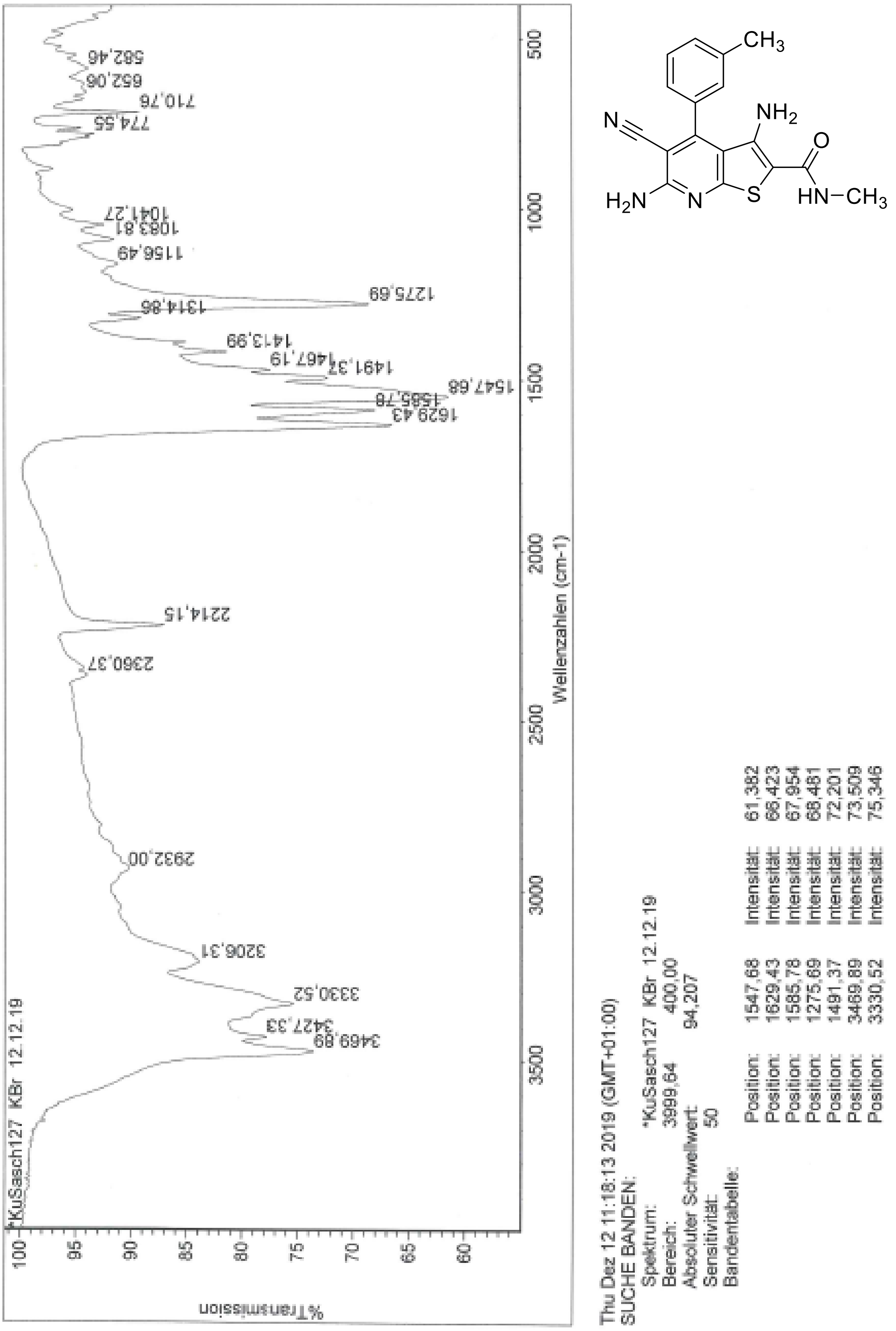

Figure S13: APCI-MS spectrum of **9j** (KuSaSch127).

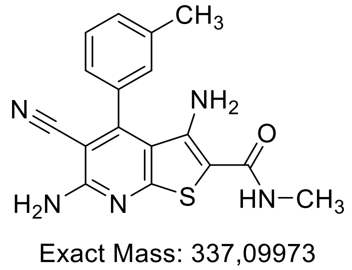

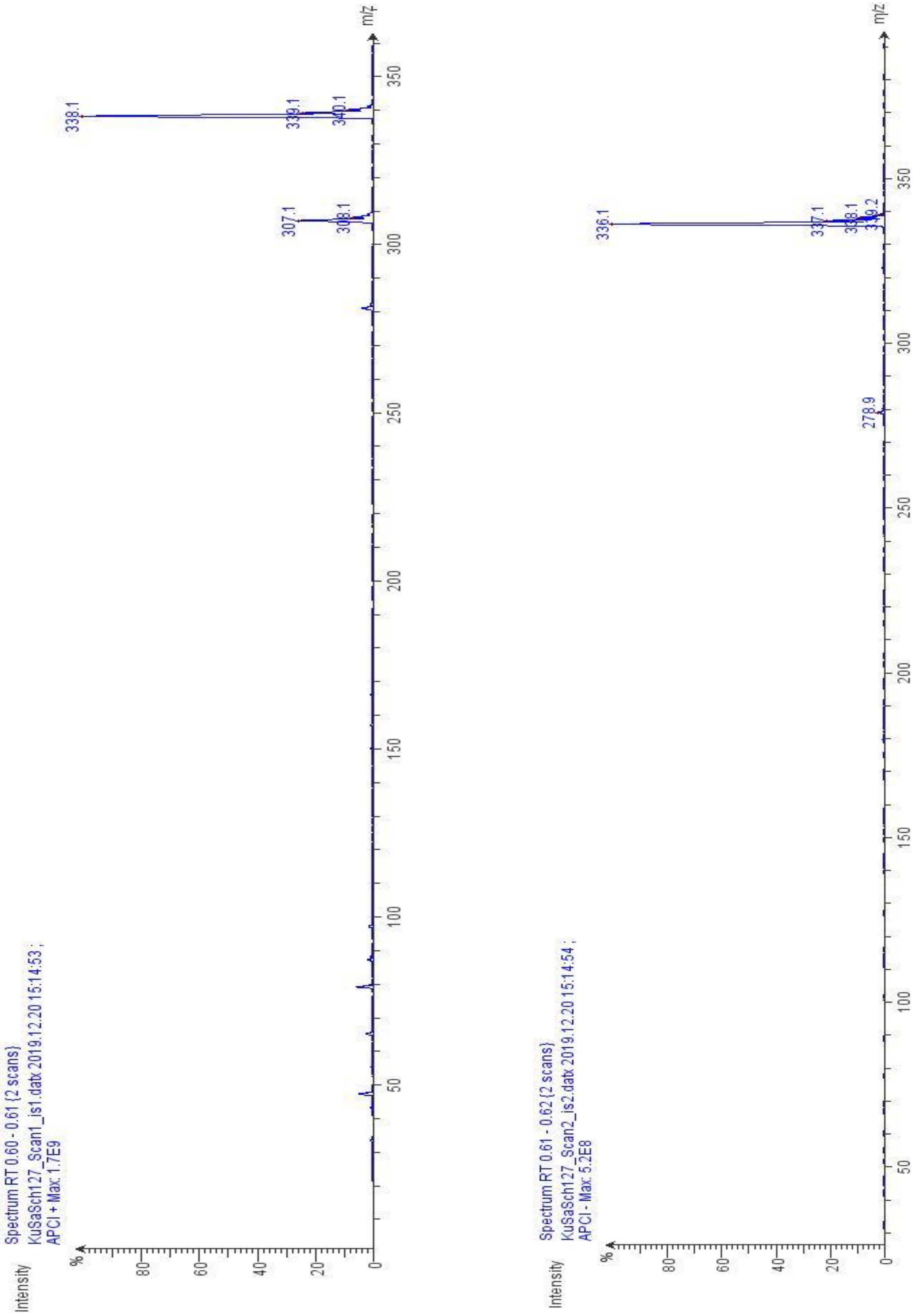

Figure S14: ^{1}H-NMR spectrum of **9m** (KuSaSch134).

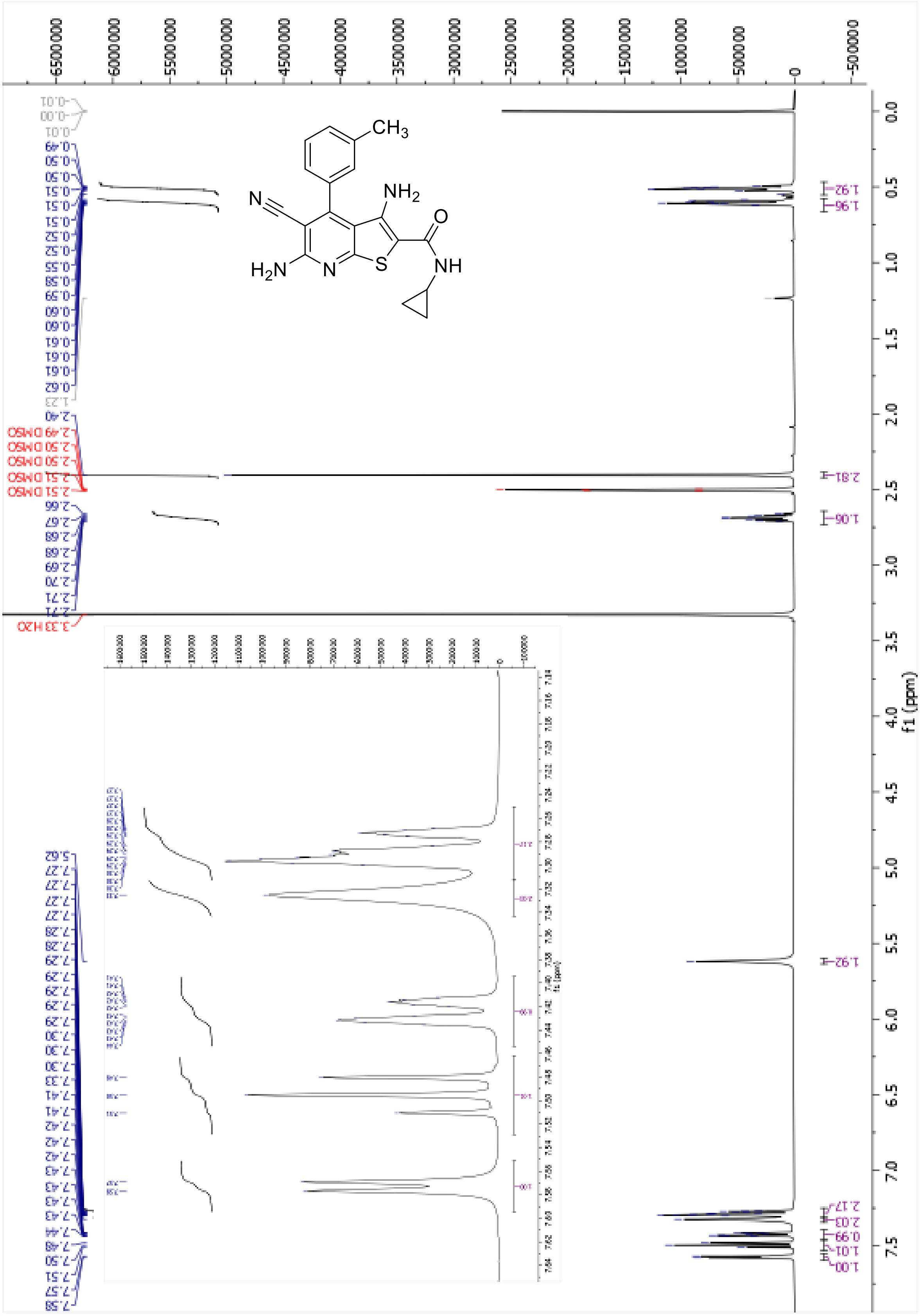

Figure S15: ^{13}C-NMR spectrum of **9m** (KuSaSch134).

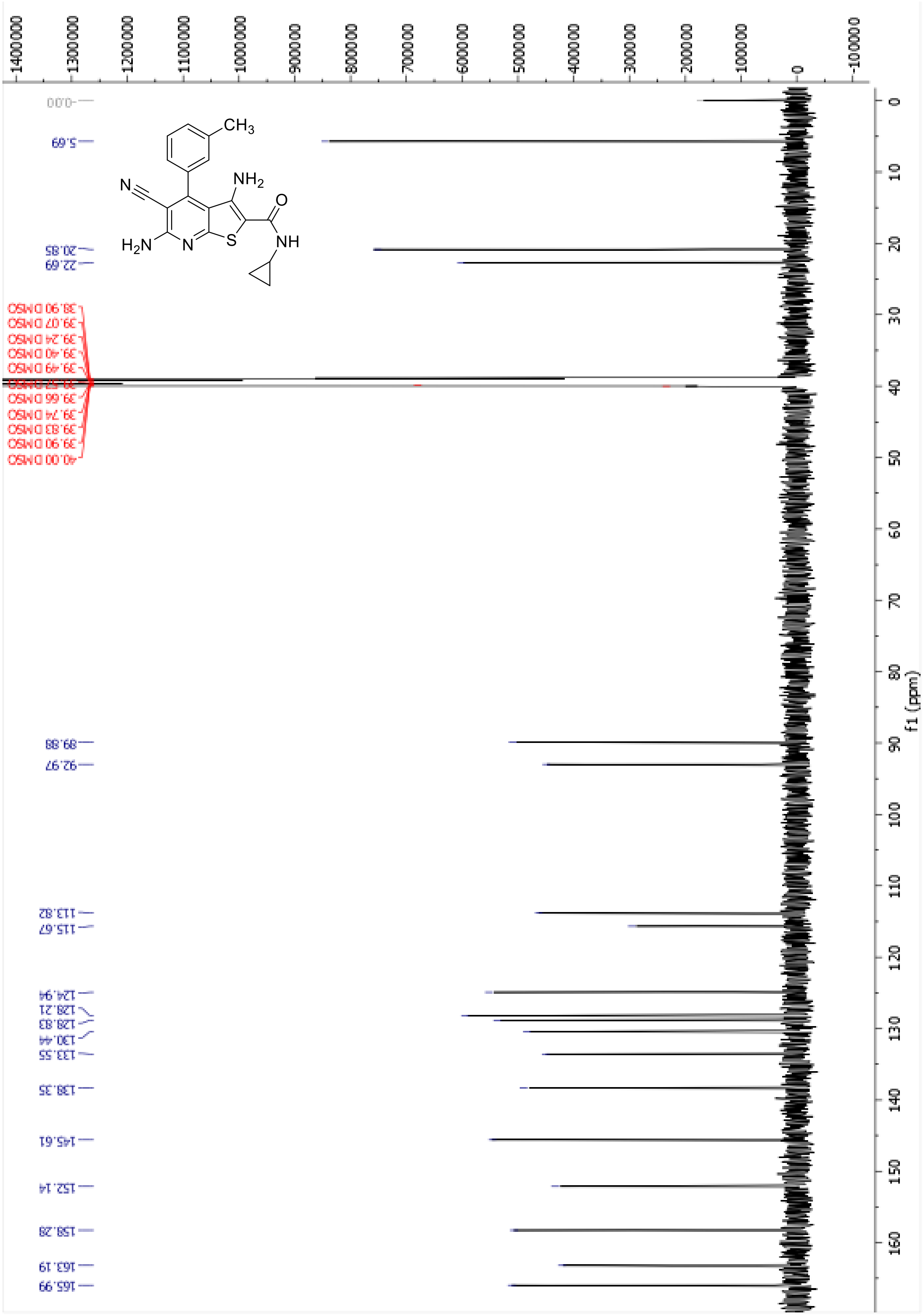

Figure S16: HSQC-NMR spectrum of **9m** (KuSaSch134). The black circle confirms two protons under one carbon signal.

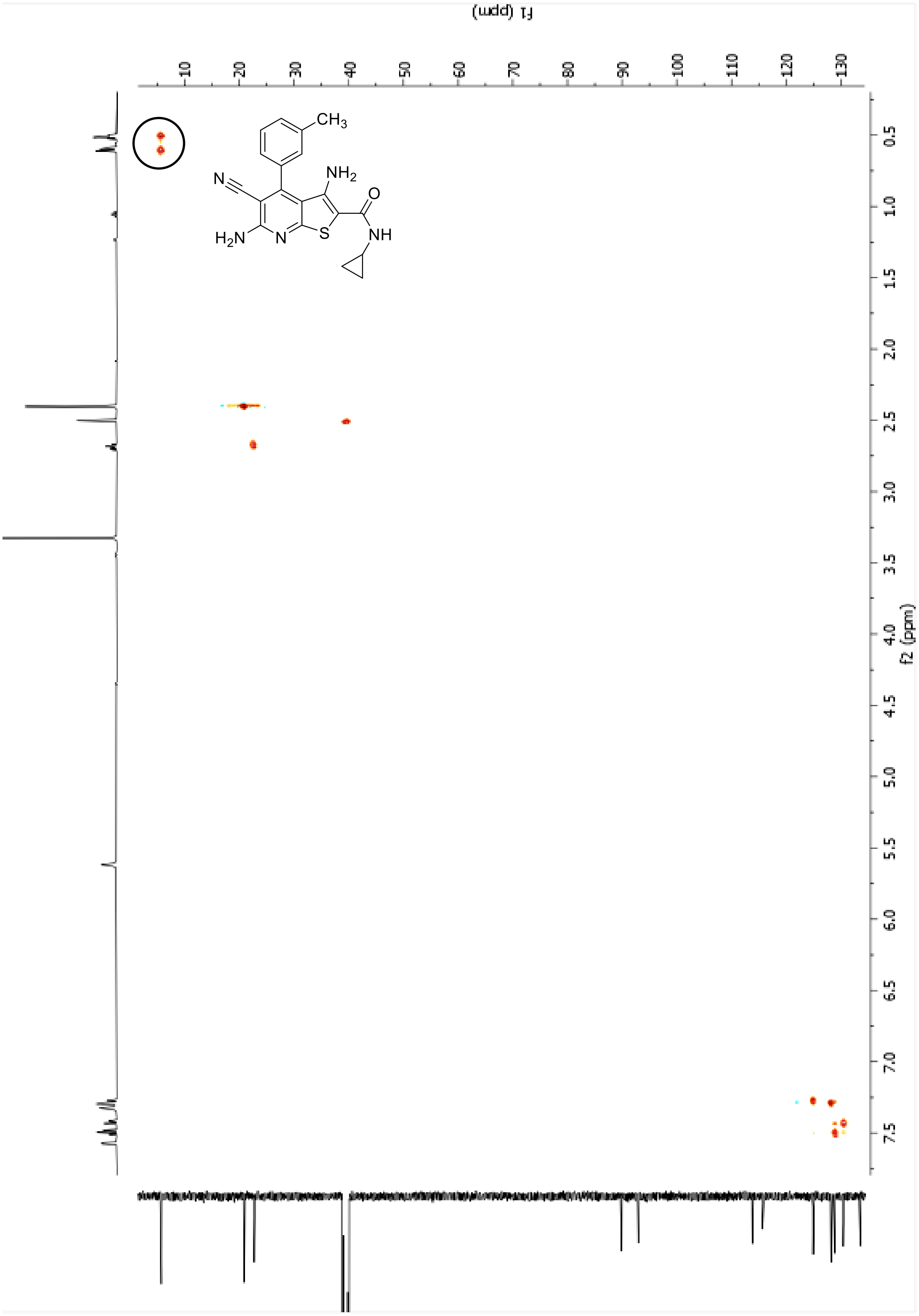

Figure S17: IR spectrum of **9m** (KuSaSch134).

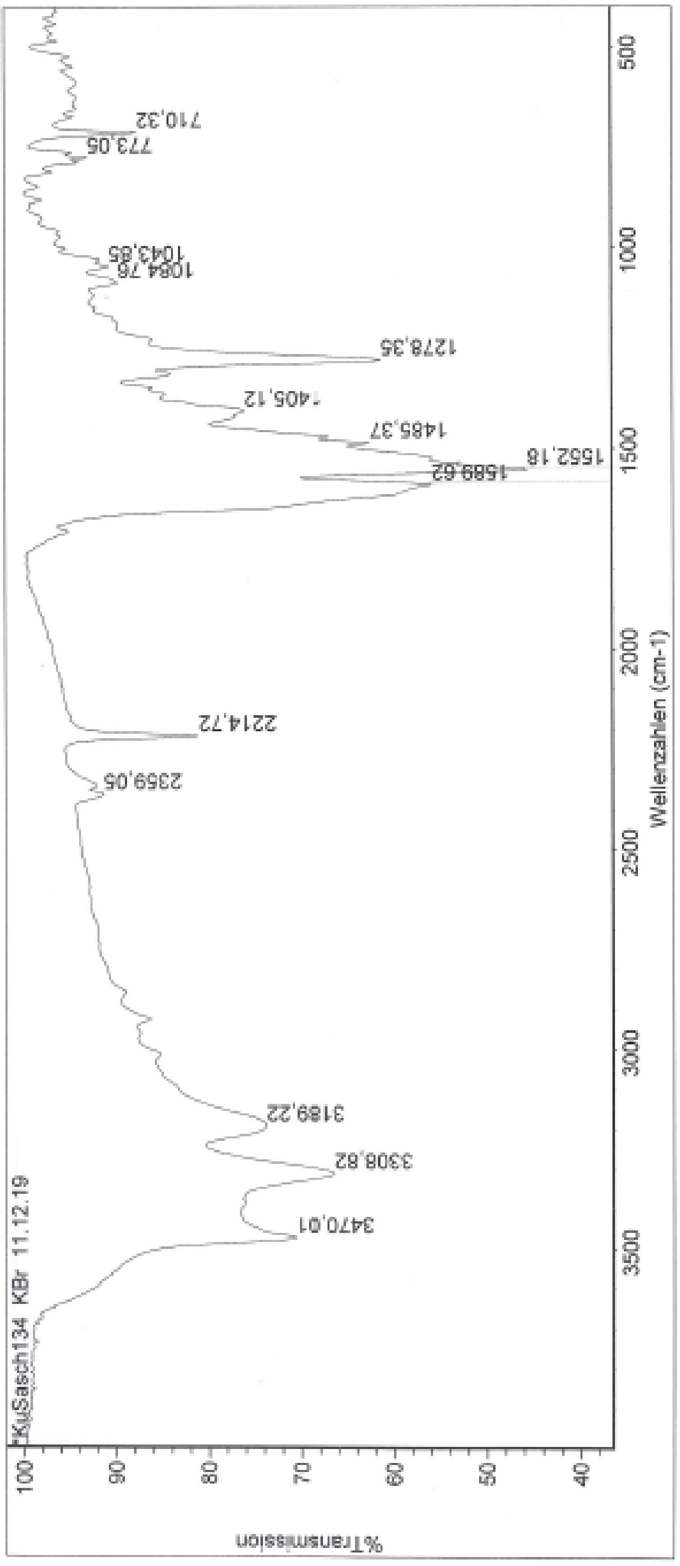

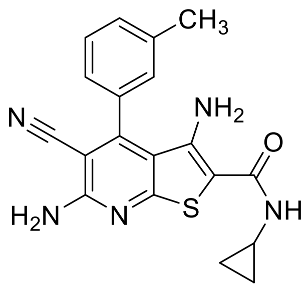

Figure S18: APCI-MS spectrum of **9m** (KuSaSch134).

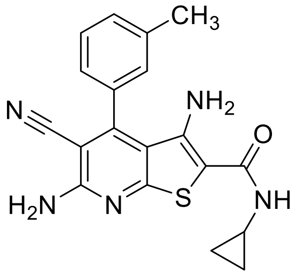

Exact Mass: 363,11538

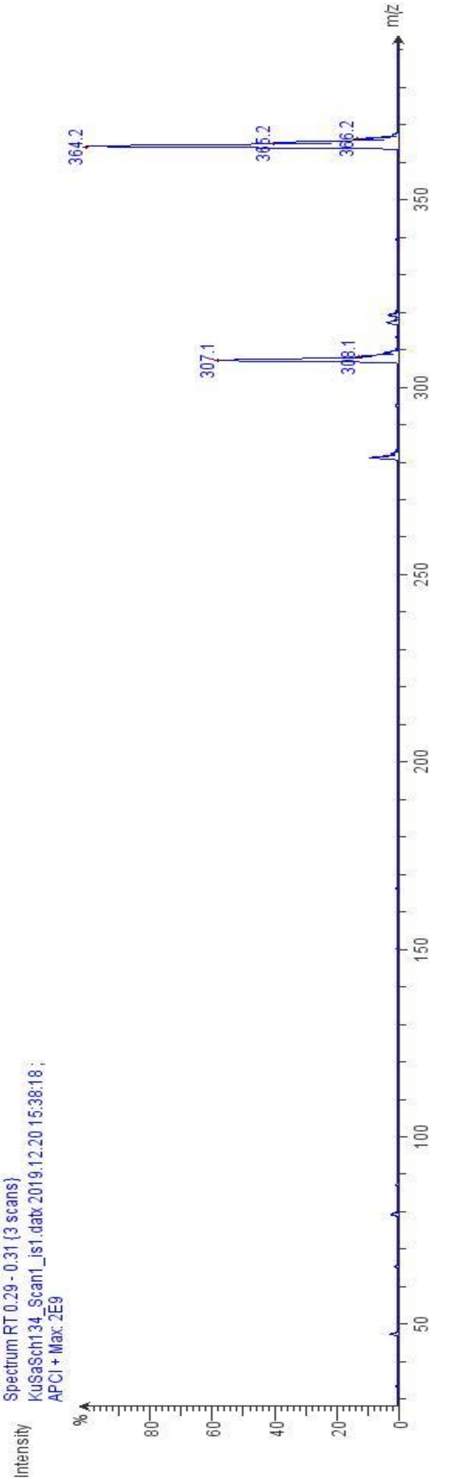

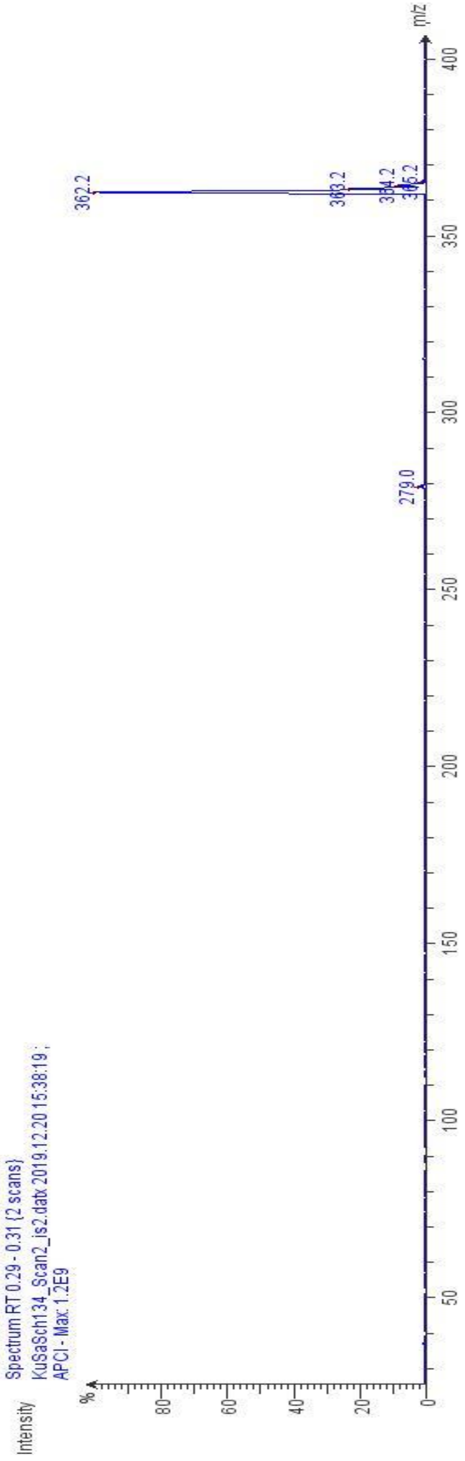

Figure S19: ^{1}H-NMR spectrum of **9n** (KuSaSch135).

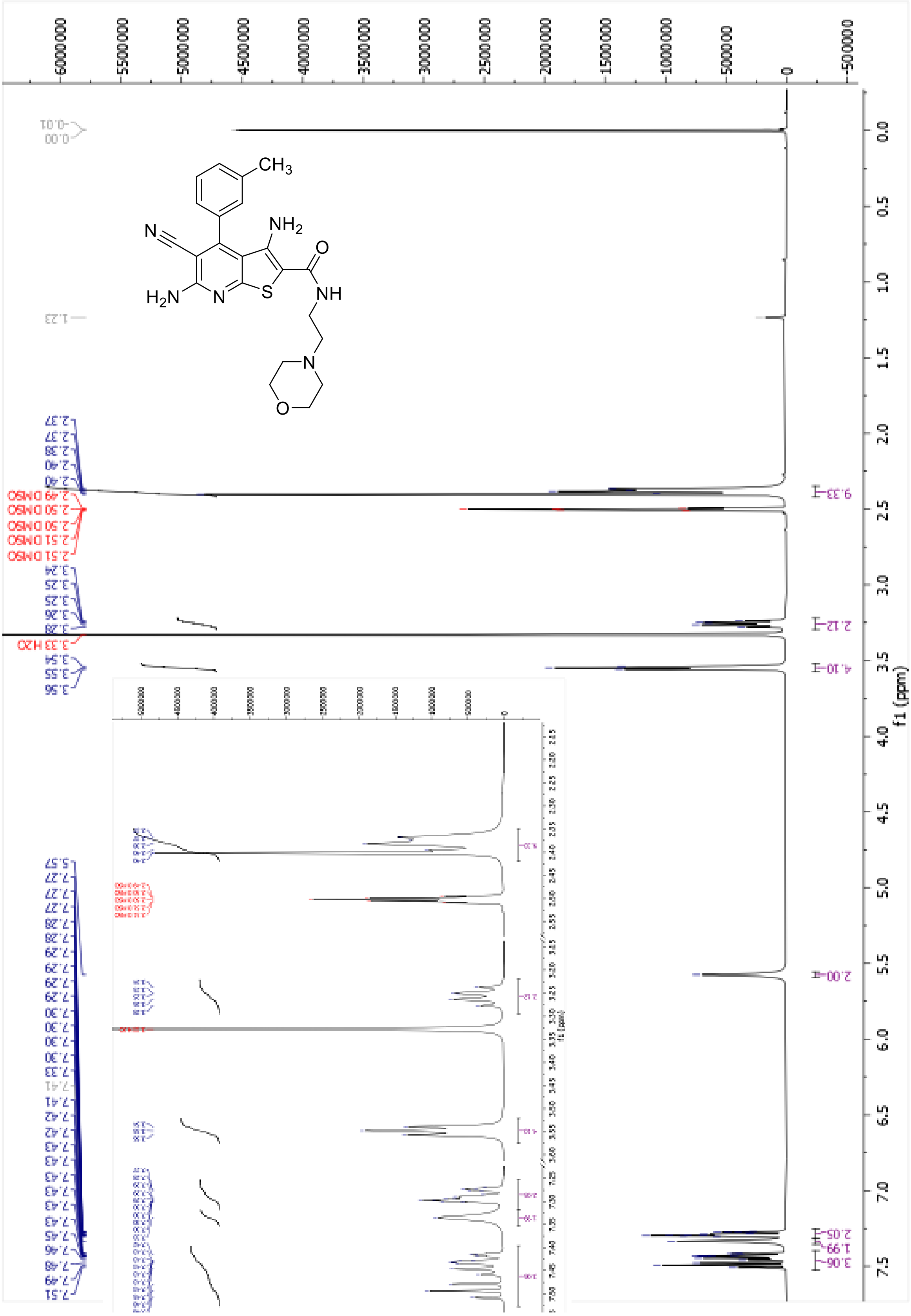

Figure S20: ^{13}C-NMR spectrum of **9n** (KuSaSch135).

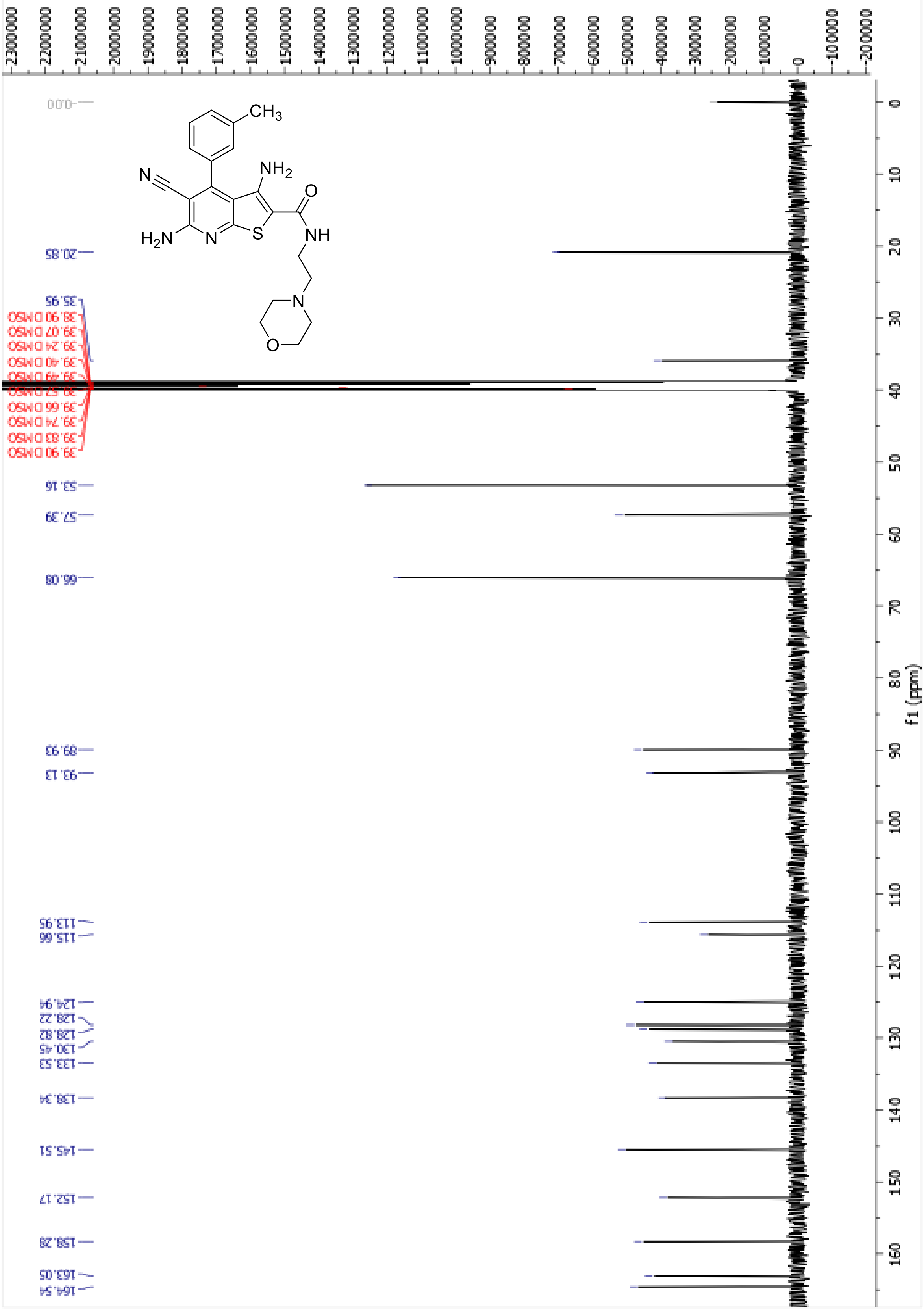

Figure S21: IR spectrum of **9n** (KuSaSch135).

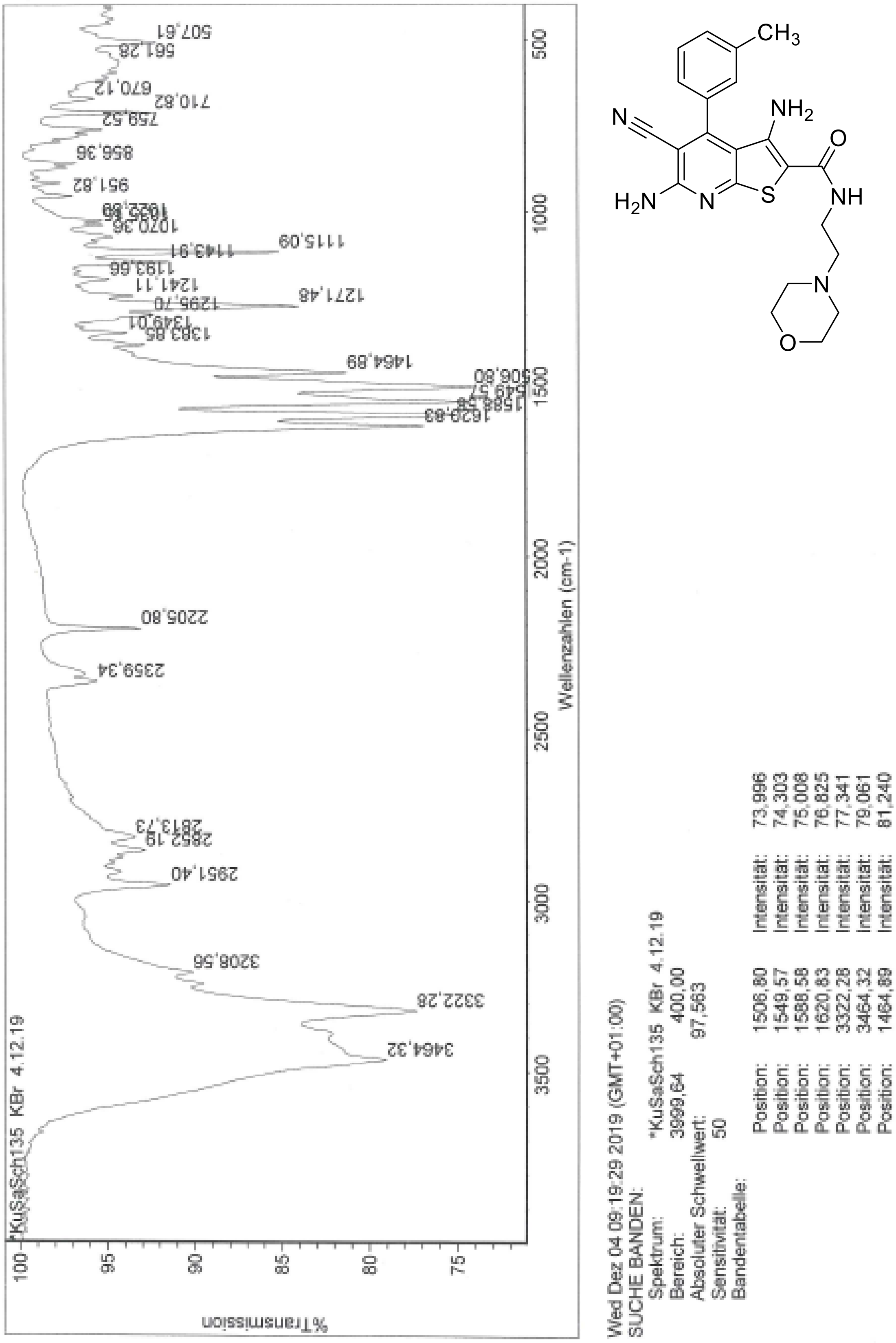

Figure S22: APCI-MS spectrum of **9n** (KuSaSch135).

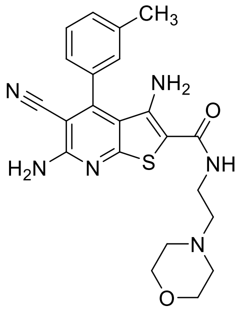

Exact Mass: 436,16814

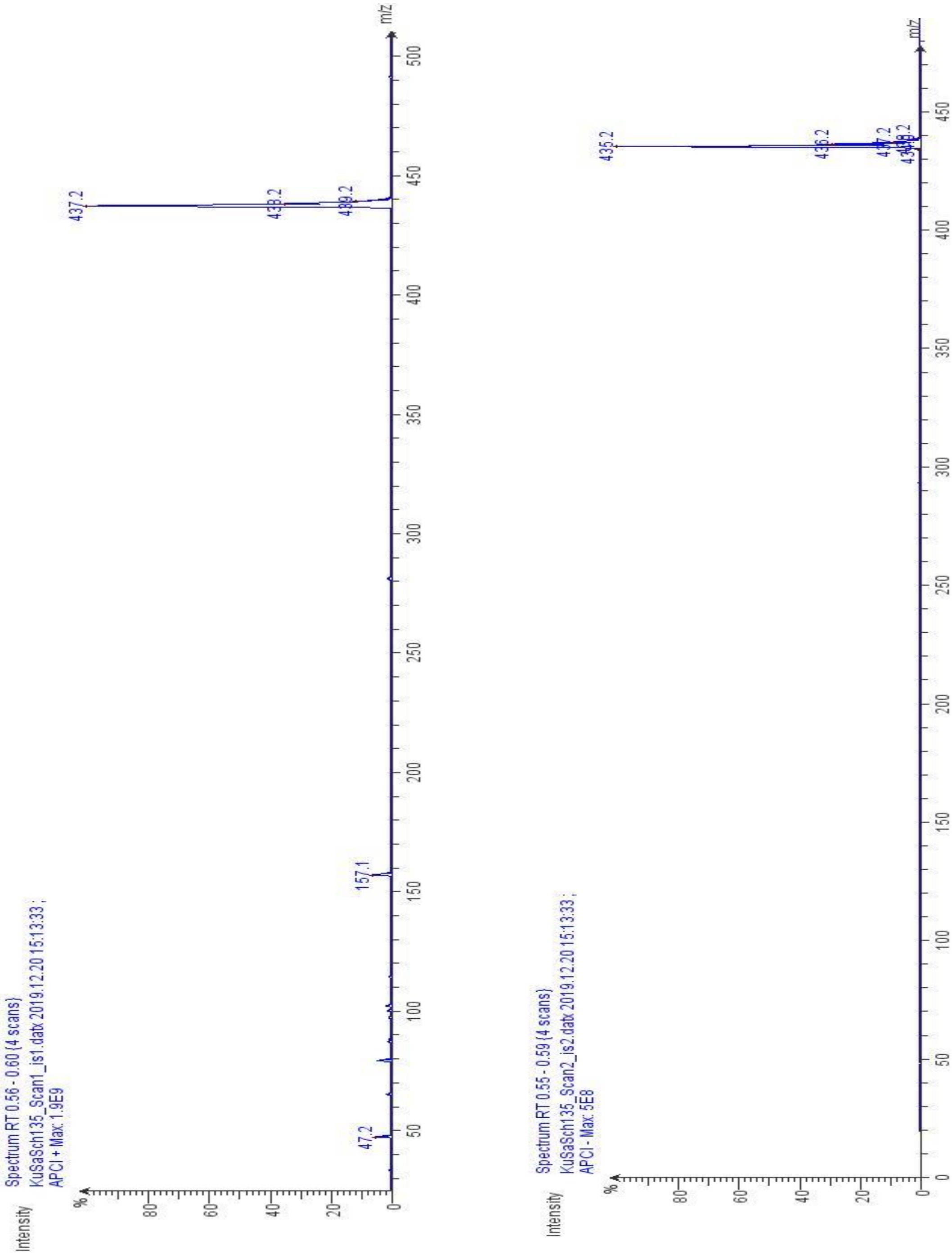

Figure S23: ^{1}H-NMR spectrum of **9y** (KuSaSch060).

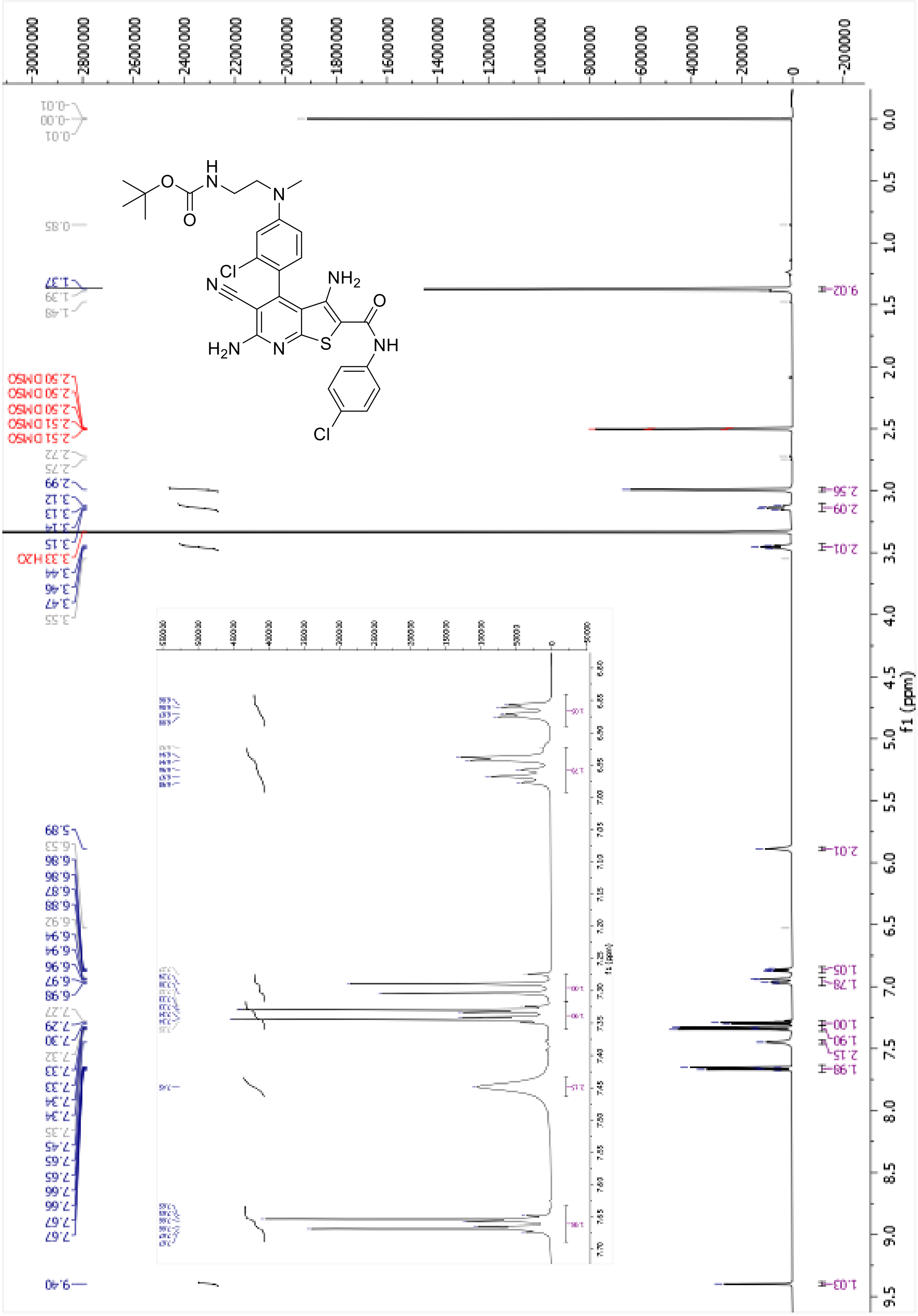

Figure S24: ^{13}C-NMR spectrum of **9y** (KuSaSch060).

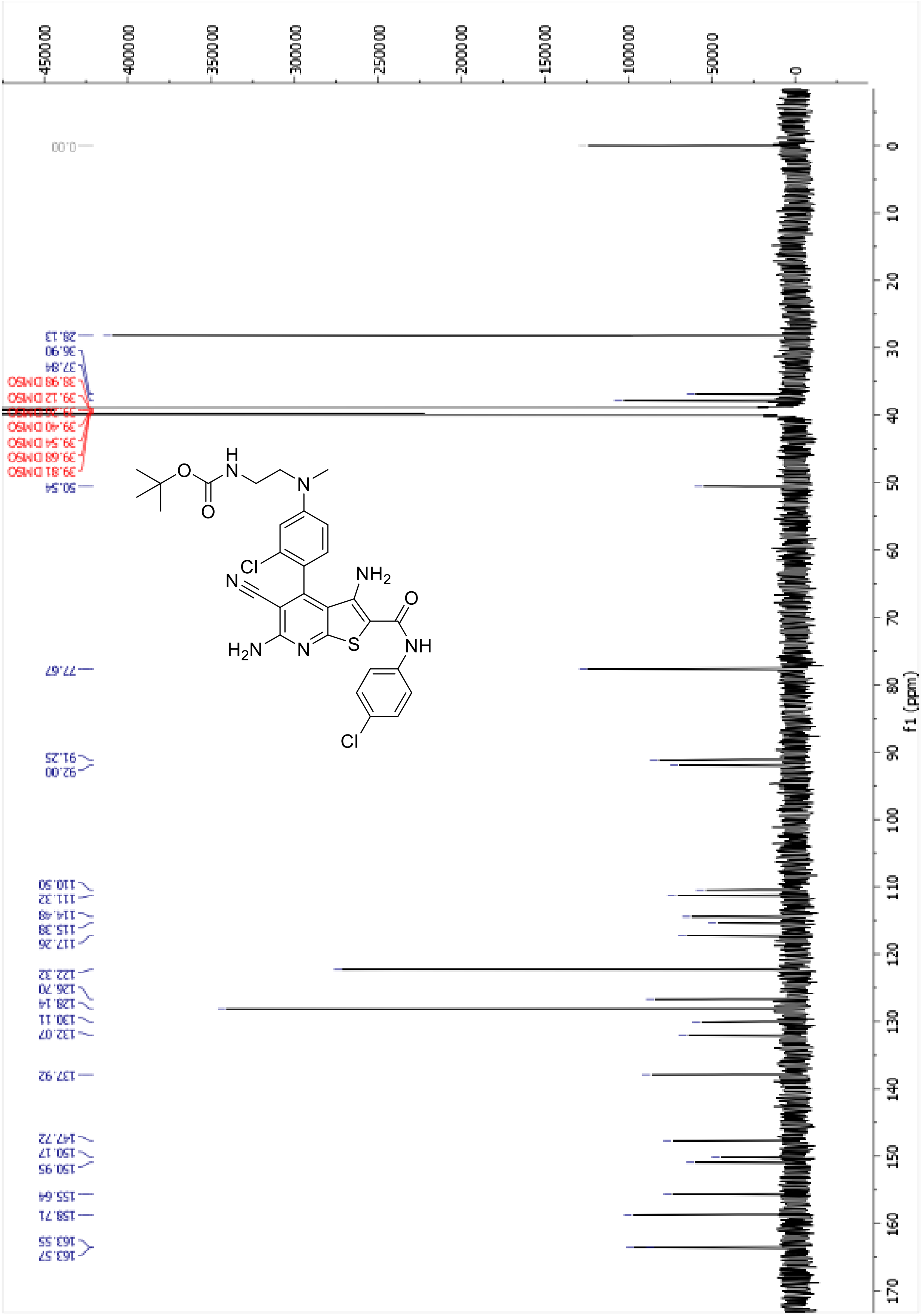

Figure S25: IR spectrum of **9y** (KuSaSch060).

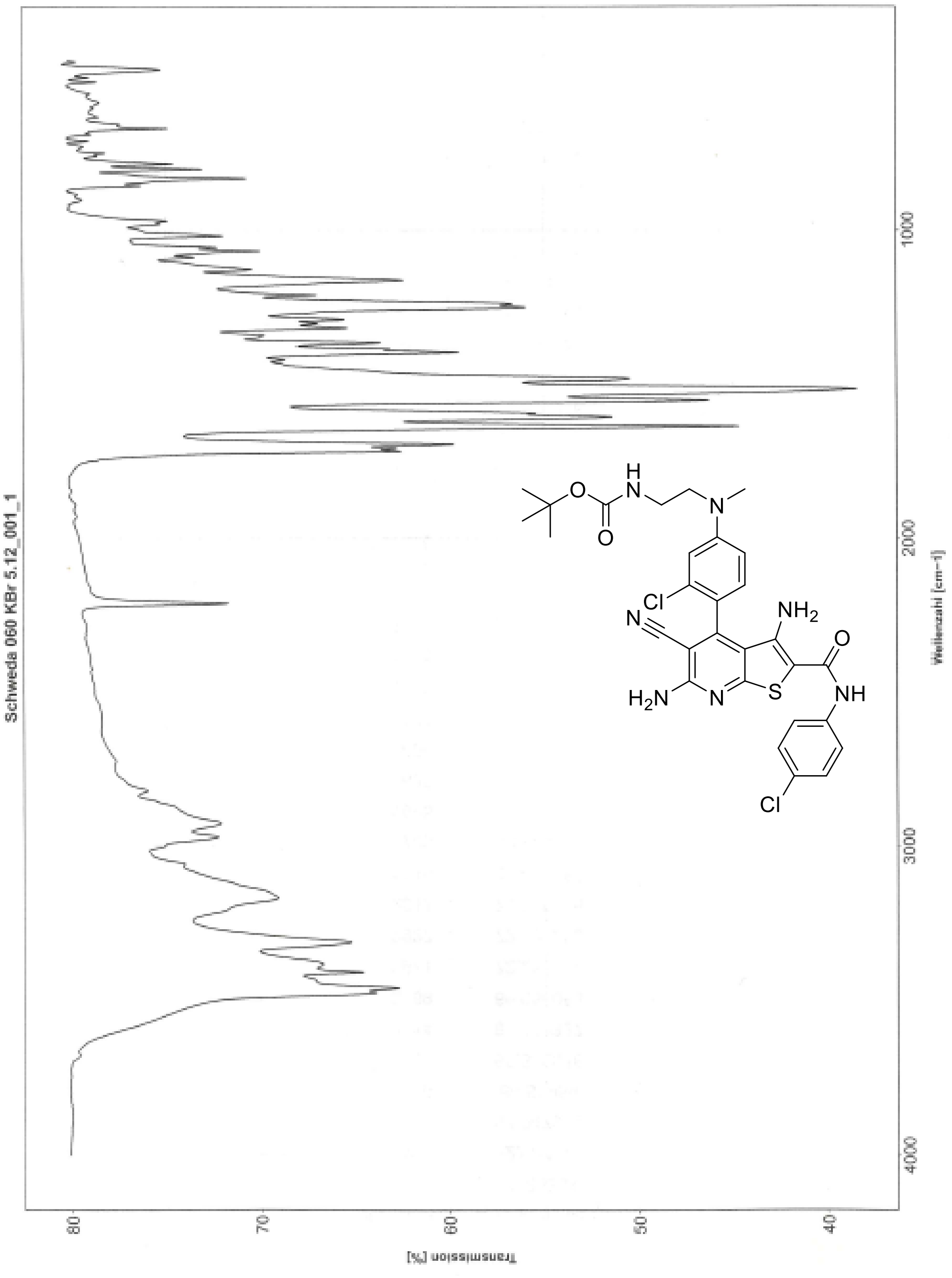

Figure S26: APCI-MS spectrum of **9y** (KuSaSch060).

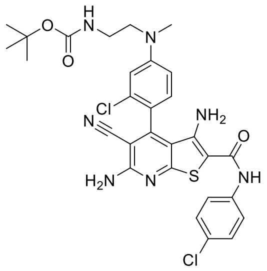

Exact Mass: 625,14296

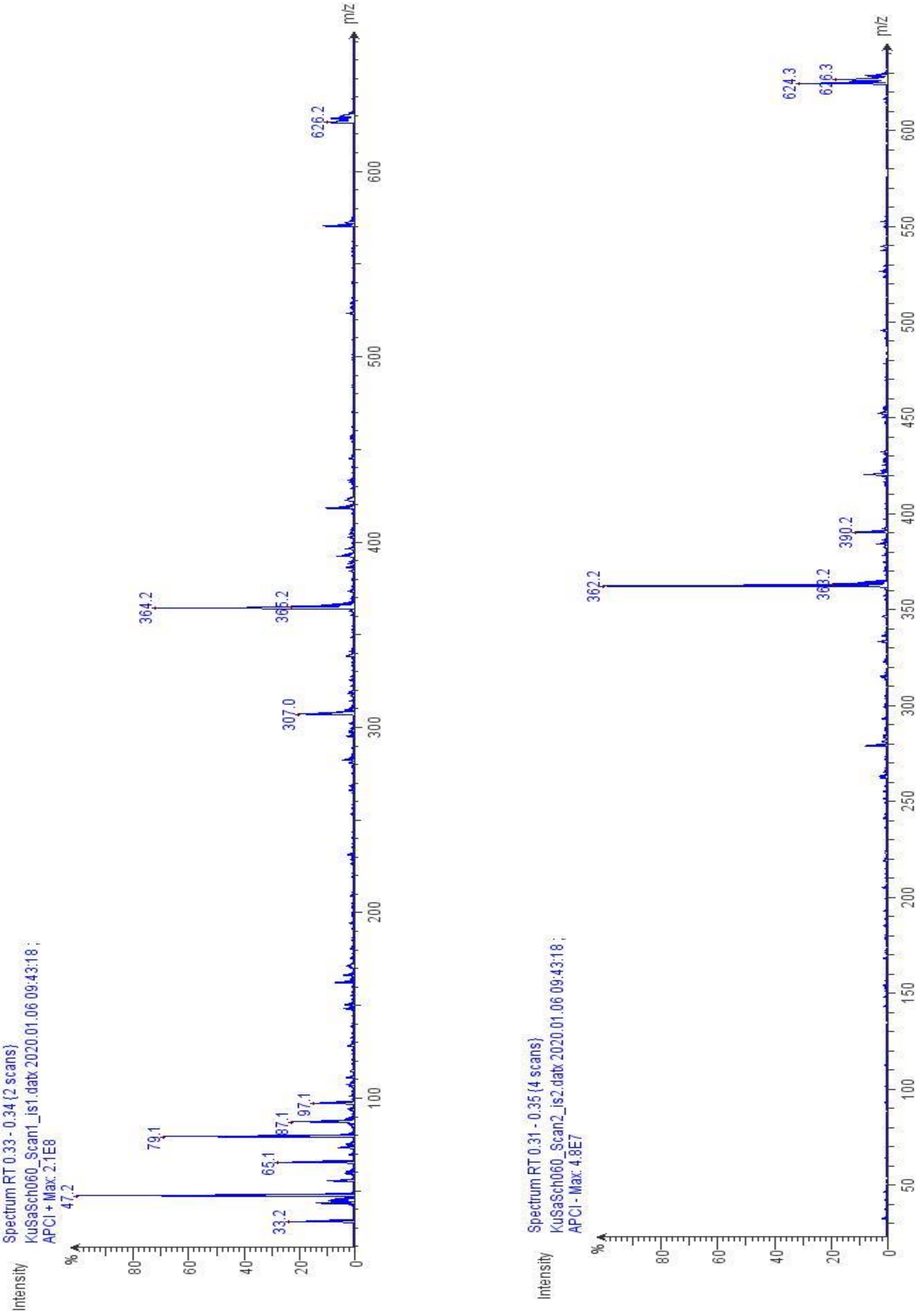

Figure S27: ^{1}H-NMR spectrum of **9z** (KuSaSch063).

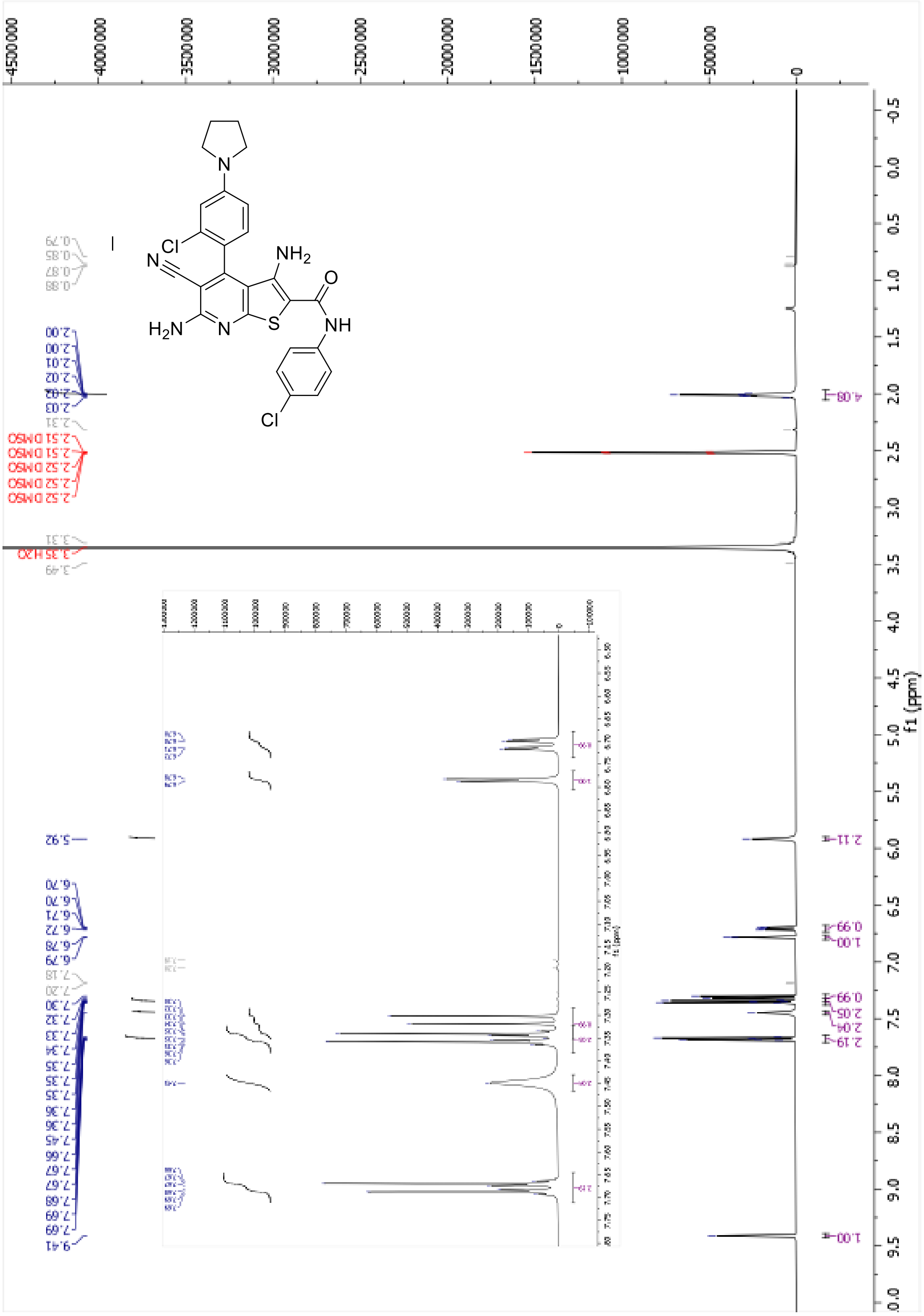

Figure S28: ^{13}C-NMR spectrum of **9z** (KuSaSch063).

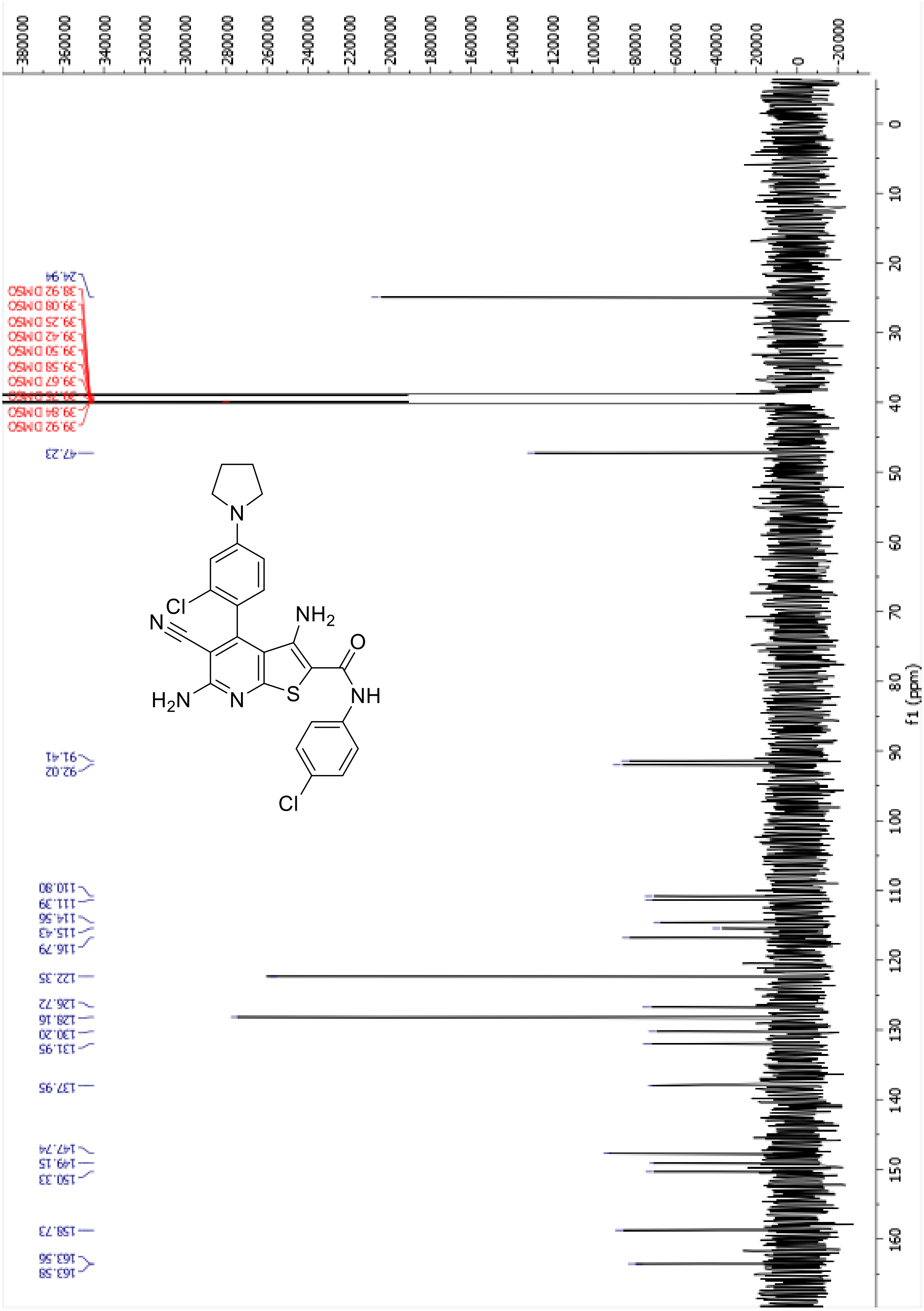

Figure S29: HSCQ-NMR spectrum of **9z** (KuSaSch063). The black circle confirms protons under the HDO signal.

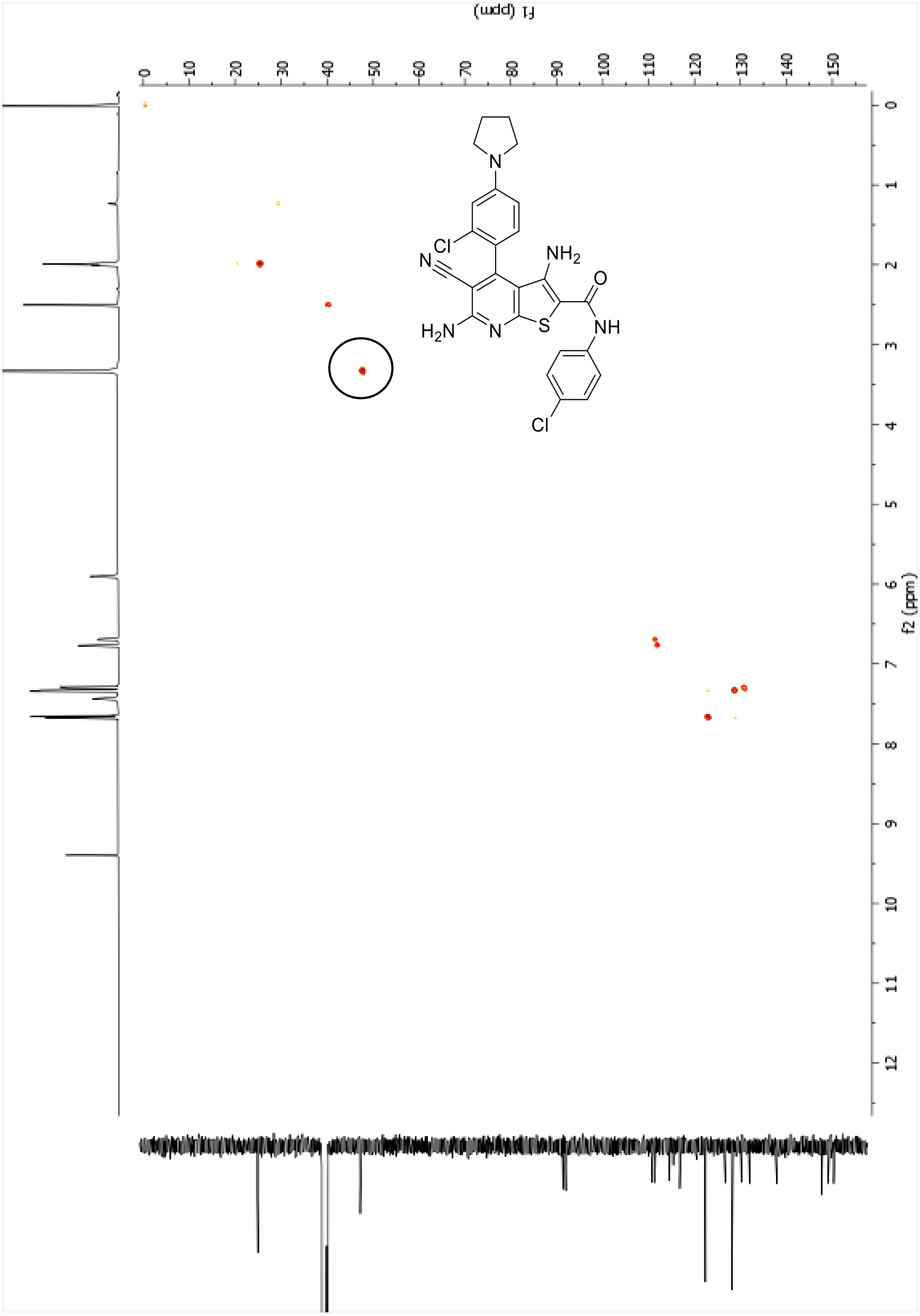

Figure S30: IR spectrum of **9z** (KuSaSch063).

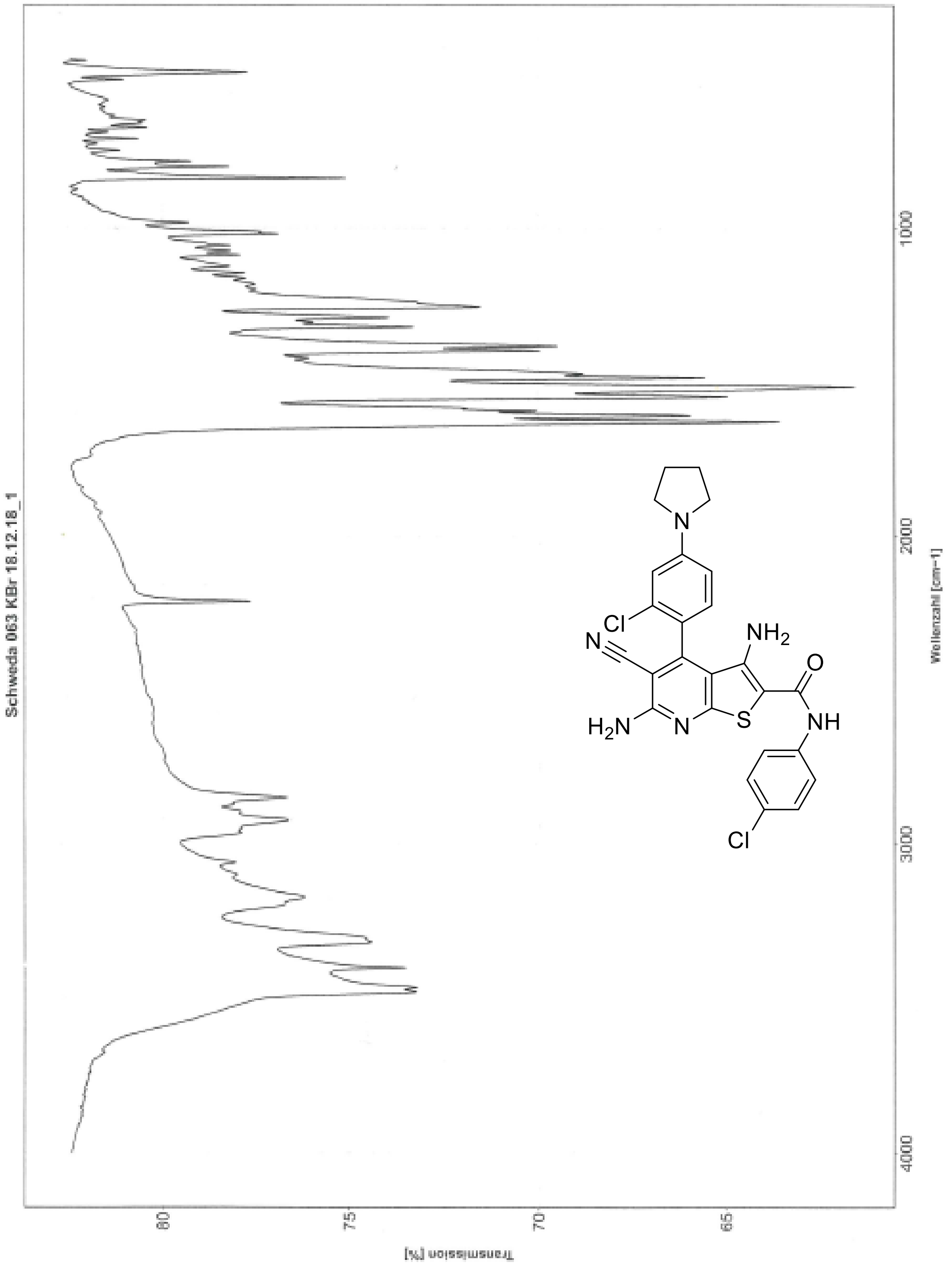

Figure S31: APCI-MS of **9z** (KuSaSch063).

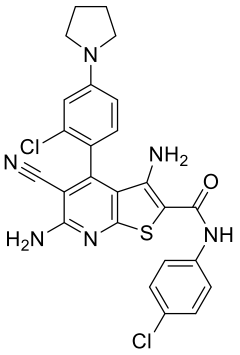

Exact Mass: 522,07964

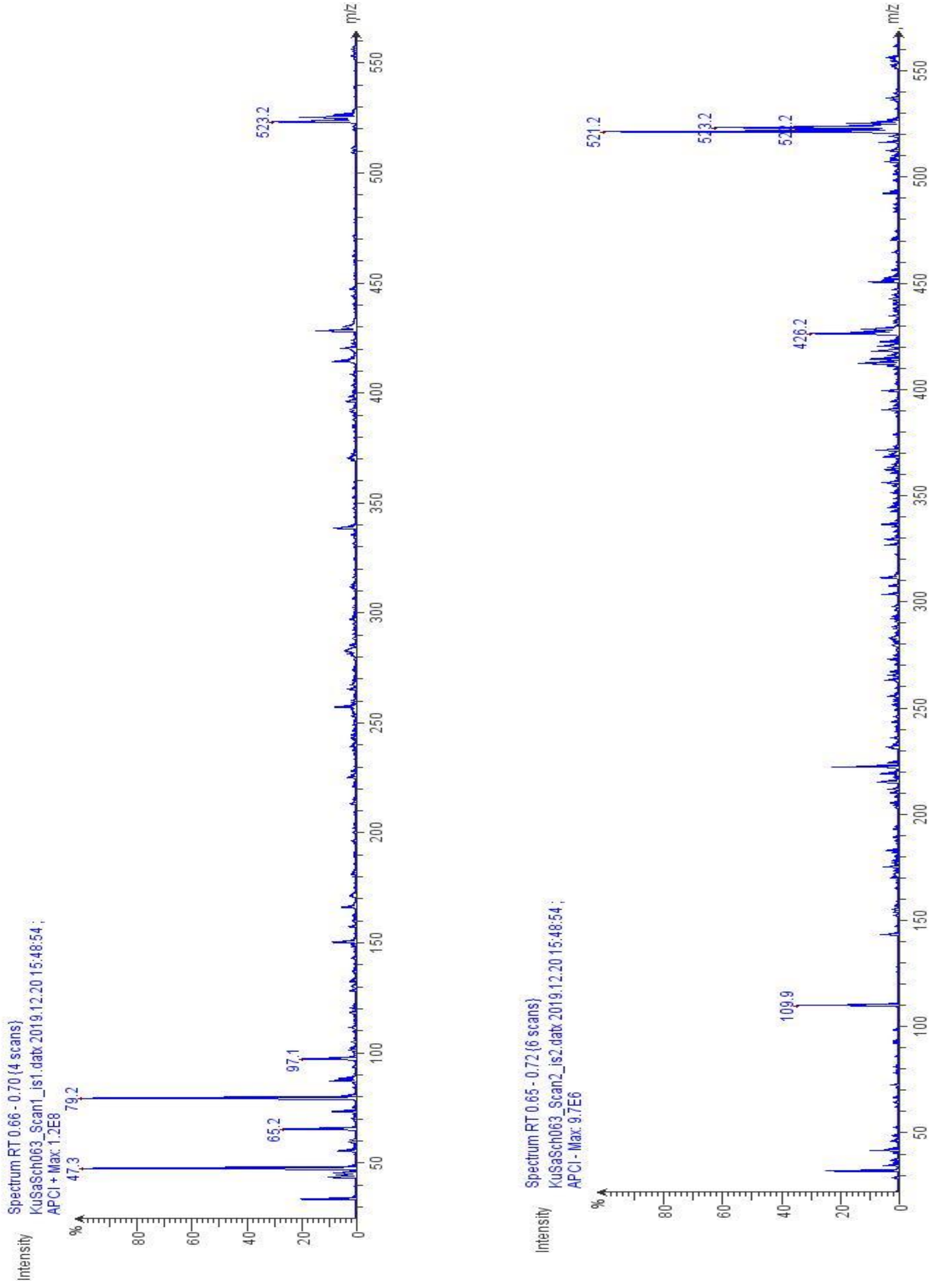

Figure S32: ^{1}H-NMR spectrum of **9ac** (KuSaSch073).

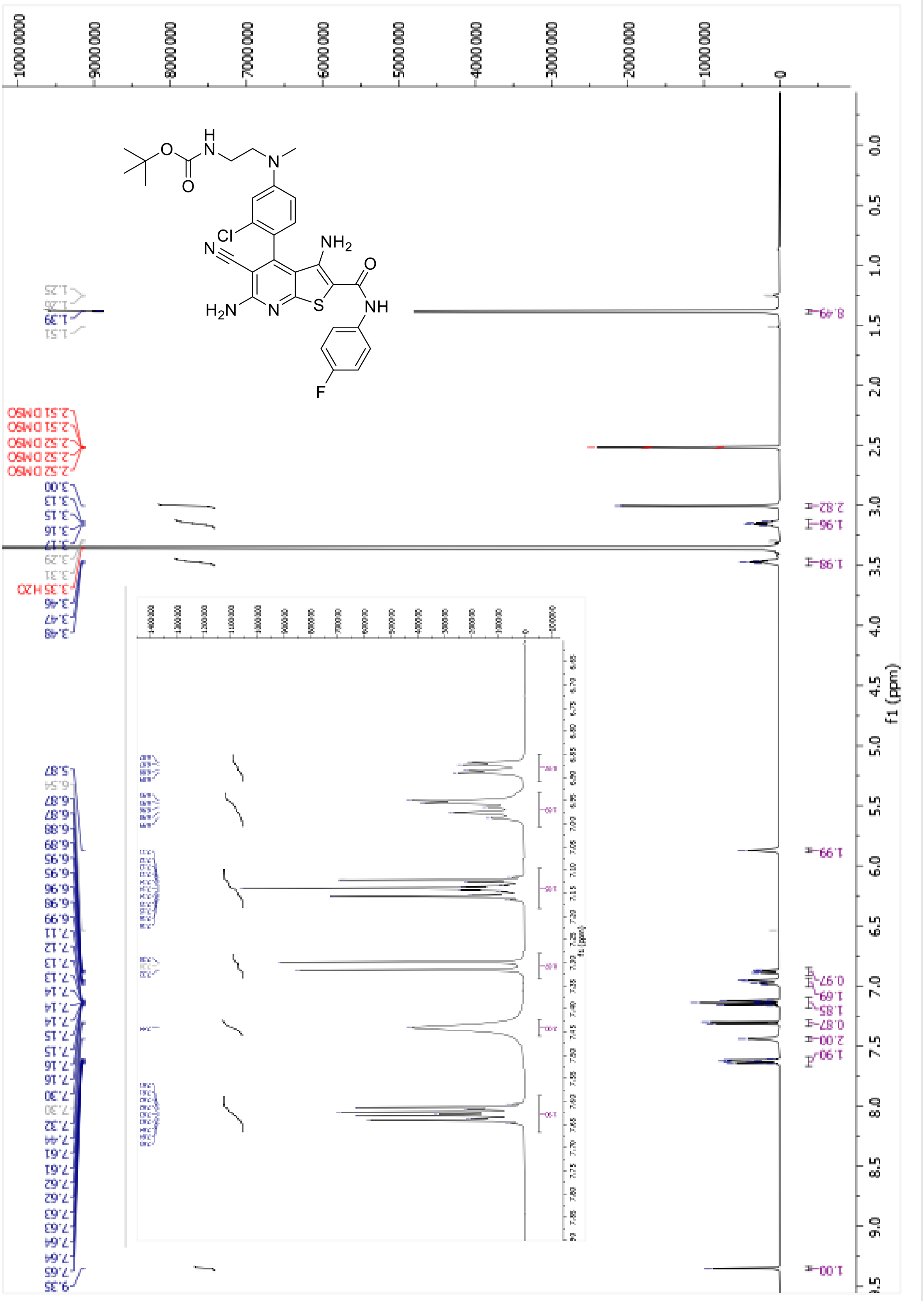

Figure S33: ^{13}C-NMR spectrum of **9ac** (KuSaSch073).

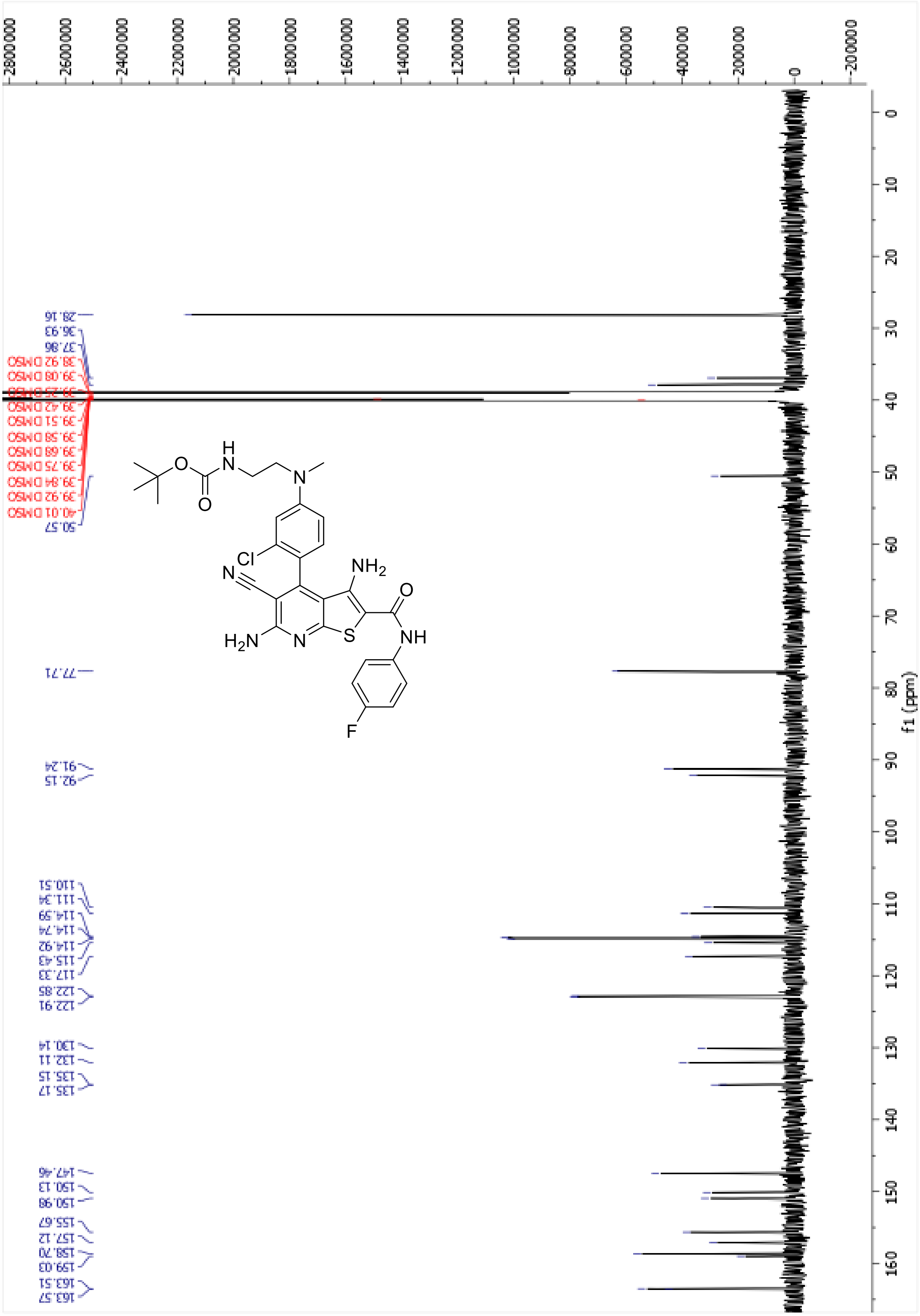

Figure S34: IR spectrum of **9ac** (KuSaSch073).

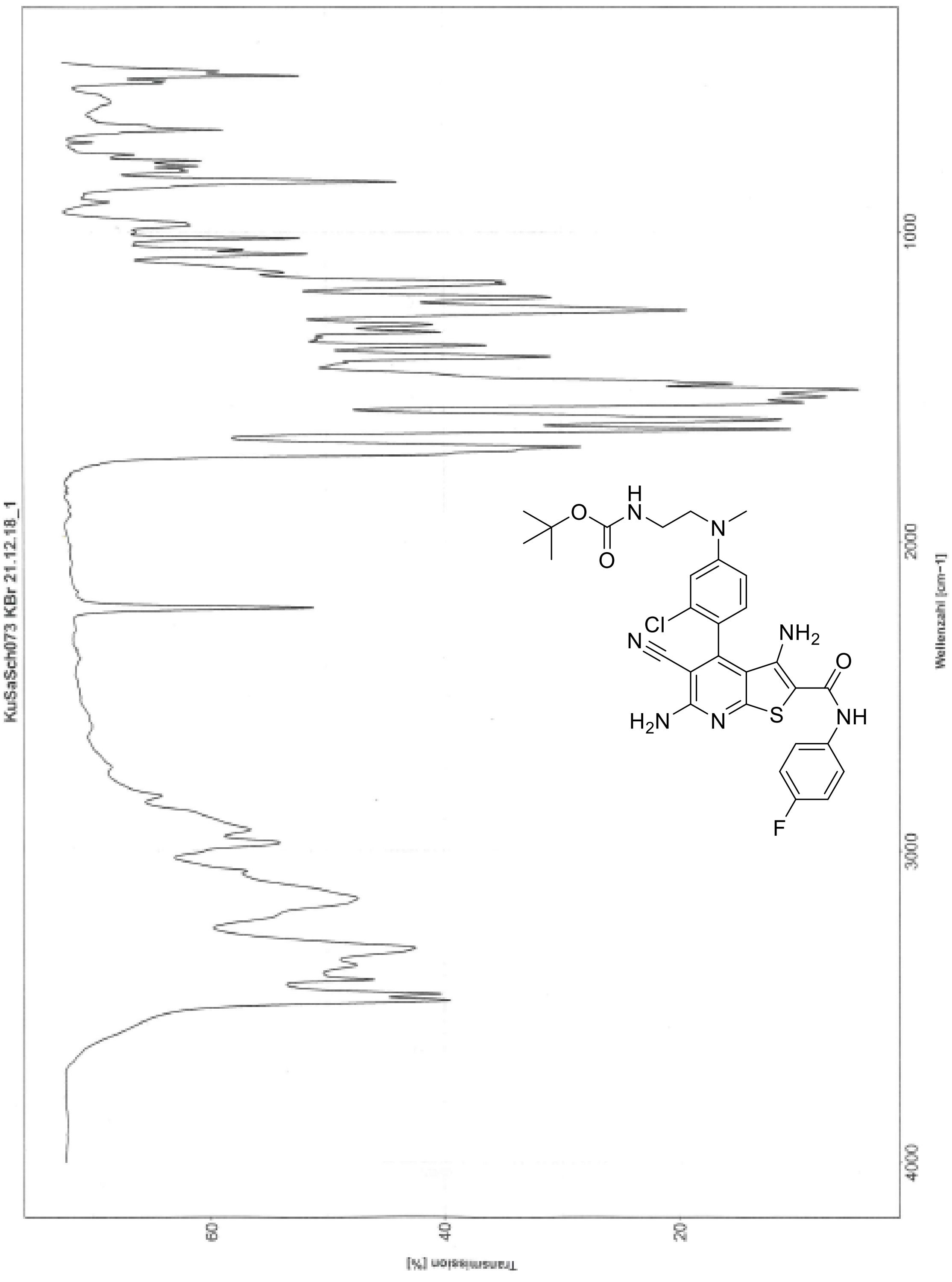

Figure S35: APCI-MS spectrum of **9ac** (KuSaSch073).

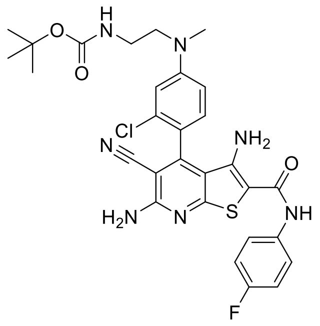

Exact Mass: 609,17251

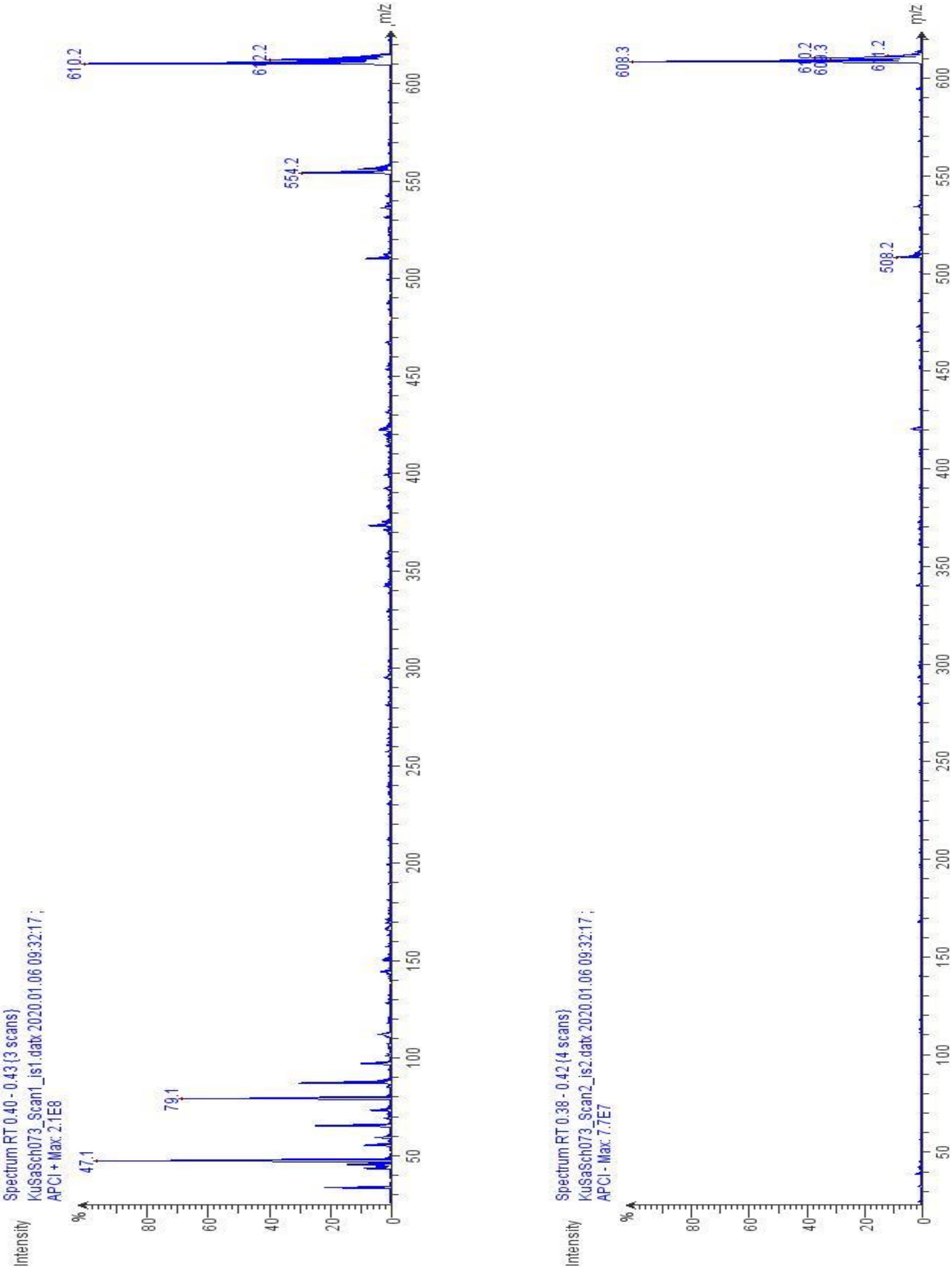

Figure S36: ^{1}H-NMR spectrum of **17a** (KuSaSch095).

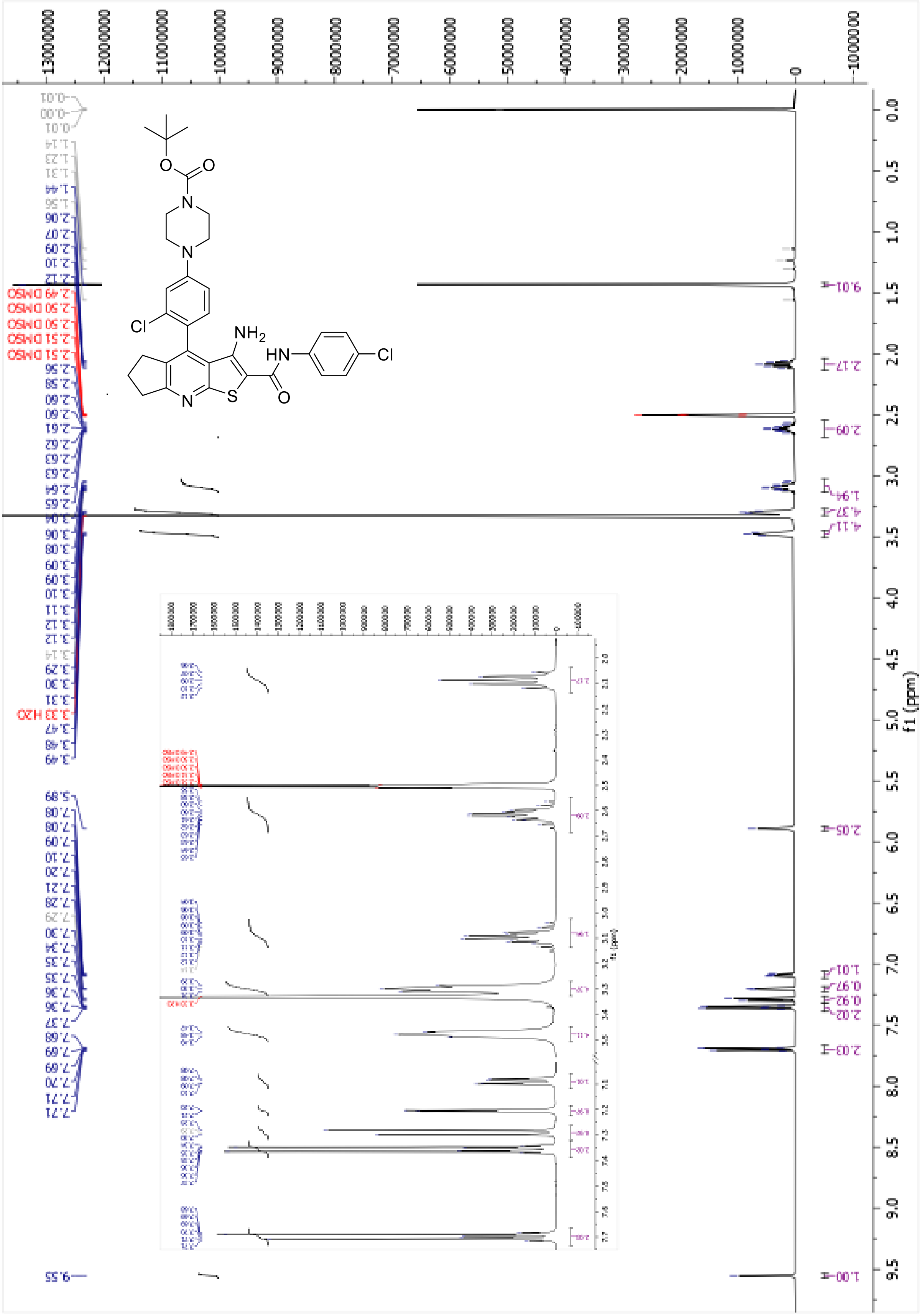

Figure S37: ^{13}C-NMR spectrum of **17a** (KuSaSch095).

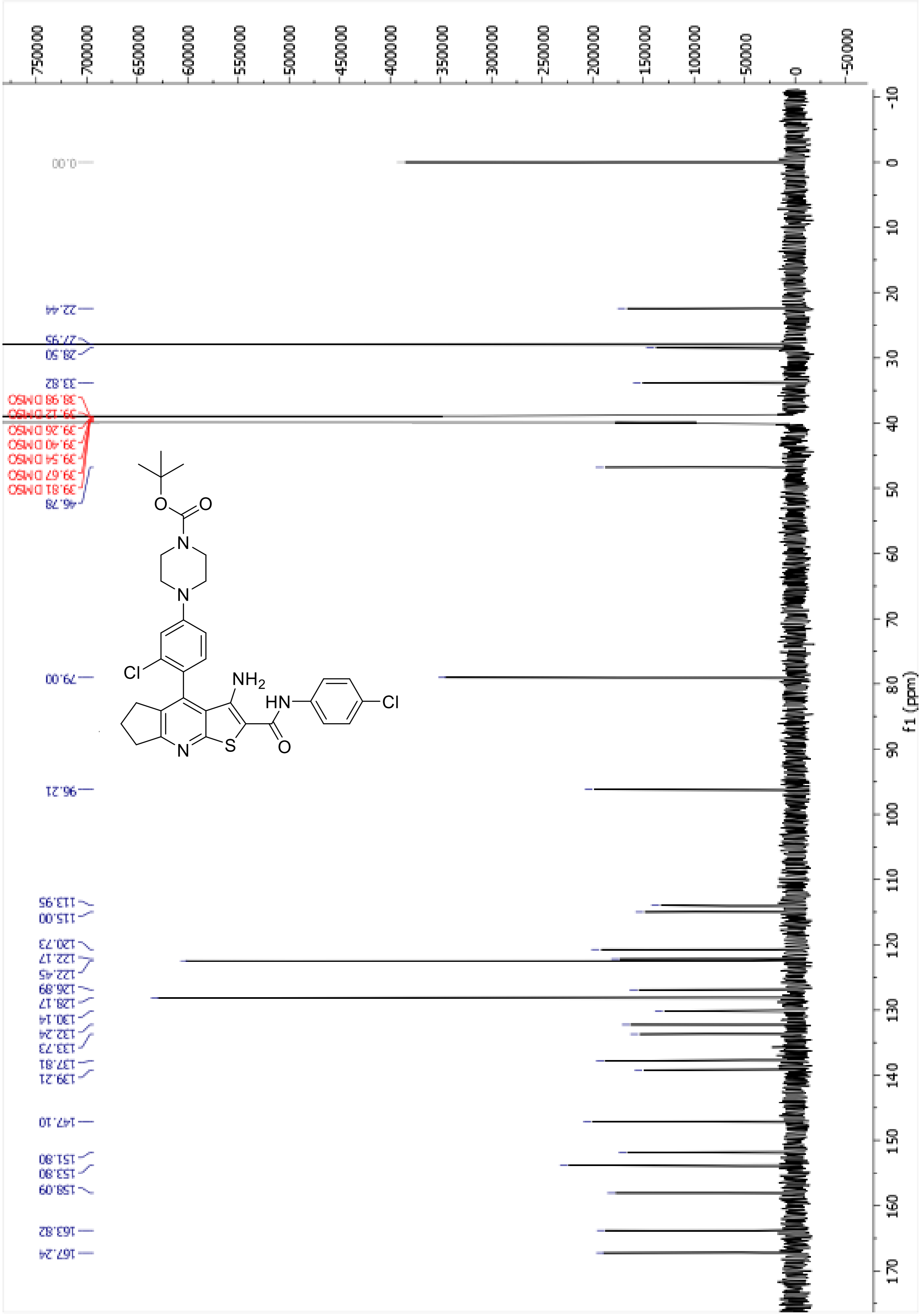

Figure S38: HSCQ-NMR spectrum of **17a** (KuSaSch095). The black circle confirms 4 protons under a missing signal for two carbons.

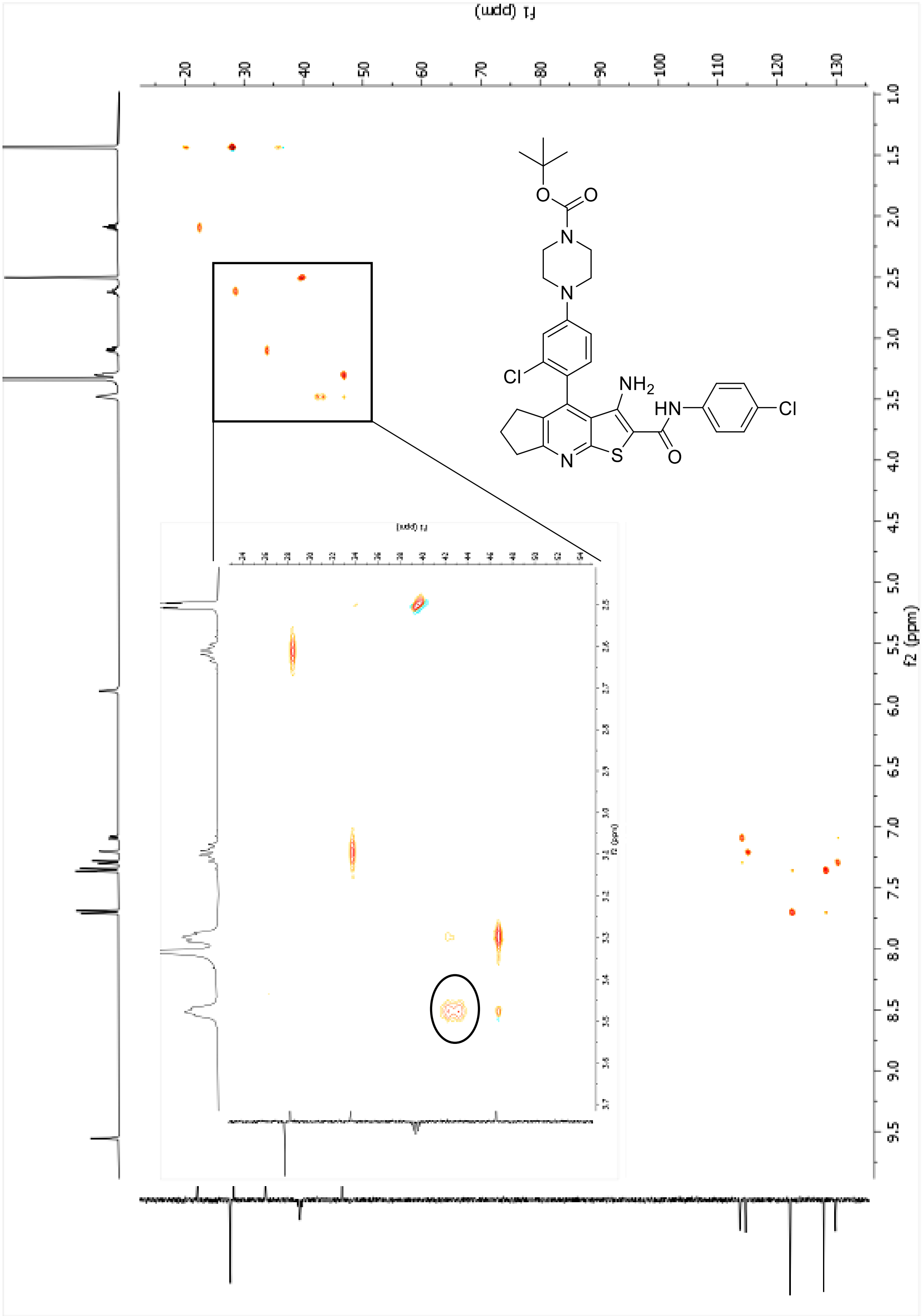

Figure S39: IR spectrum of **17a** (KuSaSch095).

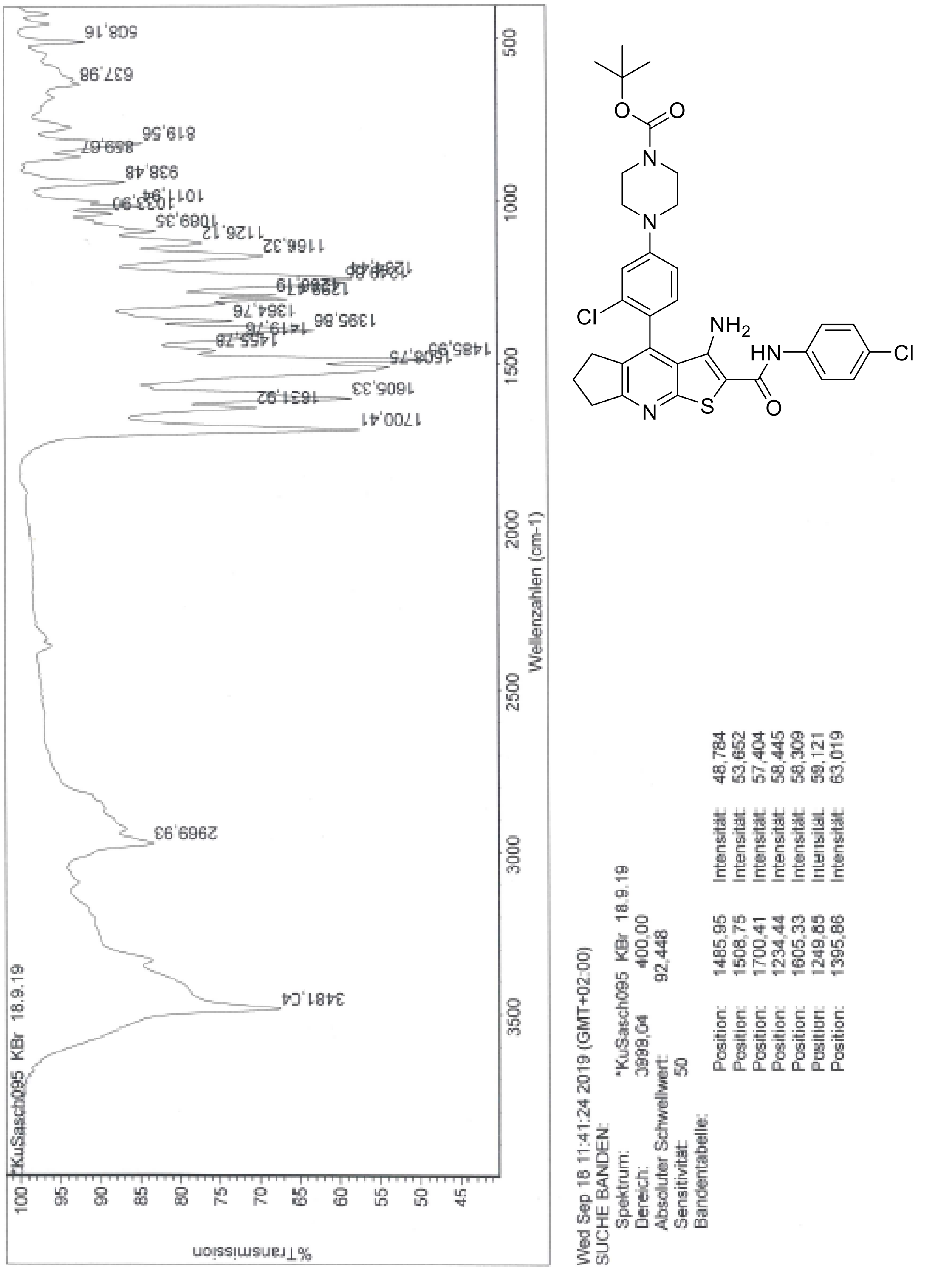

Figure S40: APCI-MS spectrum of **17a** (KuSaSch095).

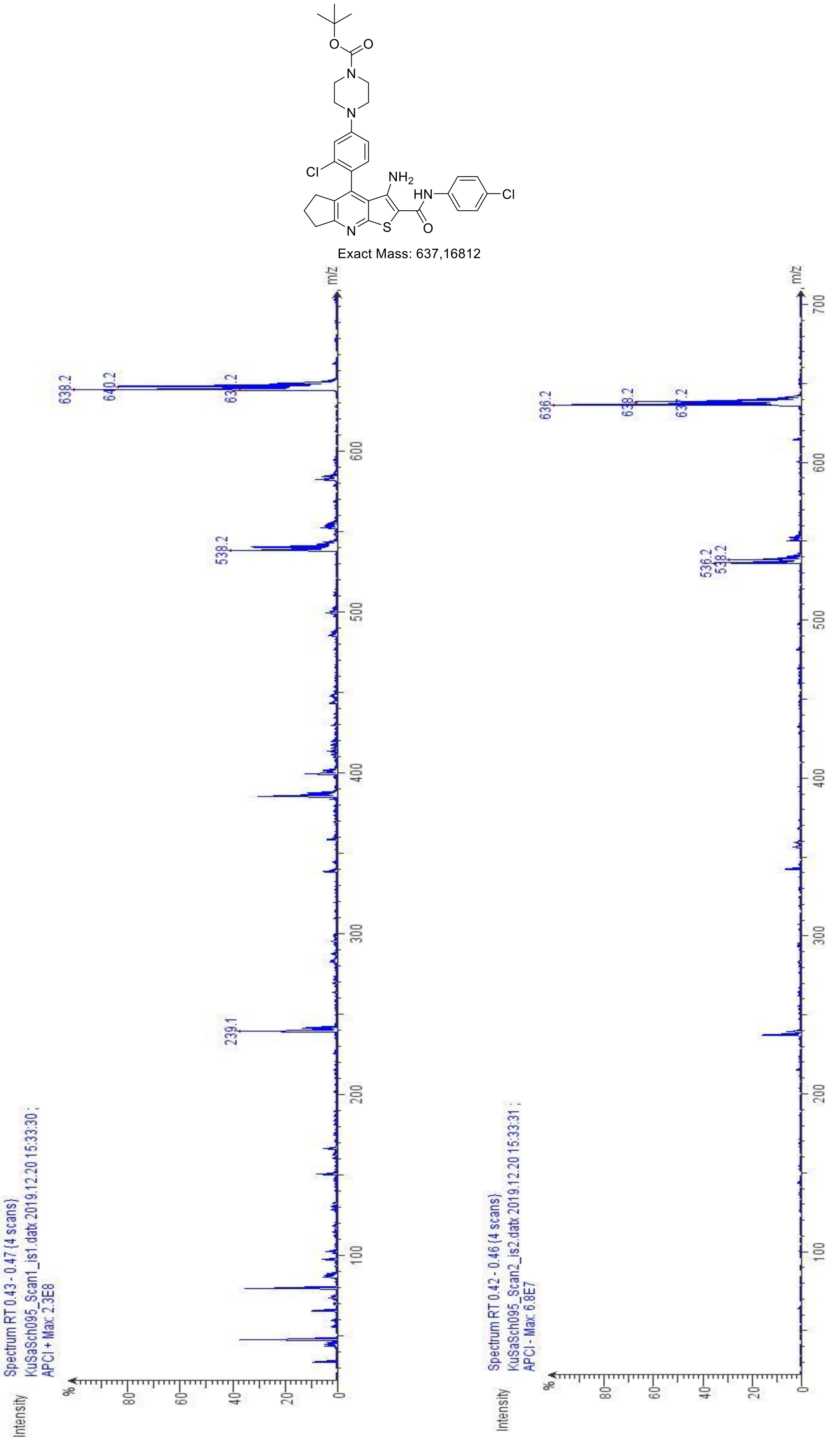

Figure S41: ^{1}H-NMR spectrum of **17b** (KuSaSch100).

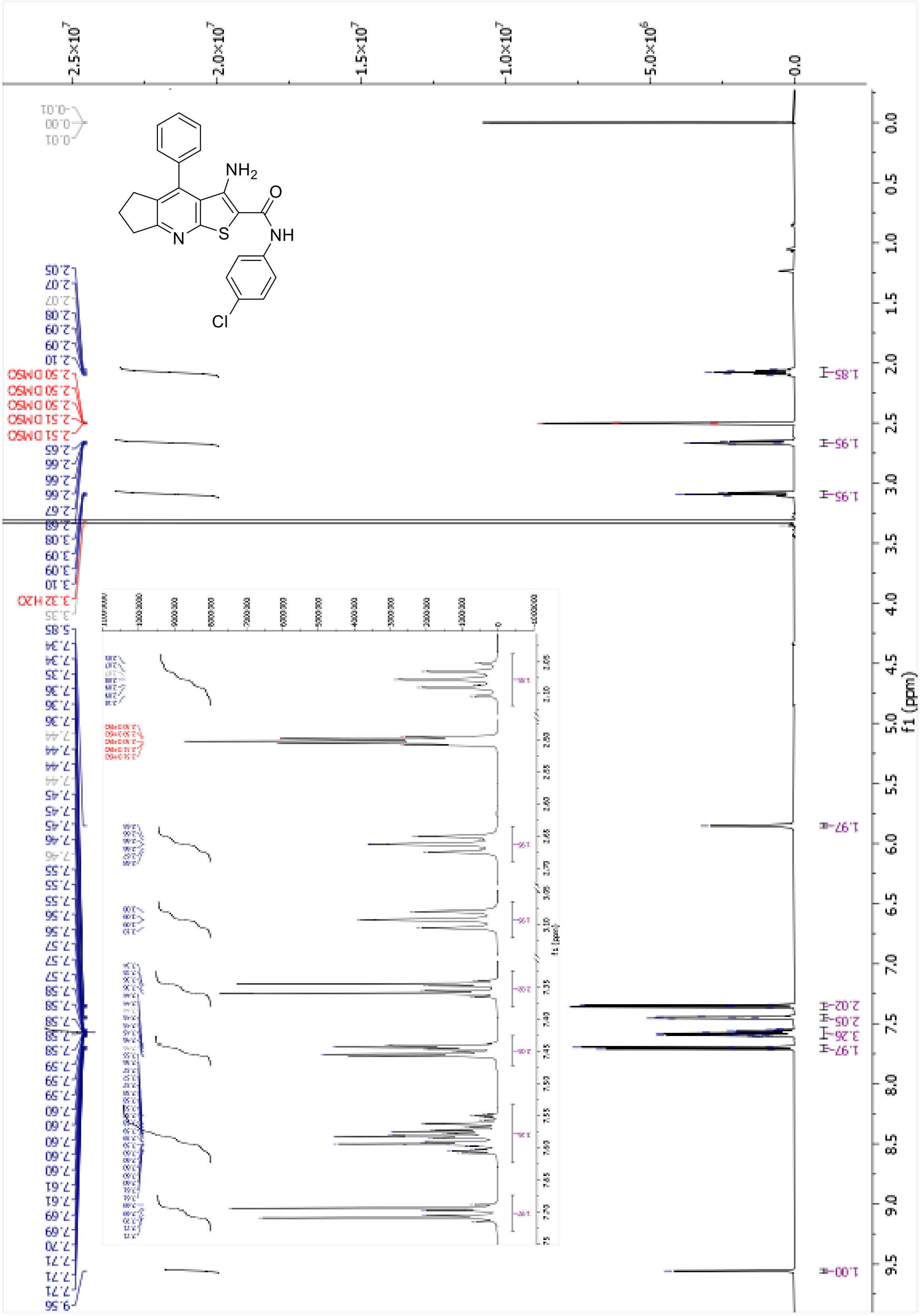

Figure S42: ^{13}C-NMR spectrum of **17b** (KuSaSch100).

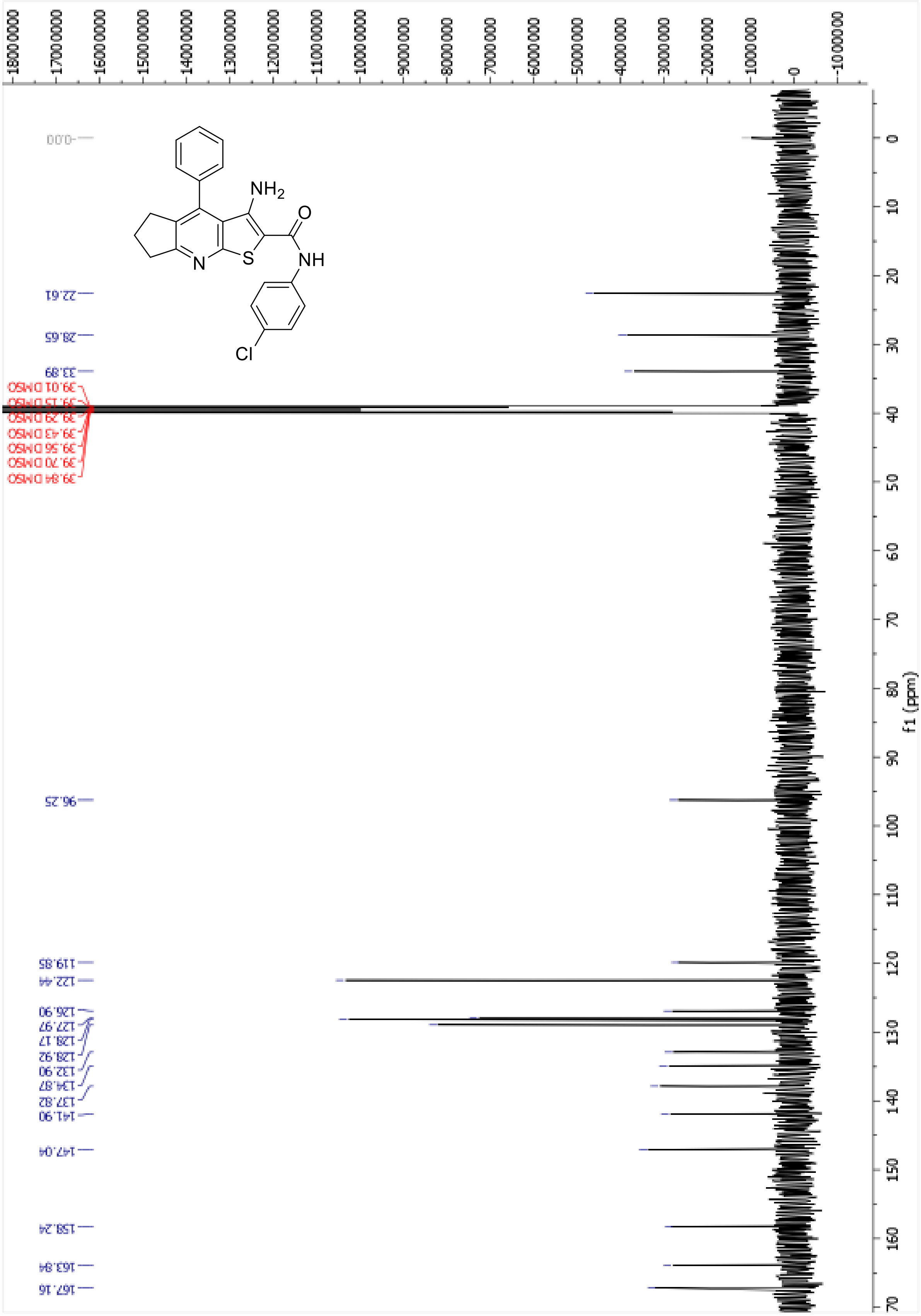

Figure S43: HSQC-NMR spectrum of **17b** (KuSaSch100). The black circle confirms 3 protons under one carbon signal.

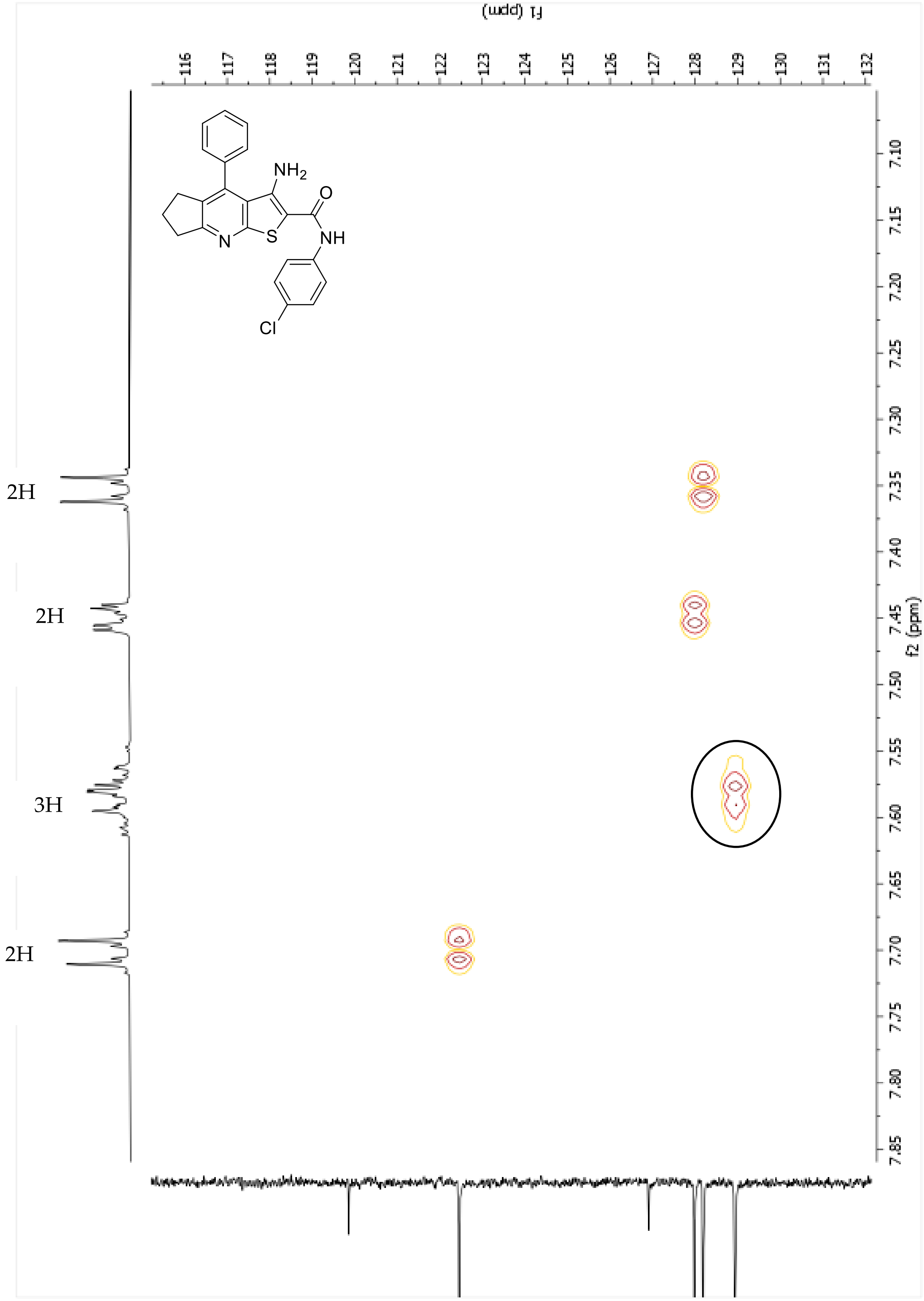

Figure S44: IR spectrum of **17b** (KuSaSch100).

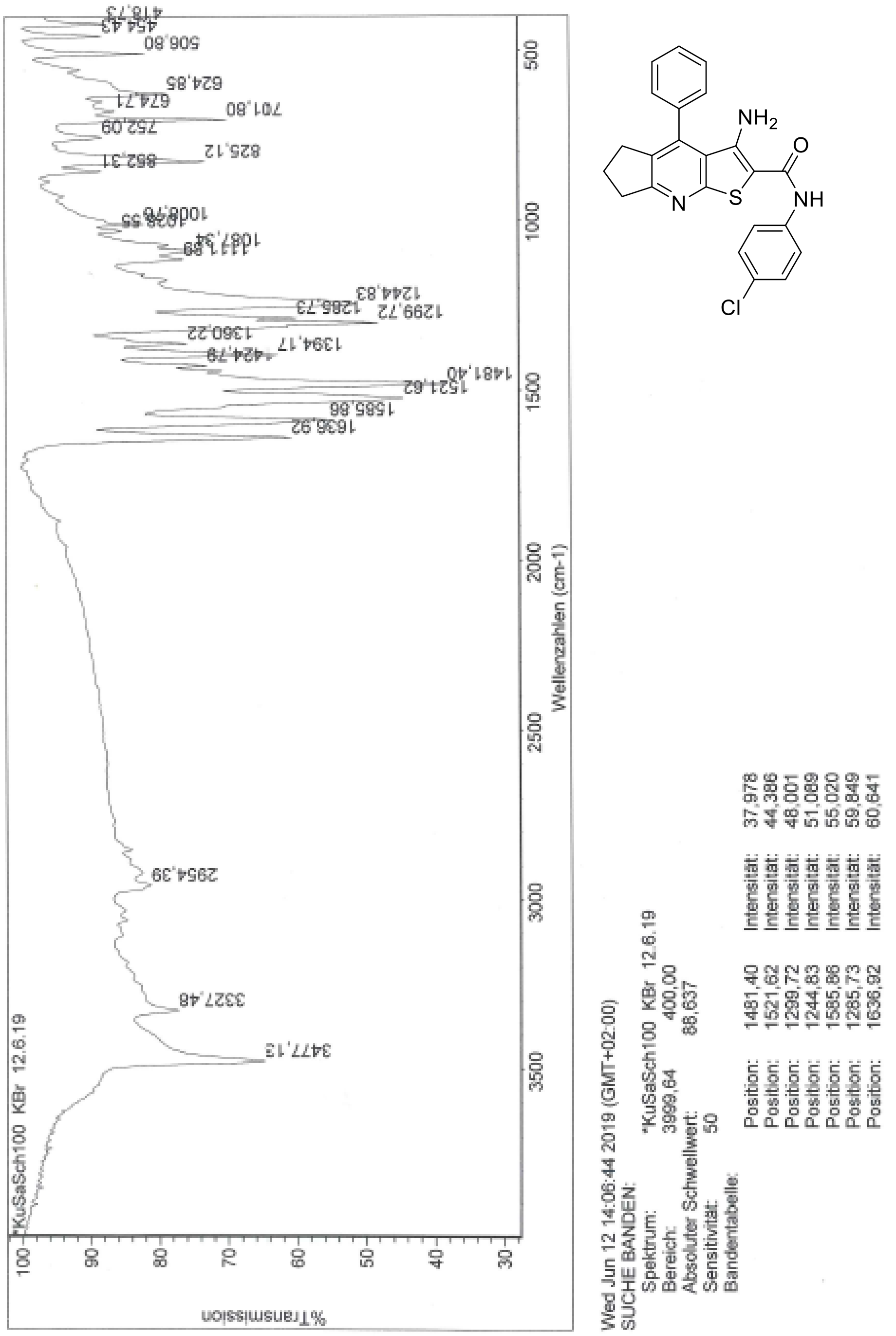

Figure S45: APCI-MS spectrum of **17b** (KuSaSch100).

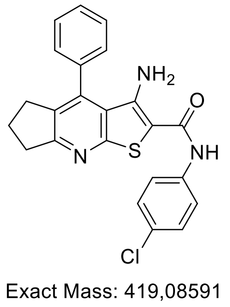

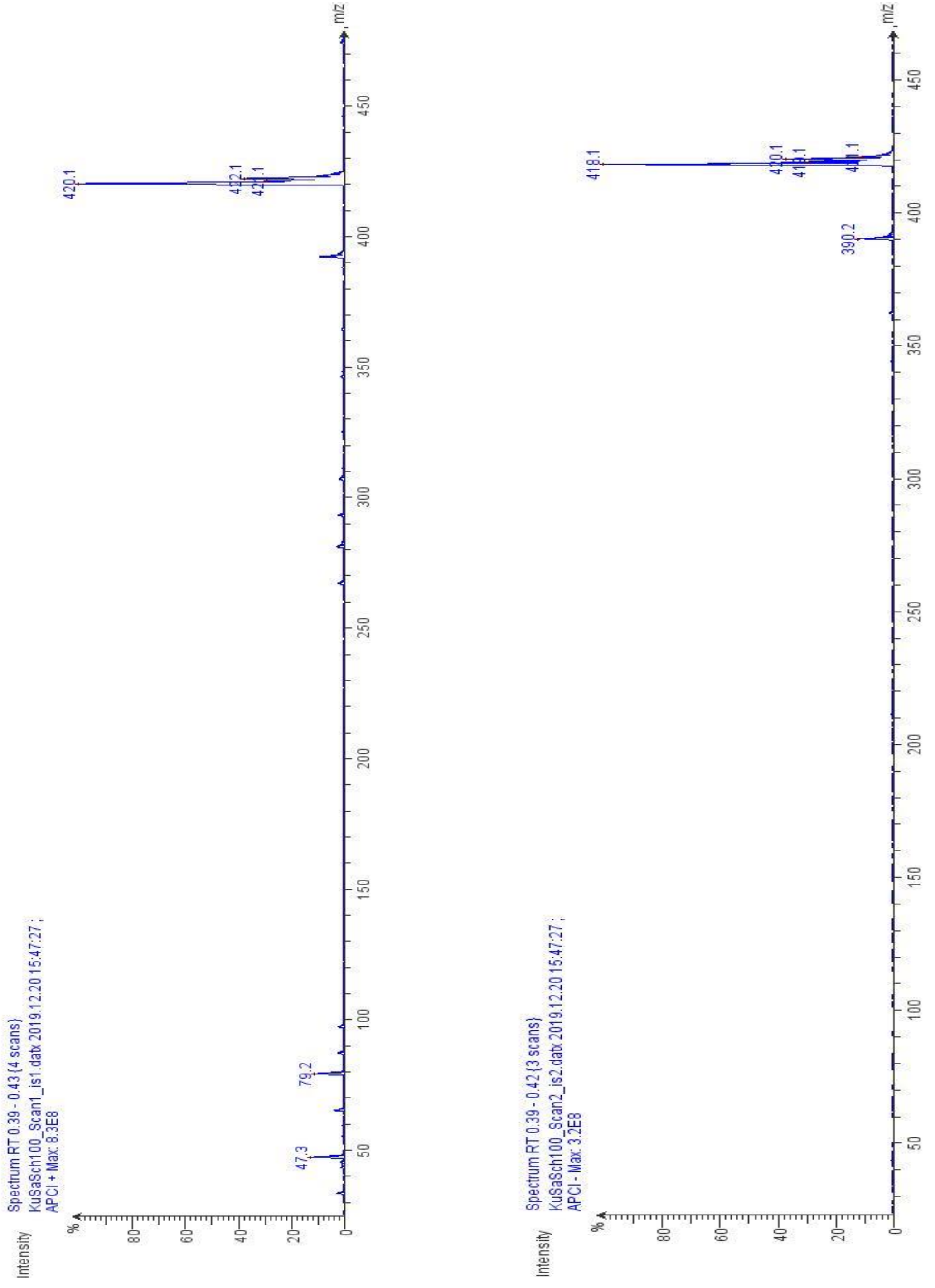

Figure S46: ^{1}H-NMR spectrum of **17e** (KuSaSch107).

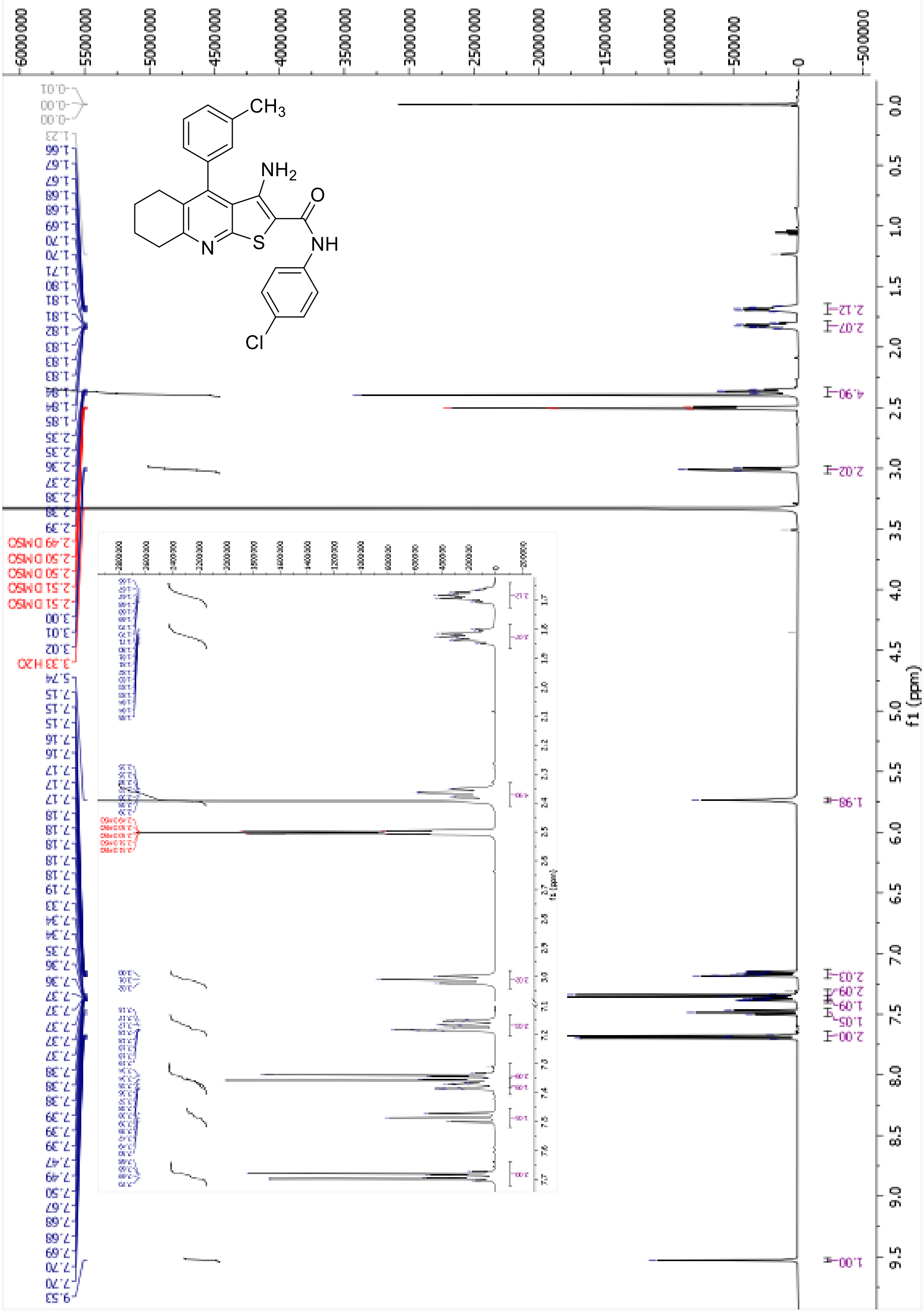

Figure S47: ^{13}C-NMR spectrum of **17e** (KuSaSch107).

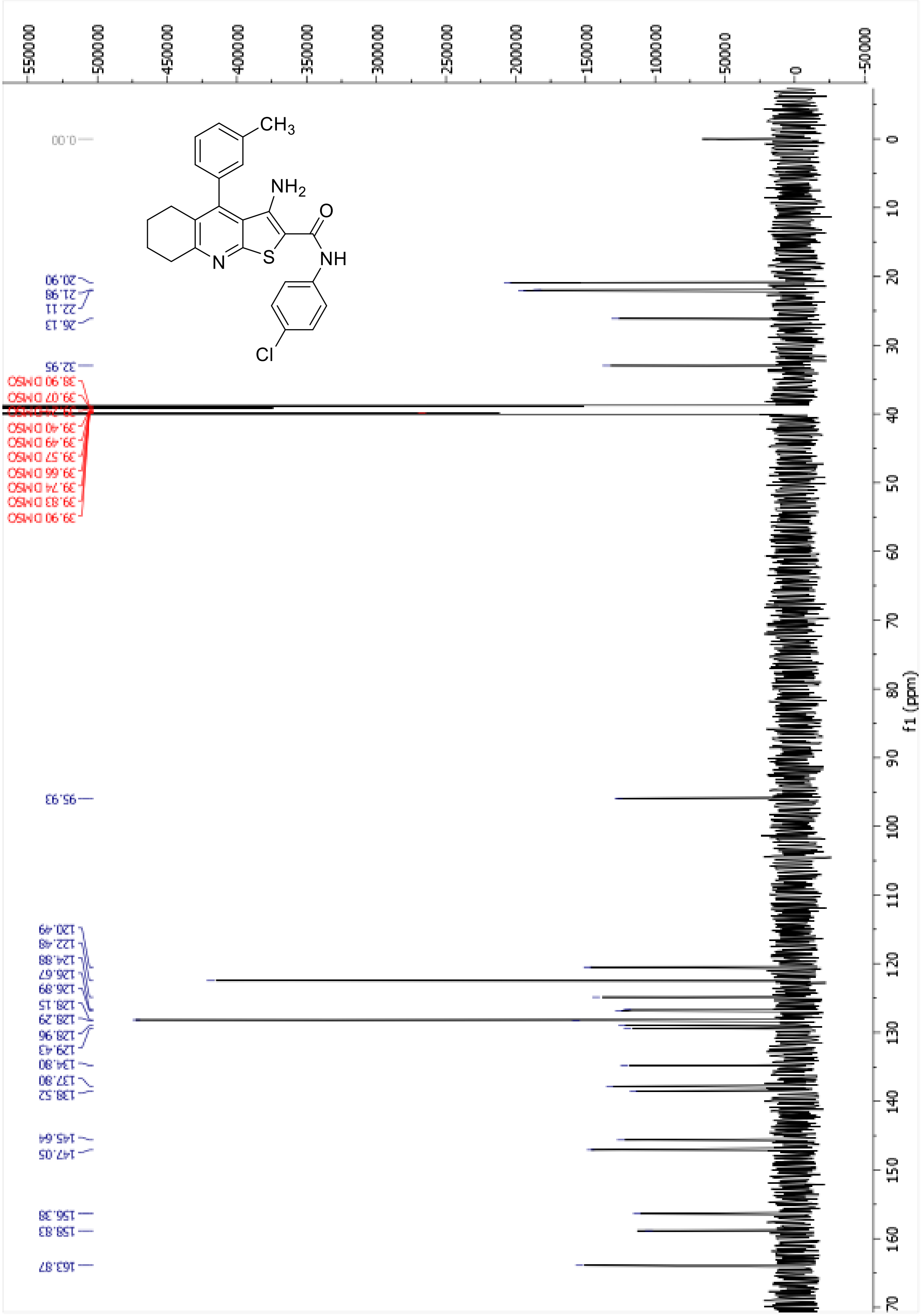

Figure S48: IR spectrum of **17e** (KuSaSch107).

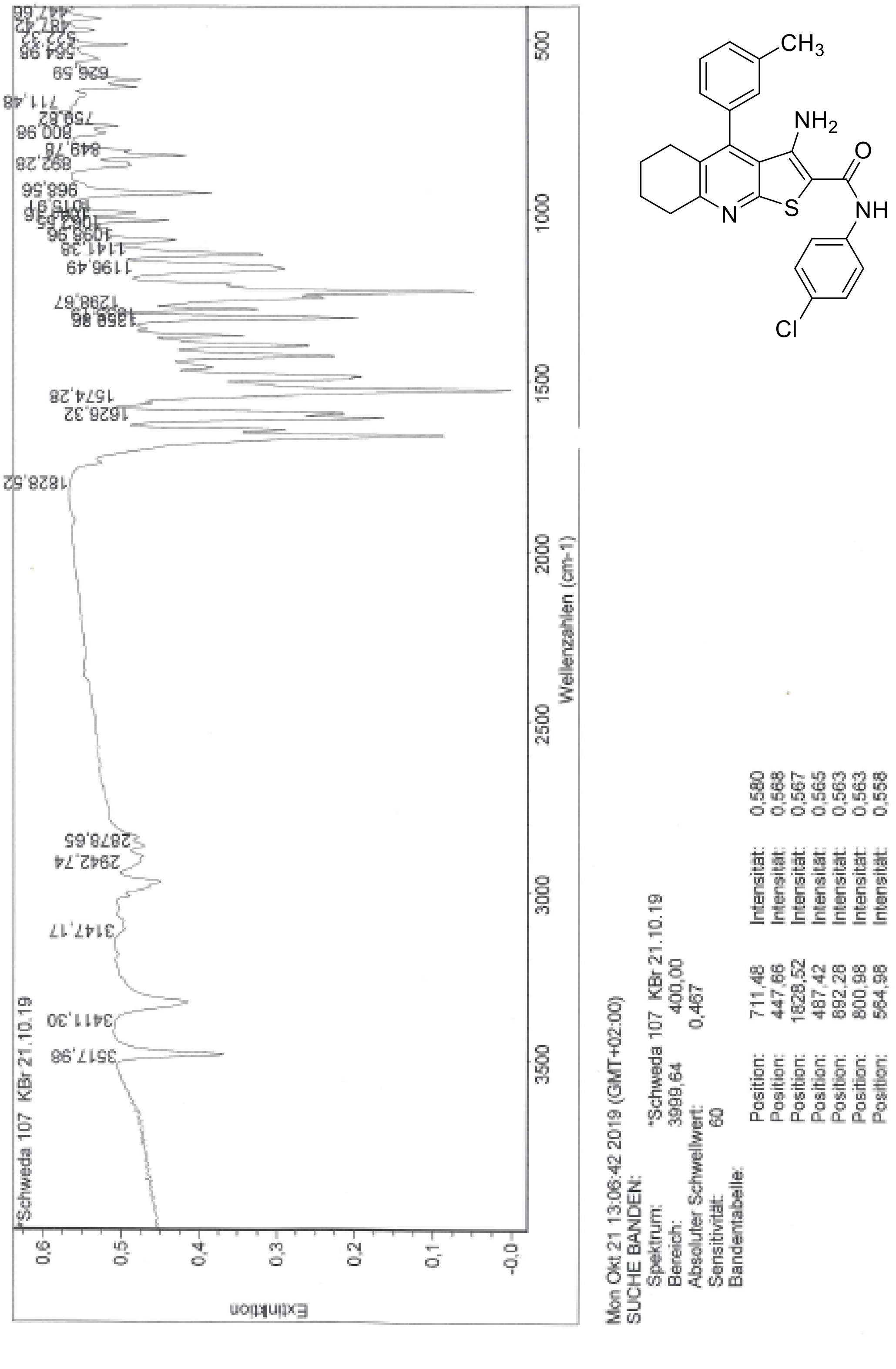

Figure S49: APCI-MS spectrum of **17e** (KuSaSch107).

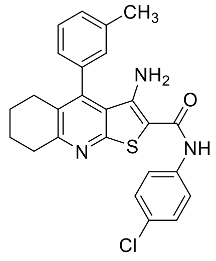

Exact Mass: 447,11721

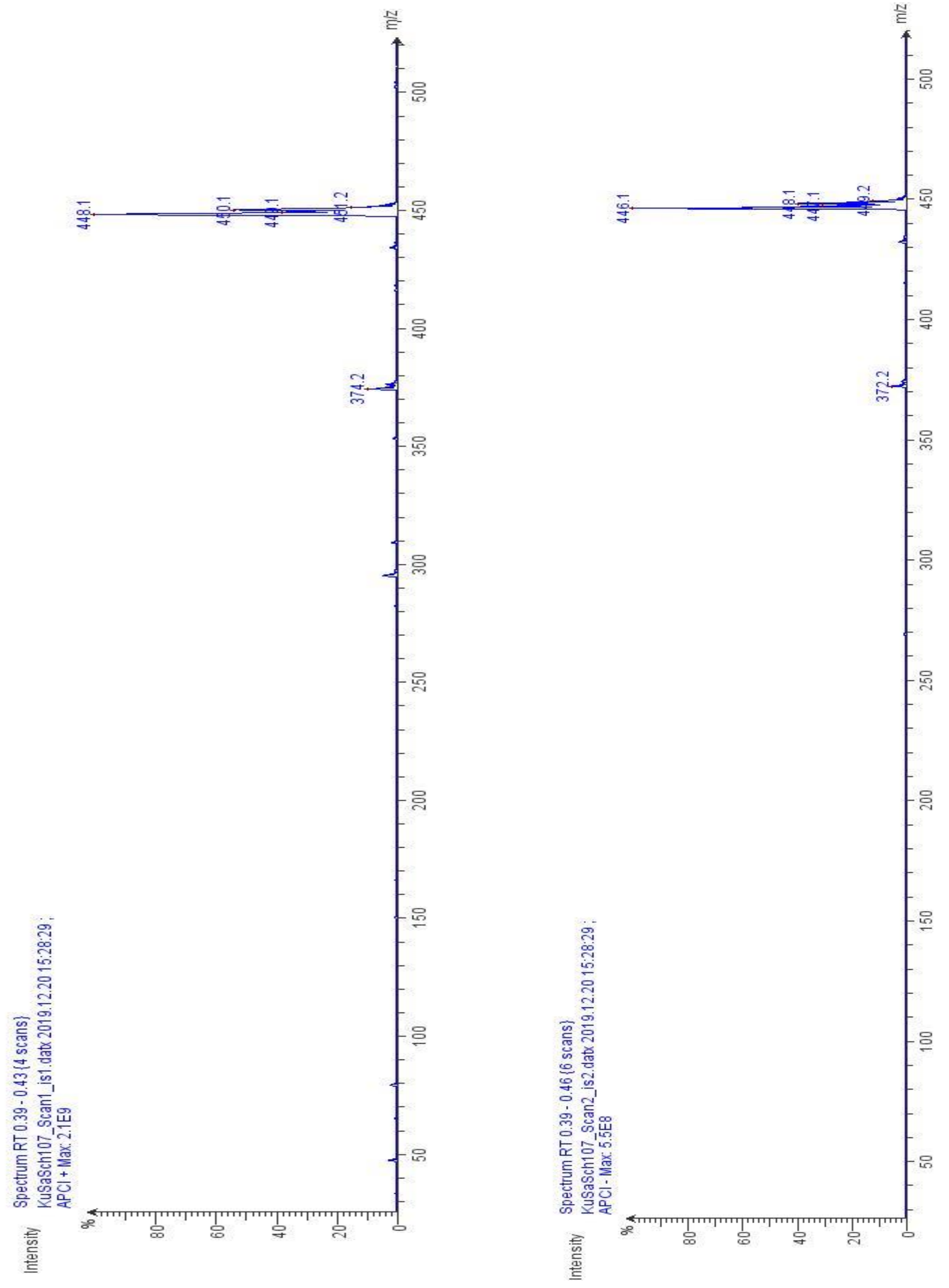

Figure S50: ^{1}H-NMR spectrum of **17f** (KuSaSch110).

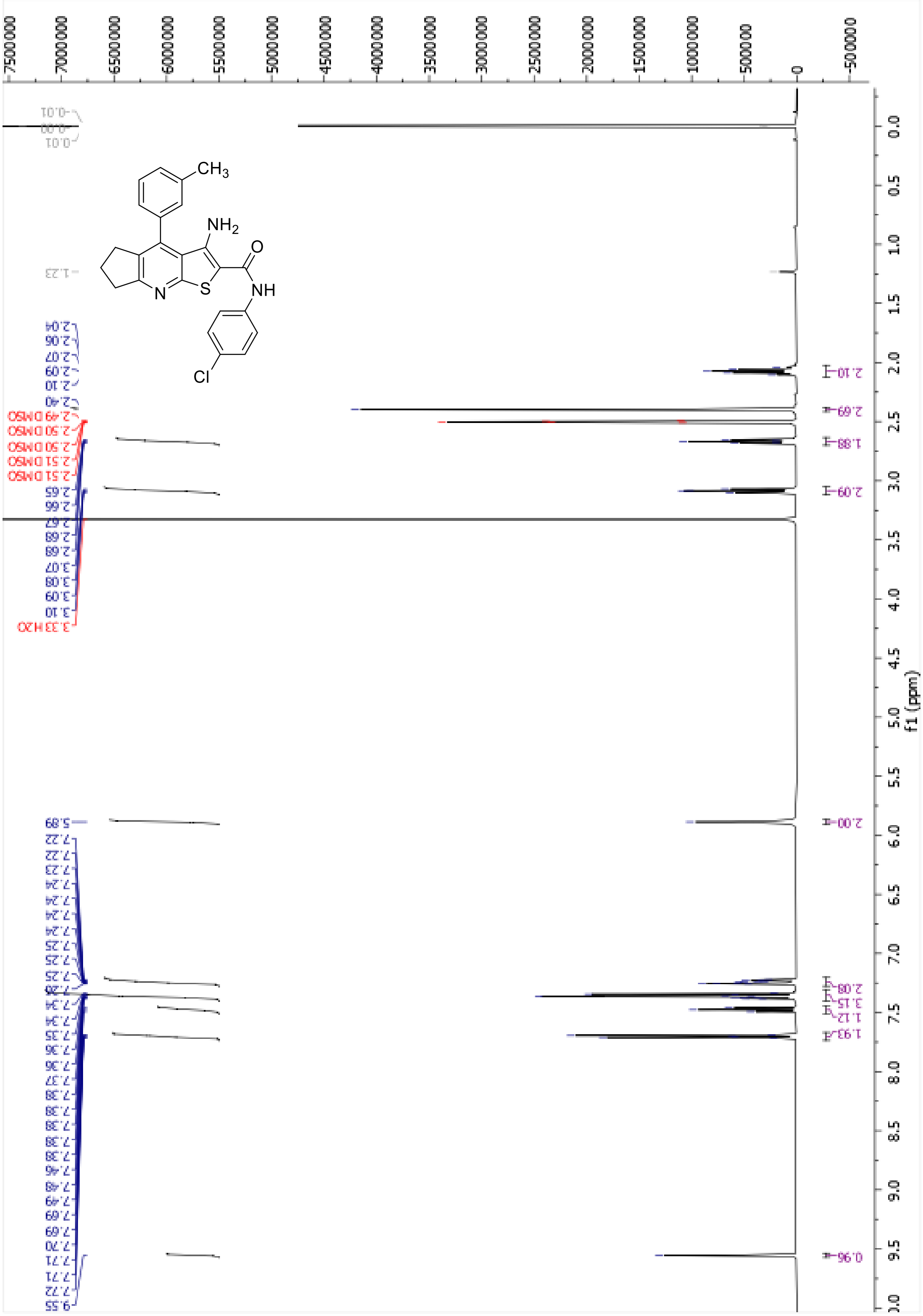

Figure S51: ^{13}C-NMR spectrum of **17f** (KuSaSch110).

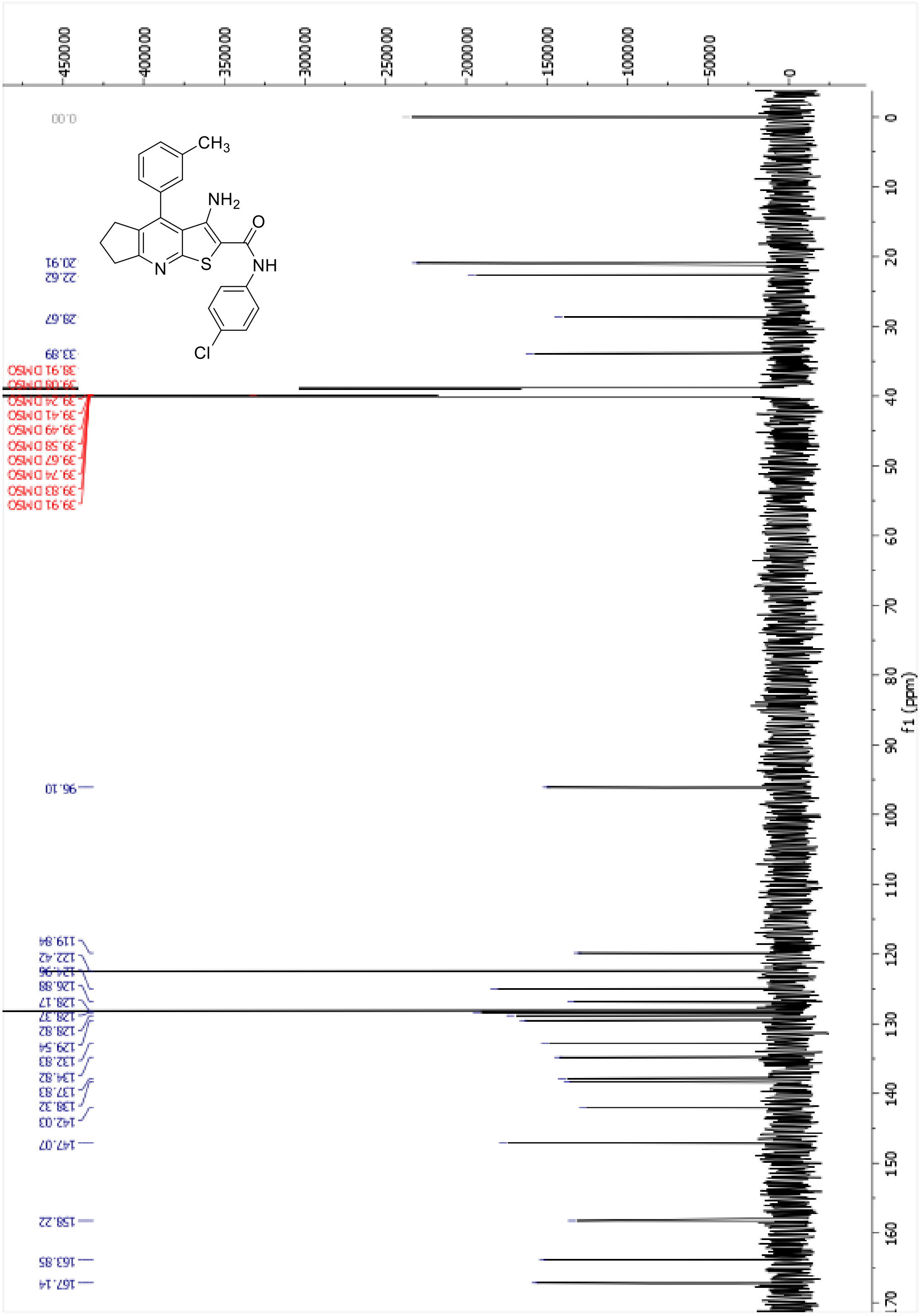

Figure S52: IR spectrum of **17f** (KuSaSch110).

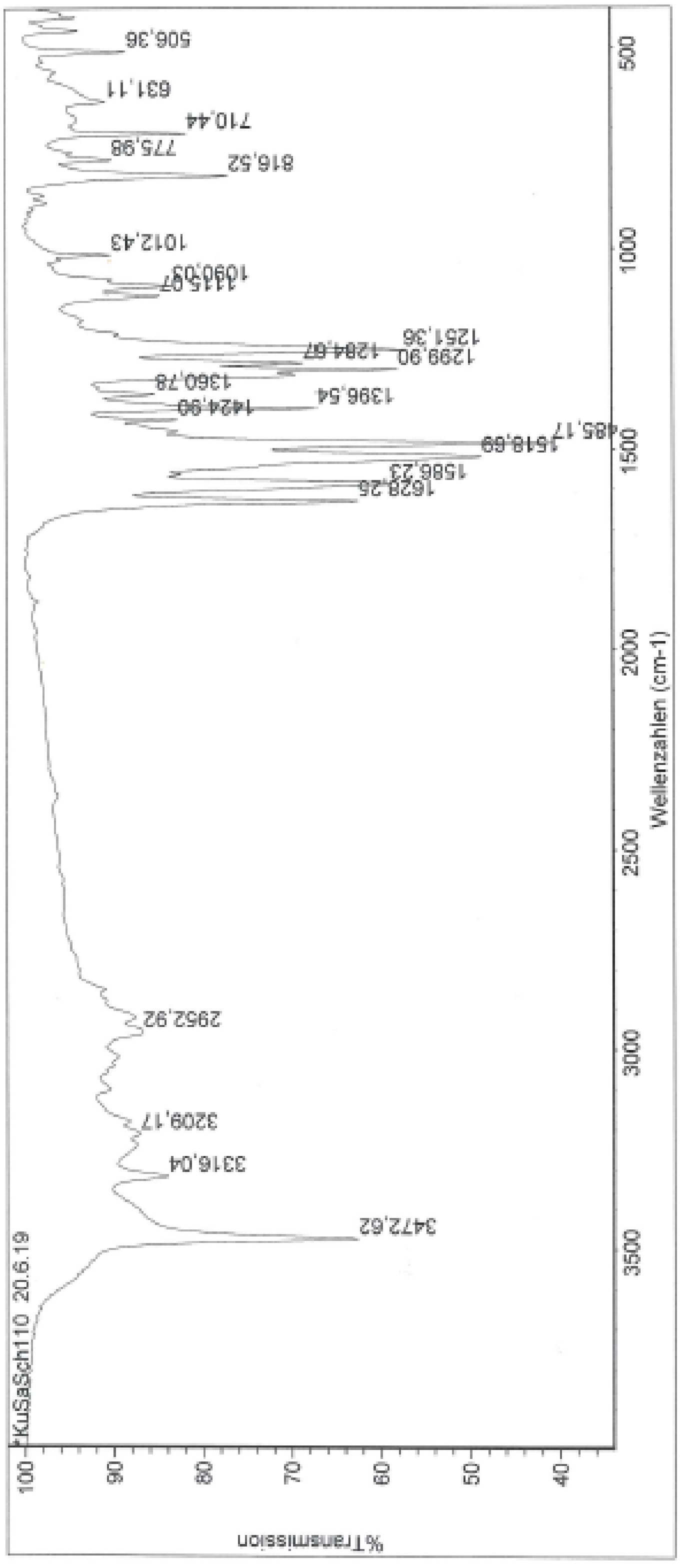

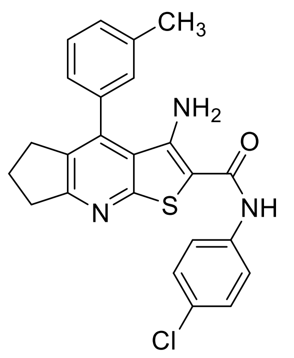

Thu Jun 20 11:21:46 2019 (GMT+02:00)
SUCHE BANDEN:
Spektrum: *KuSaSch110 20.6.19
Bereich: 3999,64 400,00
Absoluter Schwellwert: 91,017
Sensitivität: 50
Bandentabelle:

Position:	1485,17	Intensität:	40,677
Position:	1518,69	Intensität:	48,530
Position:	1251,36	Intensität:	57,328
Position:	1299,90	Intensität:	57,912
Position:	1586,23	Intensität:	58,842
Position:	1628,25	Intensität:	62,427
Position:	3472,62	Intensität:	62,577

Figure S53: APCI-MS spectrum of **17f** (KuSaSch110).

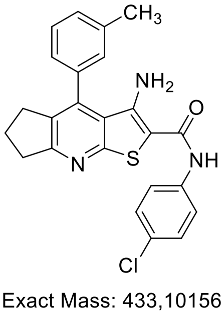

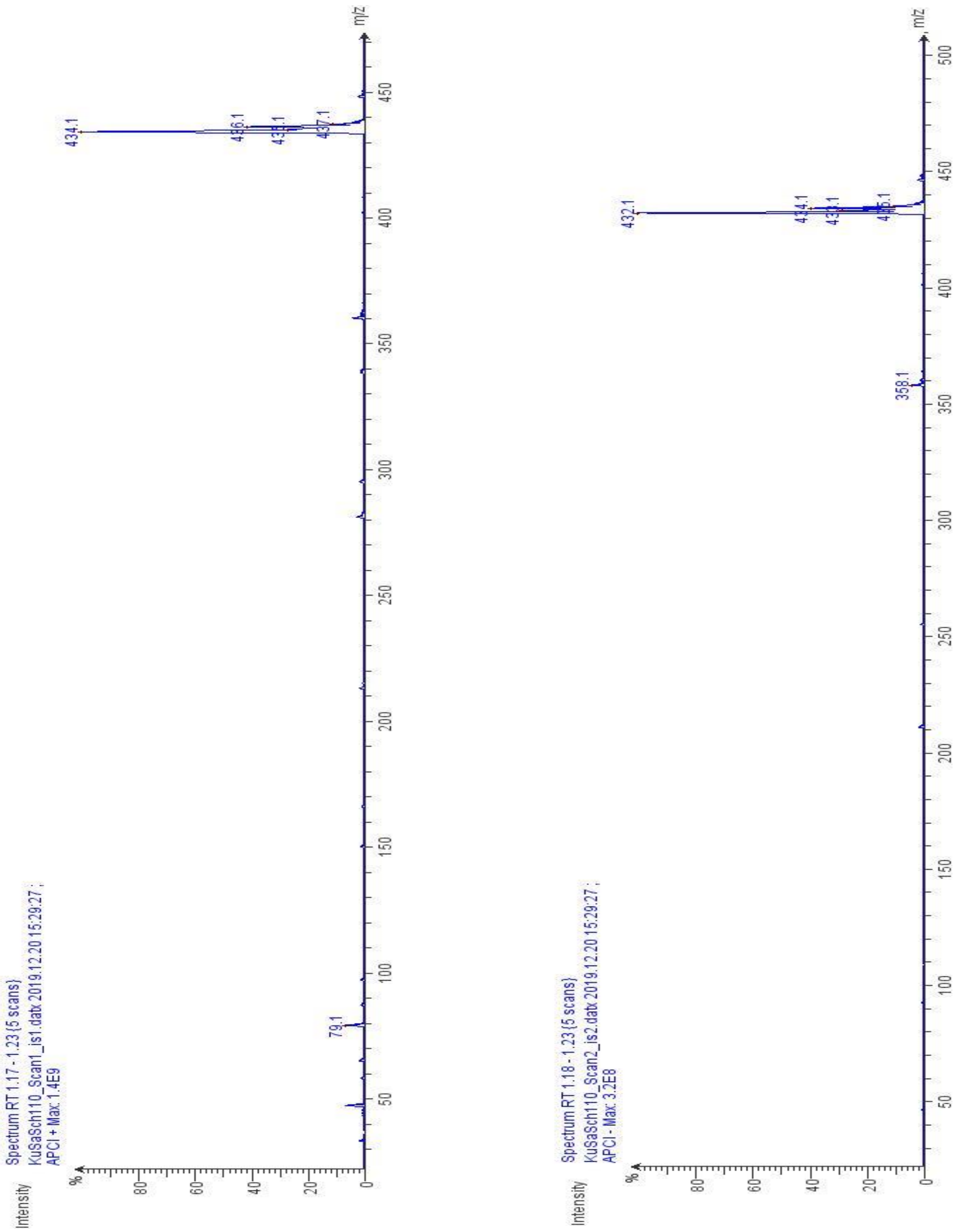

Figure S54: ^{1}H-NMR spectrum of **17g** (KuSaSch111).

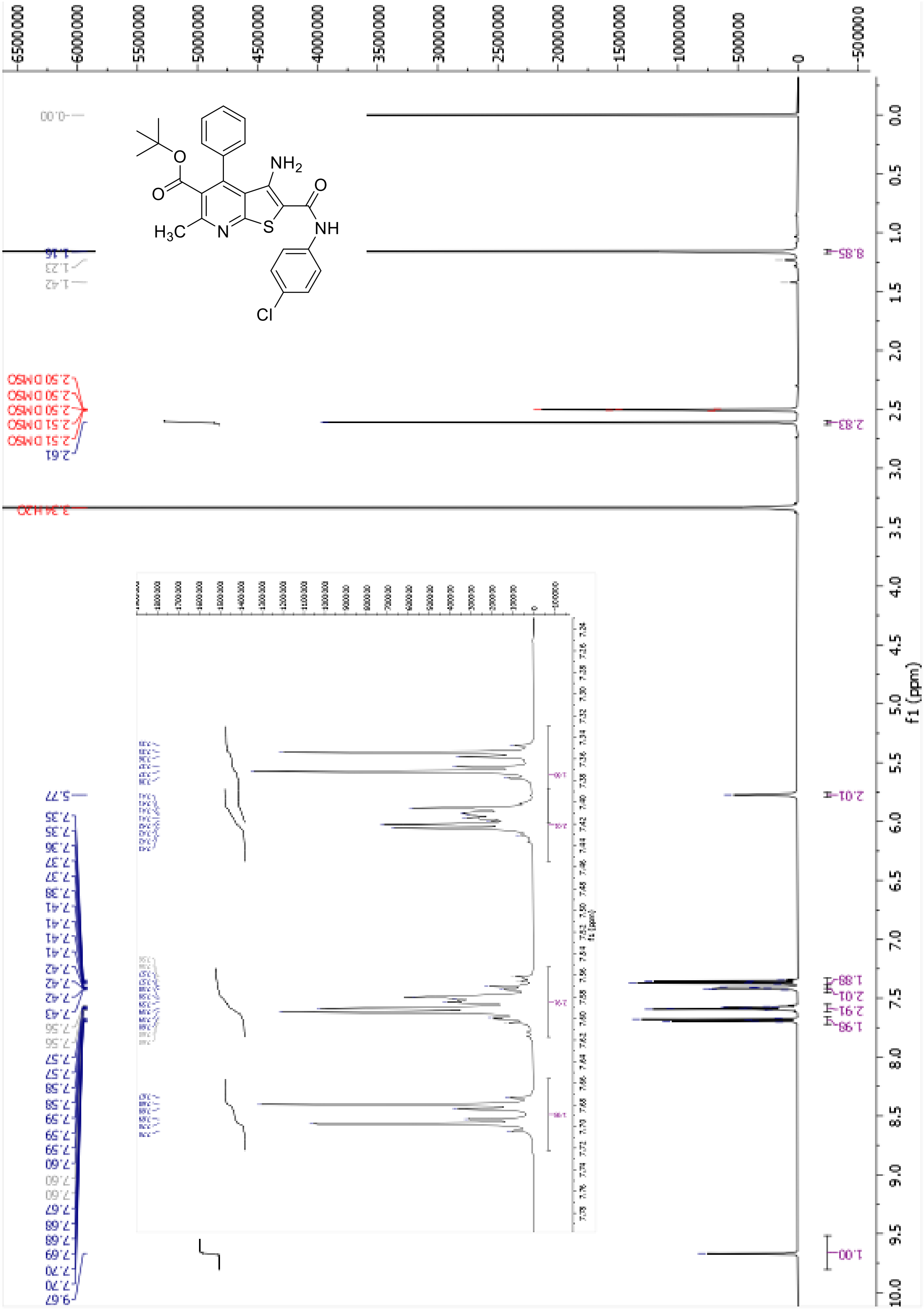

Figure S55: ^{13}C-NMR spectrum of **17f** (KuSaSch111).

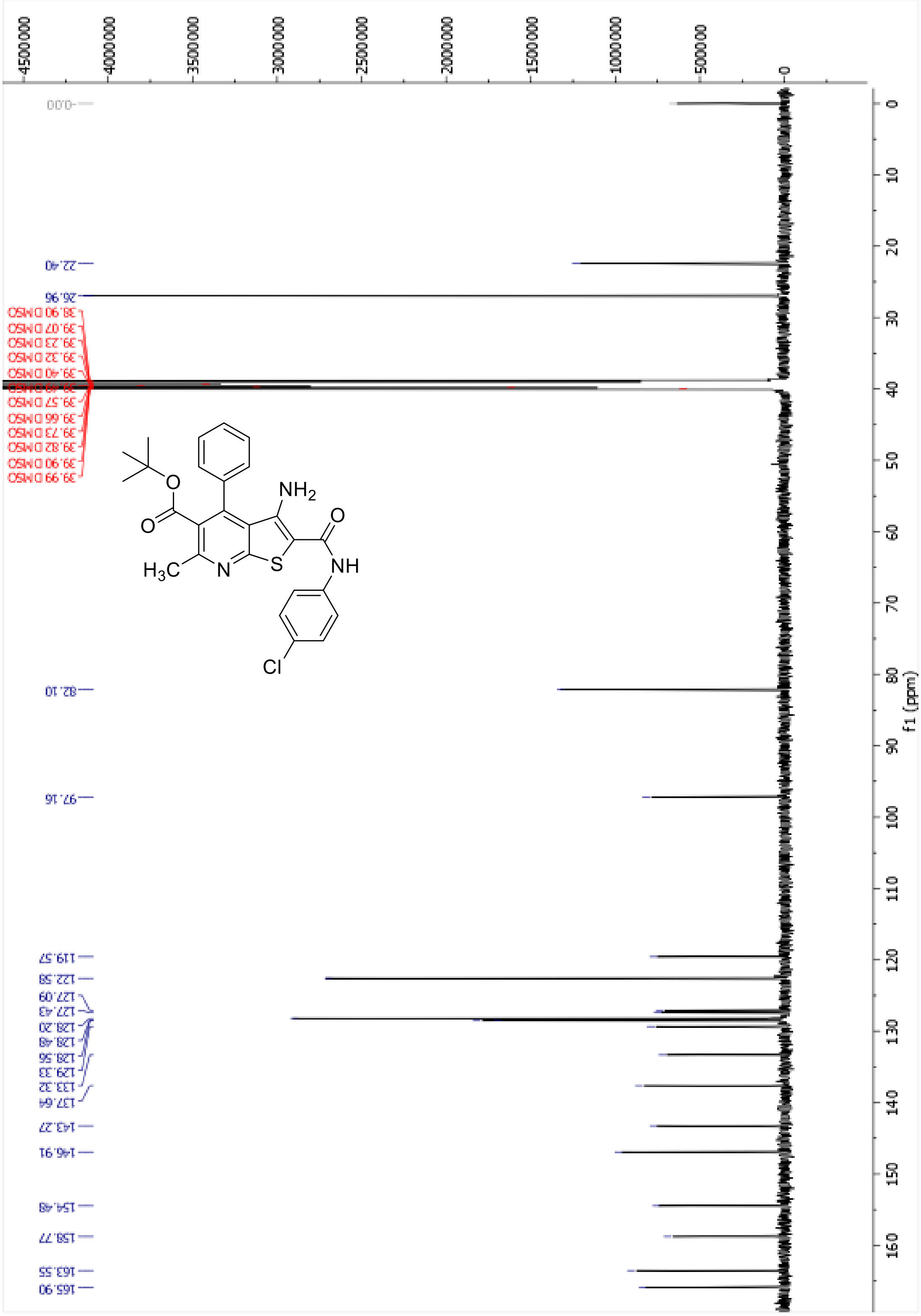

Figure S56: IR spectrum of **17f** (KuSaSch111).

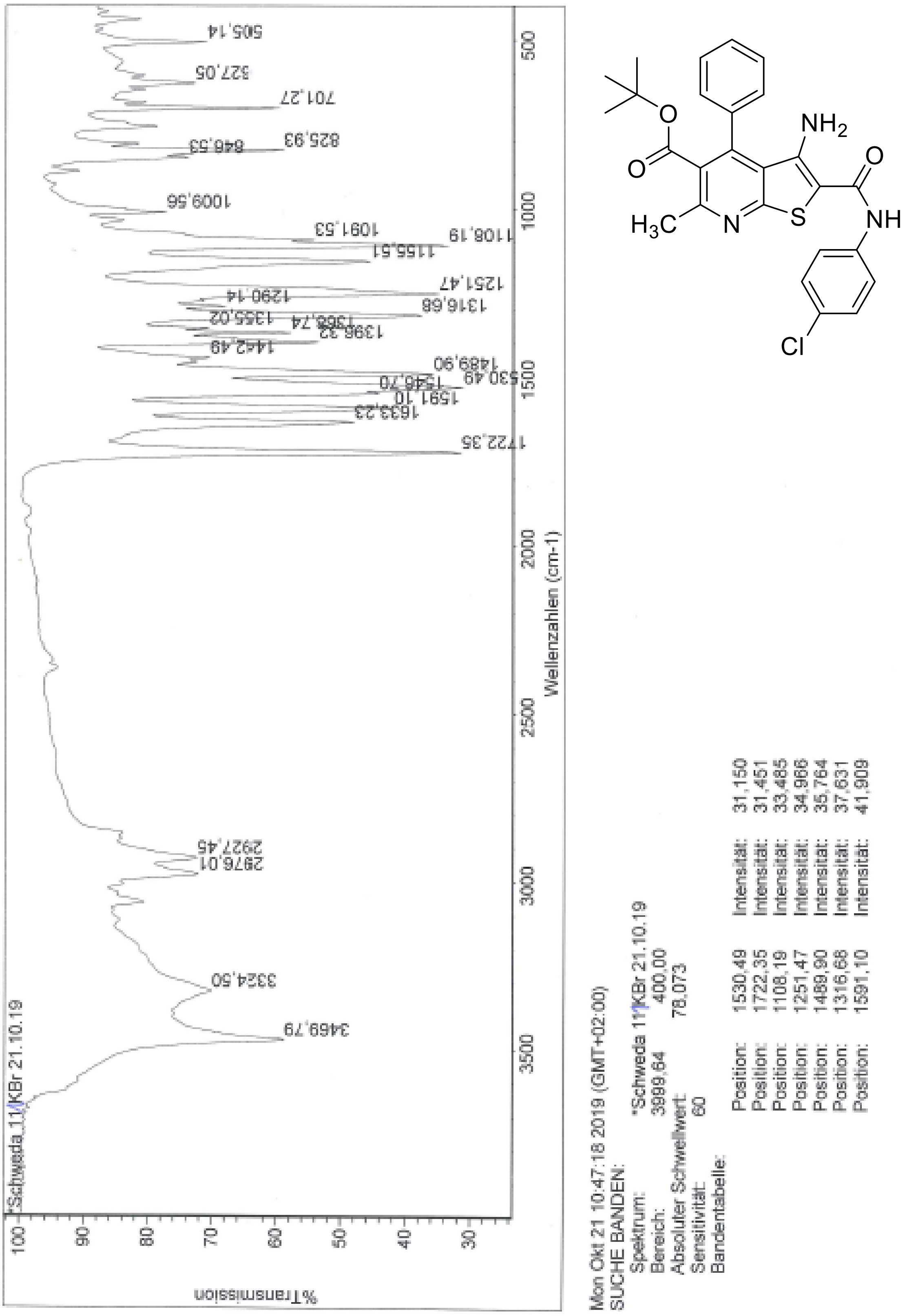

Figure S57: APCI-MS spectrum of **17f** (KuSaSch111).

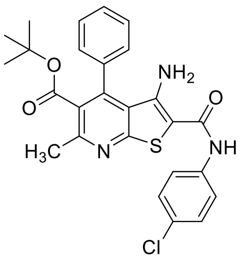

Exact Mass: 493,12269

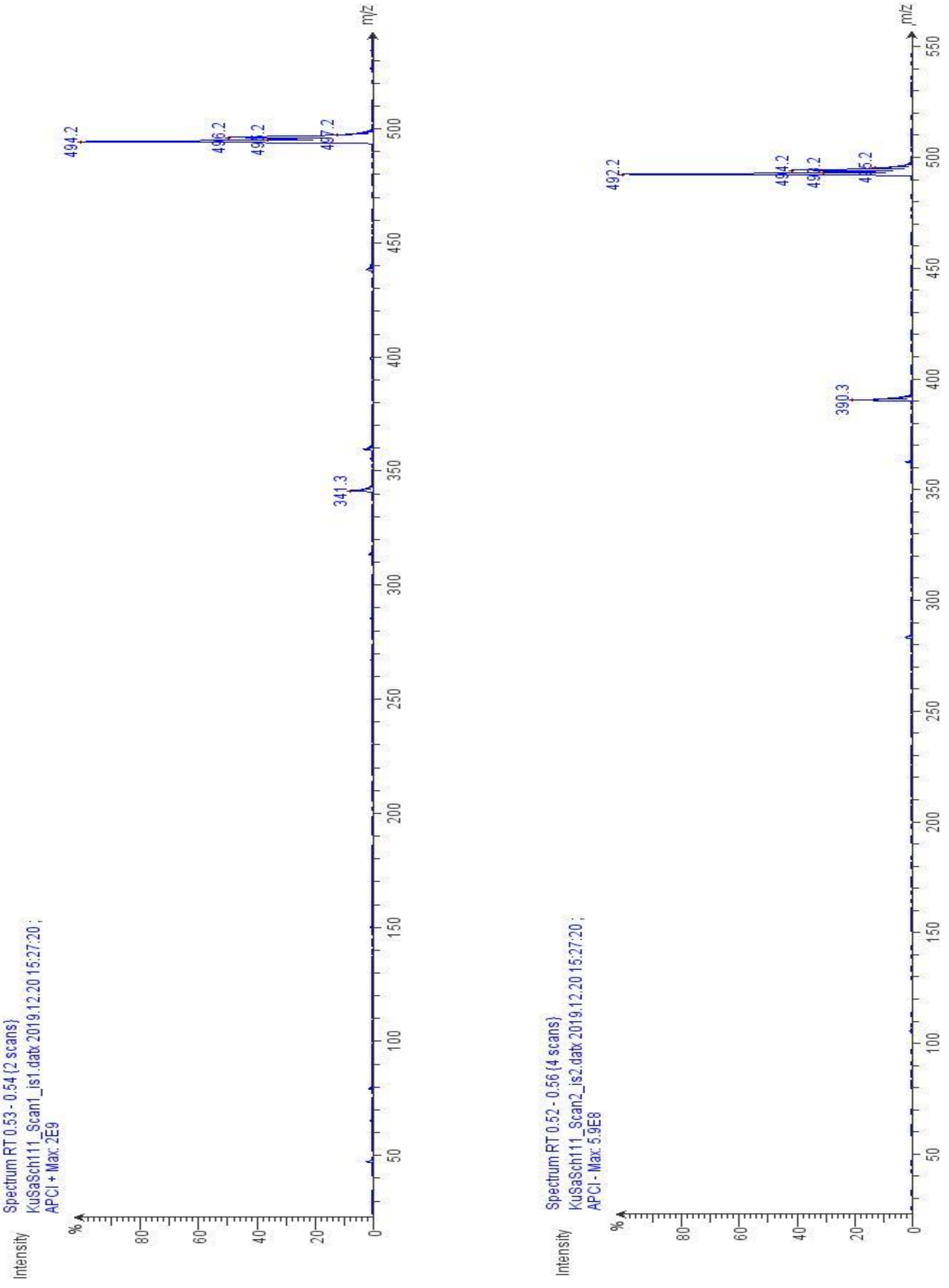

Figure S58: ^{1}H-NMR spectrum of **17h** (KuSaSch112).

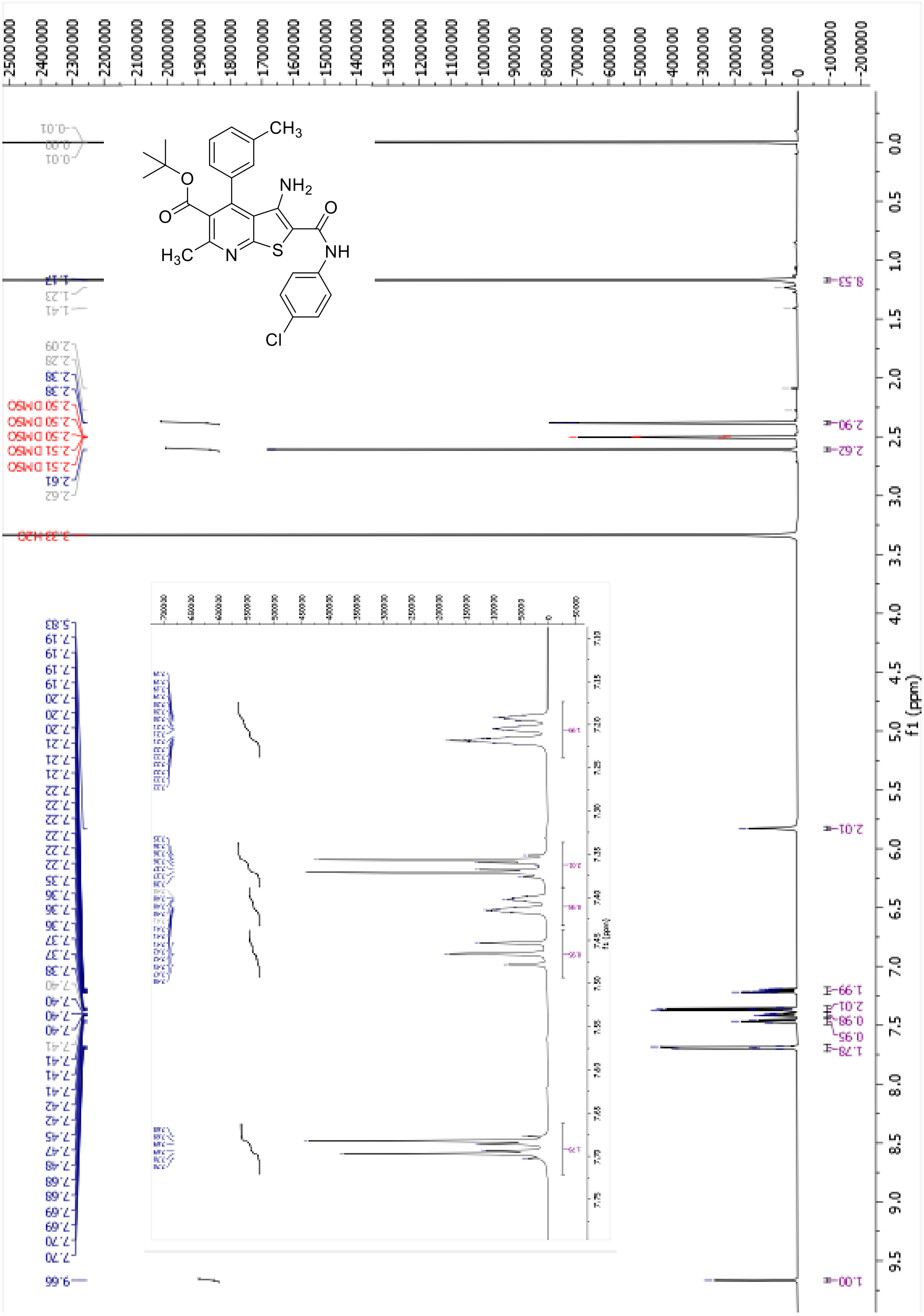

Figure S59: ^{13}C-NMR spectrum of **17h** (KuSaSch112).

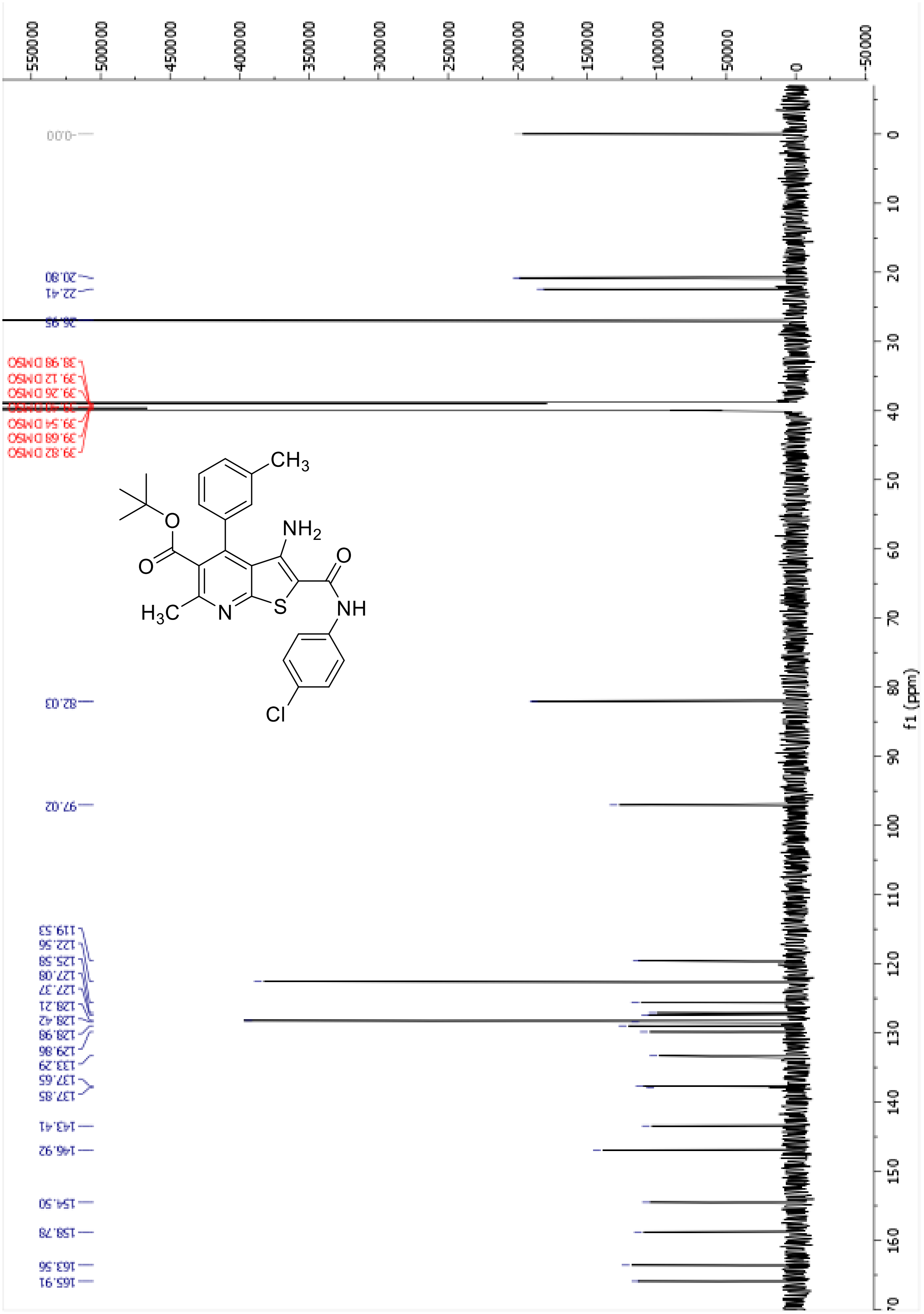

Figure S60: IR spectrum of **17h** (KuSaSch112).

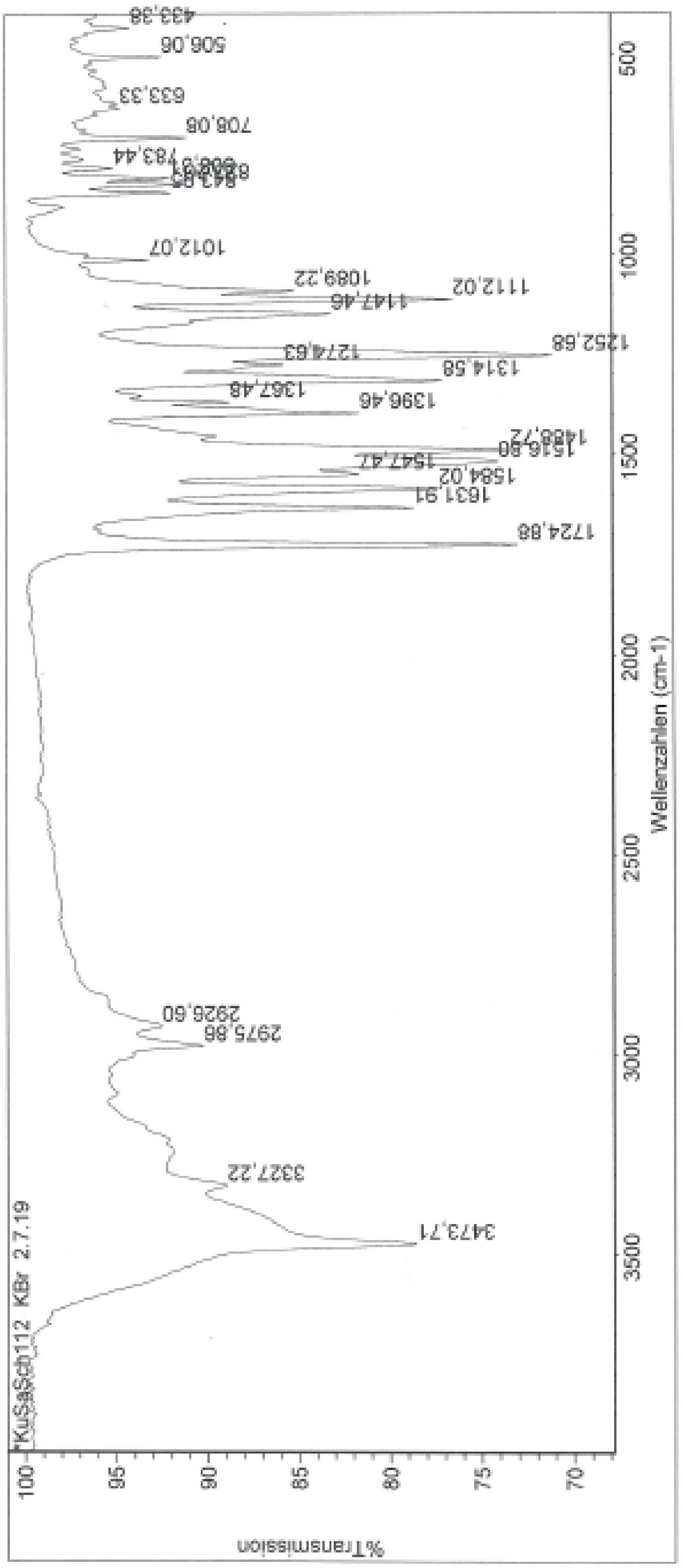

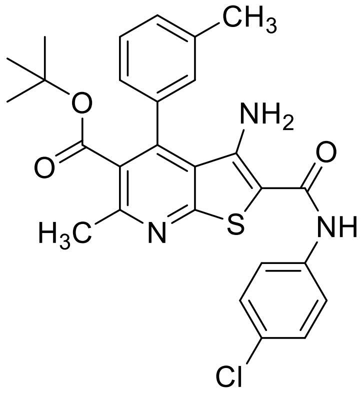

Figure S61: APCI-MS spectrum of **17h** (KuSaSch112).

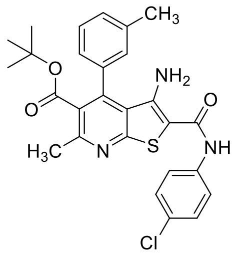

Exact Mass: 507,13834

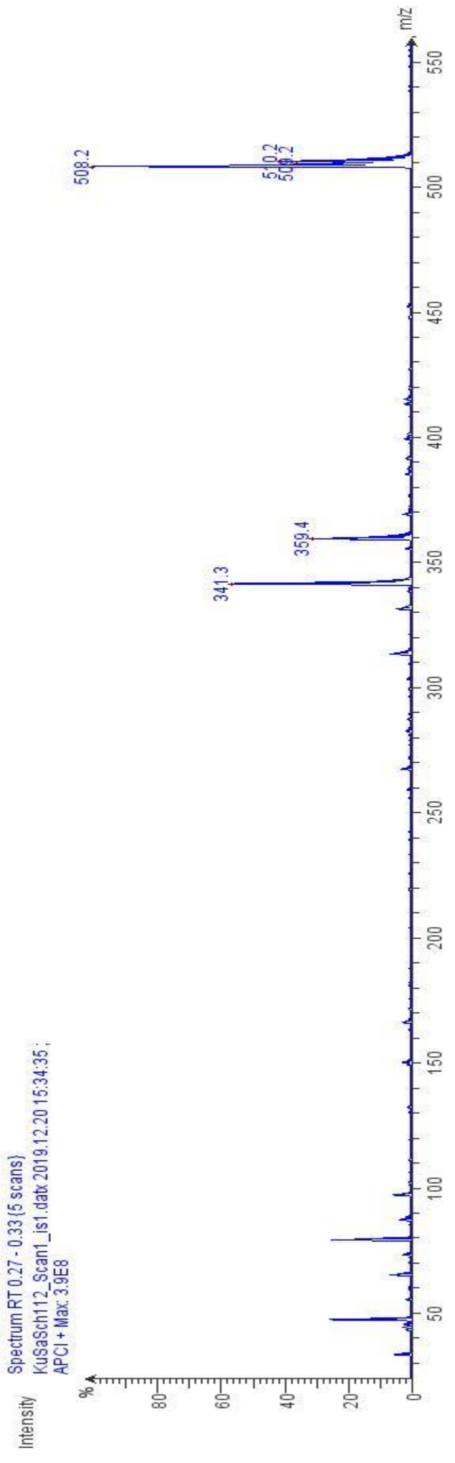

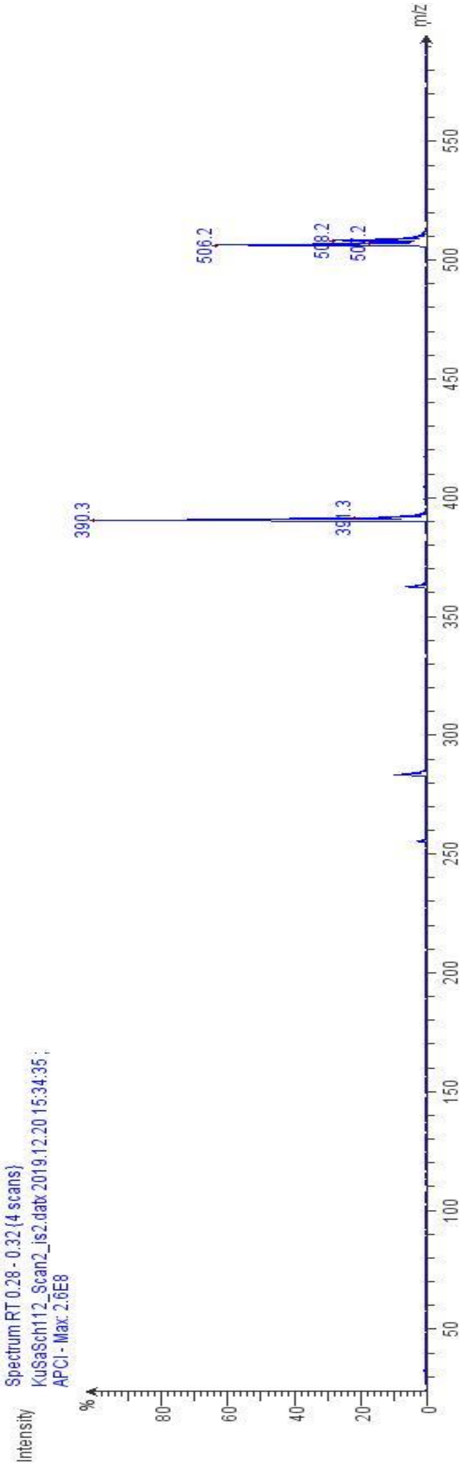

Figure S62: HPLC chromatogram of **9a** (KuSaSch018) – gradient method (HPLC 3)

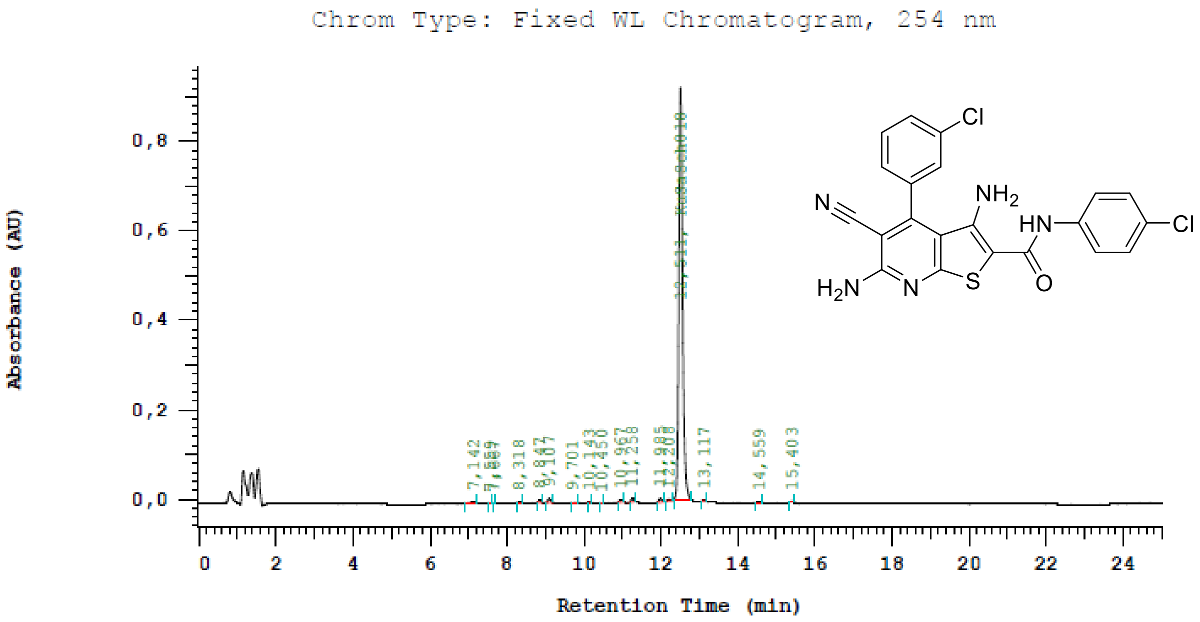

Figure S63: HPLC chromatogram of **9a** (KuSaSch018) – isocratic method (HPLC 1)

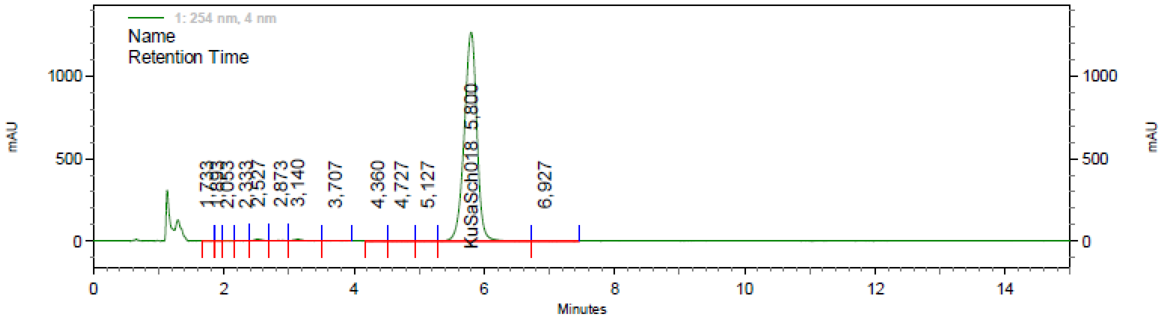

Figure S64: HPLC chromatogram of **9e** (KuSaSch031) – gradient method (HPLC 3)

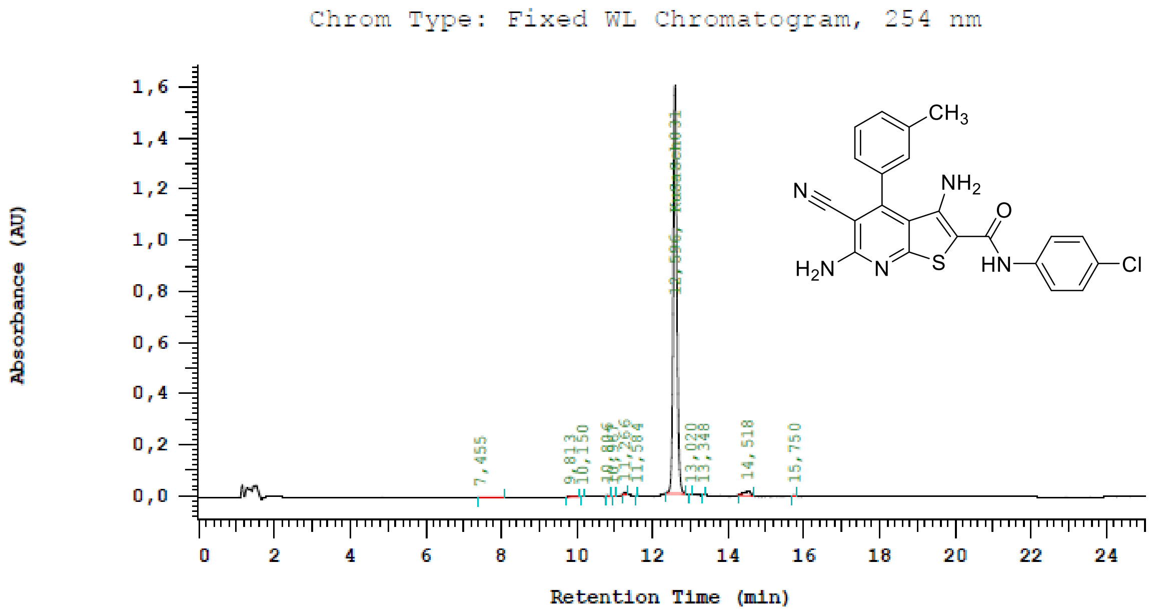

Figure S65: HPLC chromatogram of **9e** (KuSaSch031) – isocratic method (HPLC 1)

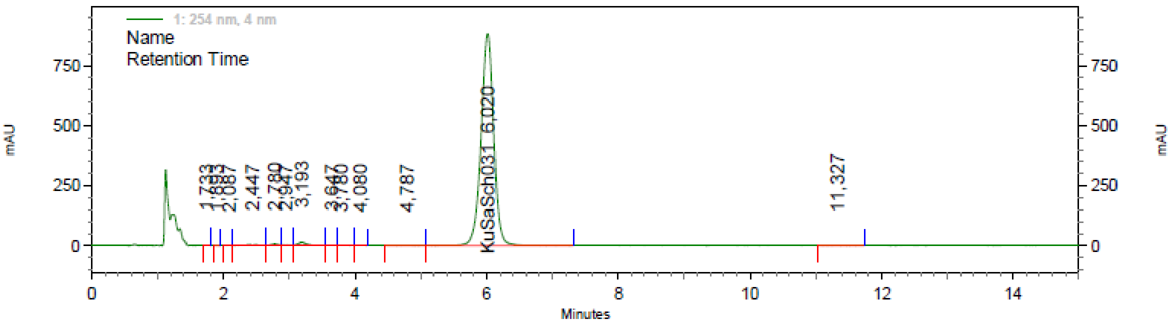

Figure S66: HPLC chromatogram of **9m** (KuSaSch134) – gradient method (HPLC 3)

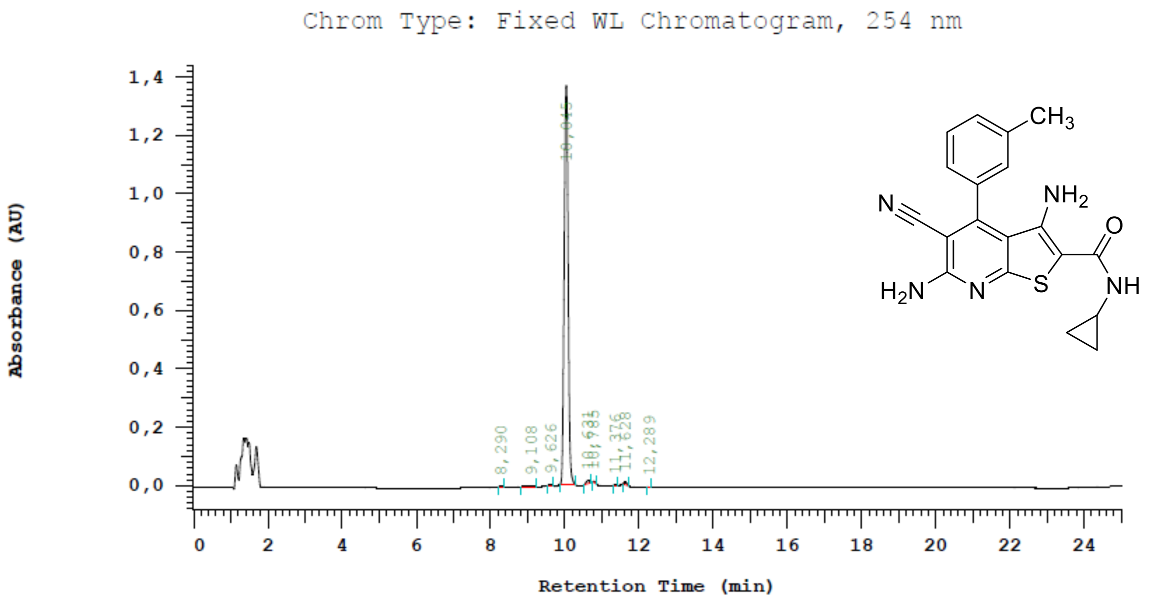

Figure S67: HPLC chromatogram of **9m** (KuSaSch134) – isocratic method (HPLC 1)

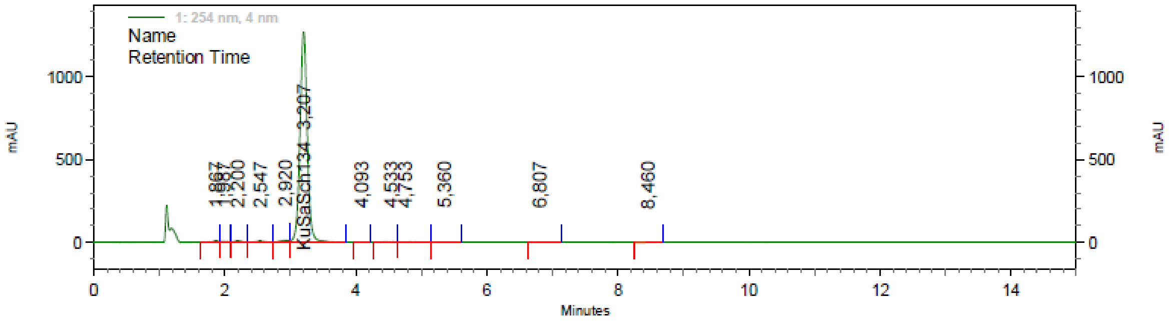

1: 254 nm. 4 nm

Figure S68: HPLC chromatogram of **9y** (KuSaSch060) – gradient method (HPLC 3)

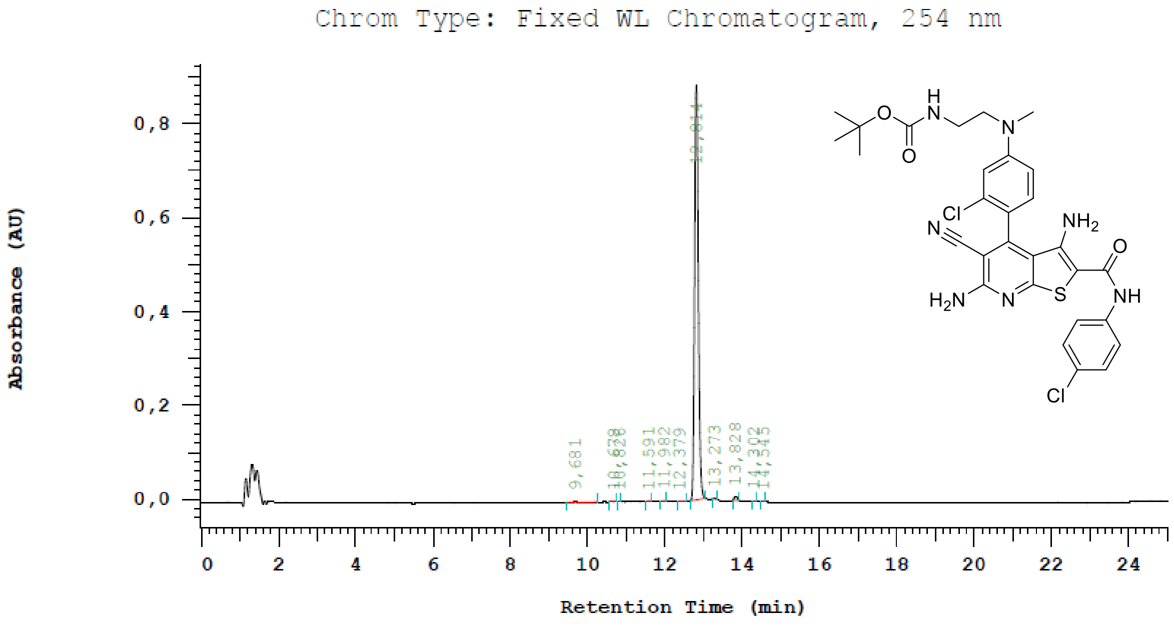

Figure S69: HPLC chromatogram of **9y** (KuSaSch060) – isocratic method (HPLC 1)

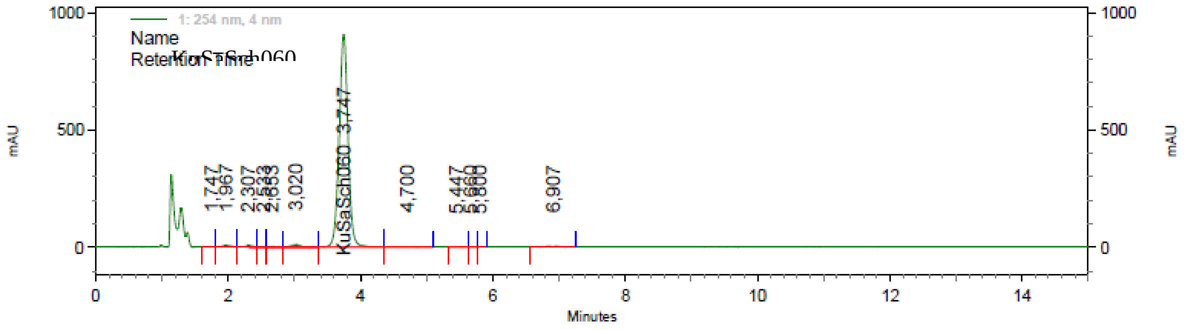

Figure S70: HPLC chromatogram of **9ac** (KuSaSch073) – gradient method (HPLC 3)

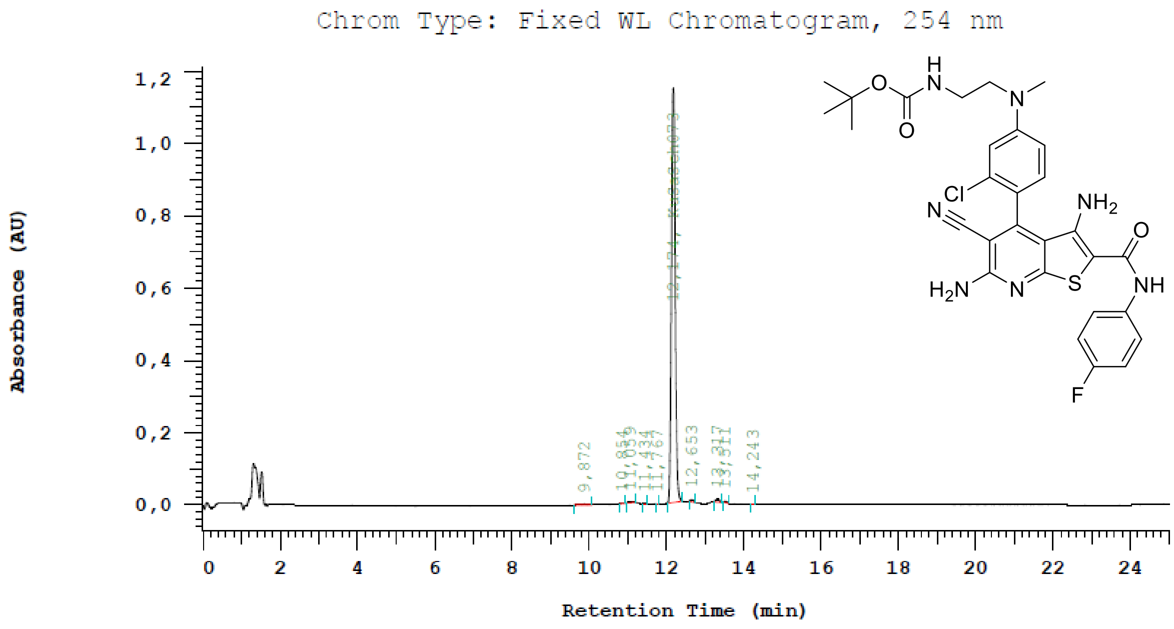

Figure S71: HPLC chromatogram of **9ac** (KuSaSch073) – isocratic method (HPLC 1)

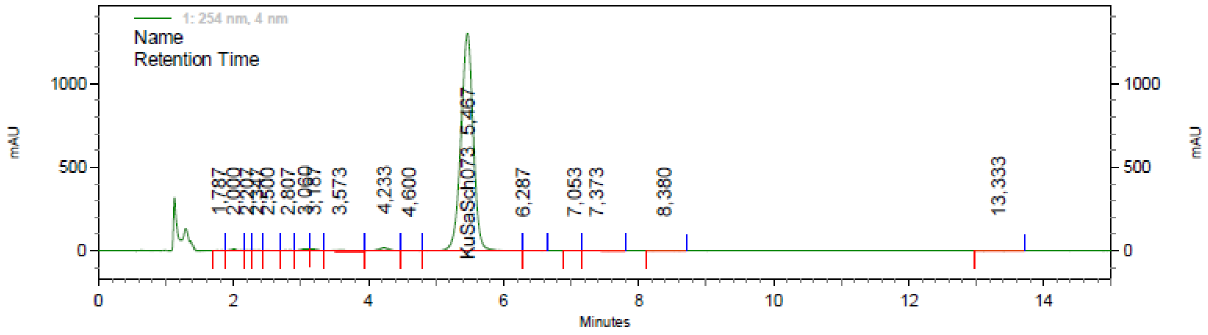

1: 254 nm. 4 nm

Figure S72: HPLC chromatogram of **17a** (KuSaSch095) – gradient method (HPLC 3)

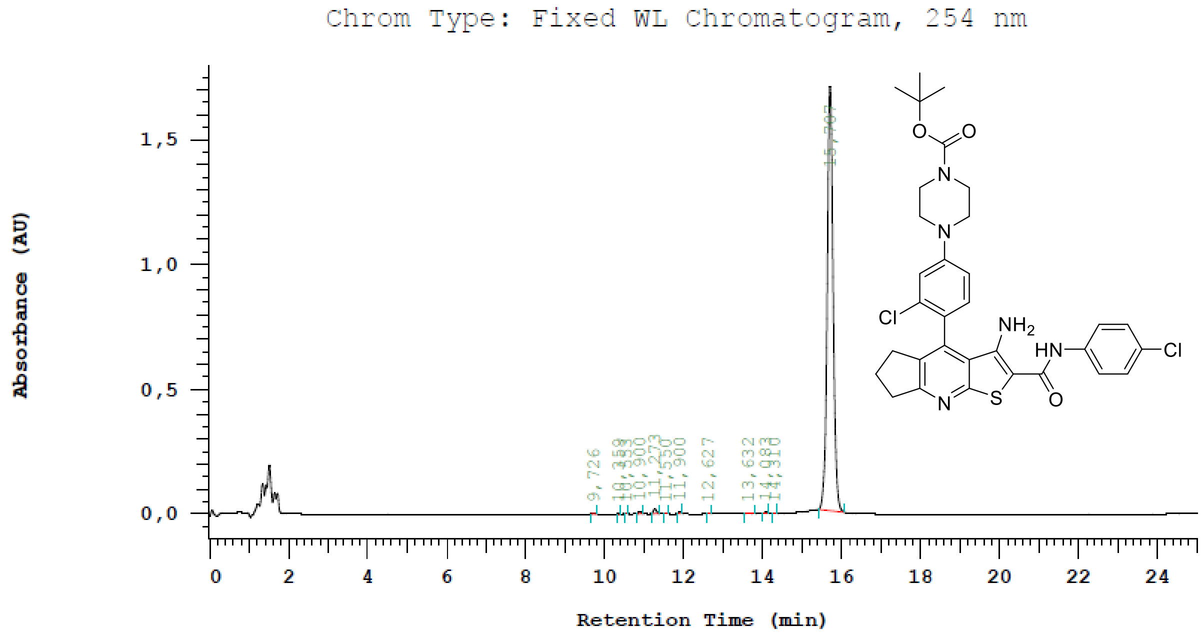

Figure S73: HPLC chromatogram of **17a** (KuSaSch095) – isocratic method (HPLC 1)

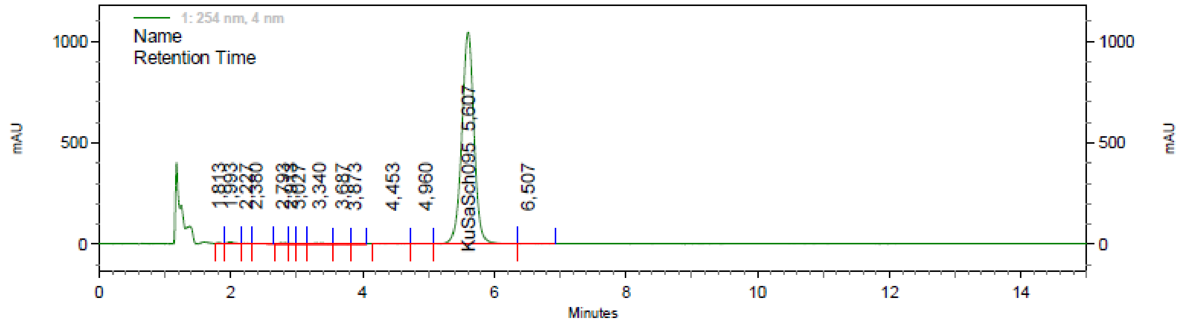

1: 254 nm. 4 nm

Figure S74: HPLC chromatogram of **17b** (KuSaSch100) – gradient method (HPLC 3)

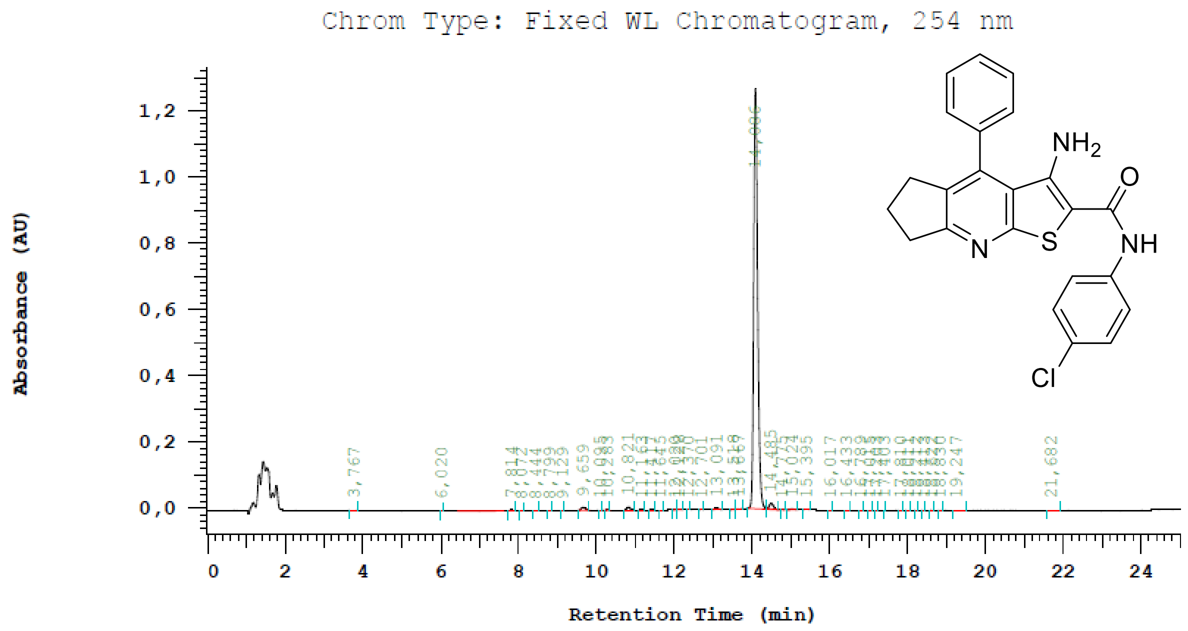

Figure S75: HPLC chromatogram of **17b** (KuSaSch100) – isocratic method (HPLC 1)

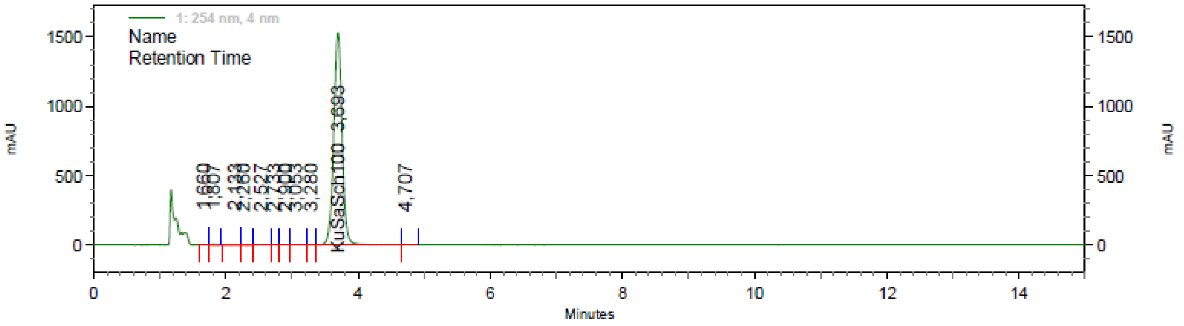

1: 254 nm. 4 nm

Figure S76: HPLC chromatogram of **17f** (KuSaSch110) – gradient method (HPLC 3)

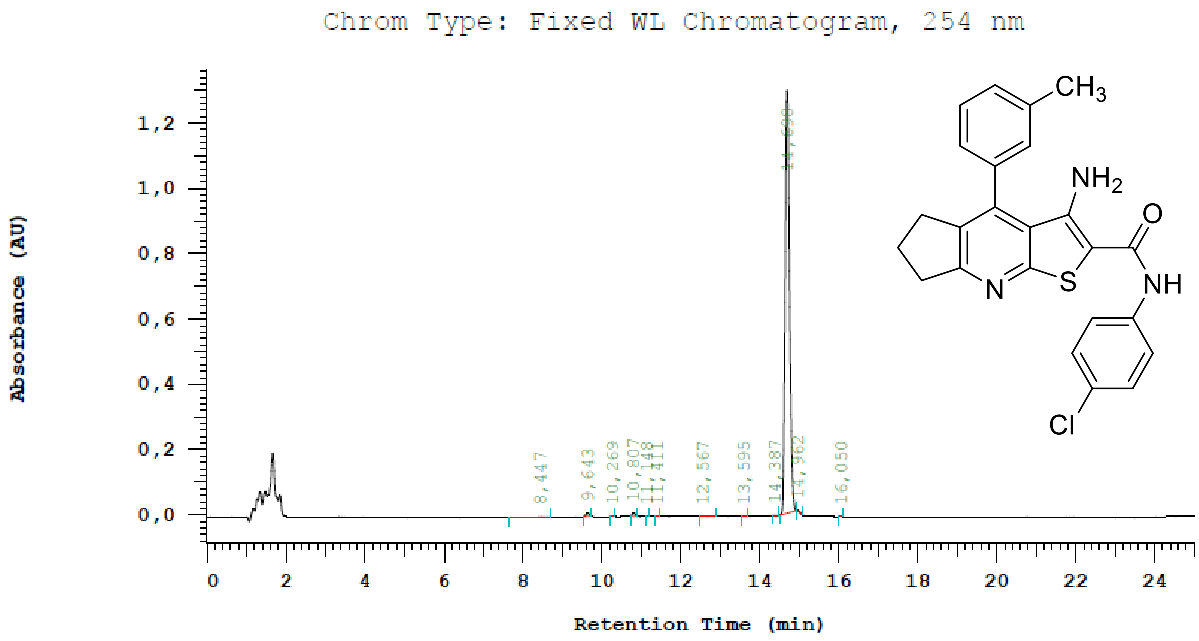

Figure S77: HPLC chromatogram of **17f** (KuSaSch110) – isocratic method (HPLC 1)

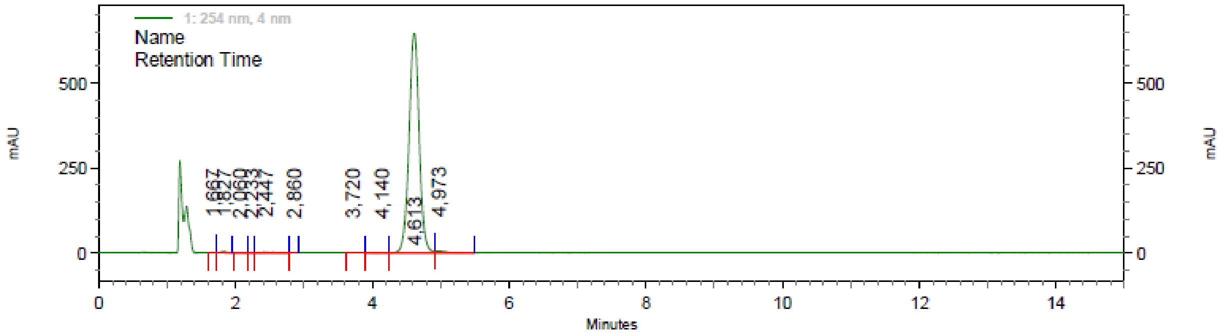

1: 254 nm. 4 nm

Figure S78: HPLC chromatogram of **17g** (KuSaSch111) – gradient method (HPLC 3)

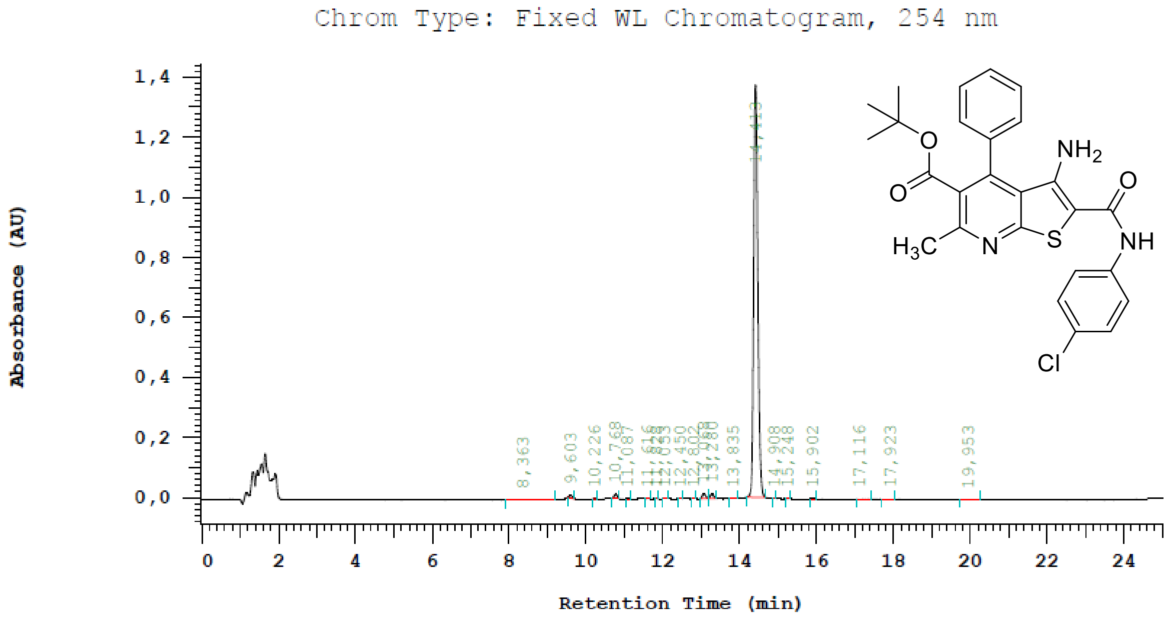

Figure S79: HPLC chromatogram of **17g** (KuSaSch111) – isocratic method (HPLC 1)

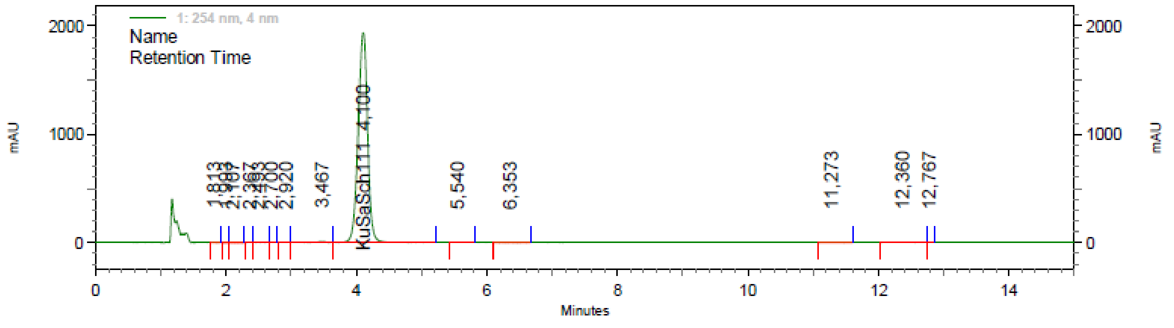

Sample Availability: Samples of the compounds are not available from the authors.

© 2020 by the authors. Submitted for possible open access publication under the terms and conditions of the Creative Commons Attribution (CC BY) license (http://creativecommons.org/licenses/by/4.0/).
